# Supplementary material for: A novel clustering approach to bipartite investor-startup networks
Source: PLoS One. 2023 Jan 5;18(1):e0279780. doi: 10.1371/journal.pone.0279780 (PMC9815571; doi:10.1371/journal.pone.0279780)
Supplement: S1 File — (PDF) [file pone.0279780.s001.pdf]

A novel clustering method for investors in bipartite investor-startup networks (Supplementary Material).

Théophile Carniel[1,2], José Halloy[2], Jean-Michel Dalle[1,3,4]

1 Agoranov, Paris, France
2 Université de Paris, CNRS, LIED UMR 8236, Paris, France
3 Sorbonne Université, Paris, France
4 Ecole Polytechnique, Palaiseau, France

Representative investor of the complete sample

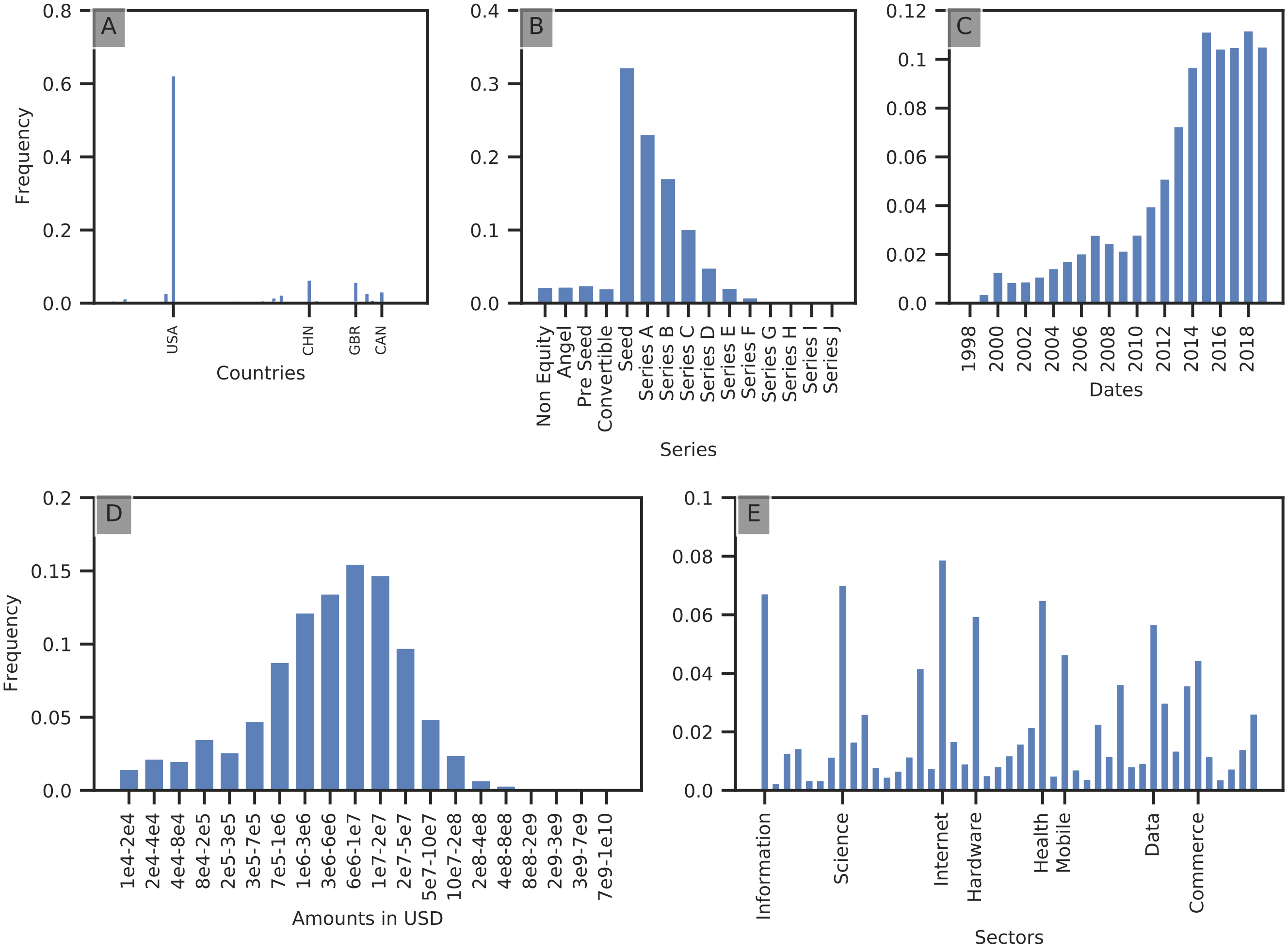

Fig S1. Representative investor of all 1014 investors in our sample.

Clustering robustness analysis

In order to compare the results of the different clusterings, scikit-learn's implementations of the Adjusted Rand Score (ARI) and of the Adjusted Mutual Information (AMI) are used. These two indices measure the similarity between two labels of the same data for different clusterings. An index value of 1 corresponds to identical partitions, an index value of 0 corresponds to two independent labelings. The AMI is used as a control for the more standard ARI as the ARI can be sensitive to strong heterogeneity in terms of cluster sizes (*Adjusting for chance clustering comparison measures*, S. Romano *et al.*, 2016).

The clustering method described is applied 5 separate times, each time computing similarity using 4 of the 5 characteristic dimensions previously defined (see Table **1** in the main text, columns **B** through **F** for a description of the resulting communities). The self-similarity in terms of community allocations between these various clustering is shown through the use of the Adjusted Rand Score and Adjusted Mutual Information indices. As the community detection algorithm used is non-deterministic, two iterations of the same algorithm for a given similarity graph can yield two slightly different sets of community allocations. The ARI and the AMI between the complete clustering (community allocation on a similarity graph computed using all 5 characteristic dimensions) with each of the various alternative clusterings (similarity graph computed using 4 of the 5 characteristic dimensions) and with itself are thus measured over 500 iterations of the community detection algorithm (Fig. S2). Virtually no difference emerges from these computations, suggesting that the alternative clusterings and the complete clustering are in strong agreement in terms of community allocations of the individual investors. The novel clustering method proposed thus shows strong robustness to dimension decimation, with minor differences in community allocation due in part to the non-determinism of the community detection algorithm.

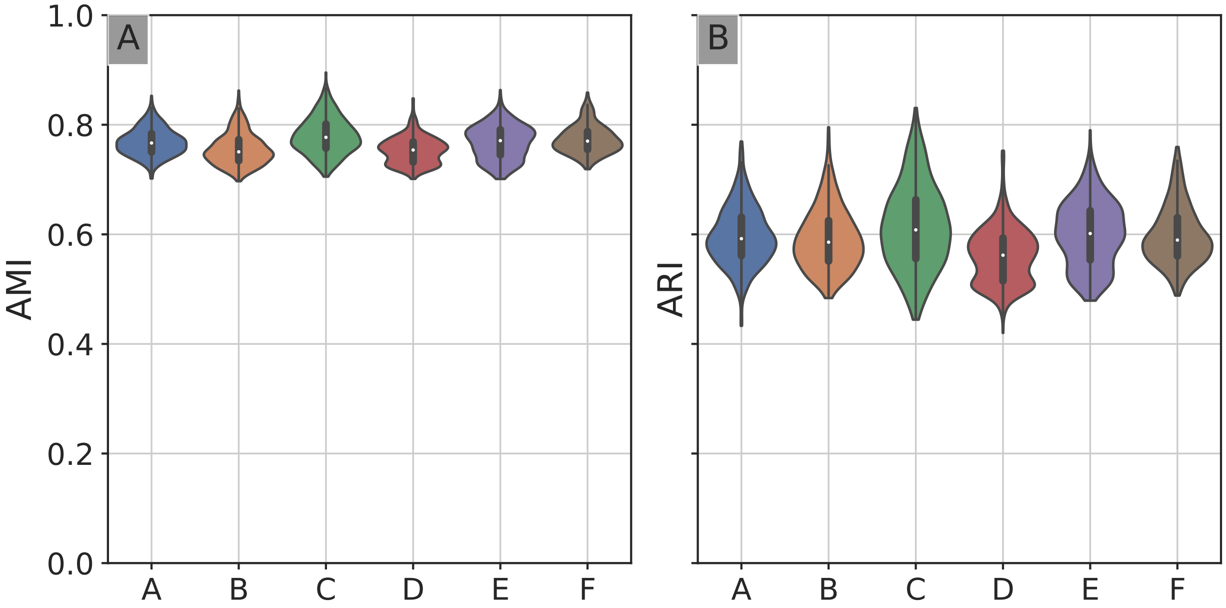

Fig S2. ARI and AMI for the various clusterings. Violin plots of the Adjusted Mutual Information (**A**) and Adjusted Rand Score (**B**) between the complete clustering and the alternative clusterings over 500 iterations. Letters on the x-axis denote the various clusterings as labeled in Table **1**. The clustering labeled A on the x-axis denotes self-similarity of the complete clustering with other iterations of the complete clustering.

Community retention between the various clusterings

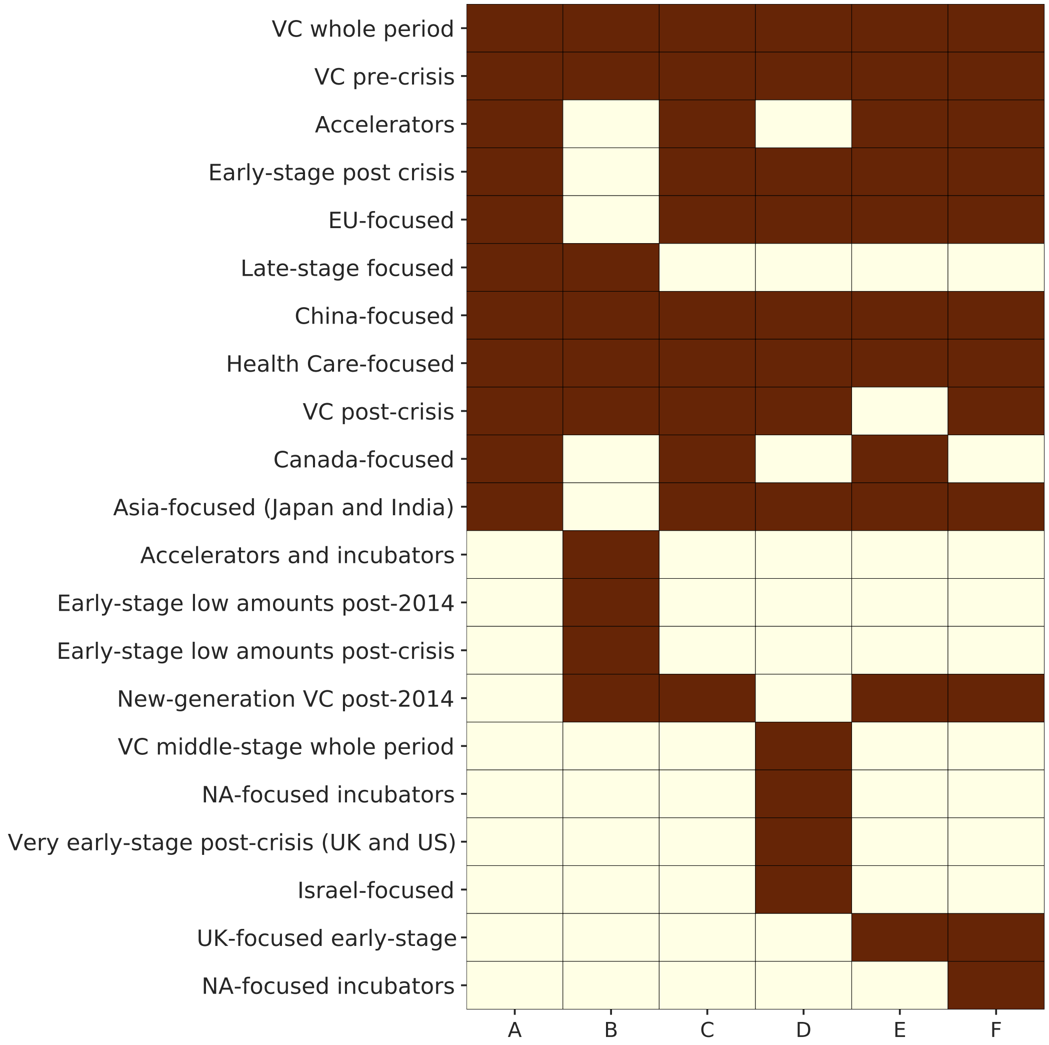

Fig S3. Presence-absence matrix for all communities in the 6 various clusterings. Dark cells denote presence of the community in a given clustering, white cells denote absence of the community.

Representative investors of all communities

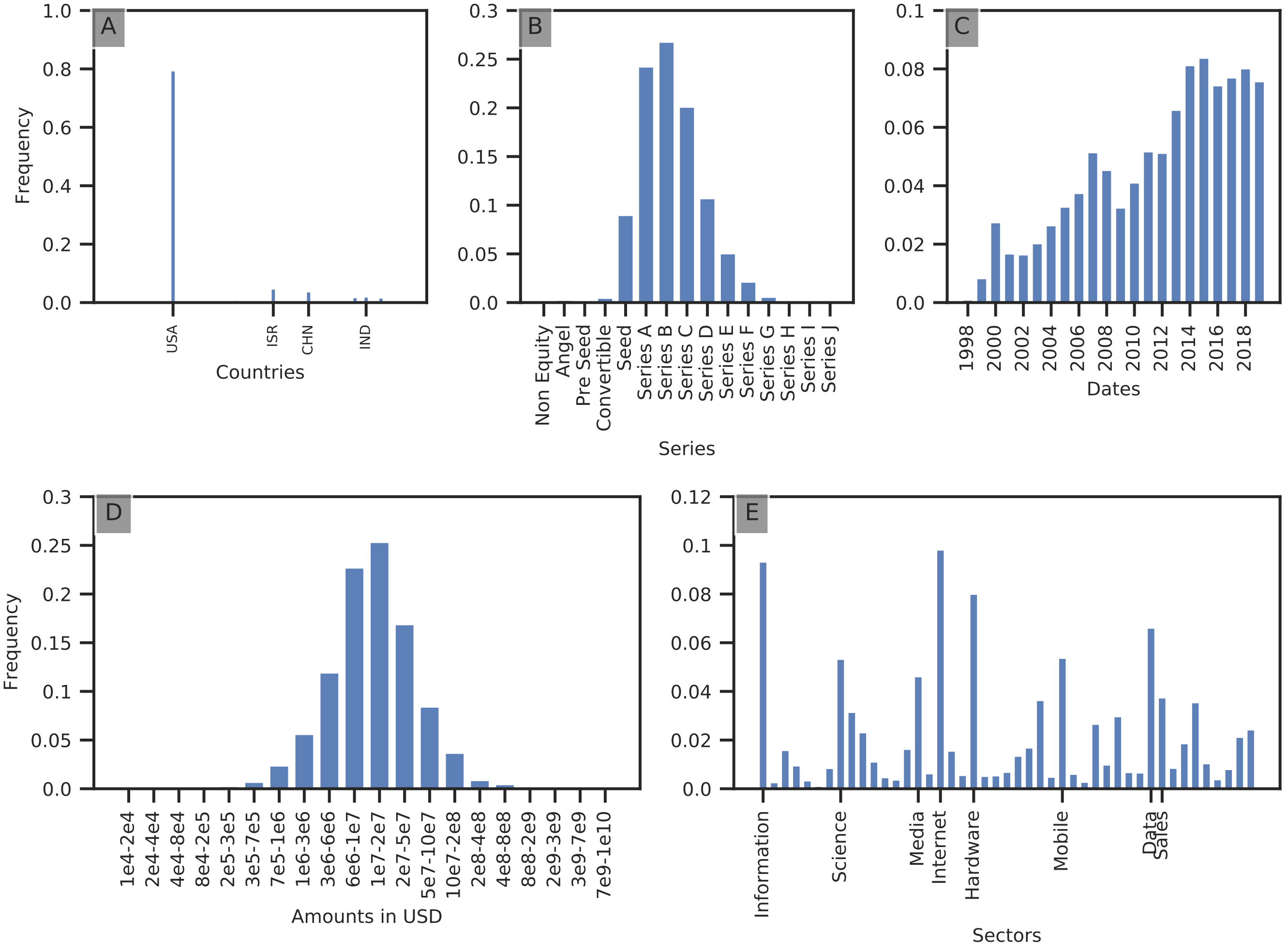

Fig S4. Representative investor of community A0.

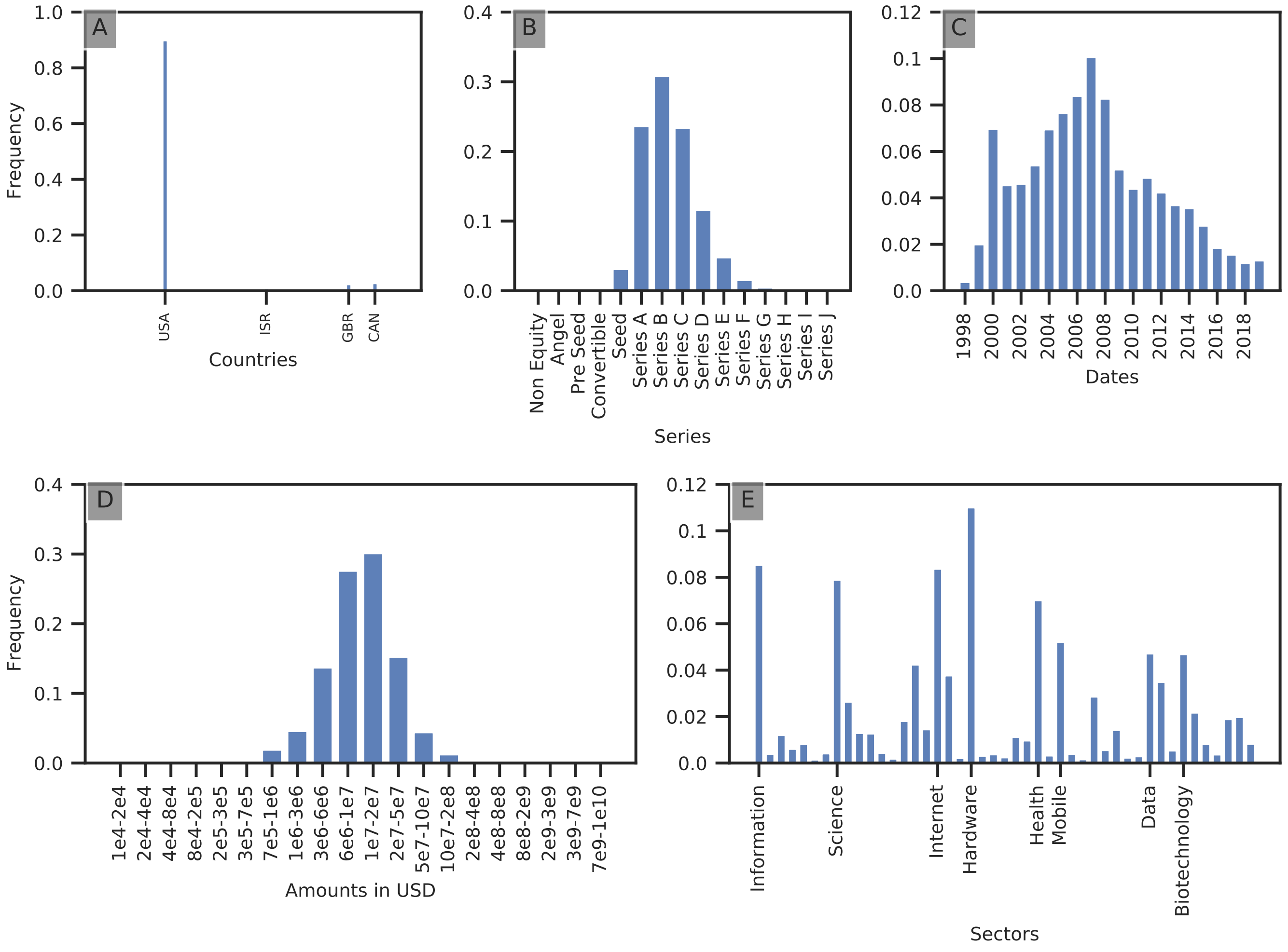

Fig S5. Representative investor of community A1.

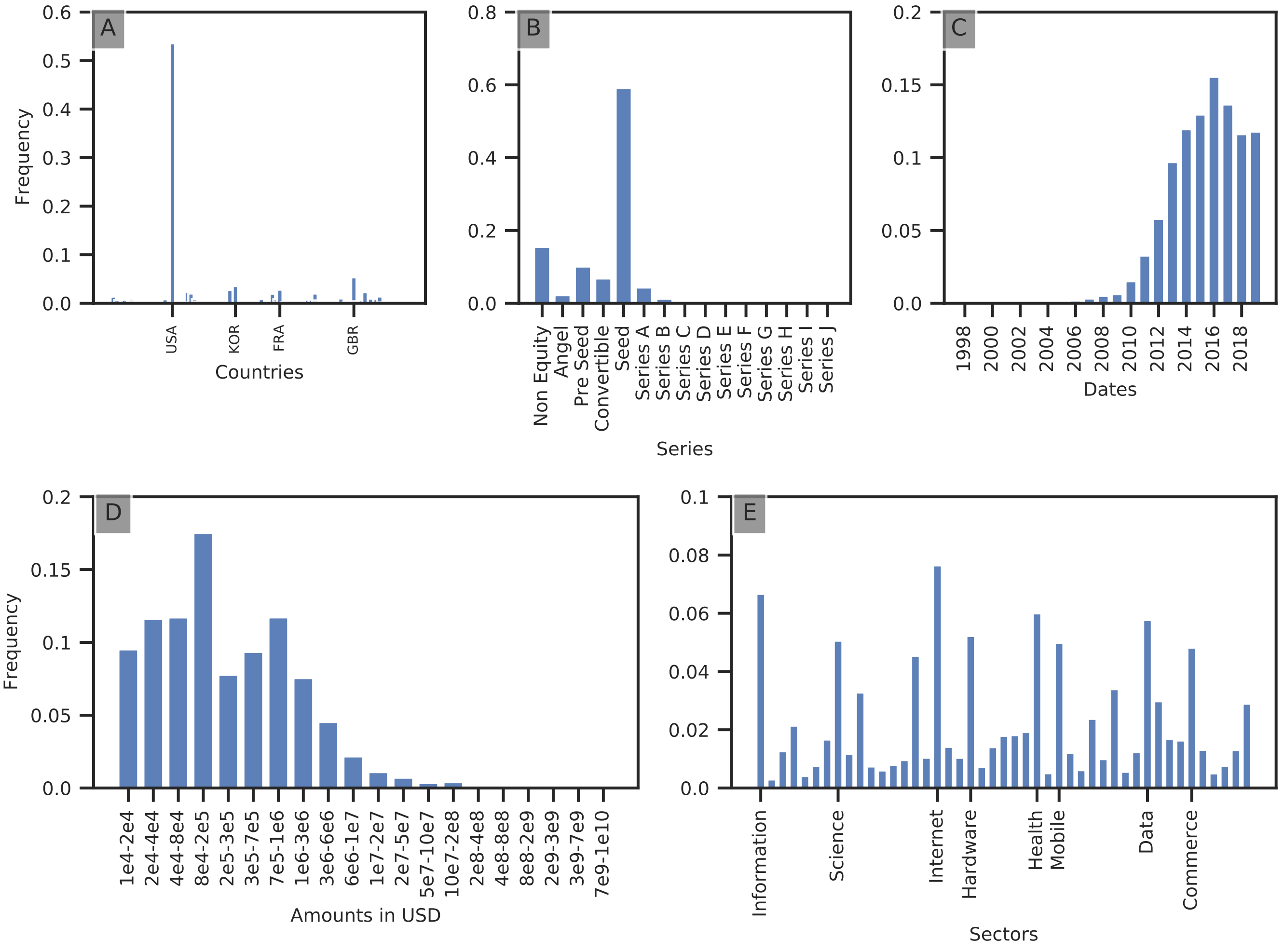

Fig S6. Representative investor of community A2.

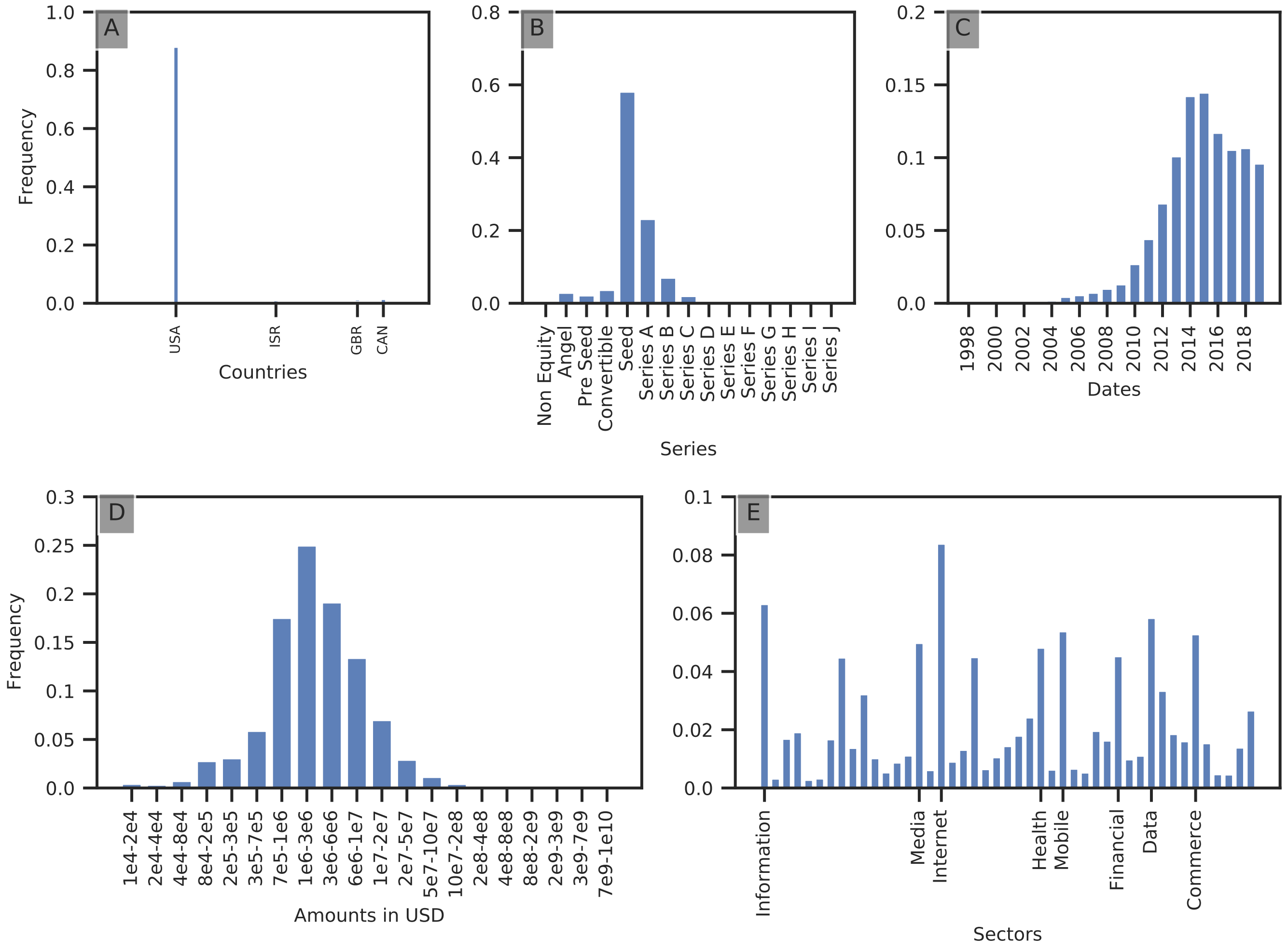

Fig S7. Representative investor of community A3.

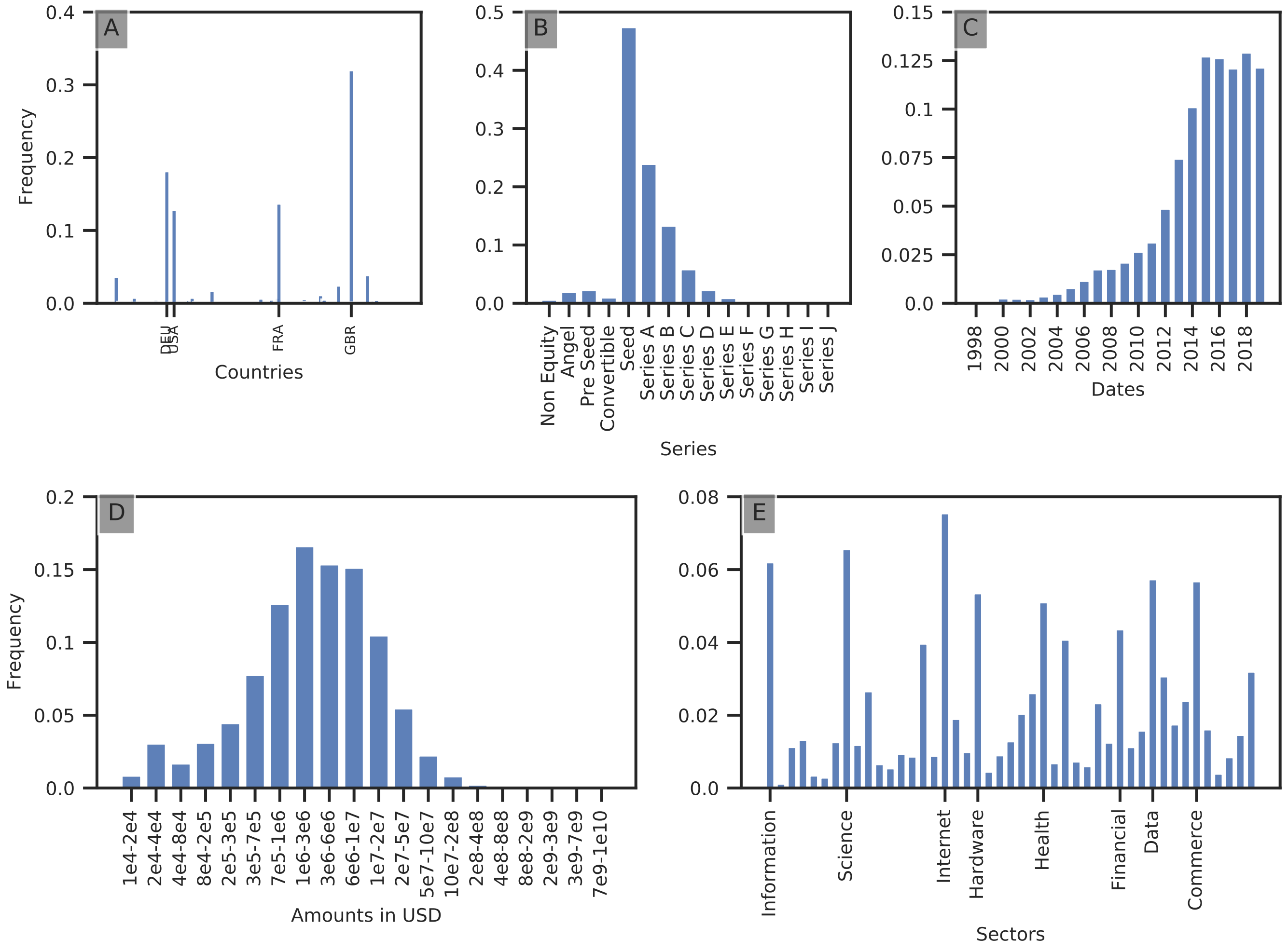

Fig S8. Representative investor of community A4.

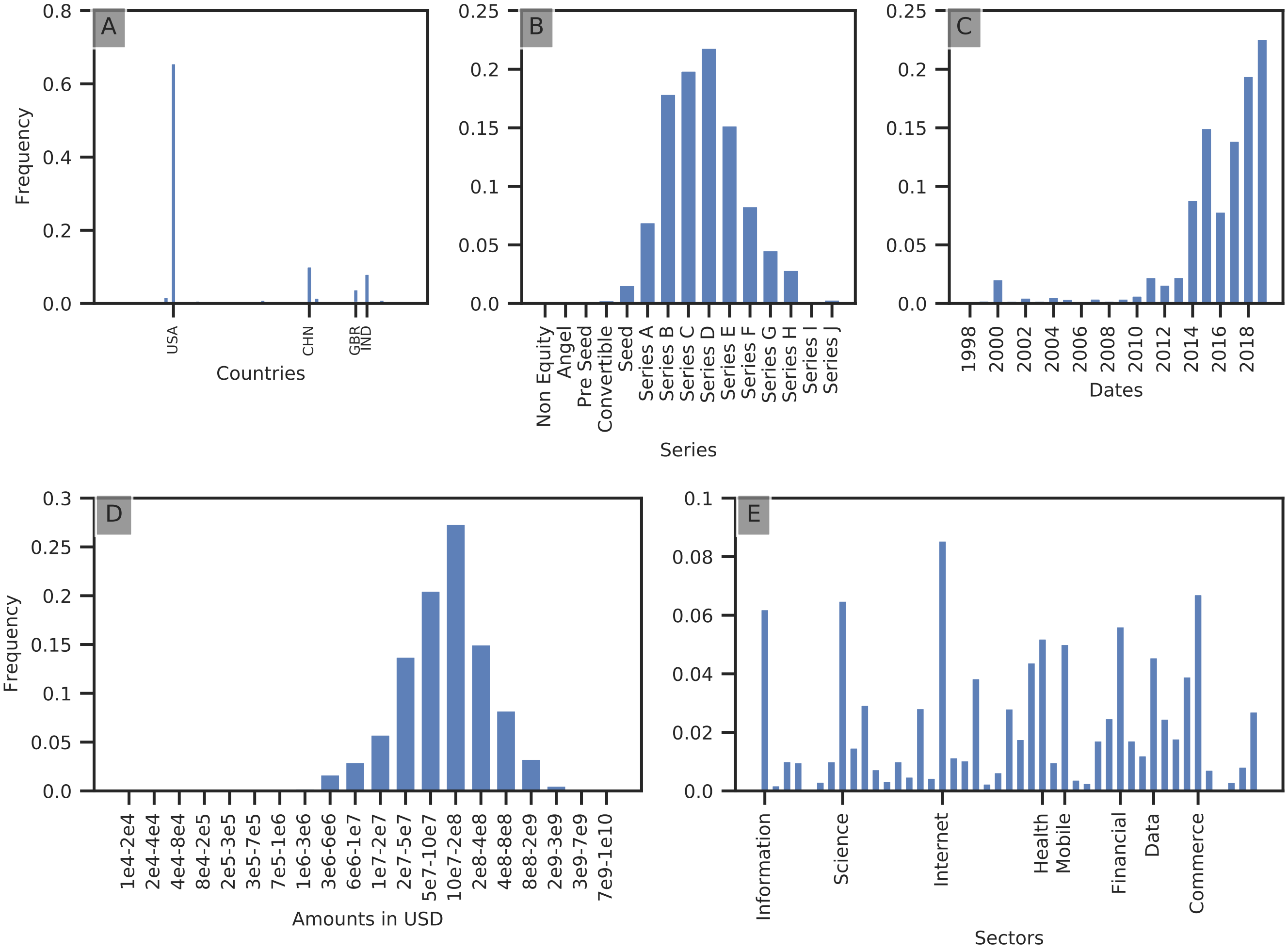

Fig S9. Representative investor of community A5.

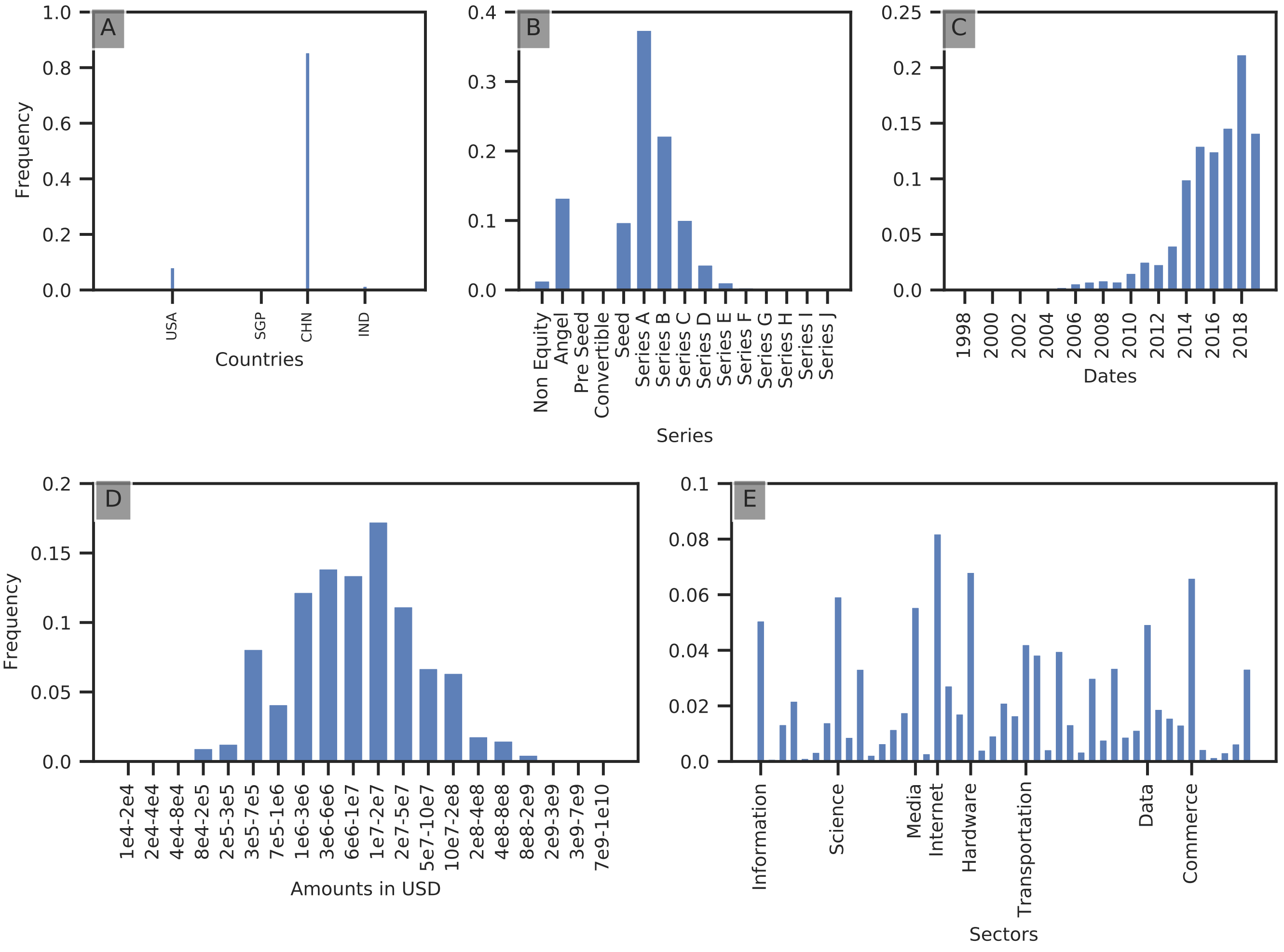

Fig S10. Representative investor of community A6.

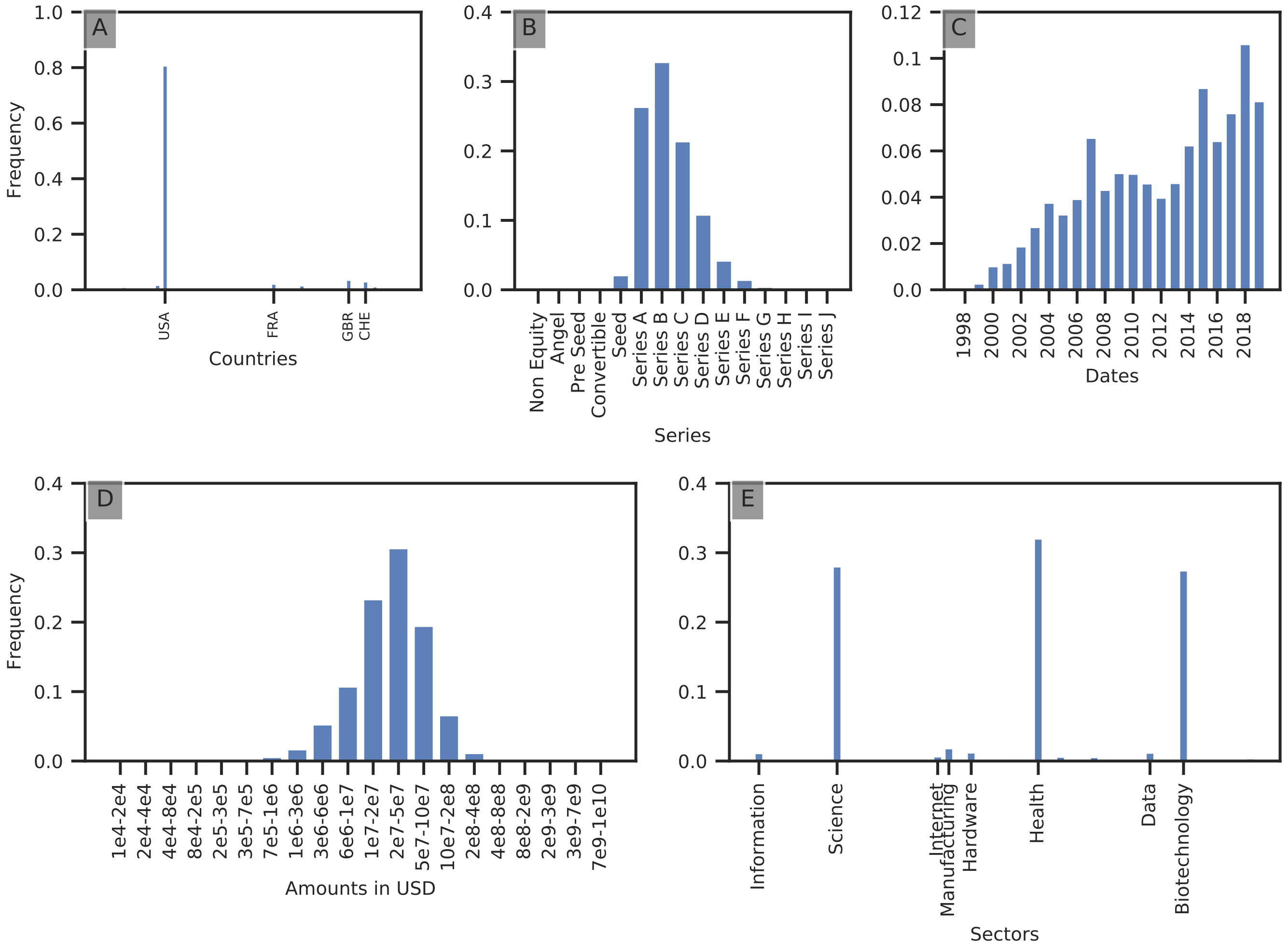

Fig S11. Representative investor of community A7.

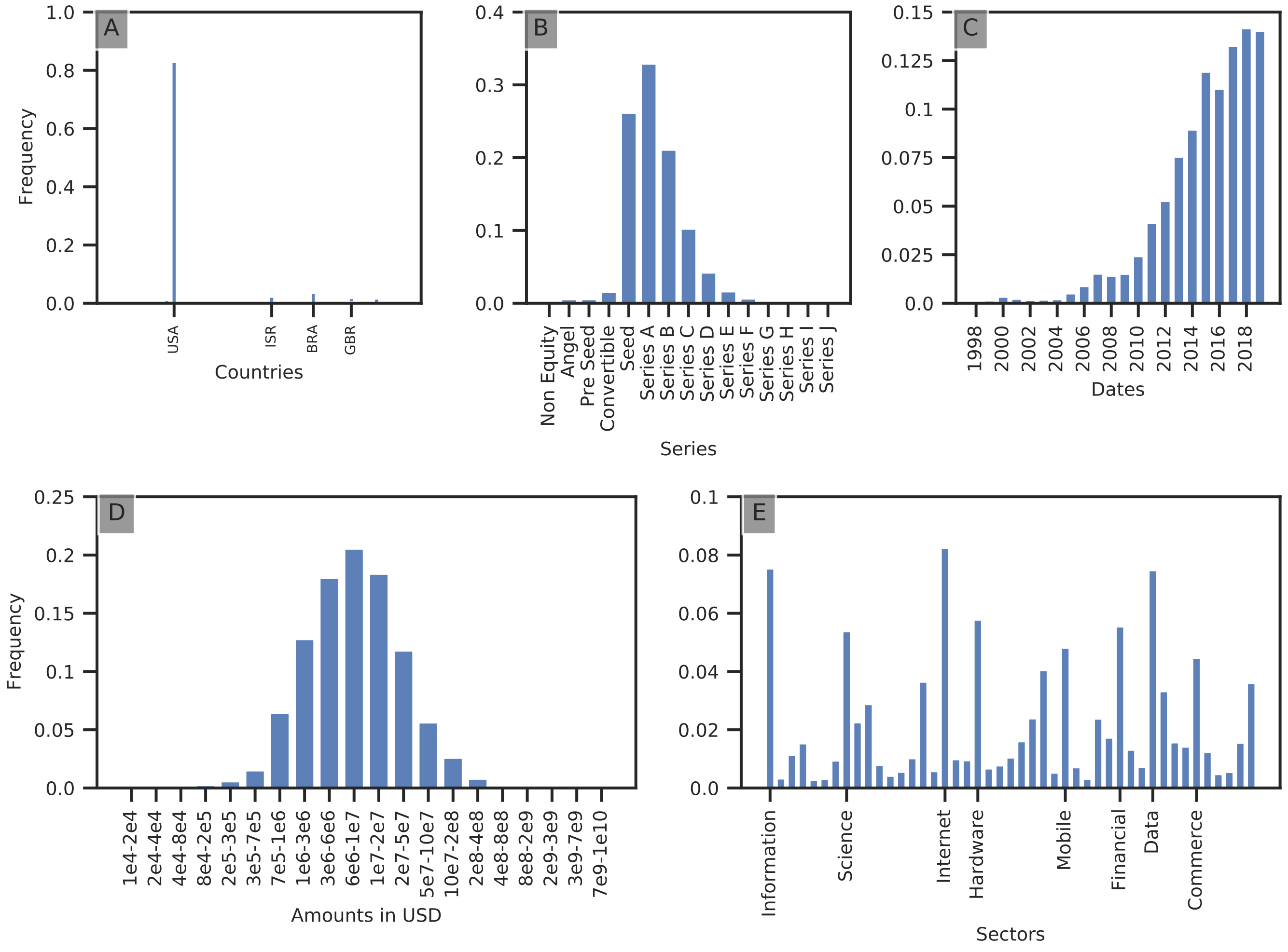

Fig S12. Representative investor of community A8.

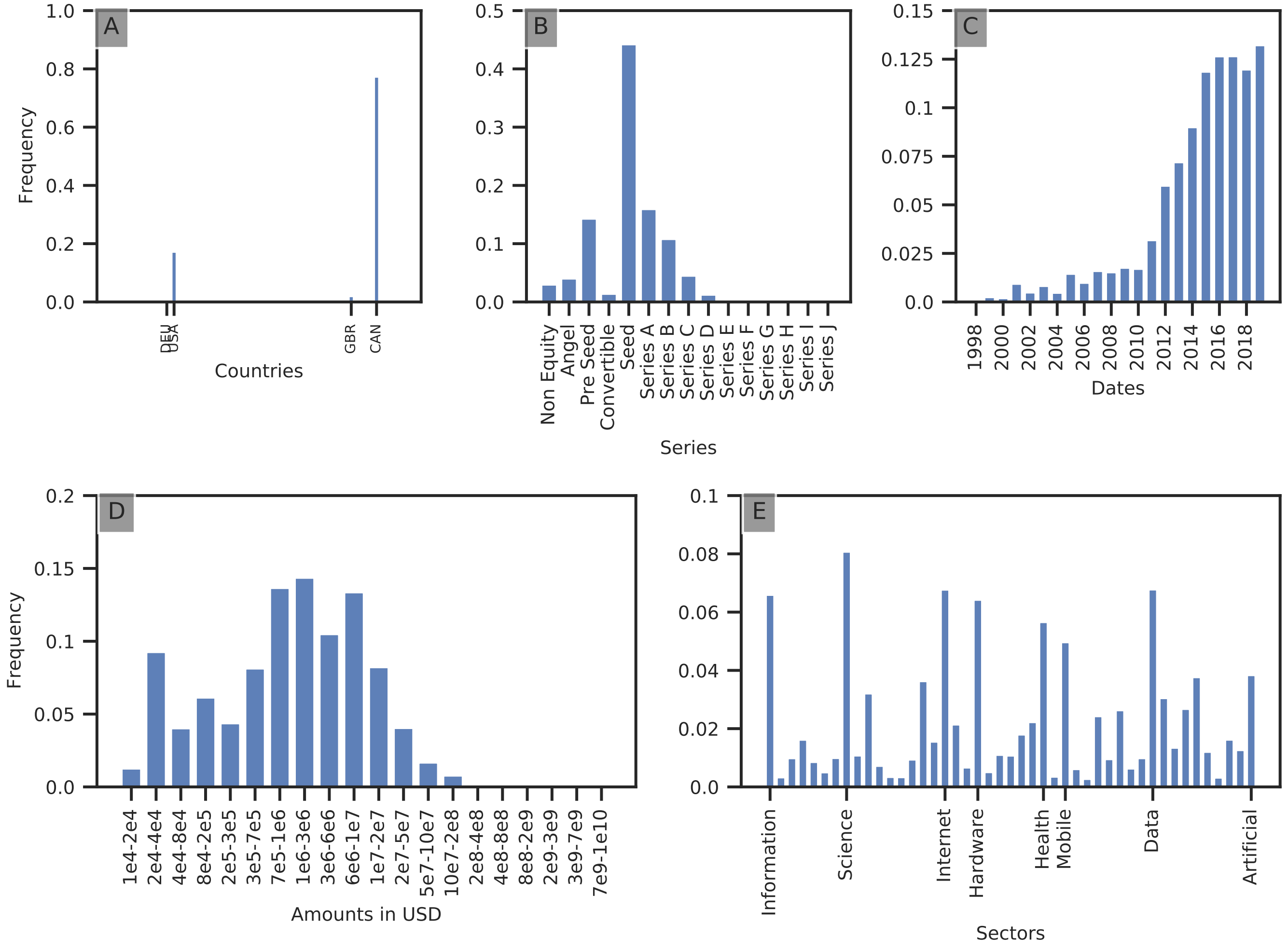

Fig S13. Representative investor of community A9.

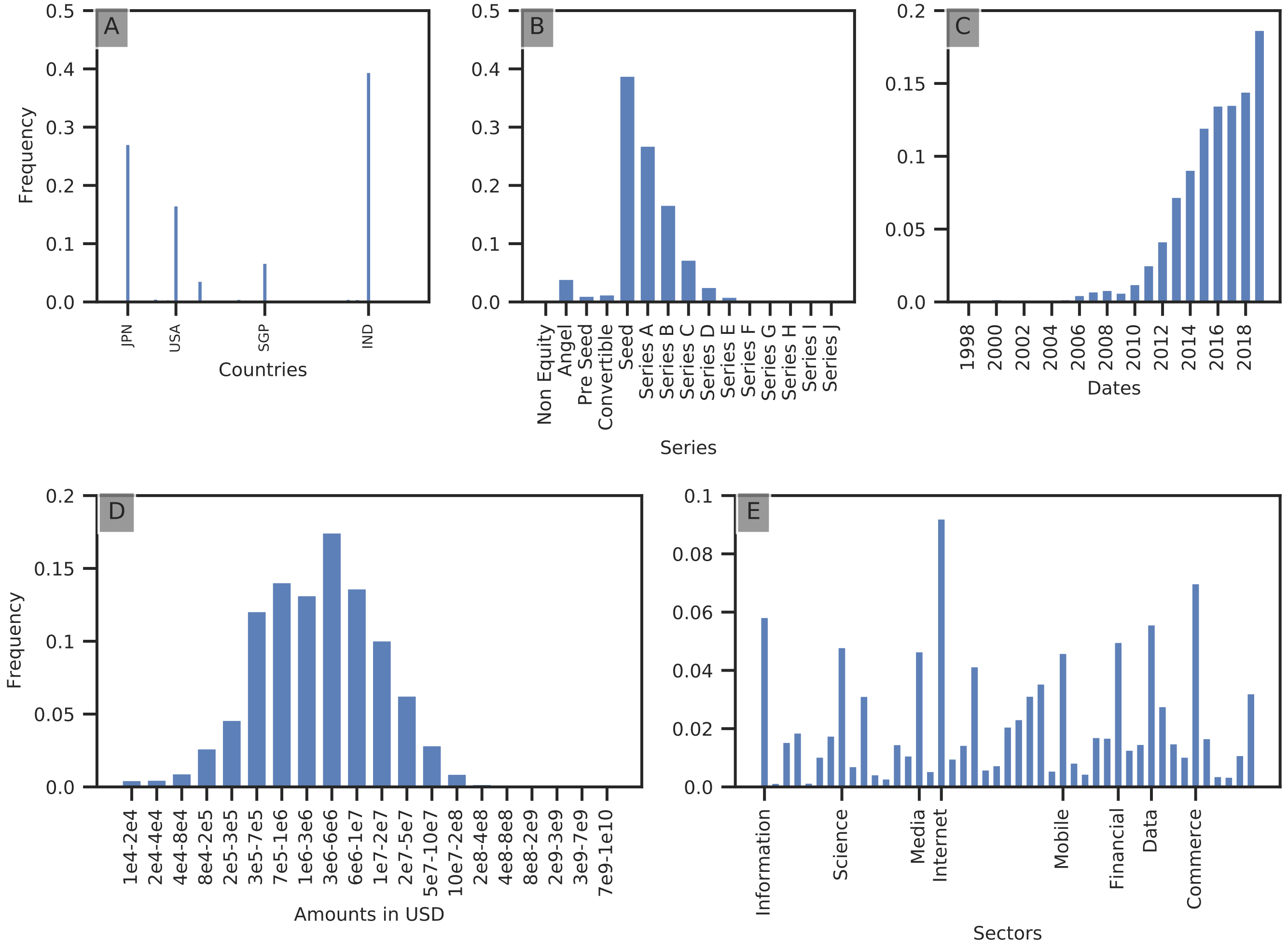

Fig S14. Representative investor of community A10.

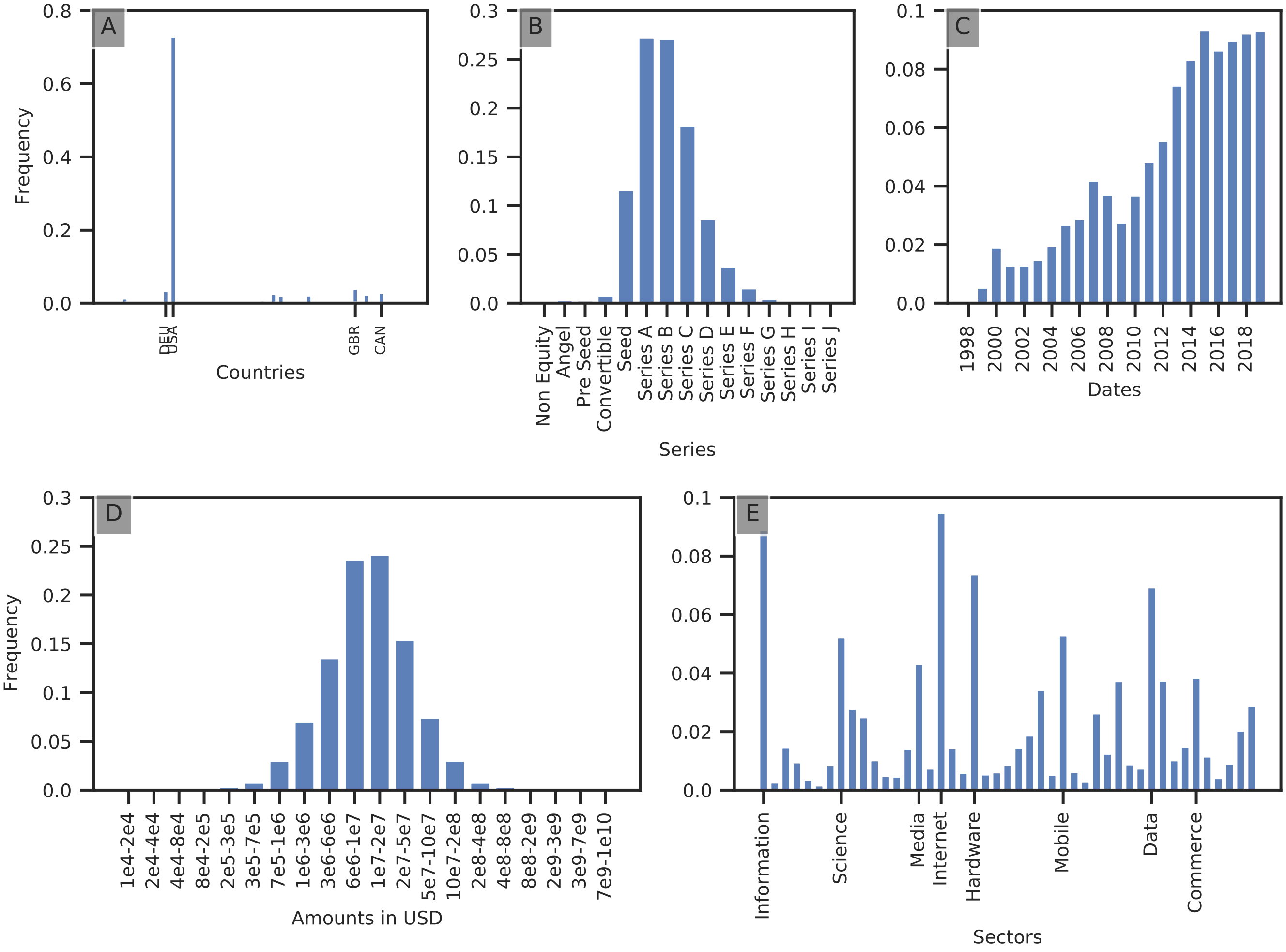

Fig S15. Representative investor of community B0.

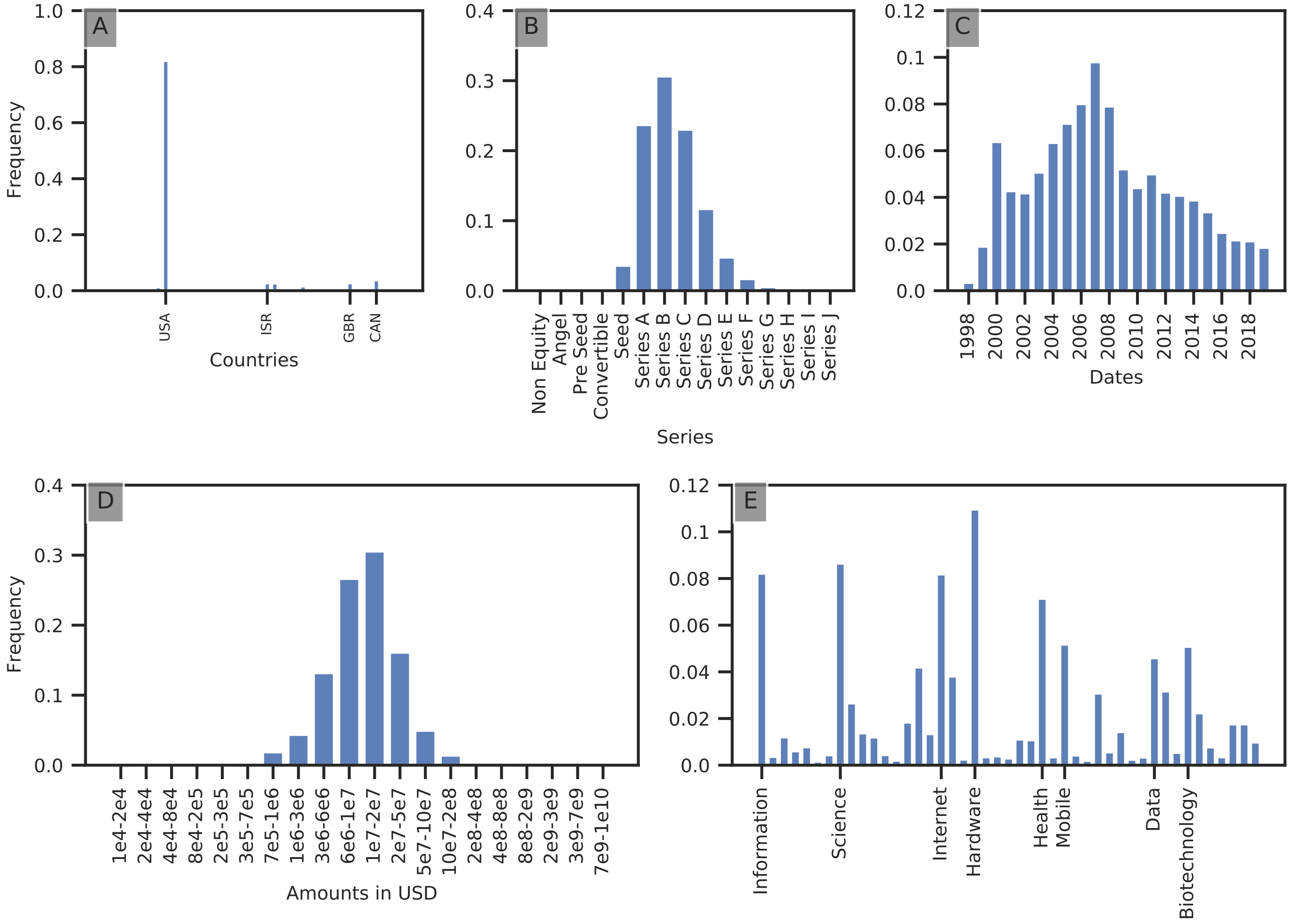

Fig S16. Representative investor of community B1.

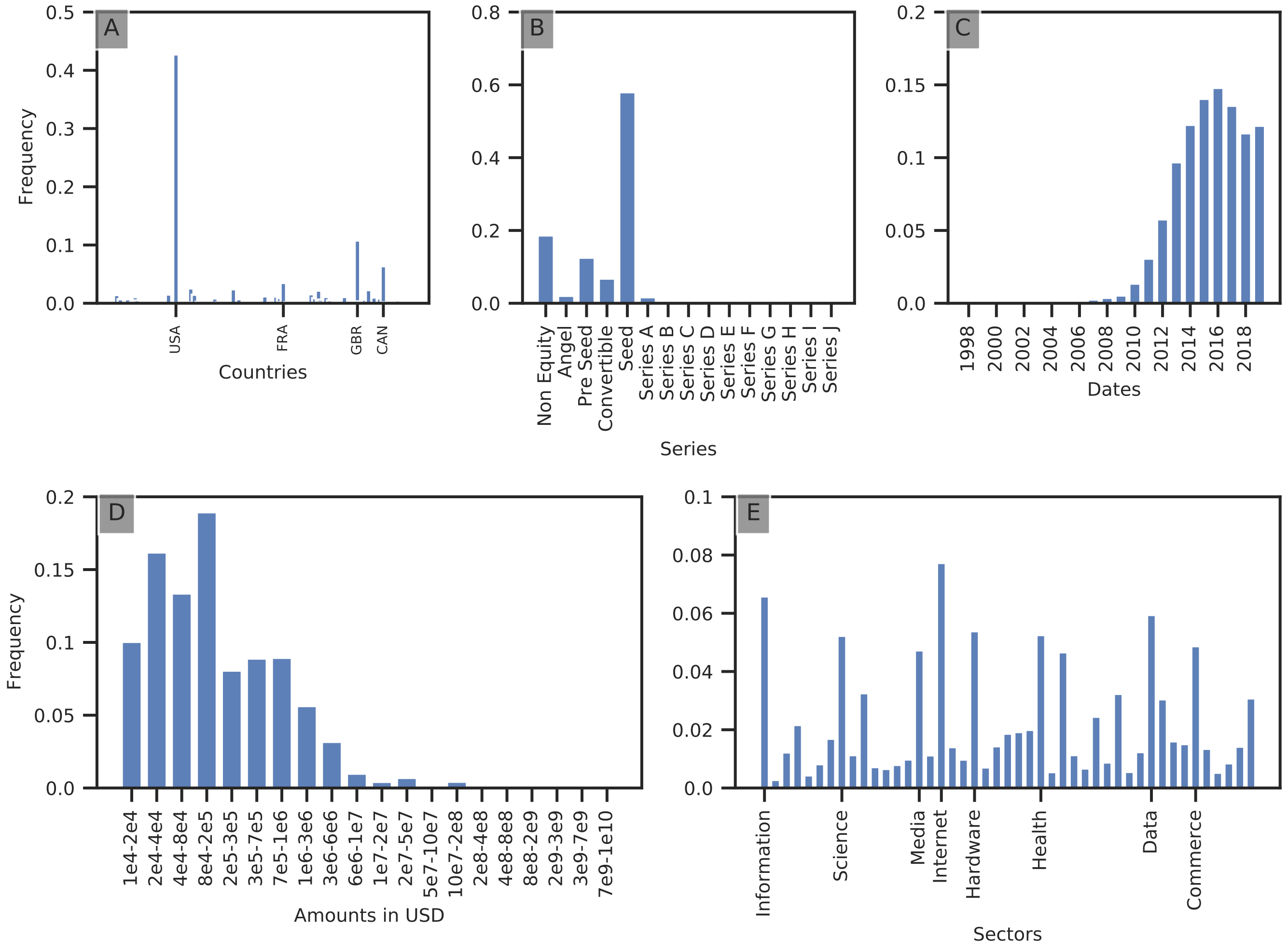

Fig S17. Representative investor of community B2.

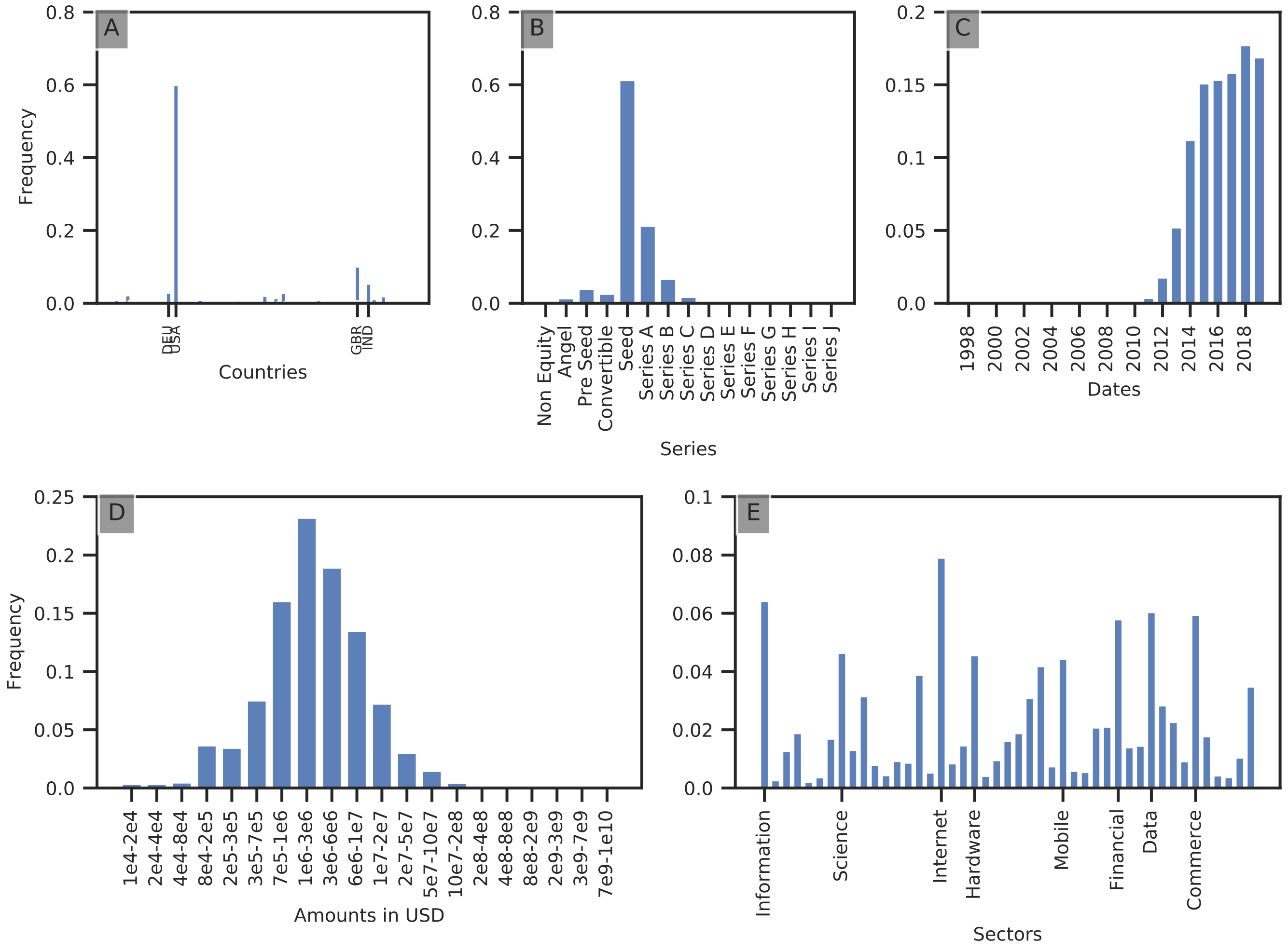

Fig S18. Representative investor of community B3.

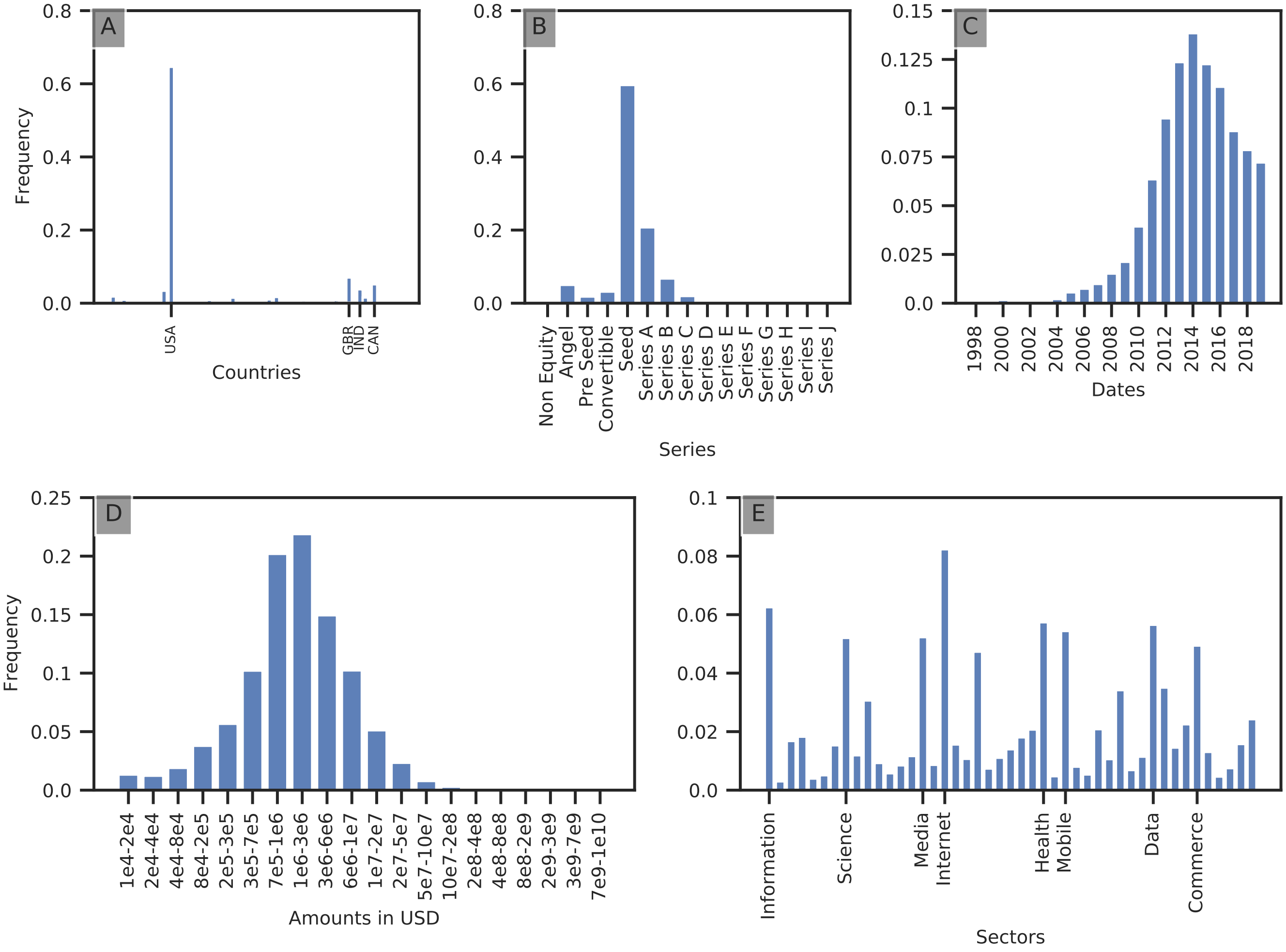

Fig S19. Representative investor of community B4.

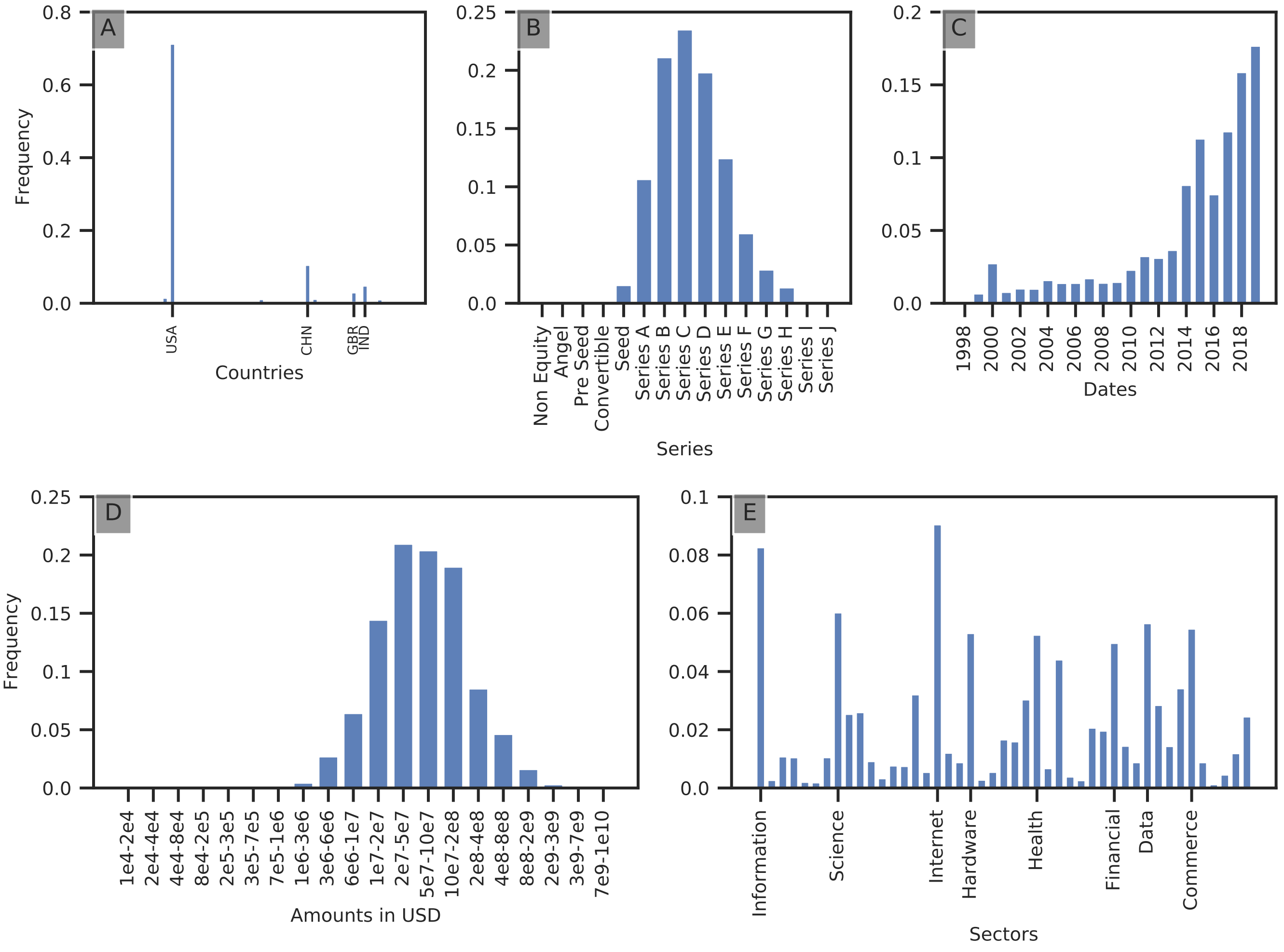

Fig S20. Representative investor of community B5.

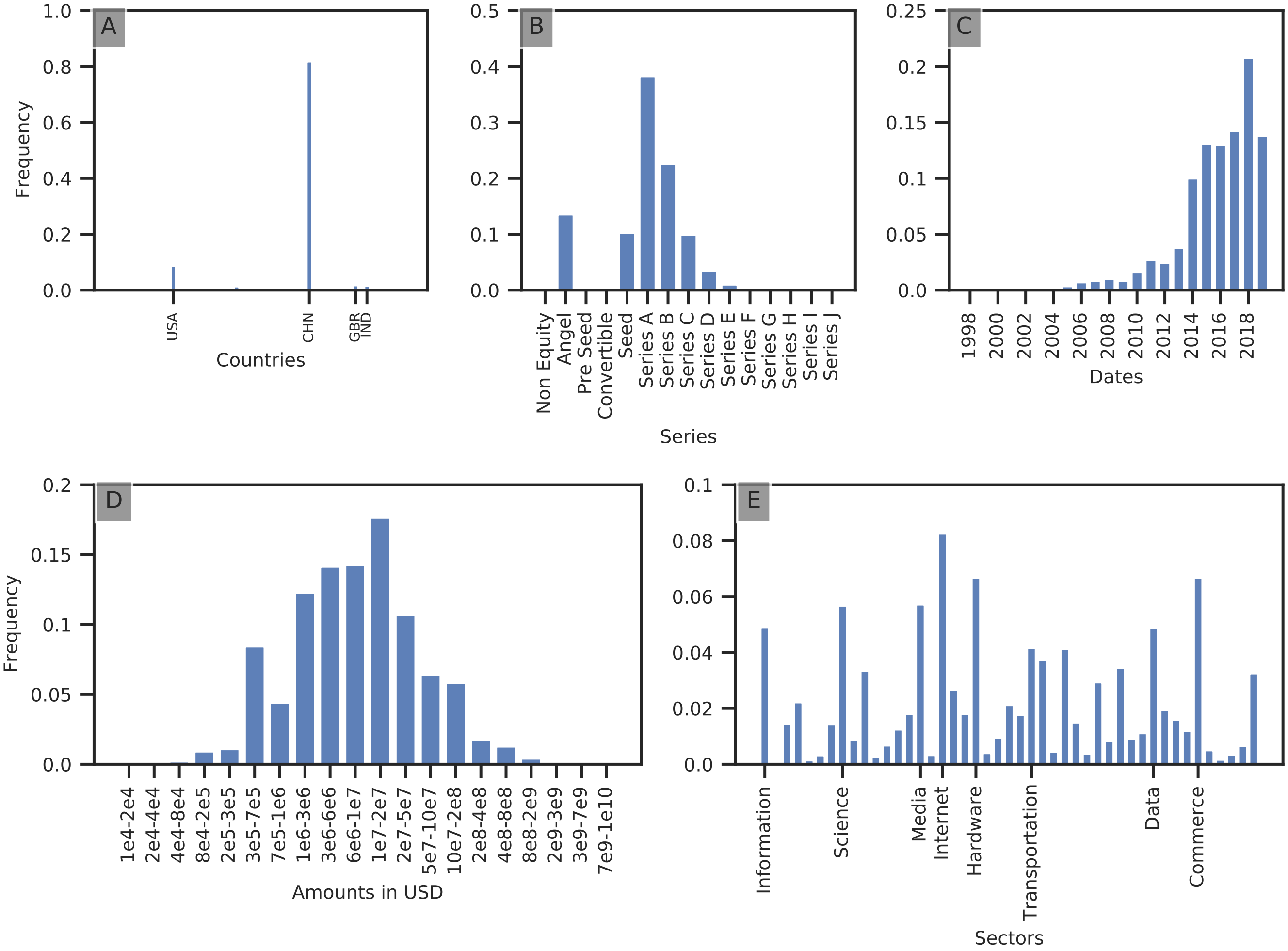

Fig S21. Representative investor of community B6.

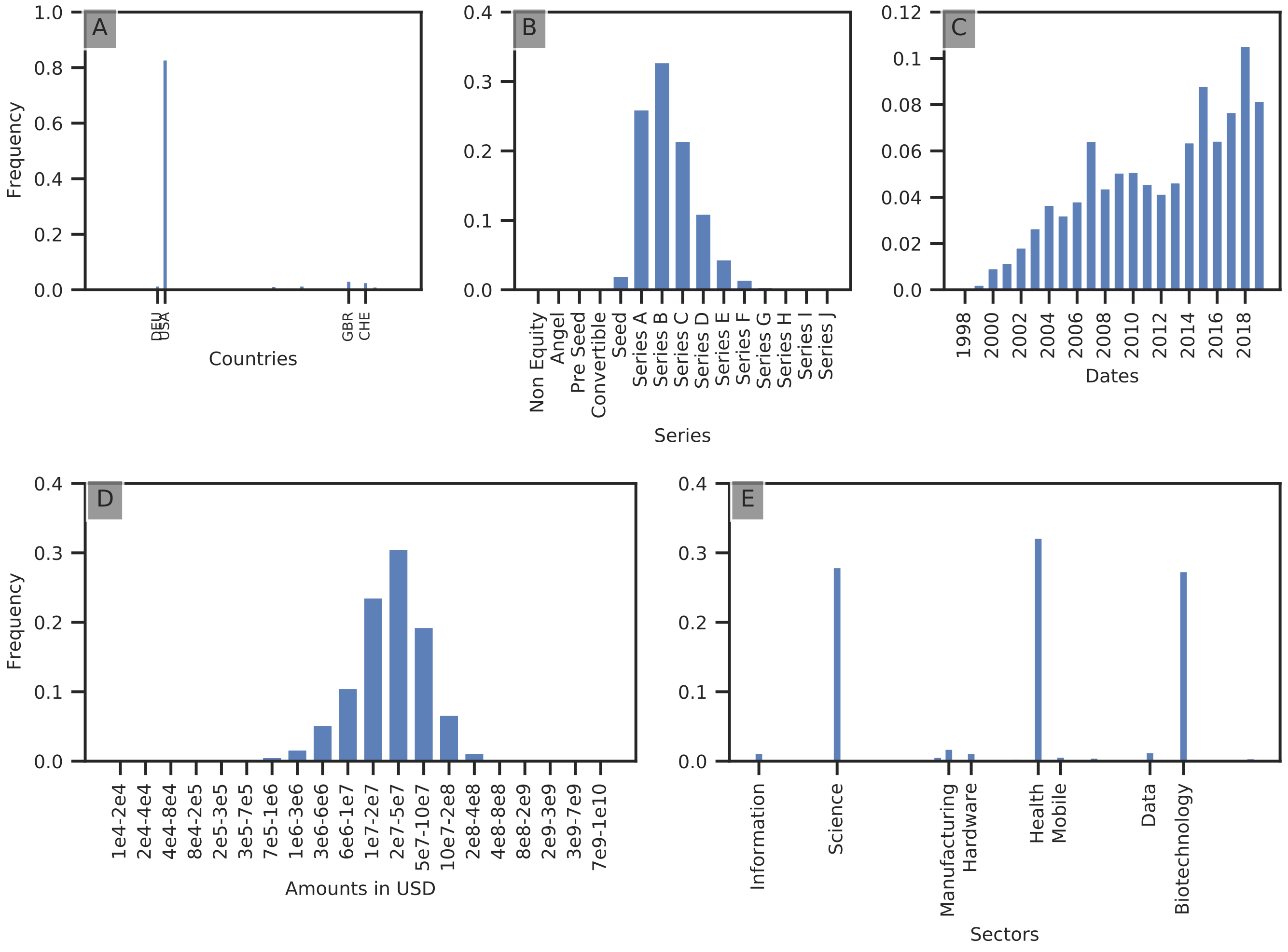

Fig S22. Representative investor of community B7.

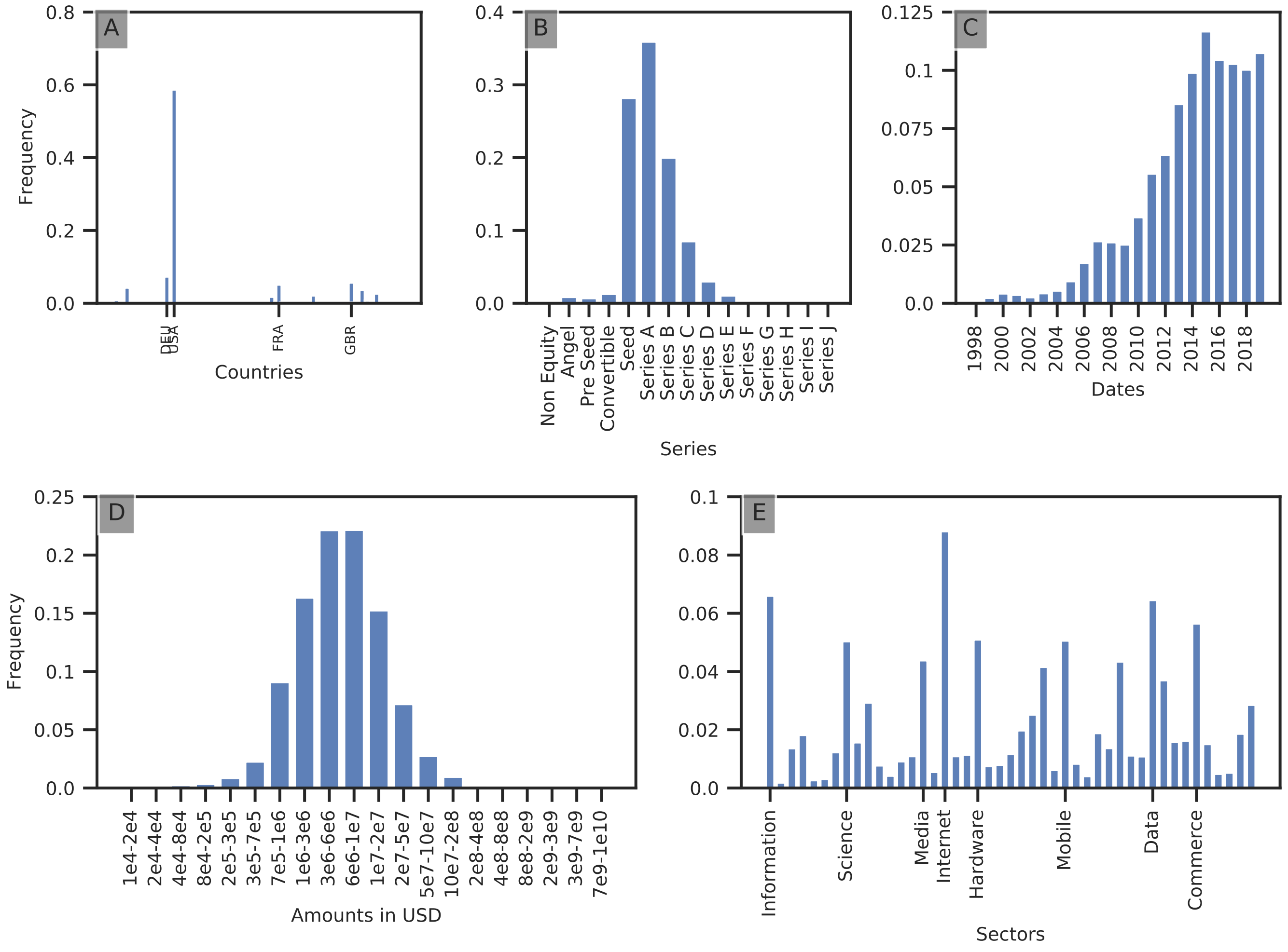

Fig S23. Representative investor of community B8.

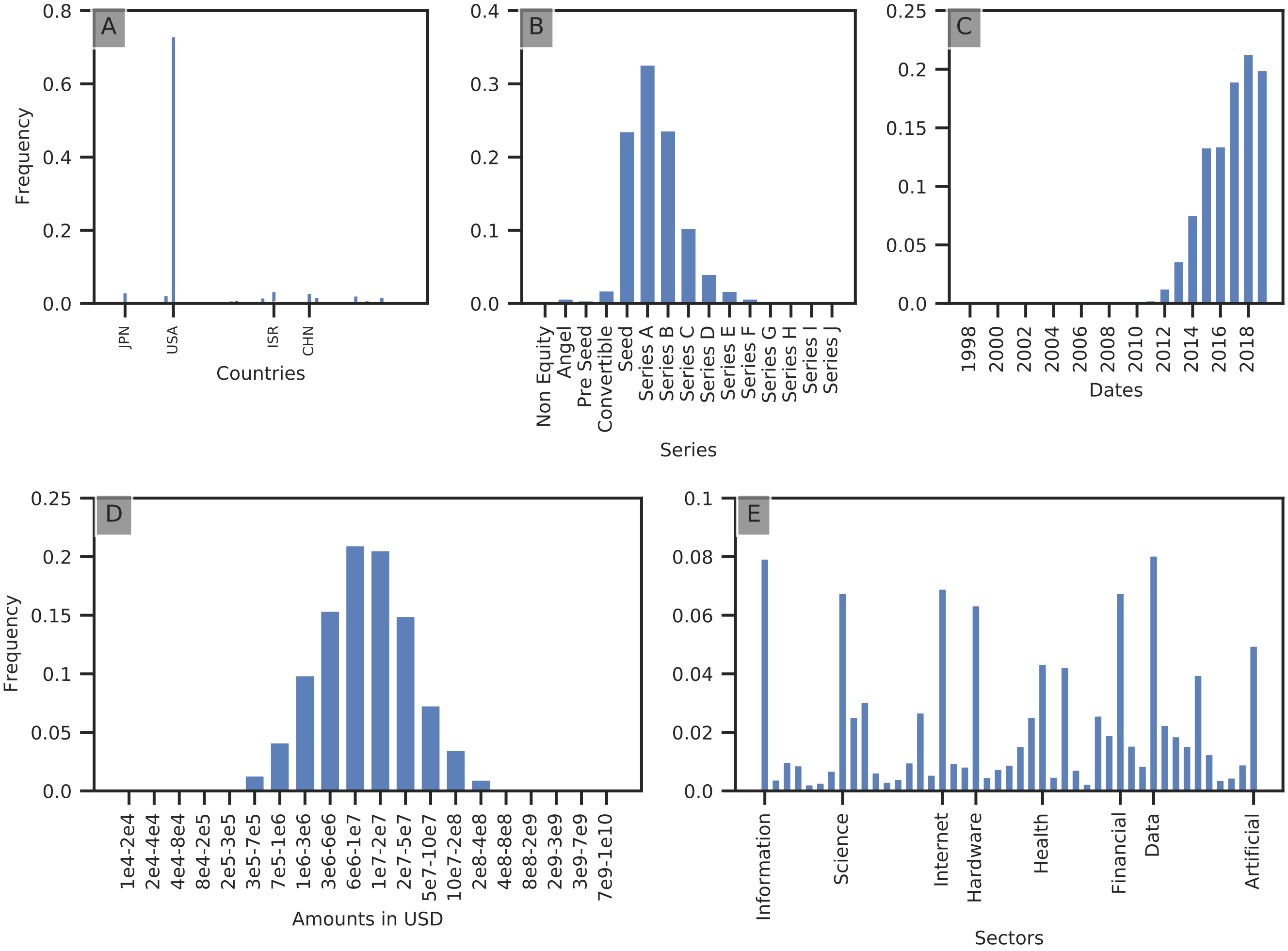

Fig S24. Representative investor of community B9.

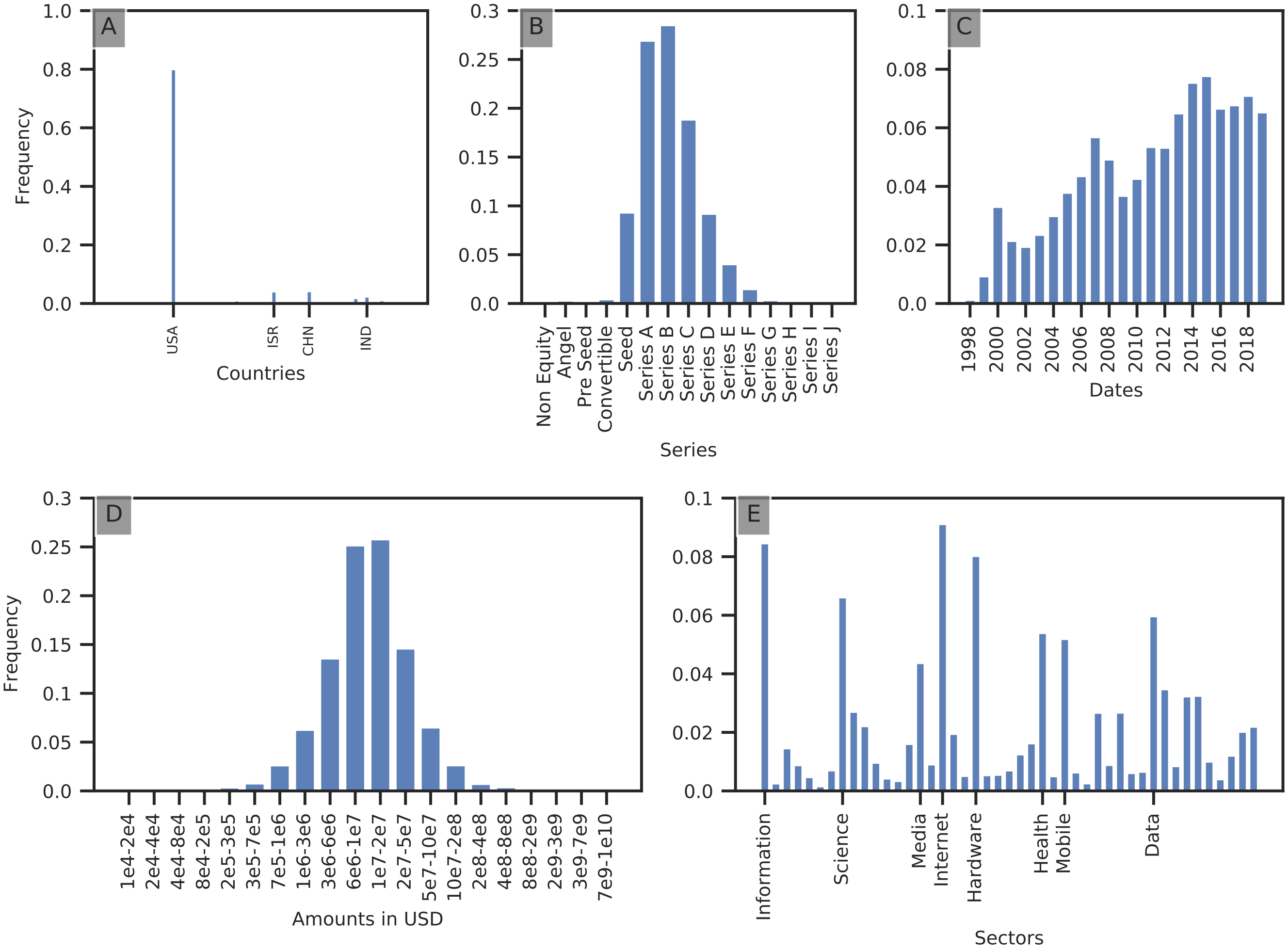

Fig S25. Representative investor of community C0.

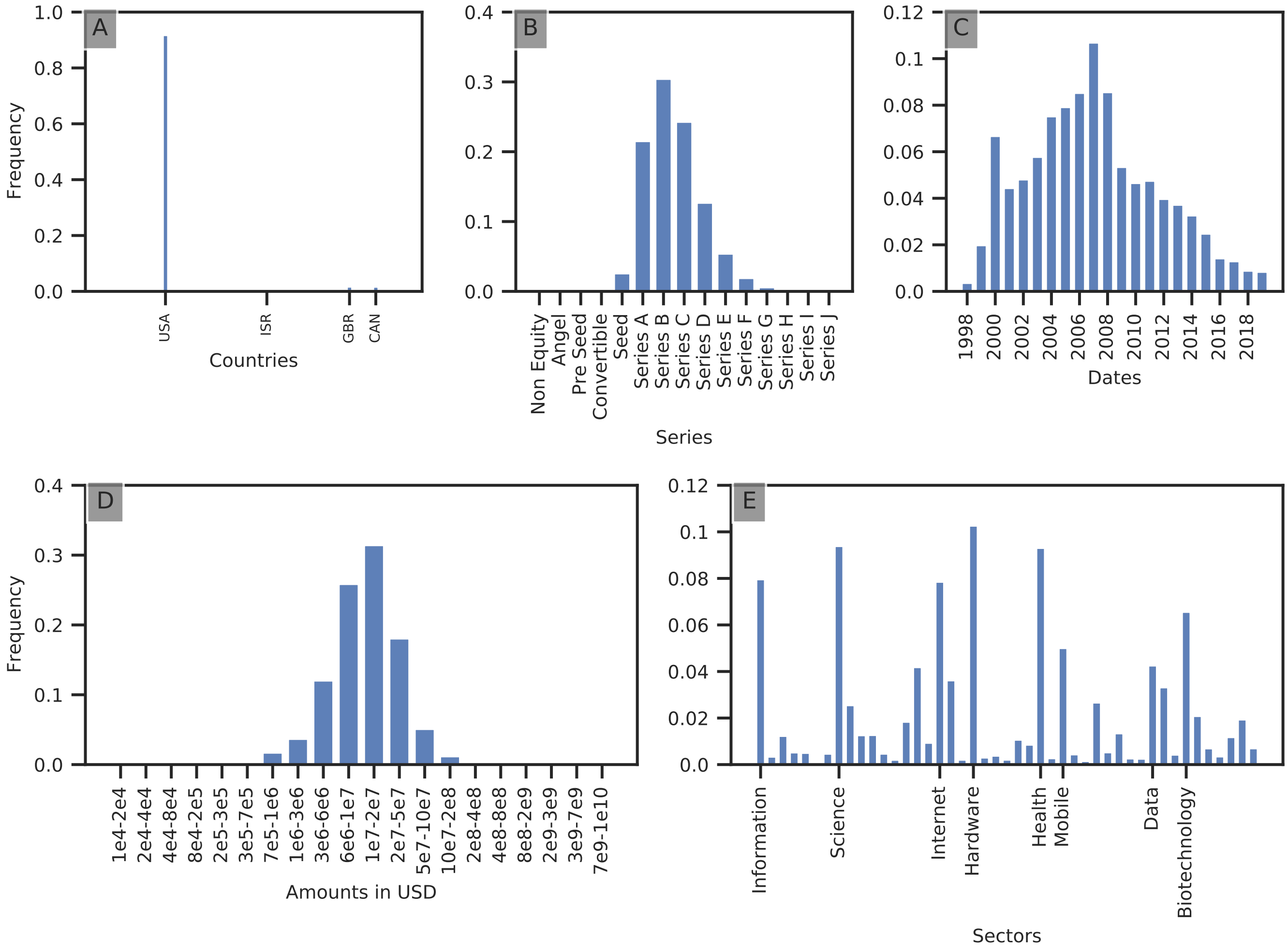

Fig S26. Representative investor of community C1.

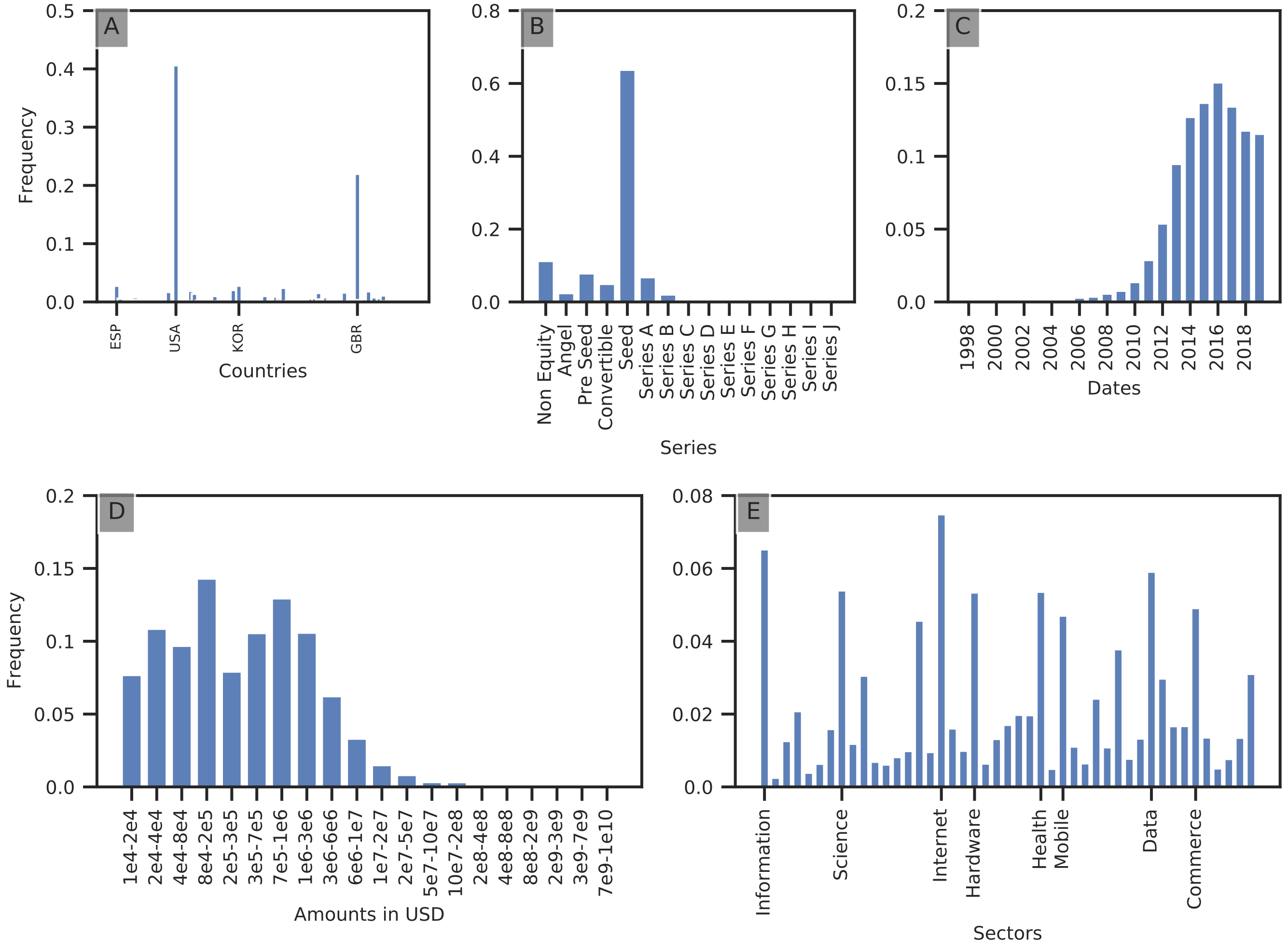

Fig S27. Representative investor of community C2.

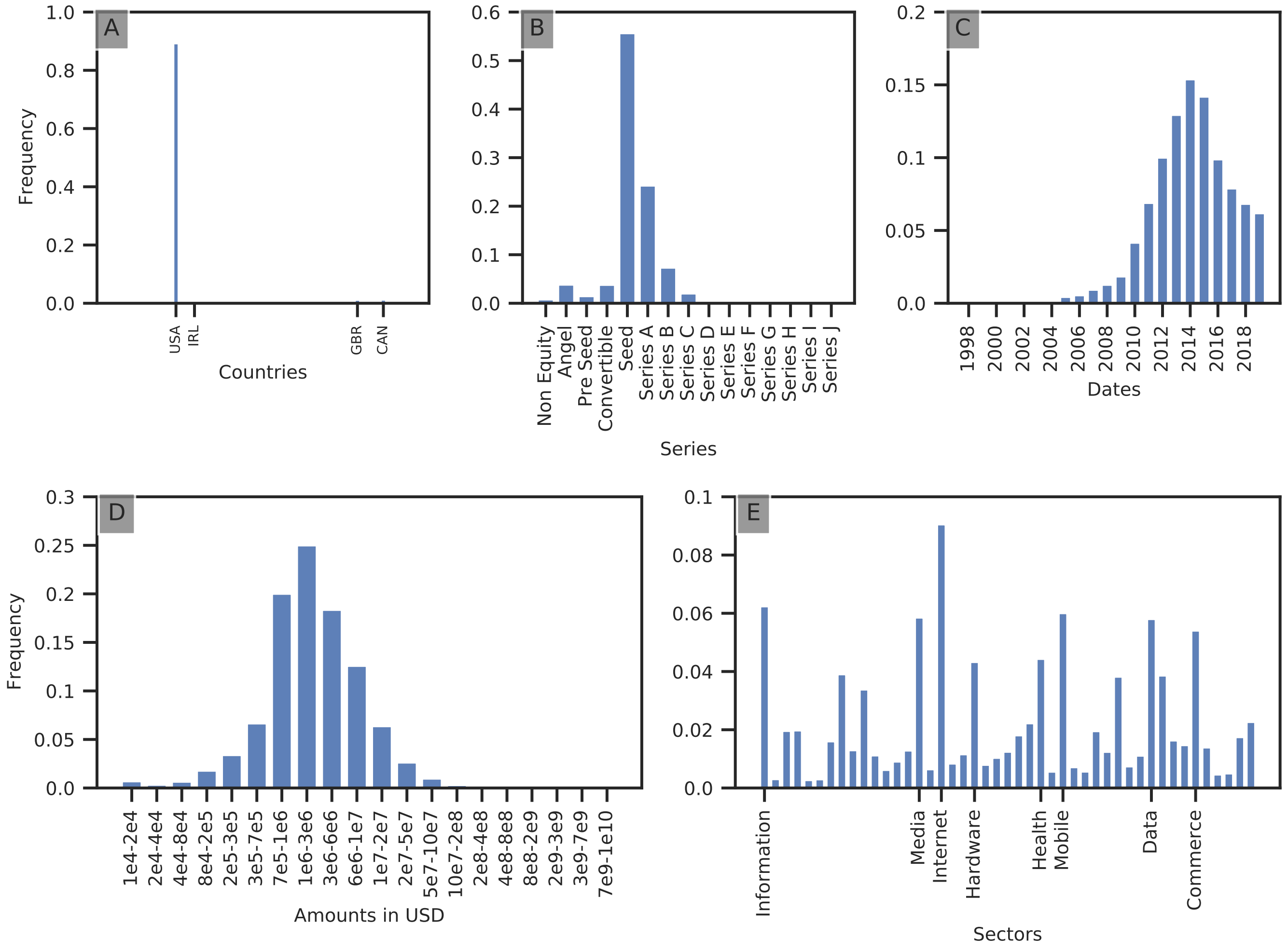

Fig S28. Representative investor of community C3.

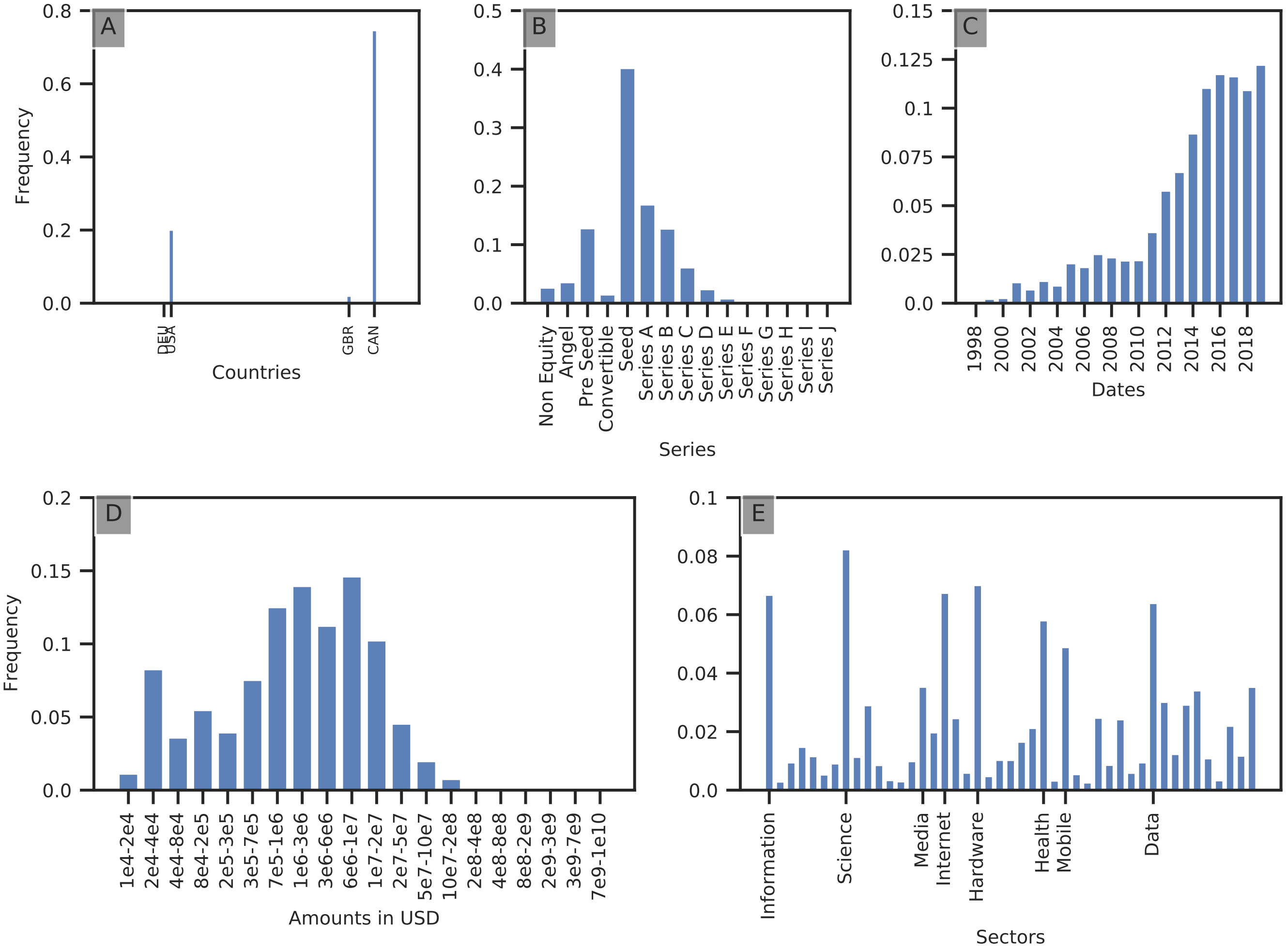

Fig S29. Representative investor of community C4.

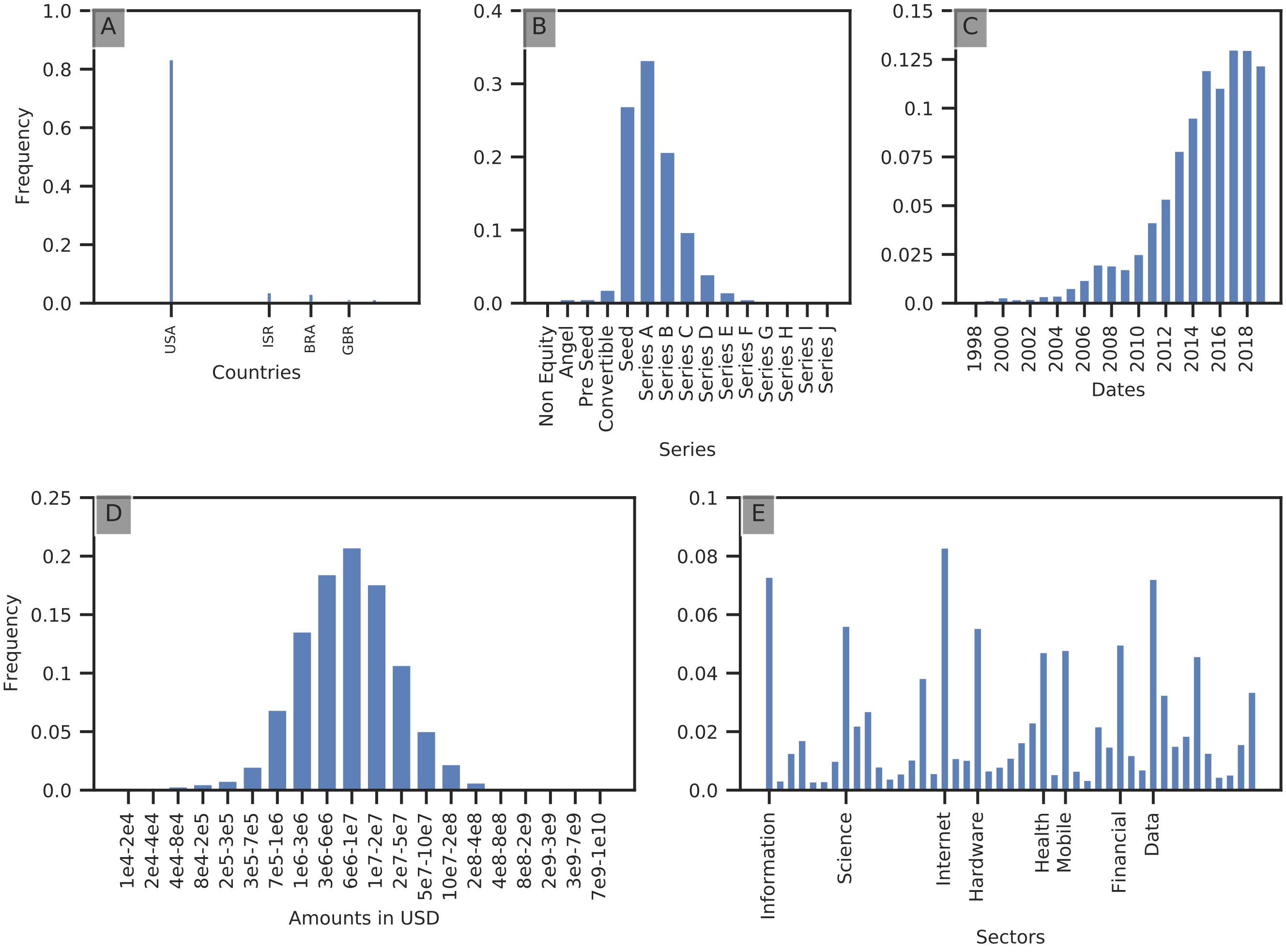

Fig S30. Representative investor of community C5.

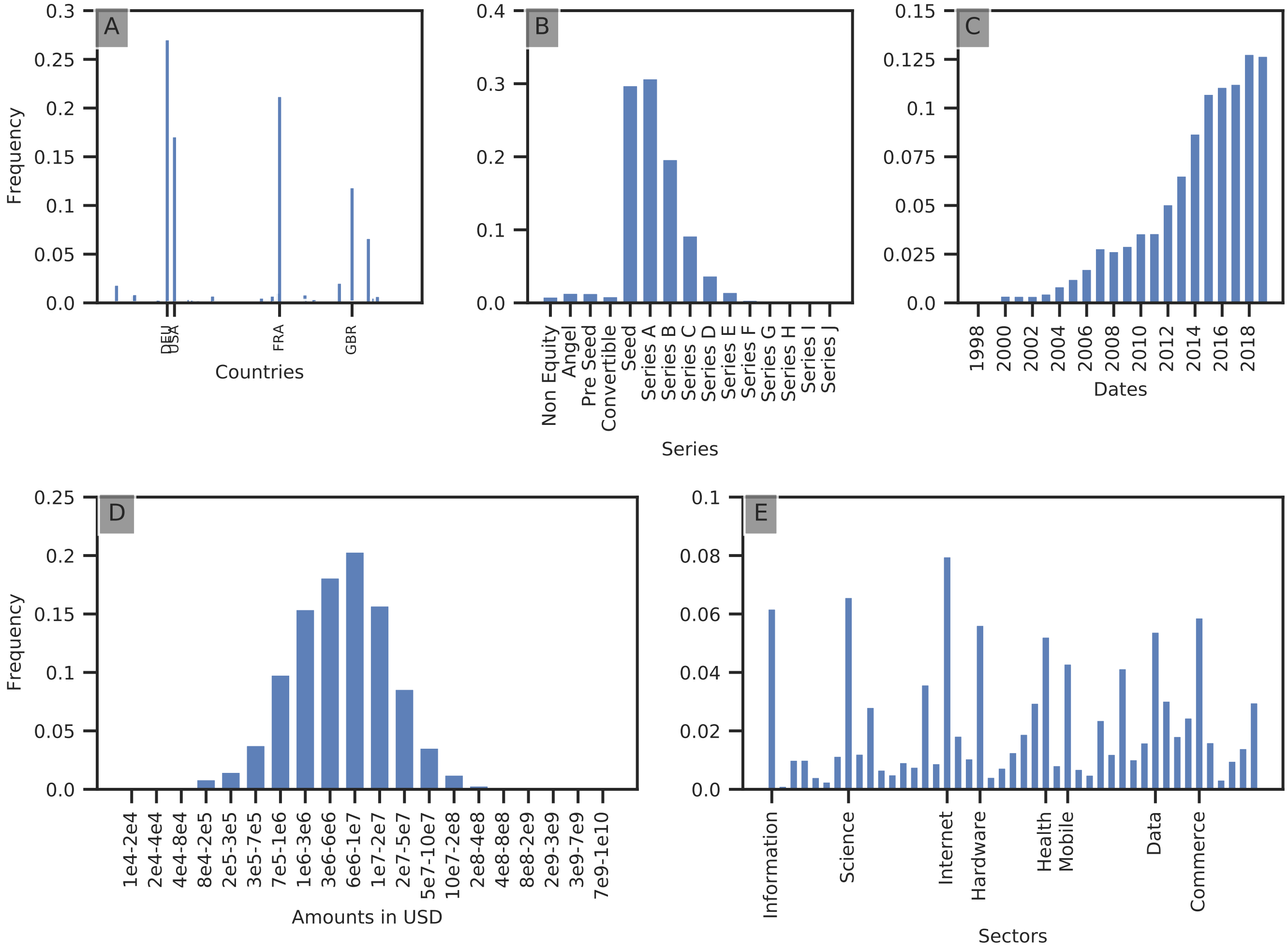

Fig S31. Representative investor of community C6.

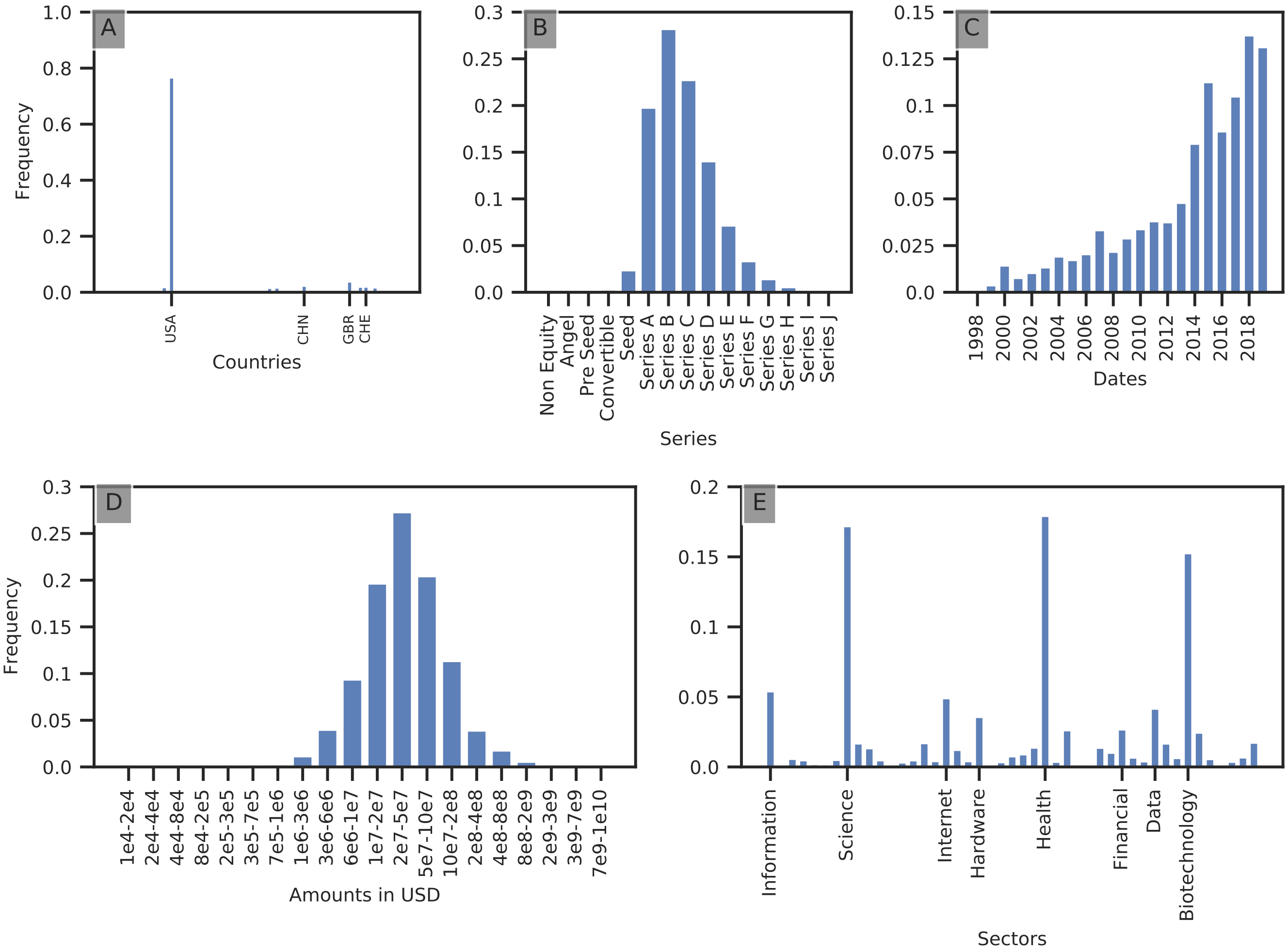

Fig S32. Representative investor of community C7.

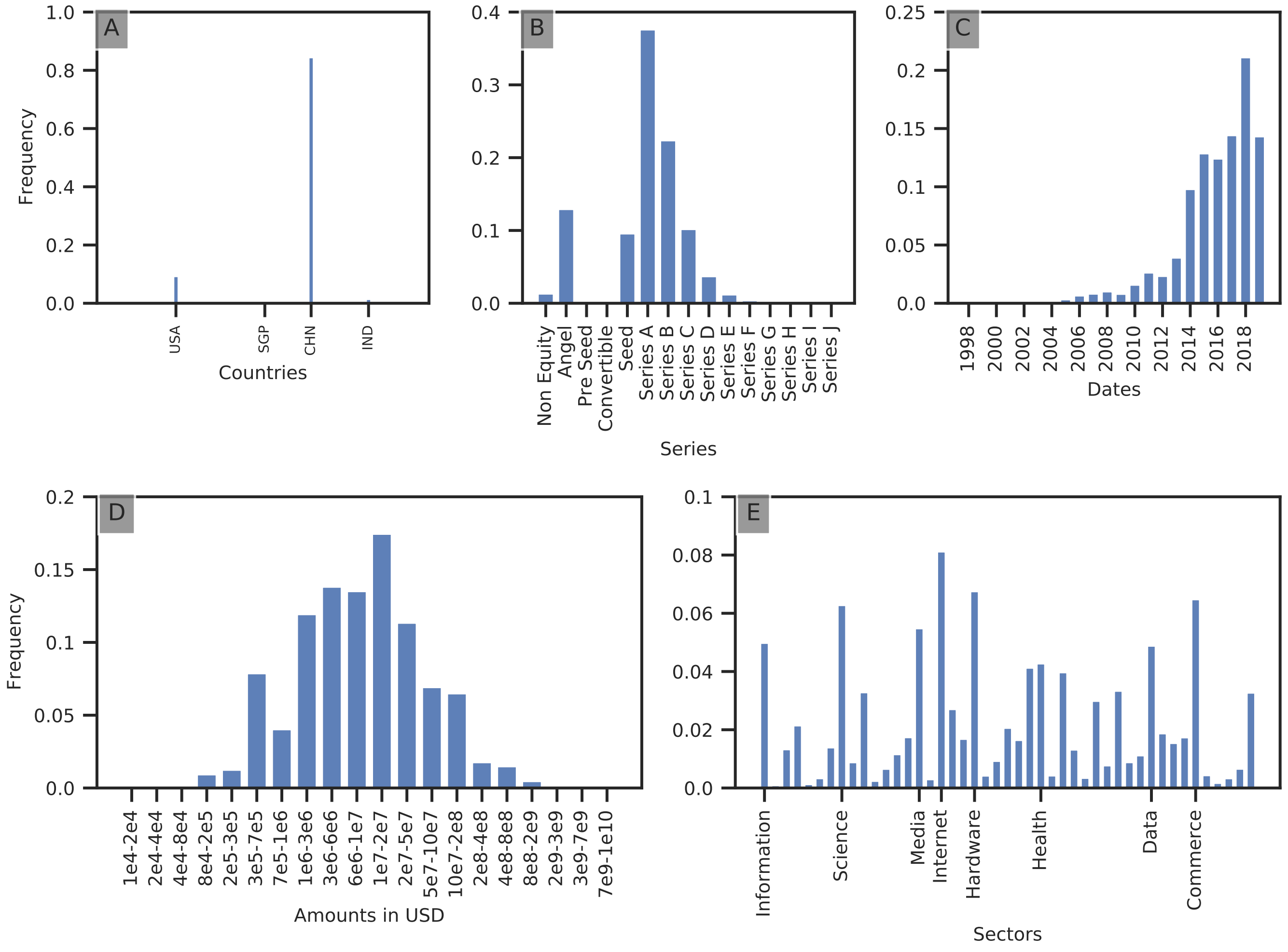

Fig S33. Representative investor of community C8.

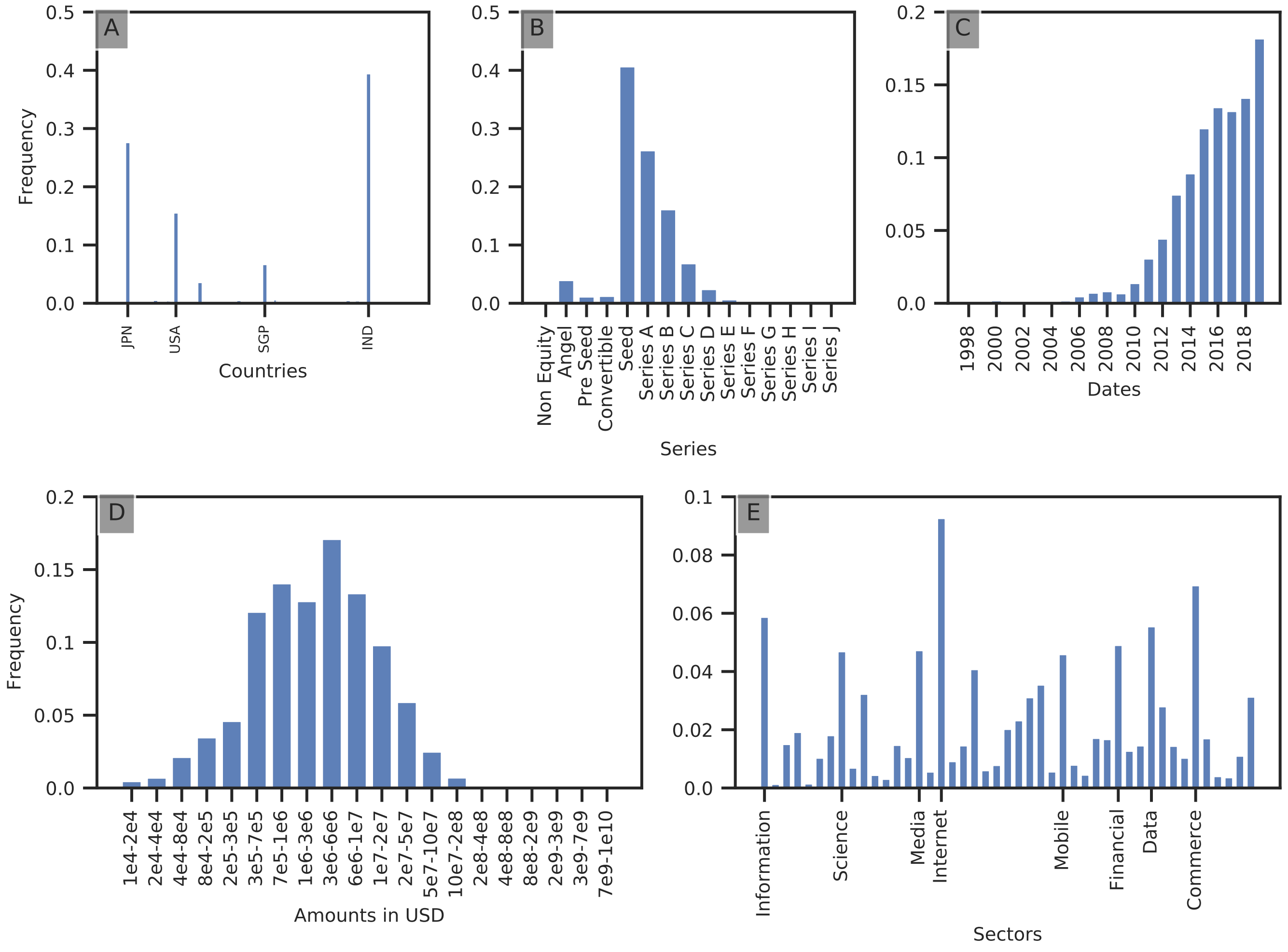

Fig S34. Representative investor of community C9.

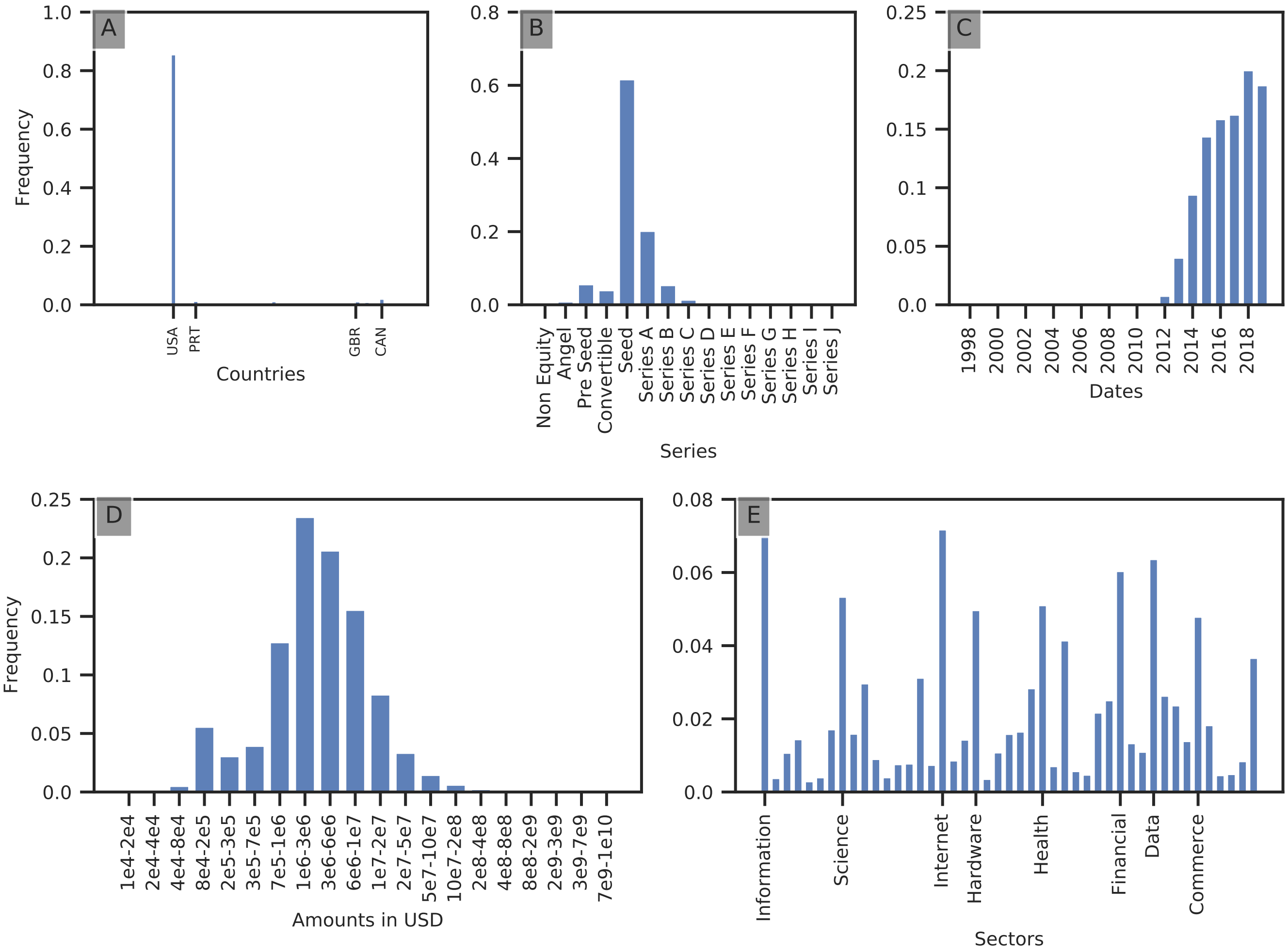

Fig S35. Representative investor of community C10.

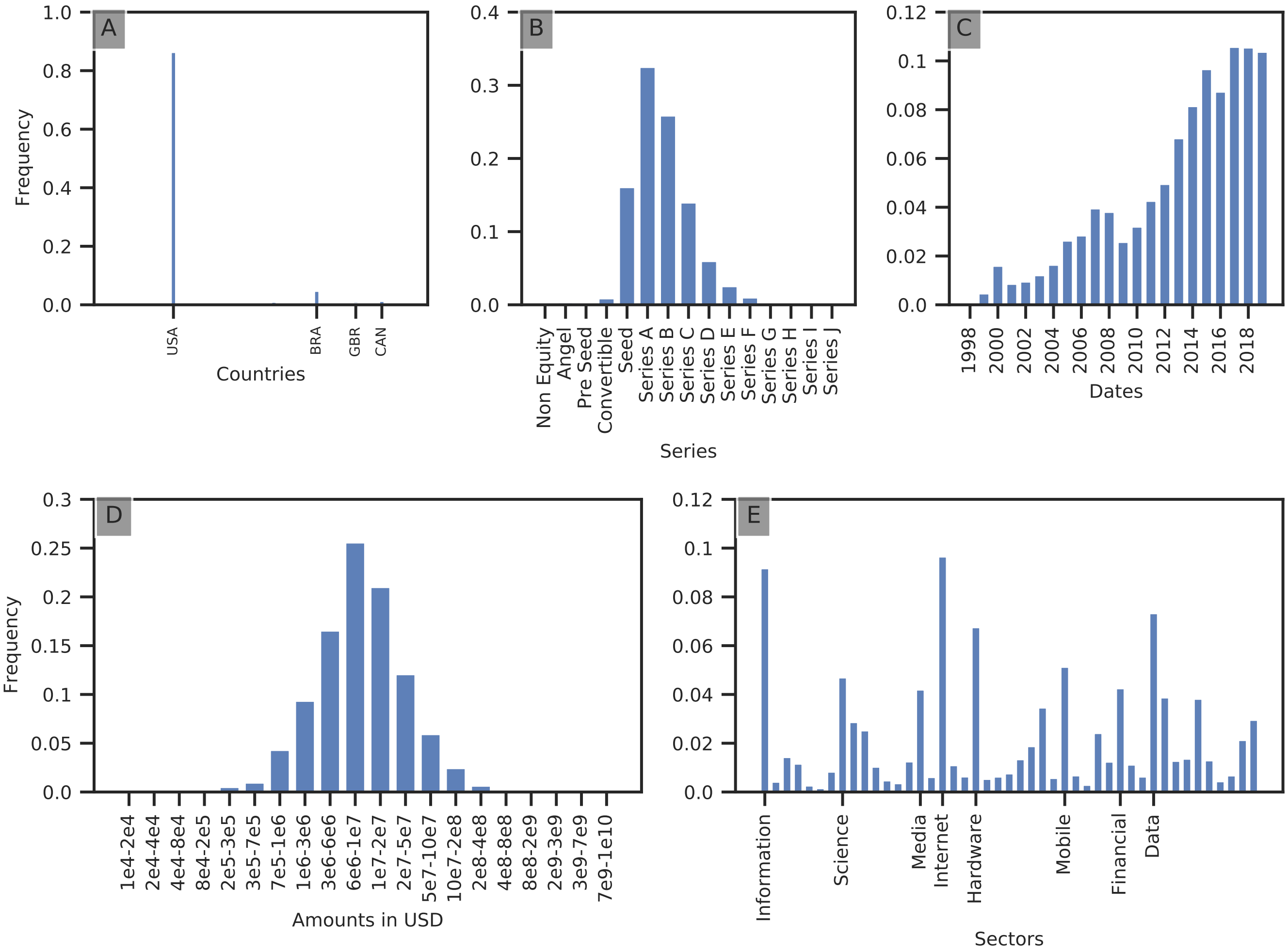

Fig S36. Representative investor of community D0.

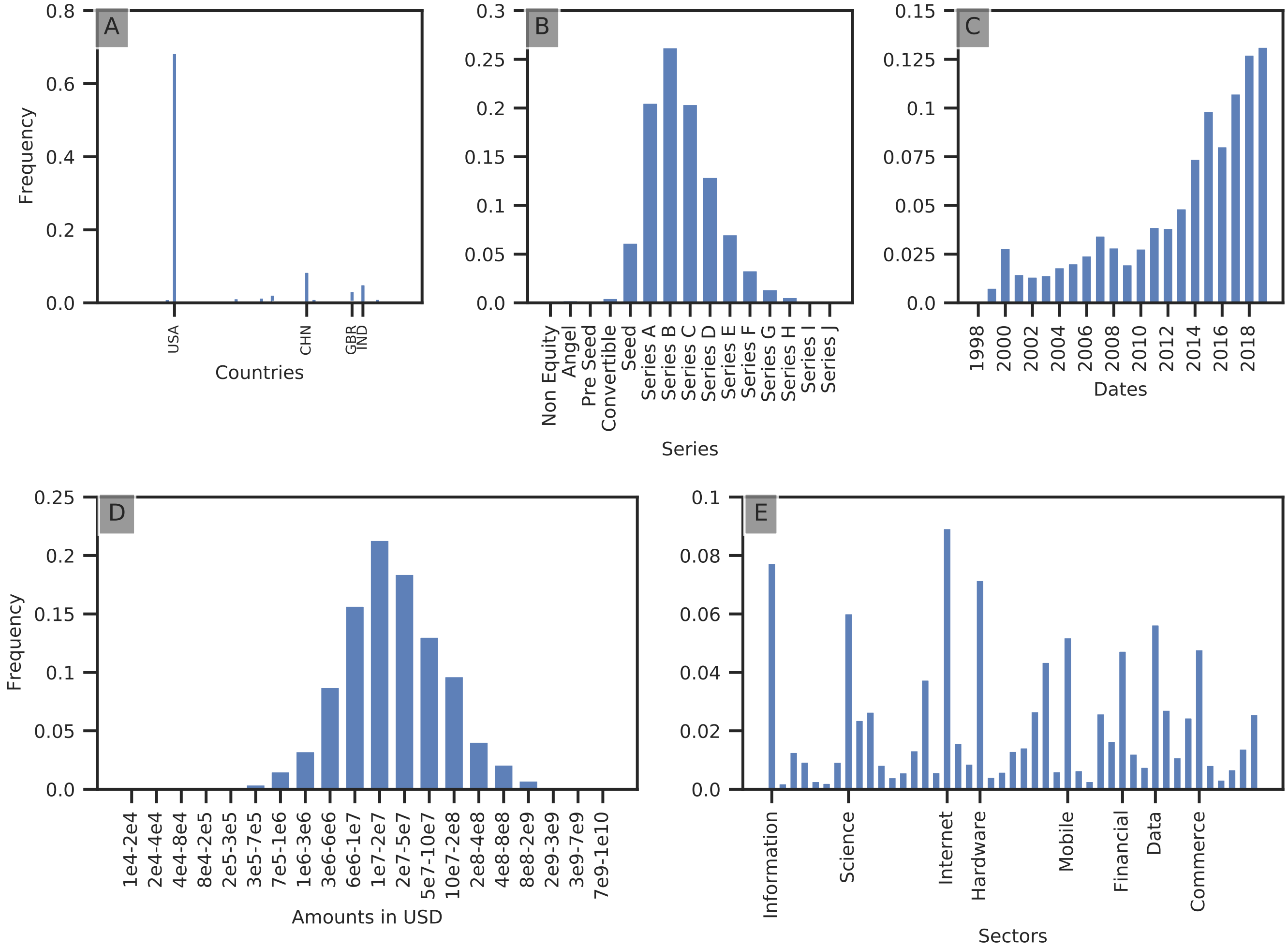

Fig S37. Representative investor of community D1.

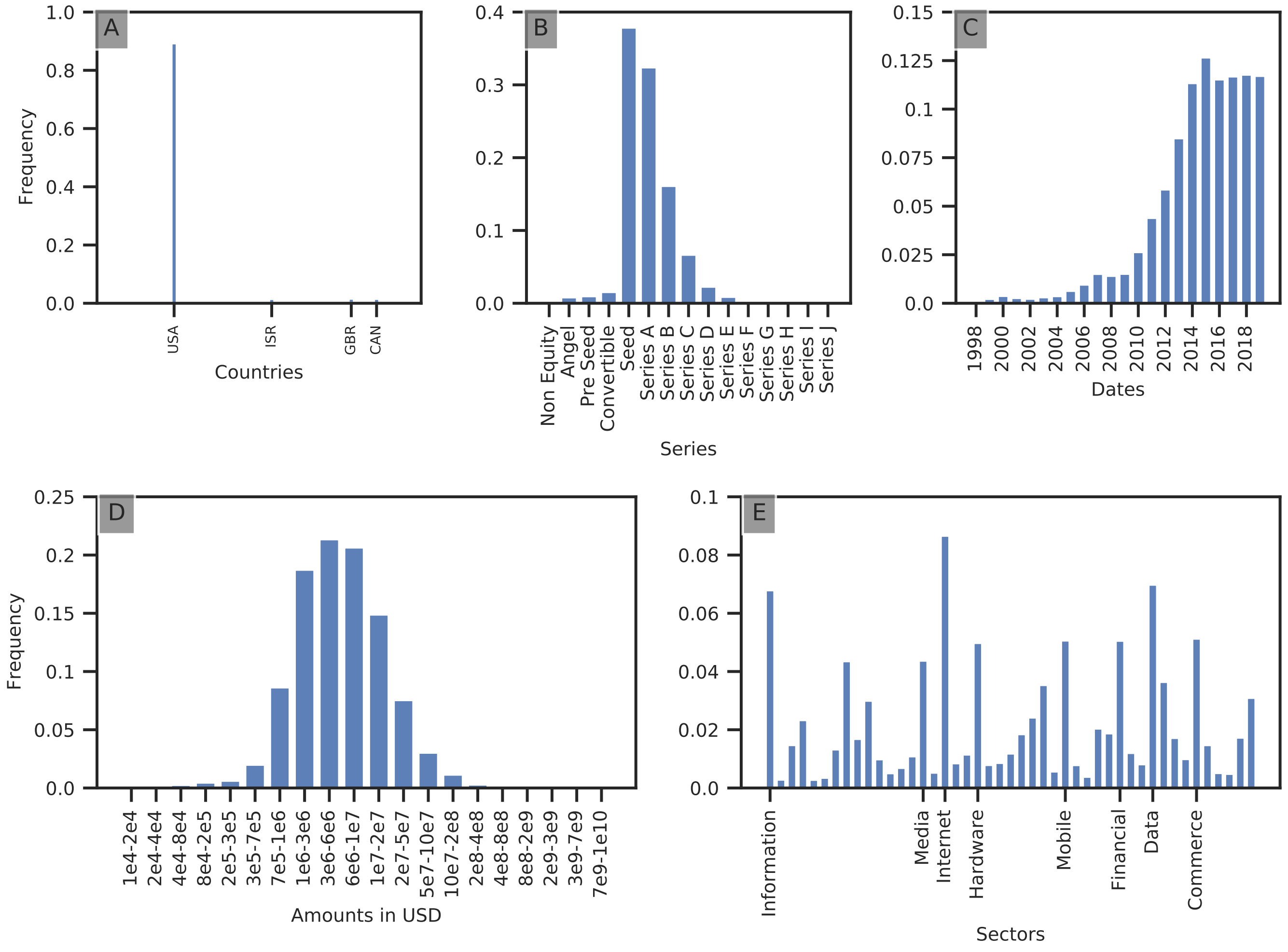

Fig S38. Representative investor of community D2.

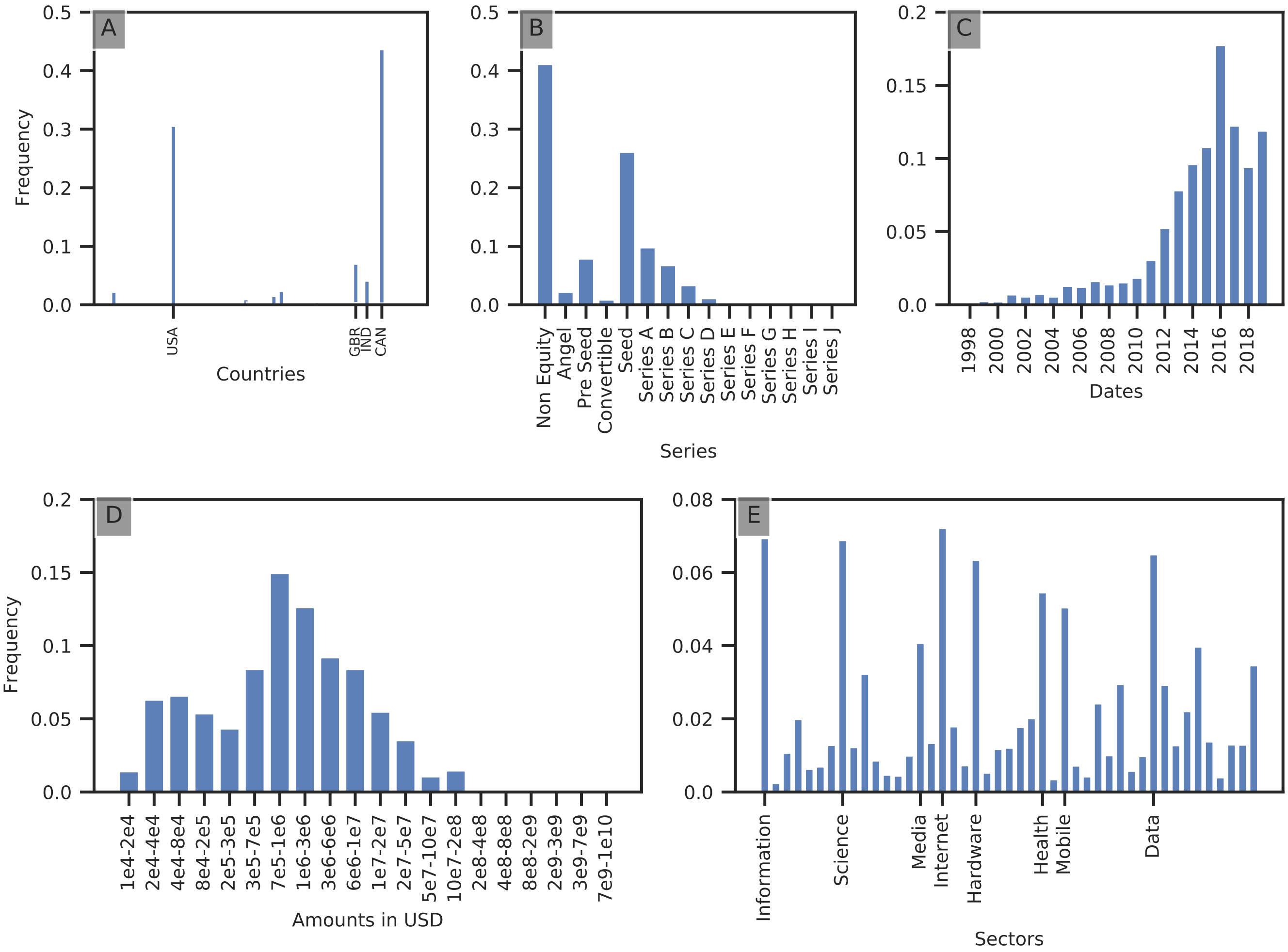

Fig S39. Representative investor of community D3.

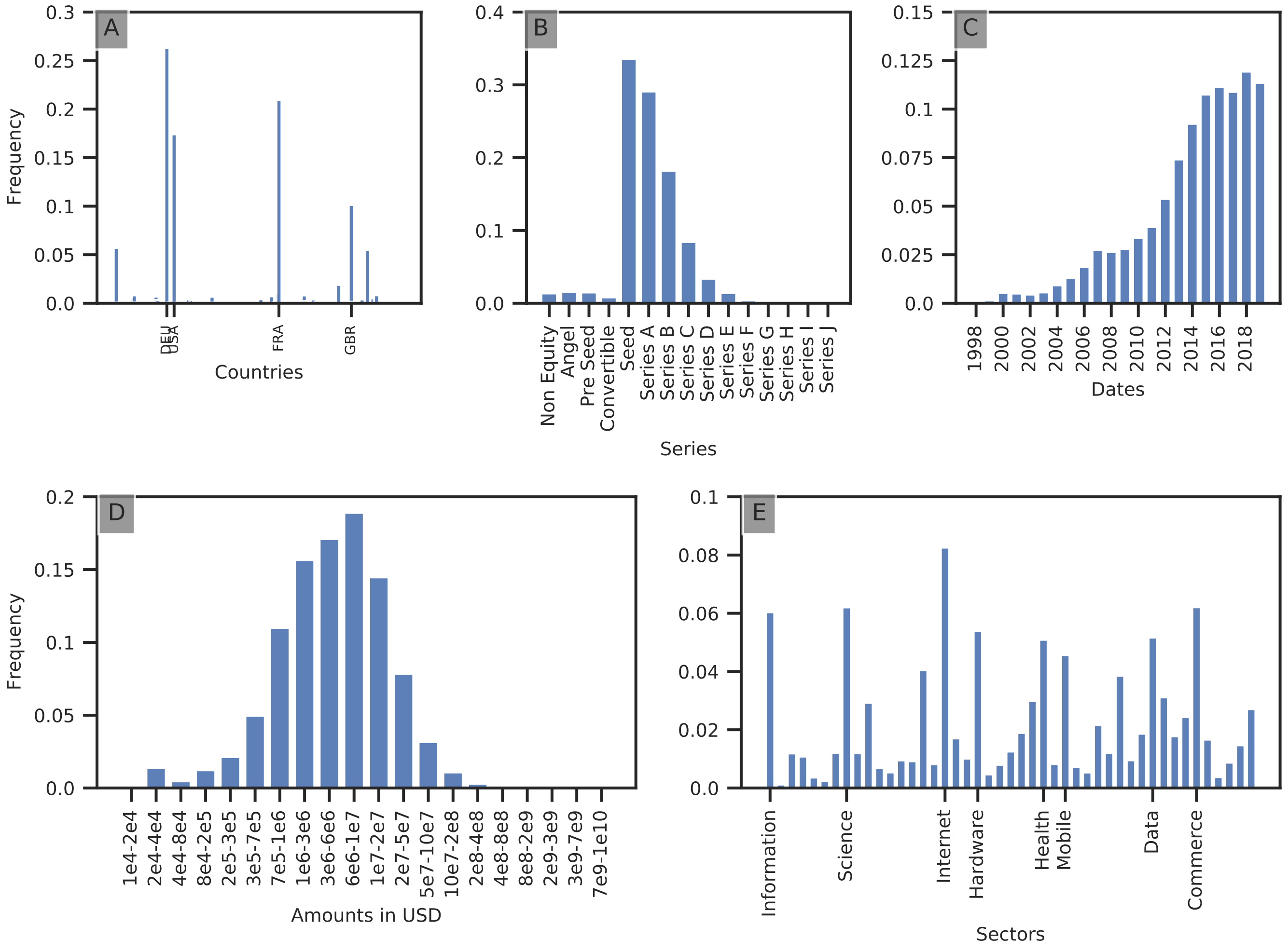

Fig S40. Representative investor of community D4.

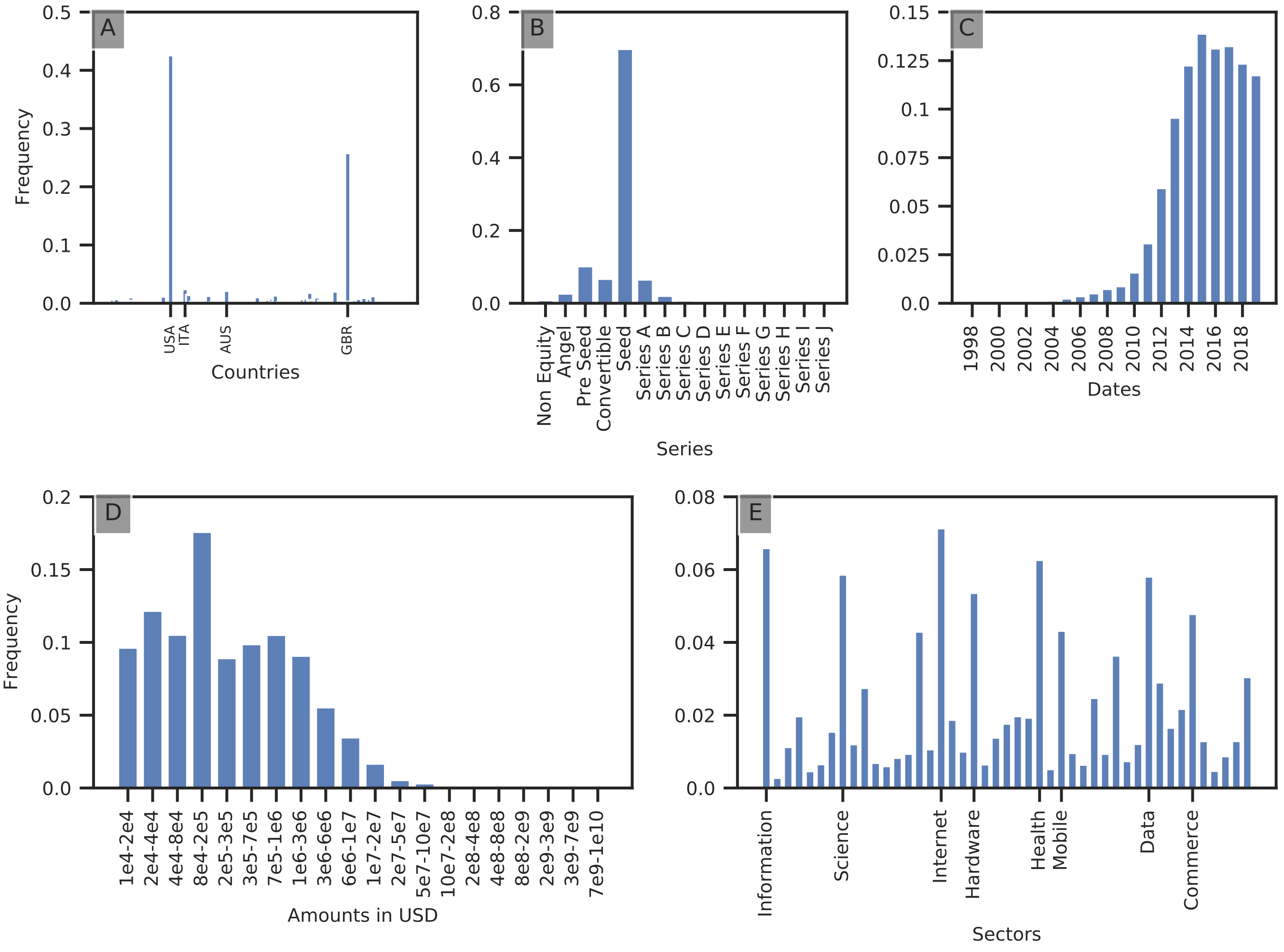

Fig S41. Representative investor of community D5.

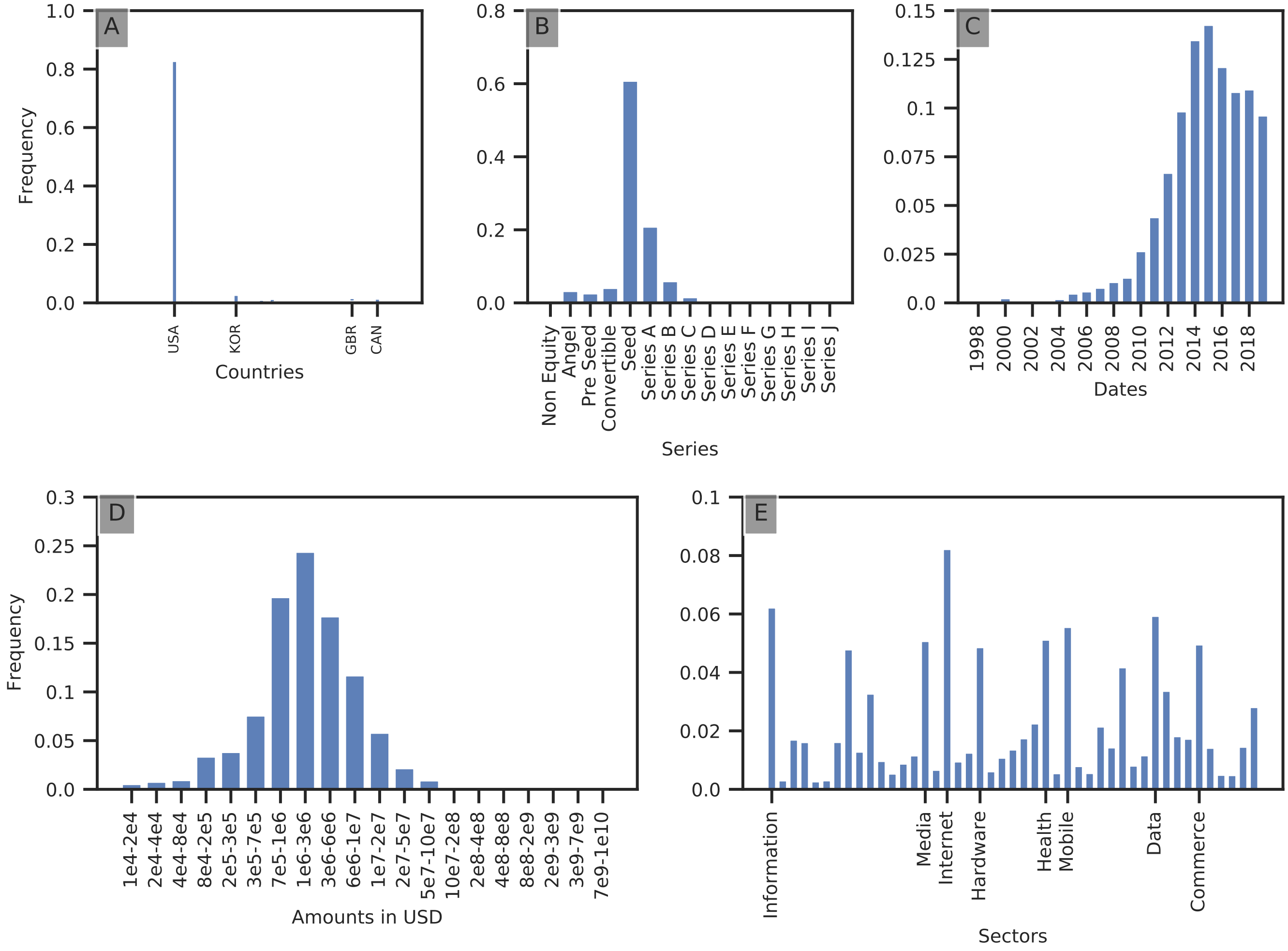

Fig S42. Representative investor of community D6.

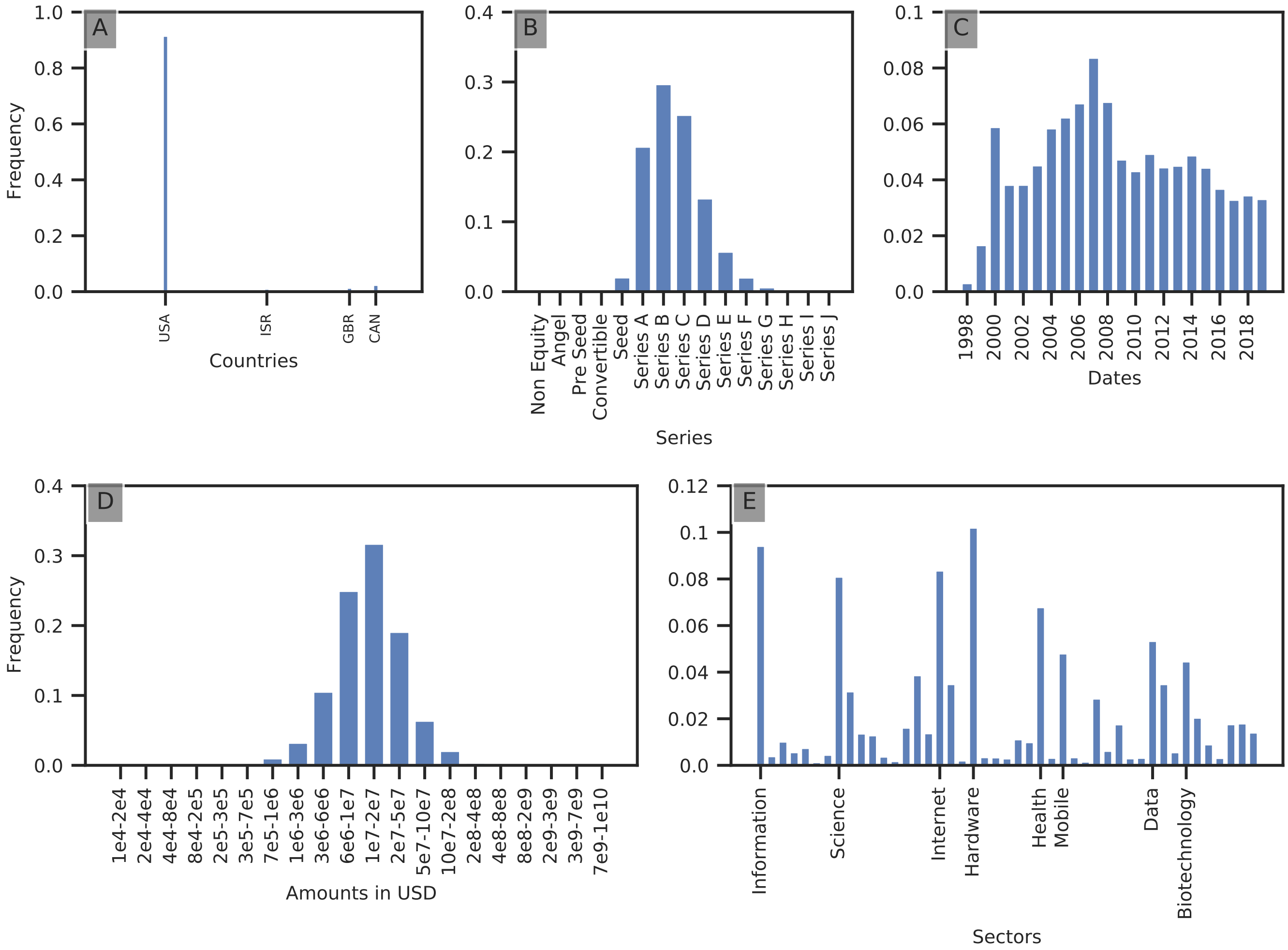

Fig S43. Representative investor of community D7.

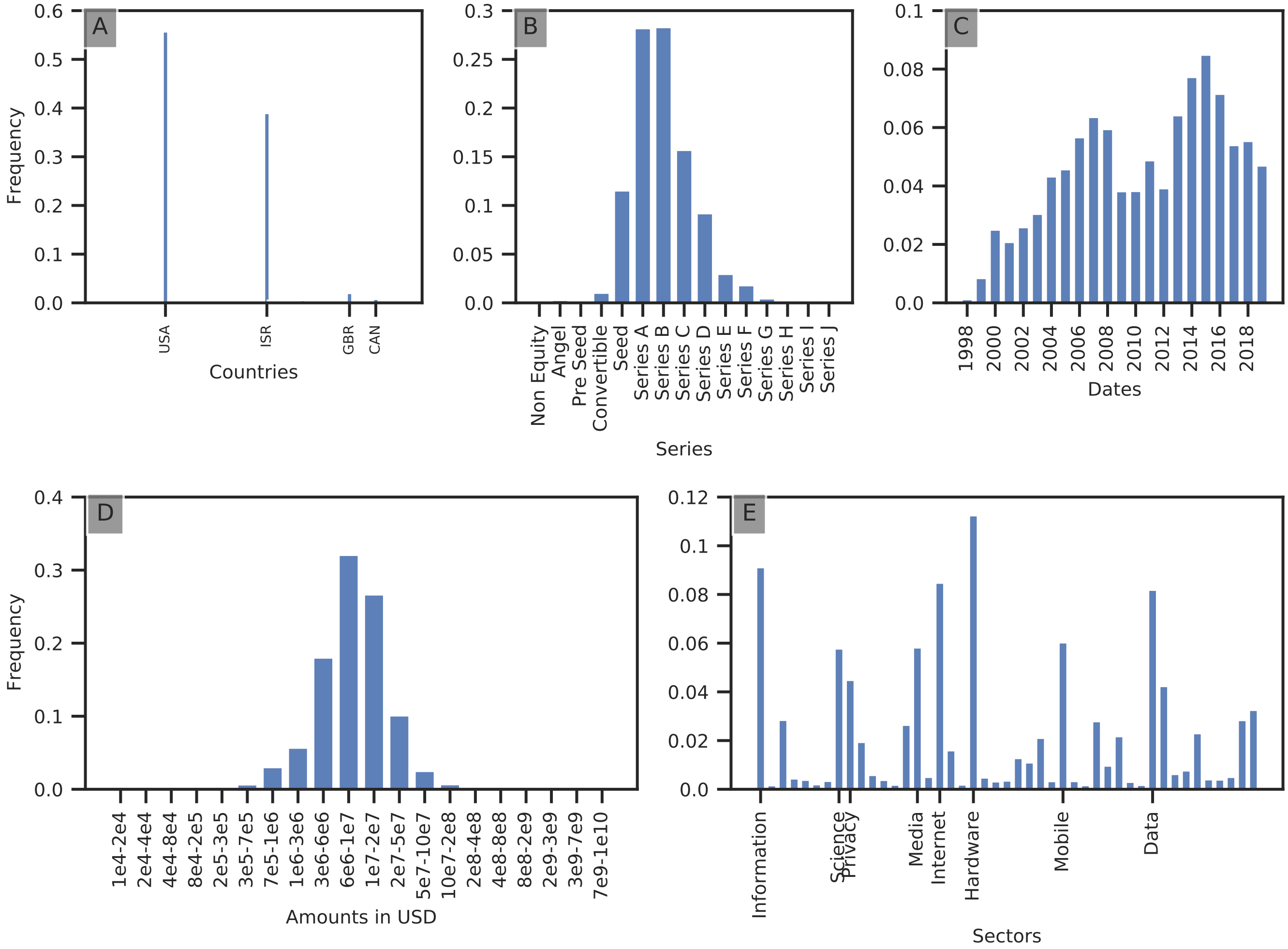

Fig S44. Representative investor of community D8.

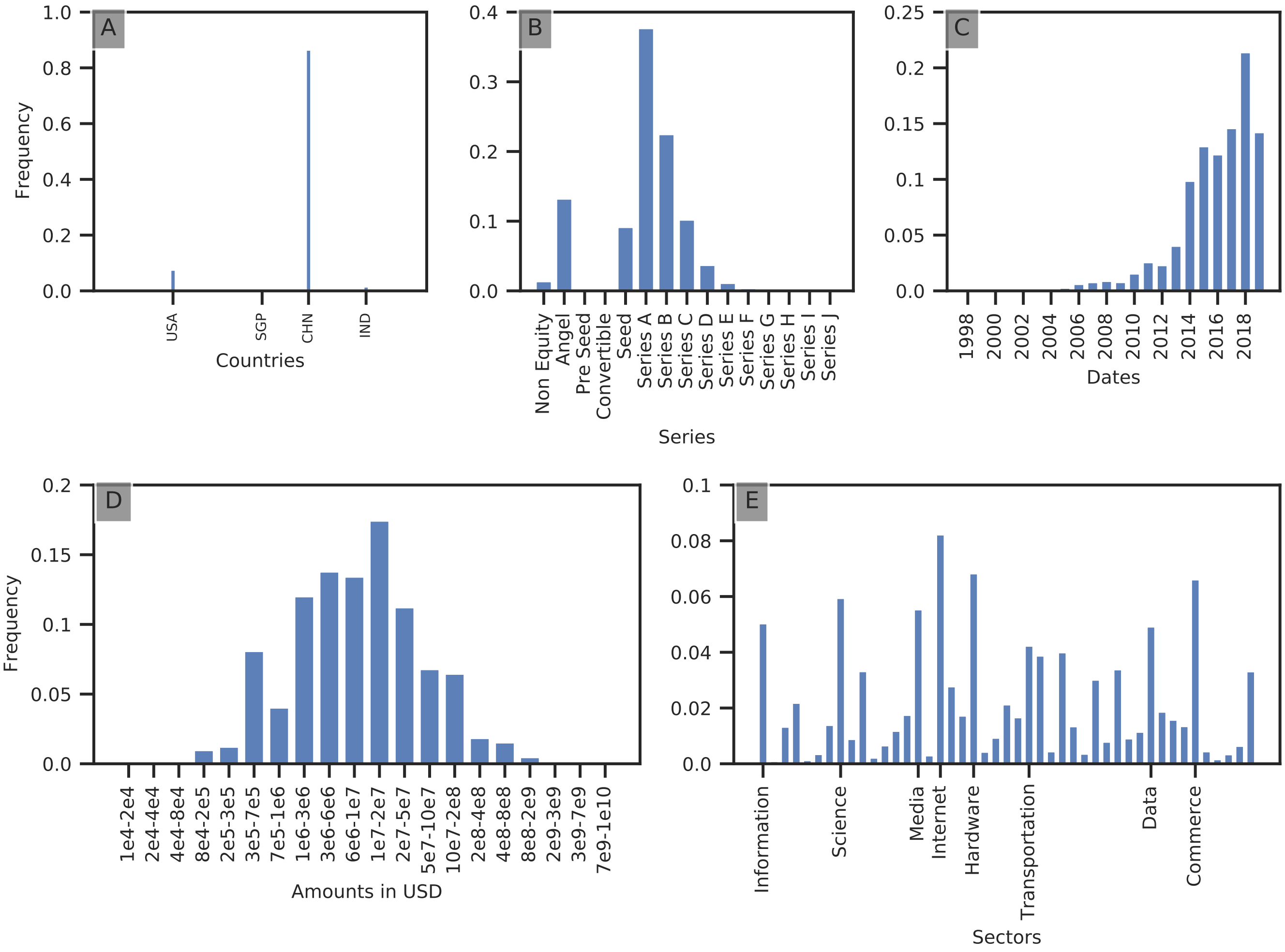

Fig S45. Representative investor of community D9.

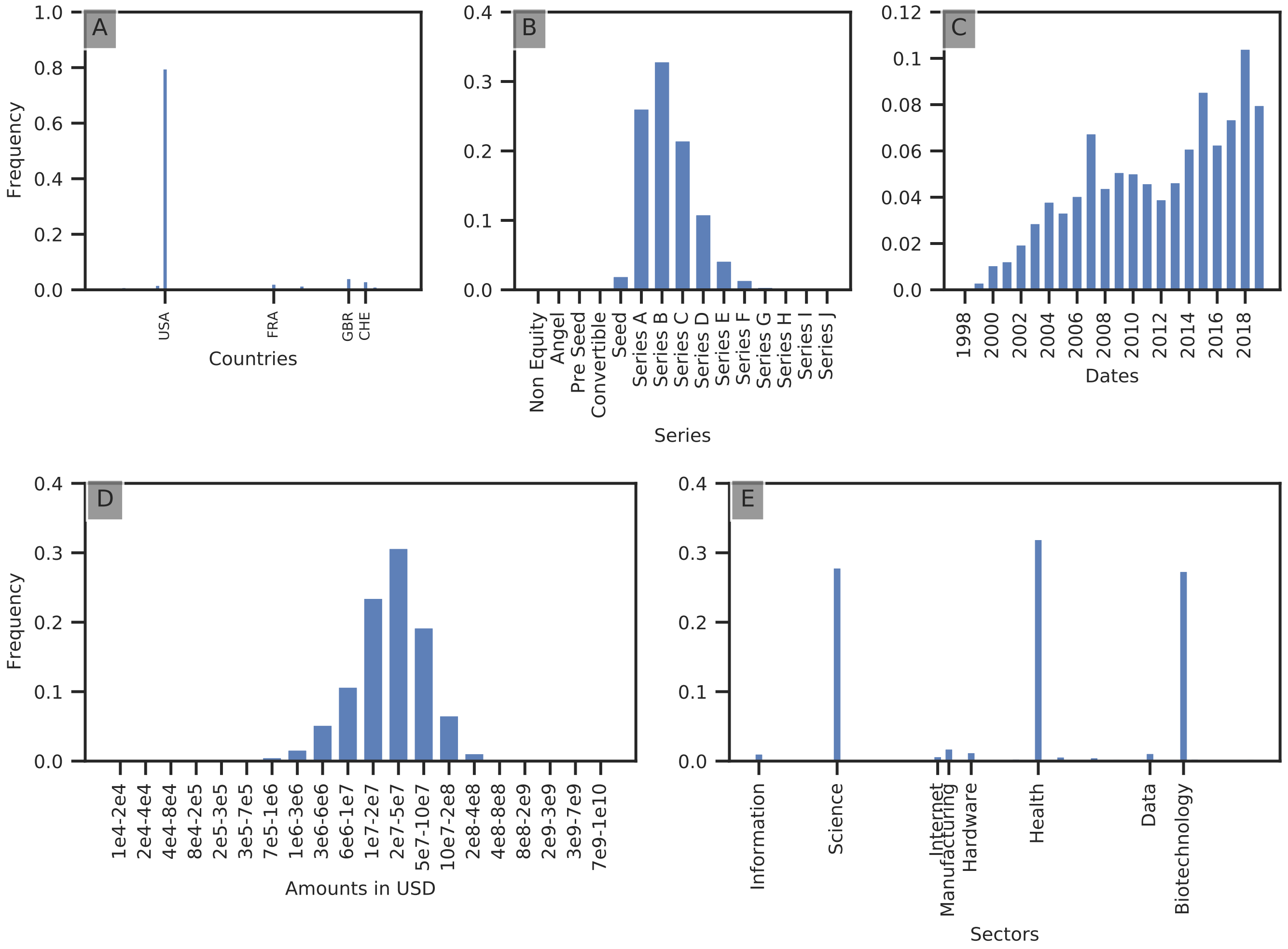

Fig S46. Representative investor of community D10.

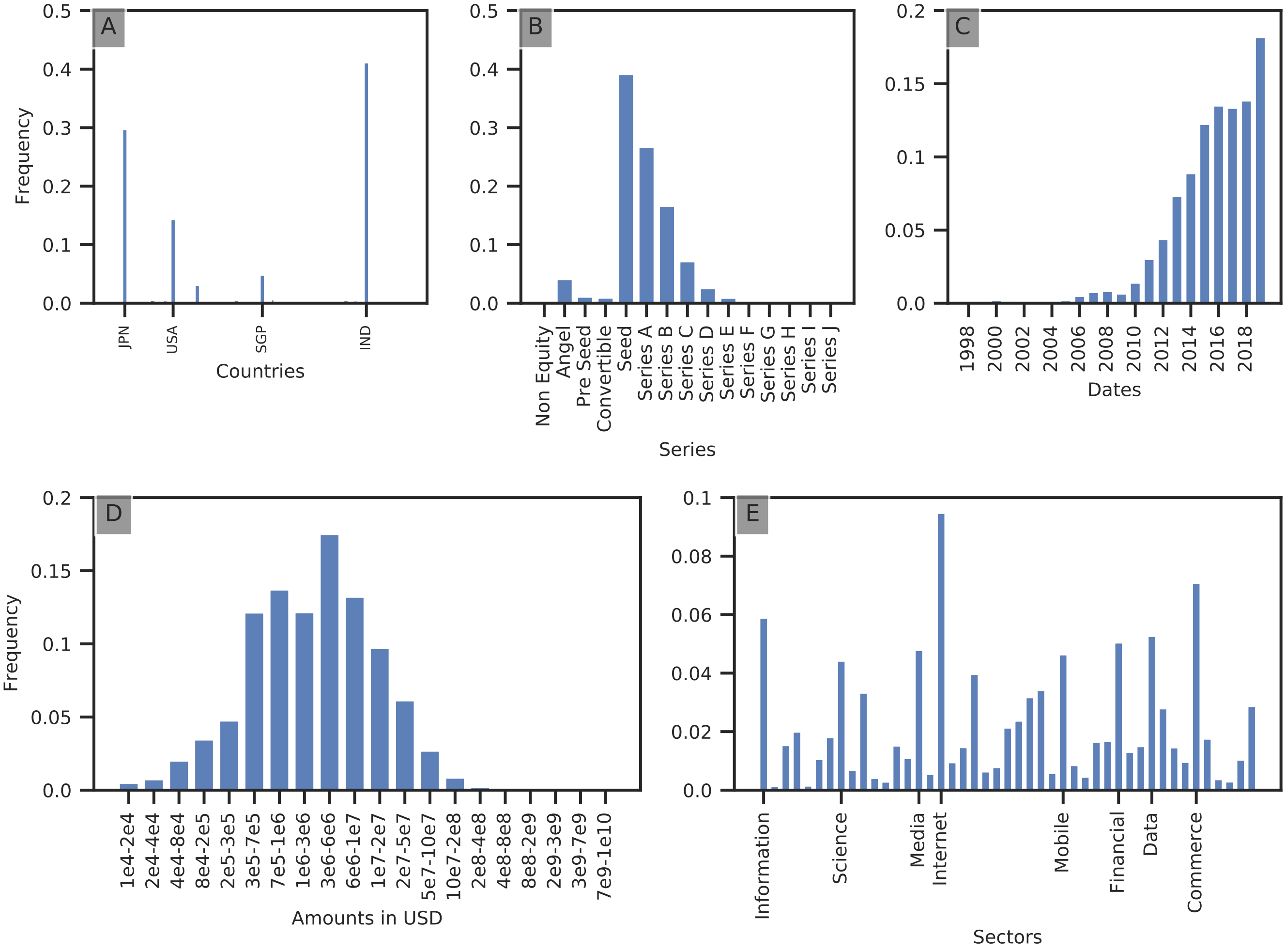

Fig S47. Representative investor of community D11.

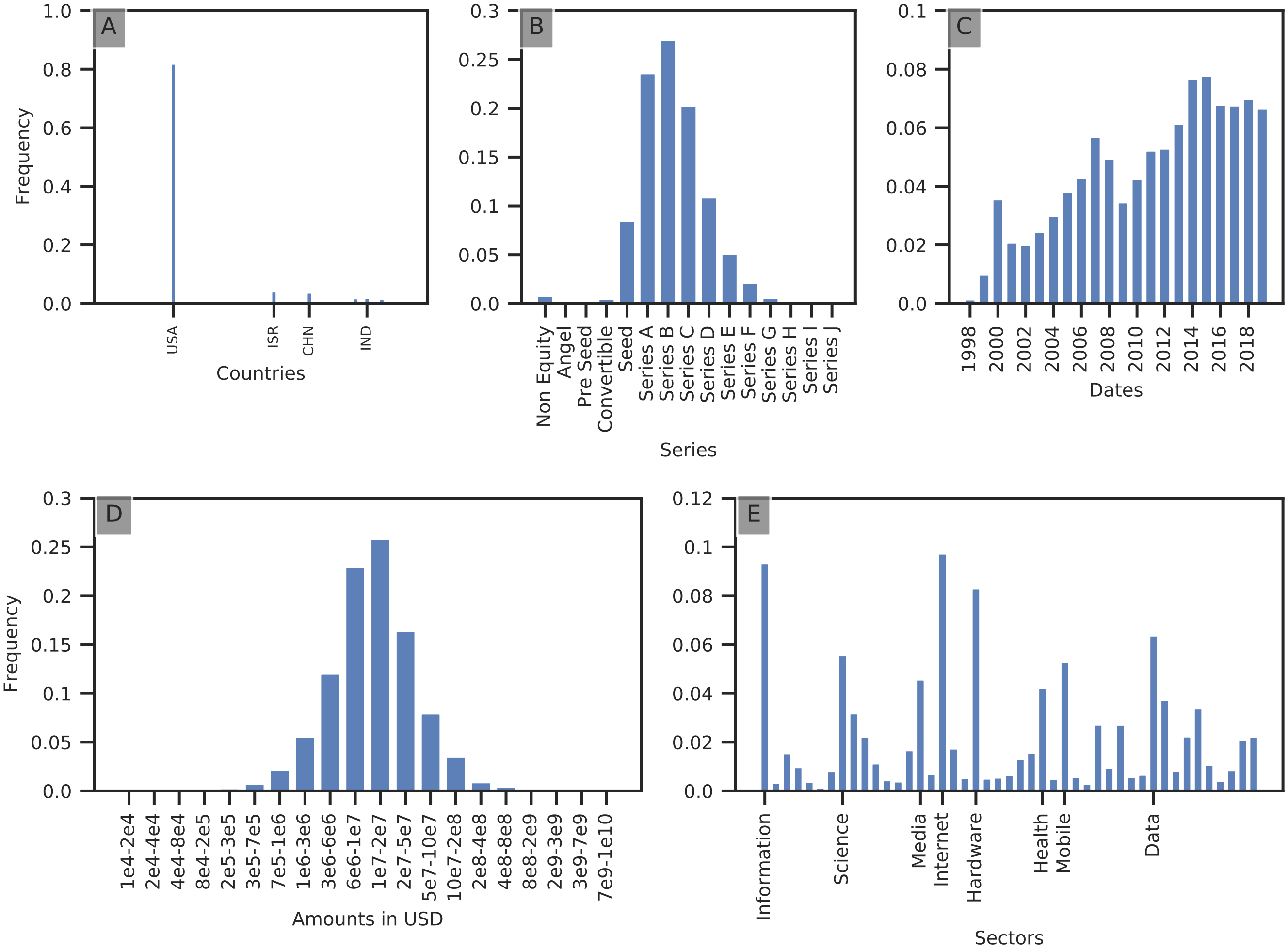

Fig S48. Representative investor of community E0.

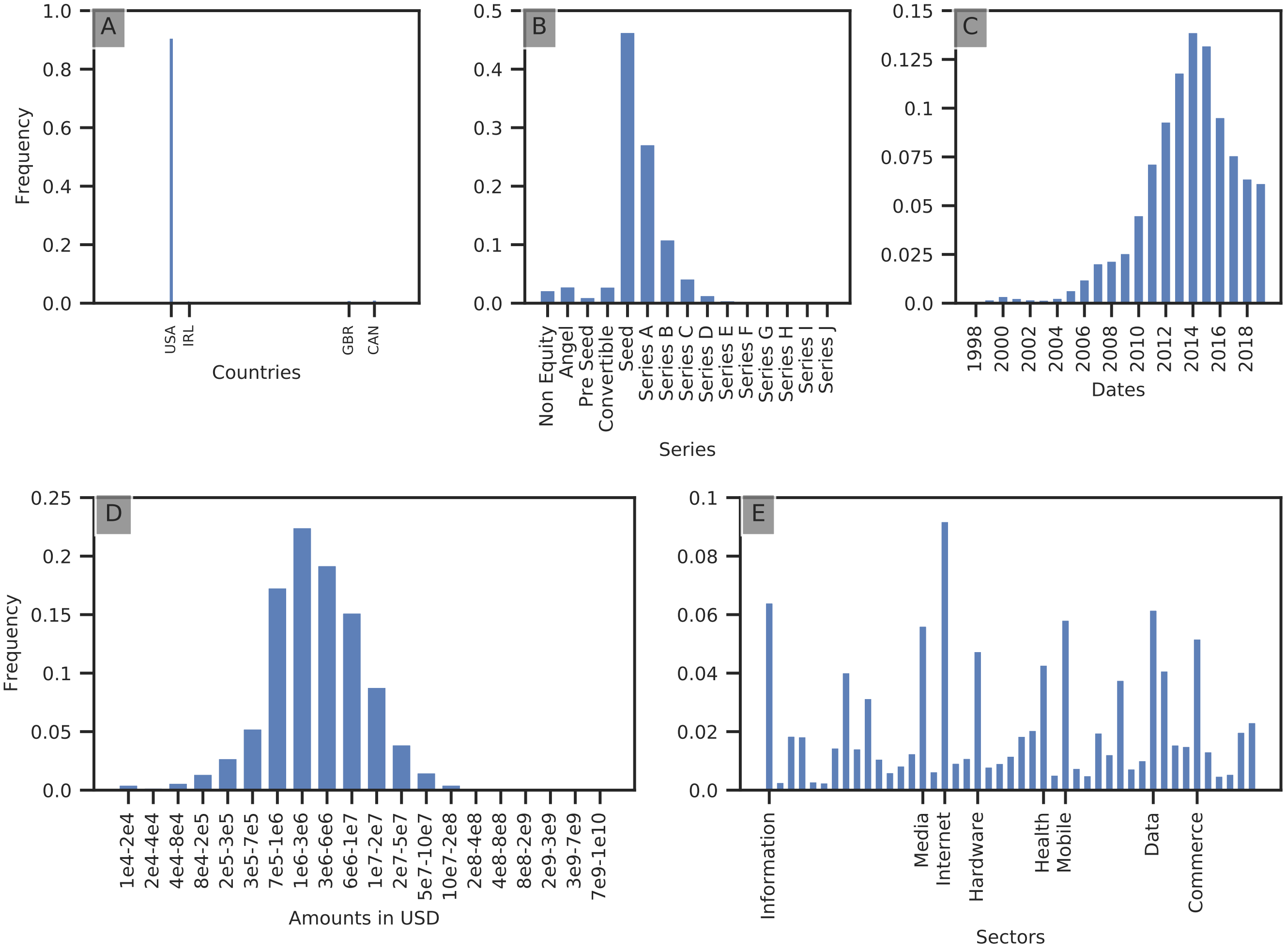

Fig S49. Representative investor of community E1.

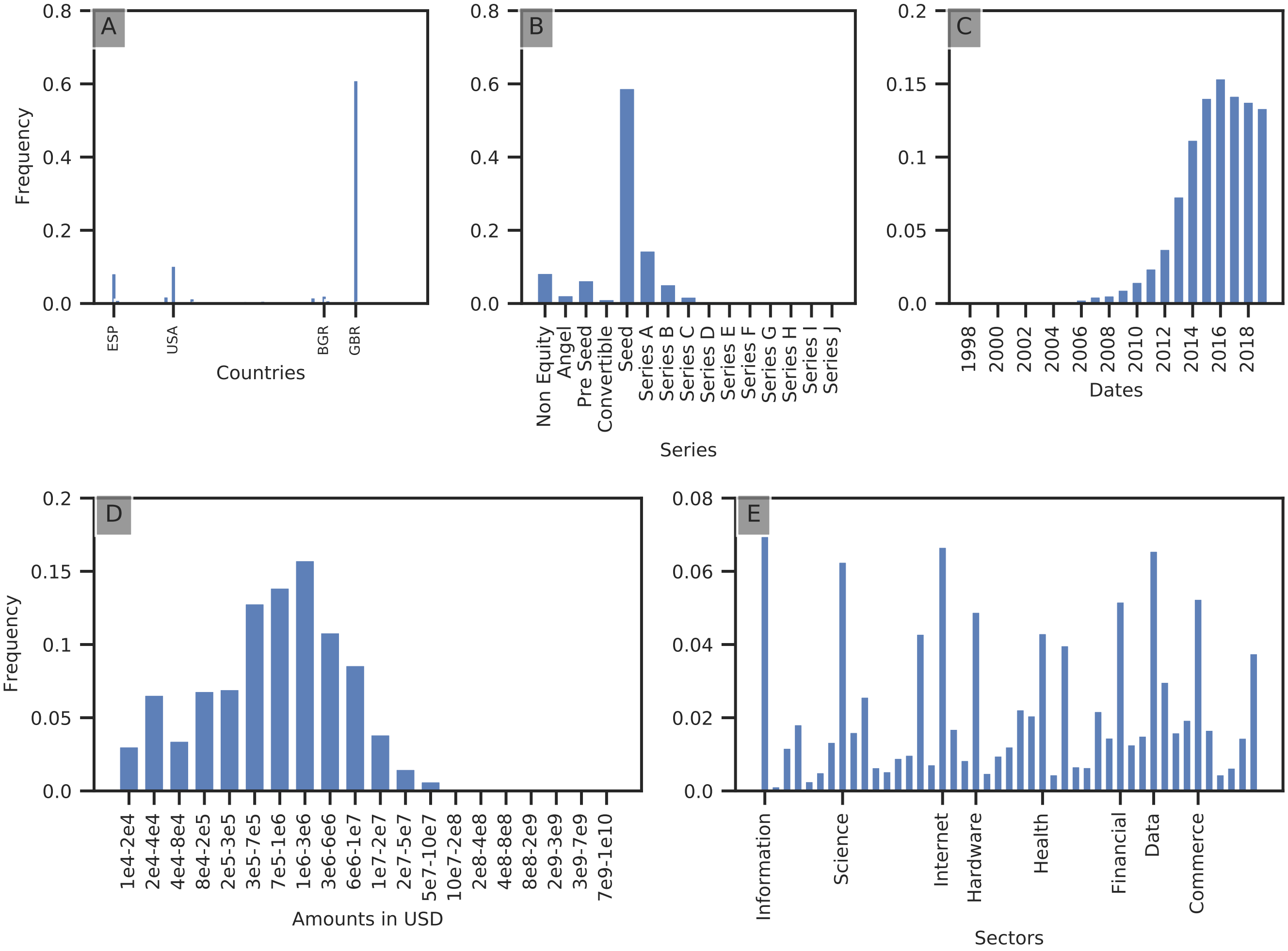

Fig S50. Representative investor of community E2.

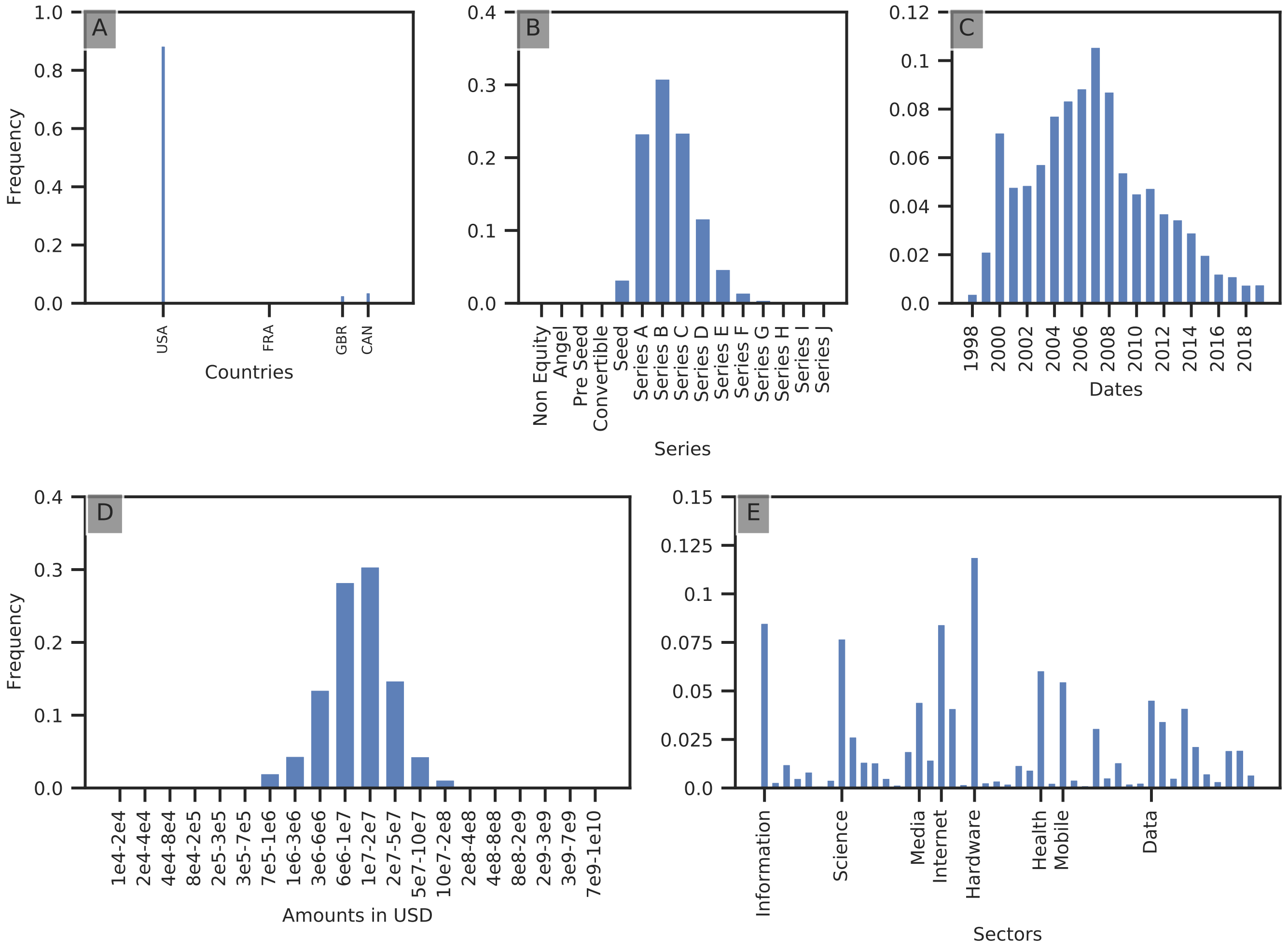

Fig S51. Representative investor of community E3.

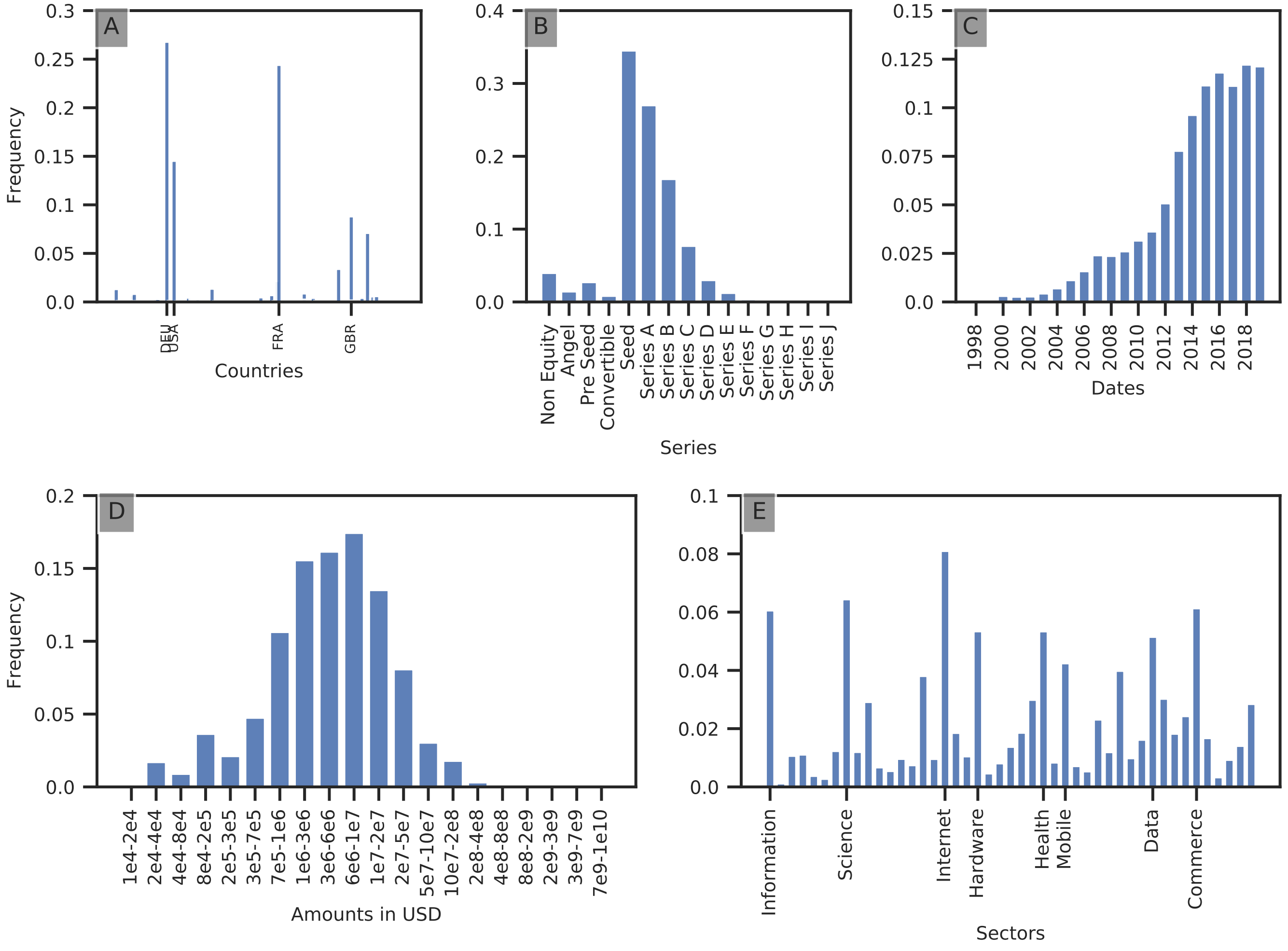

Fig S52. Representative investor of community E4.

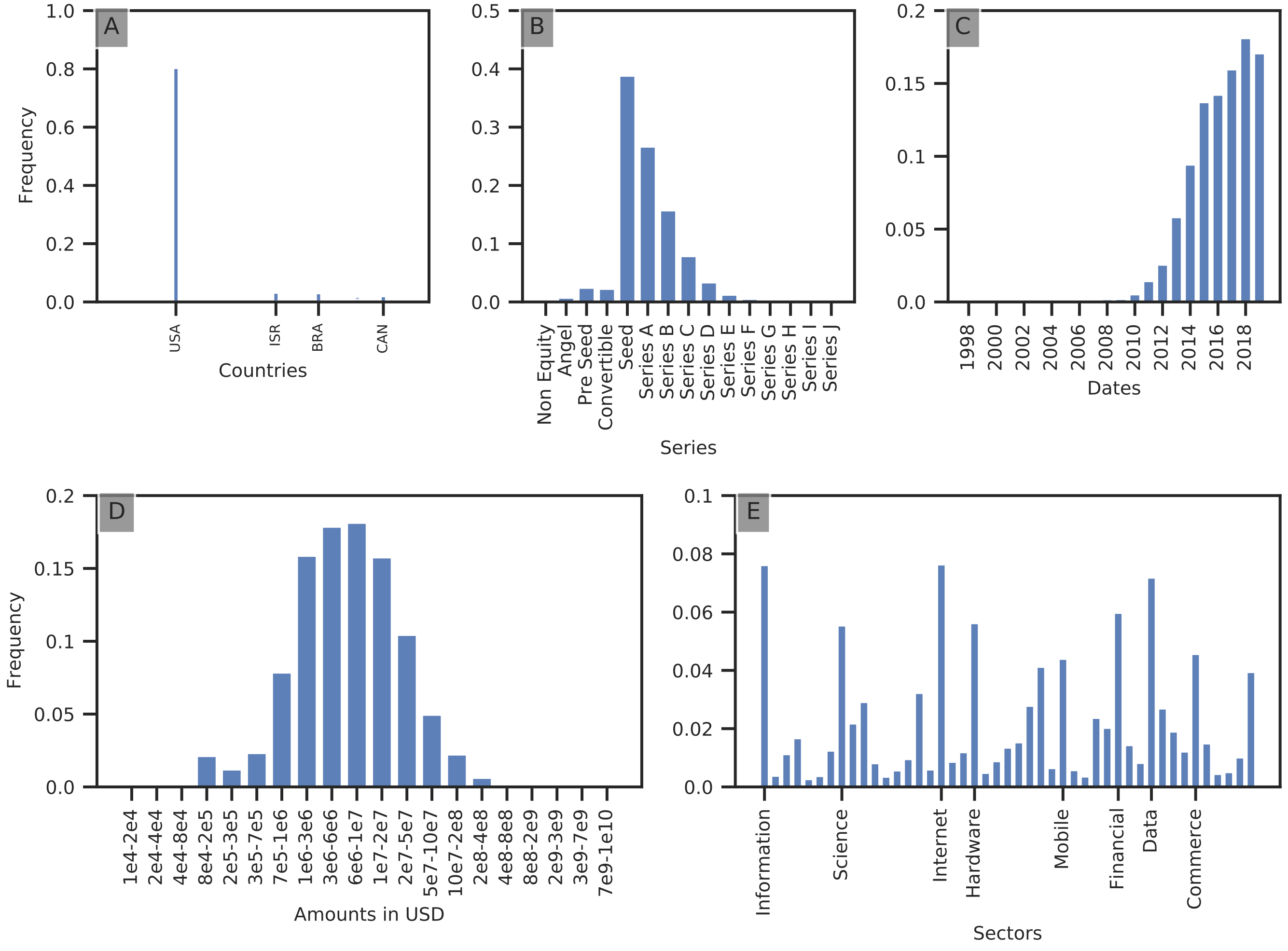

Fig S53. Representative investor of community E5.

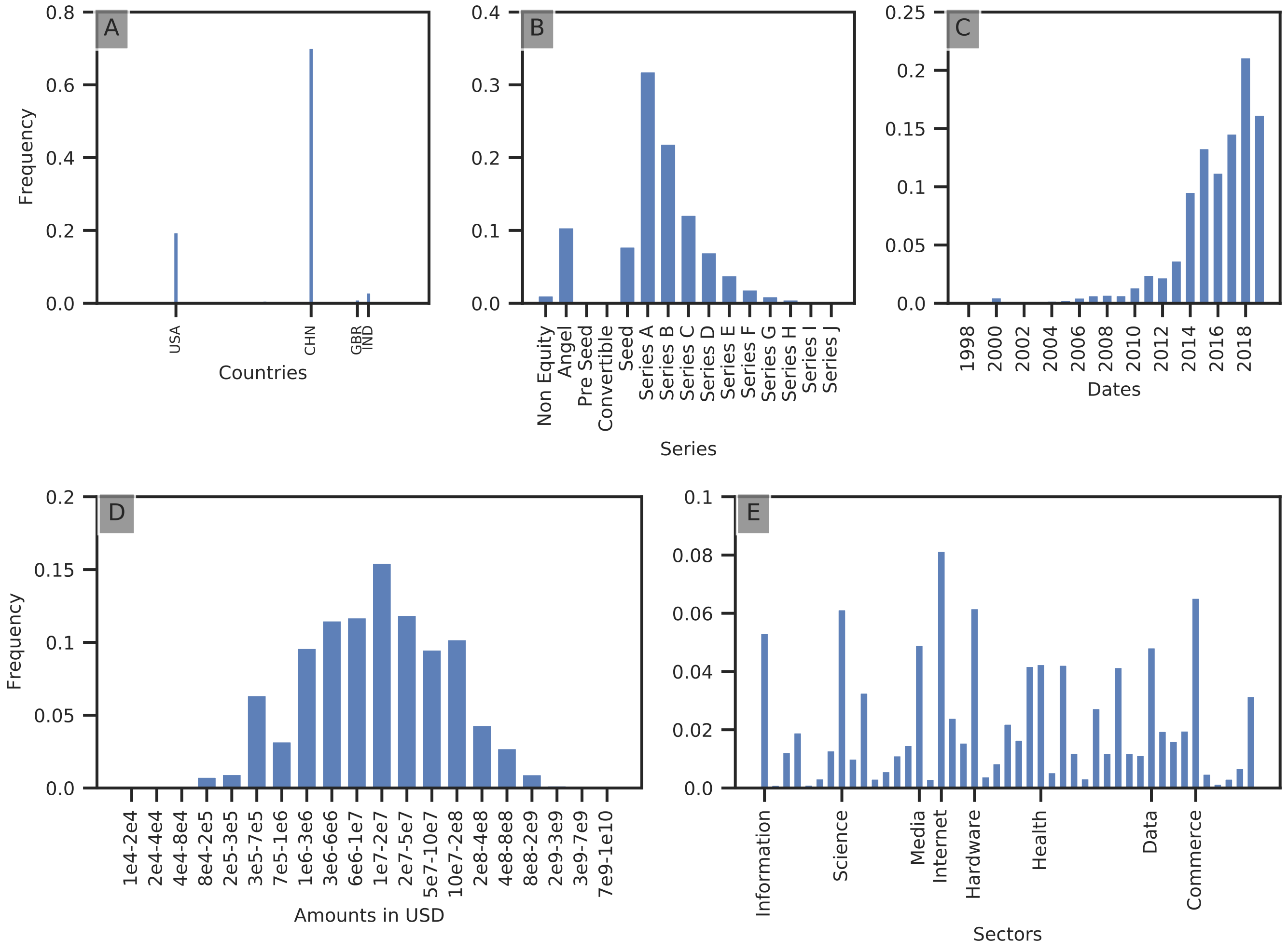

Fig S54. Representative investor of community E6.

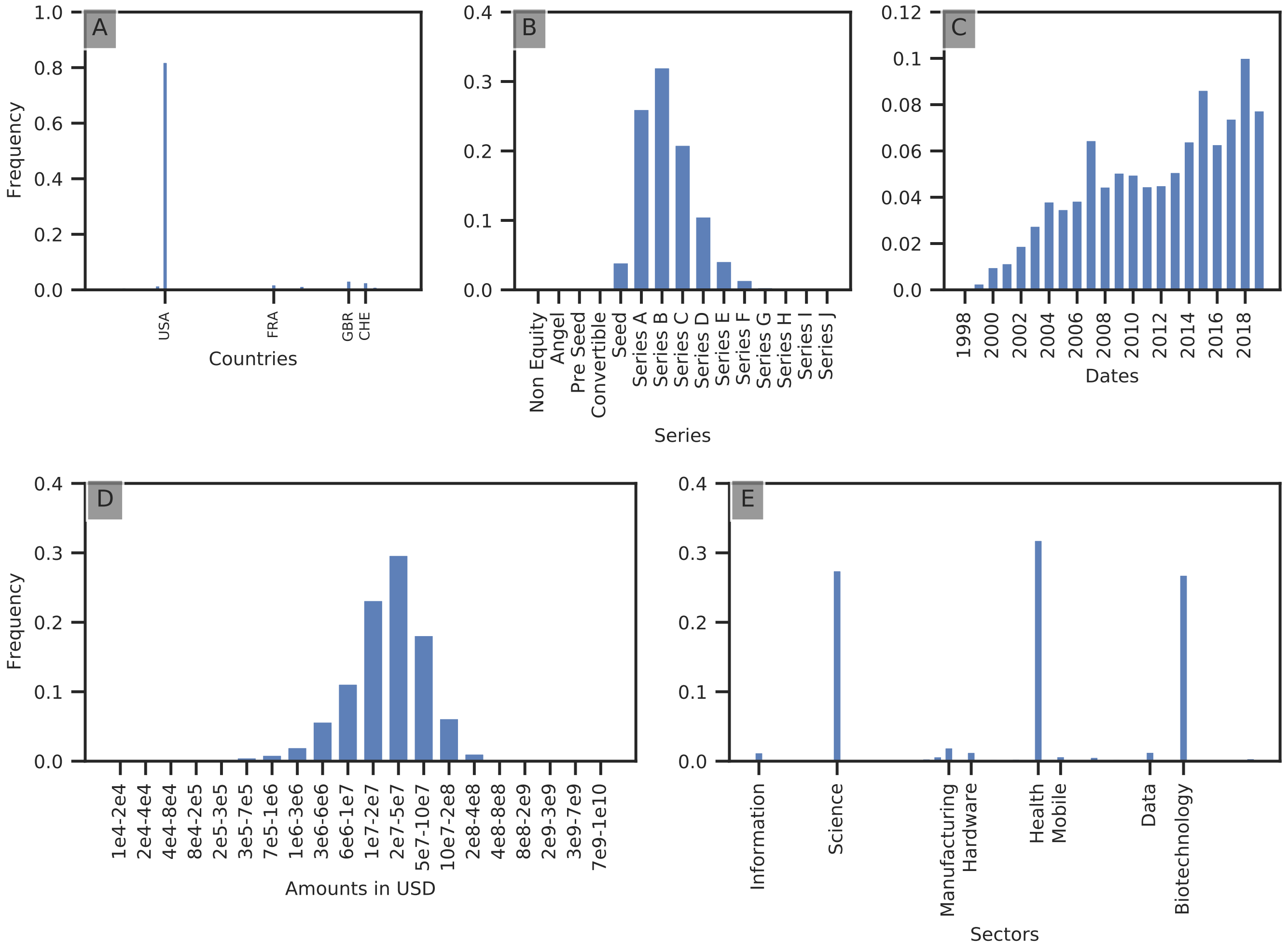

Fig S55. Representative investor of community E7.

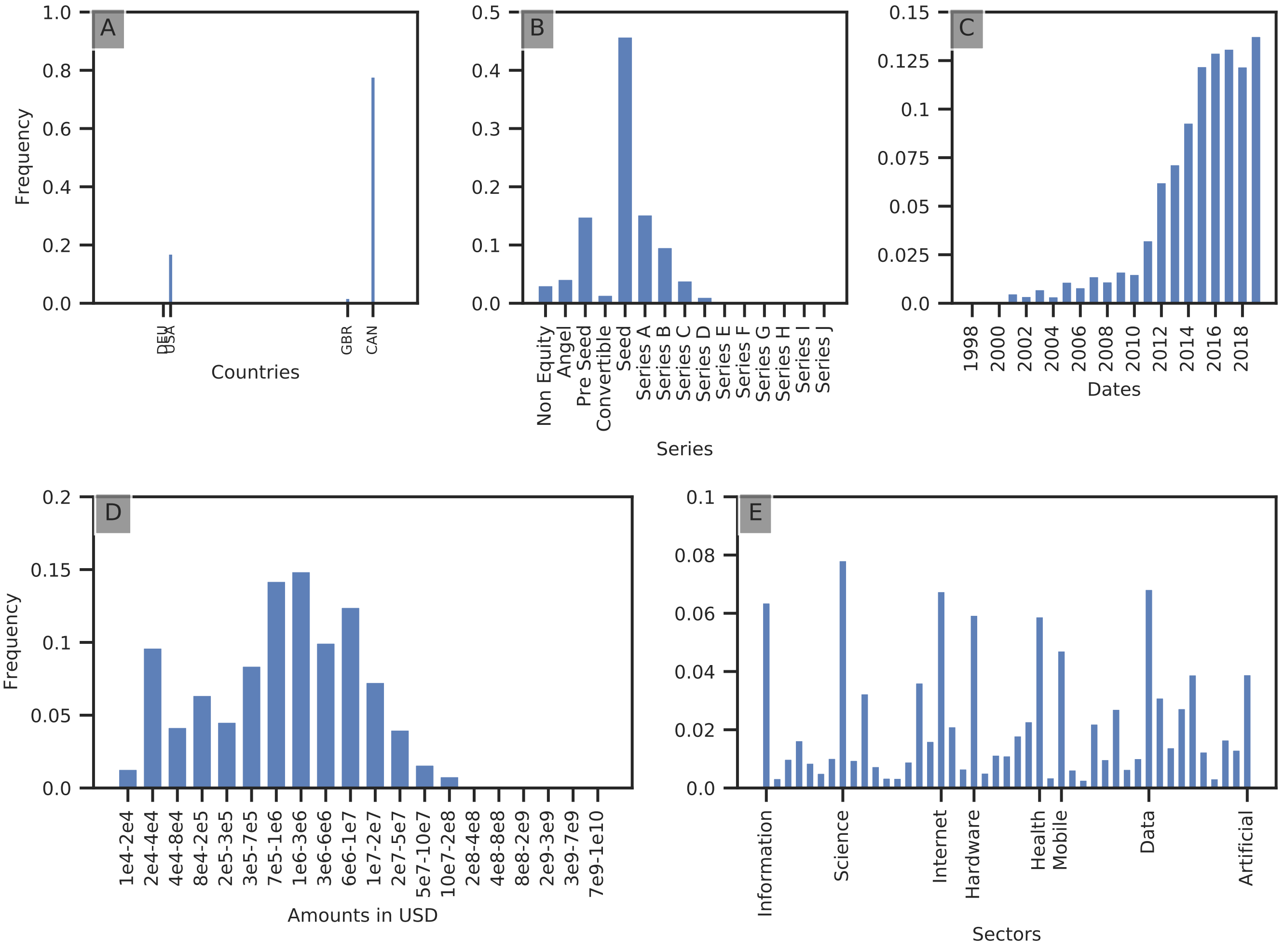

Fig S56. Representative investor of community E8.

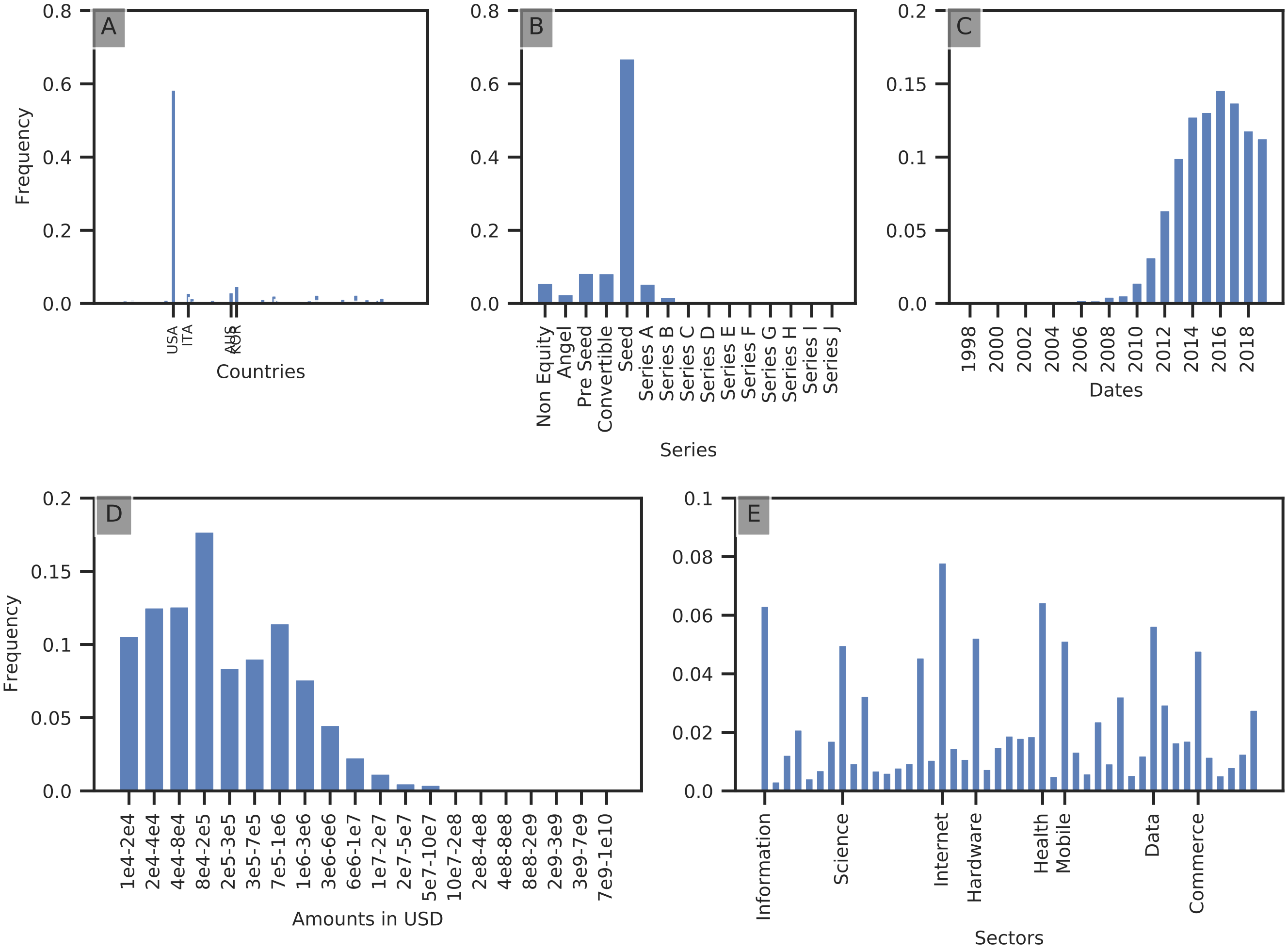

Fig S57. Representative investor of community E9.

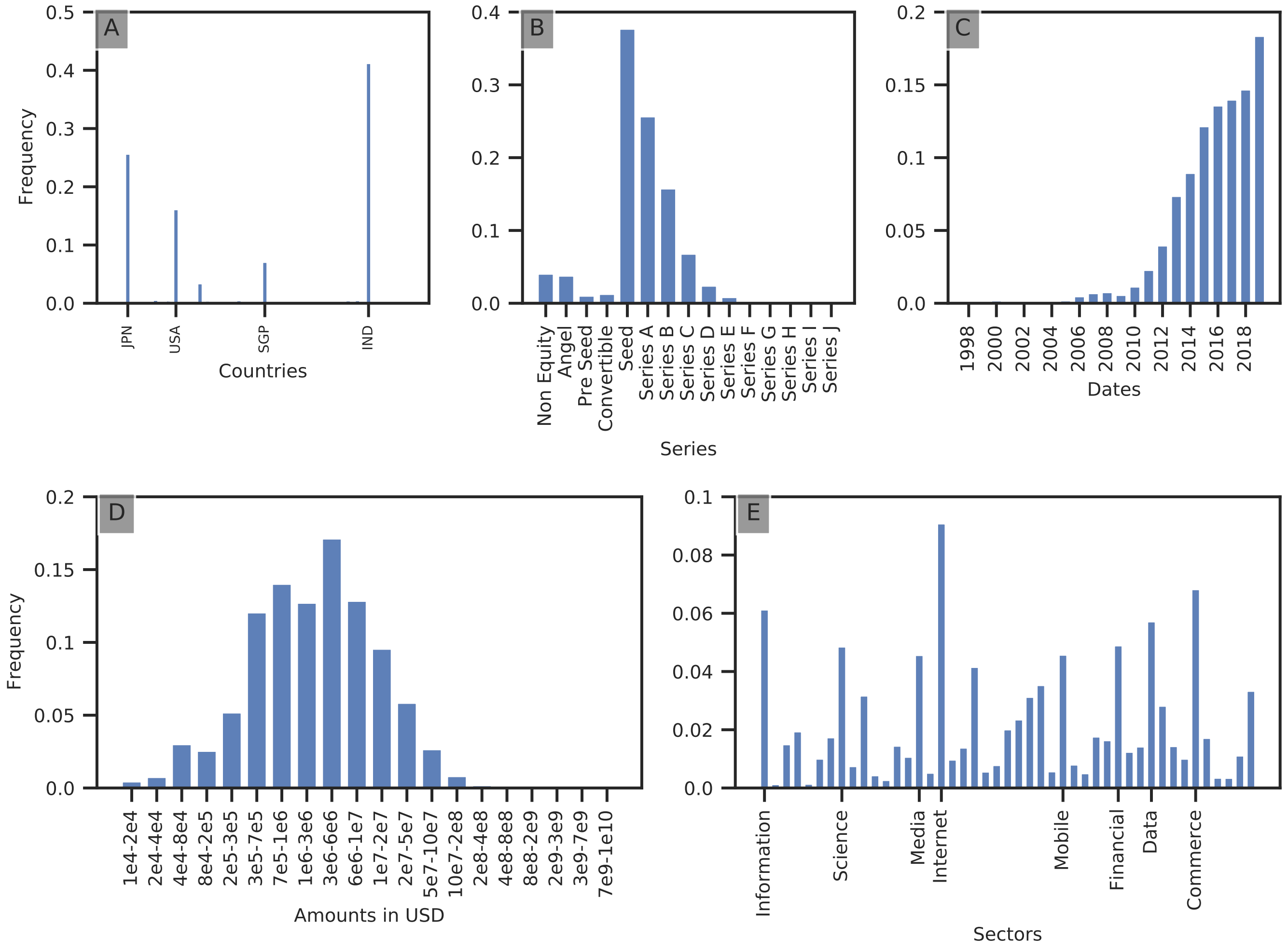

Fig S58. Representative investor of community E10.

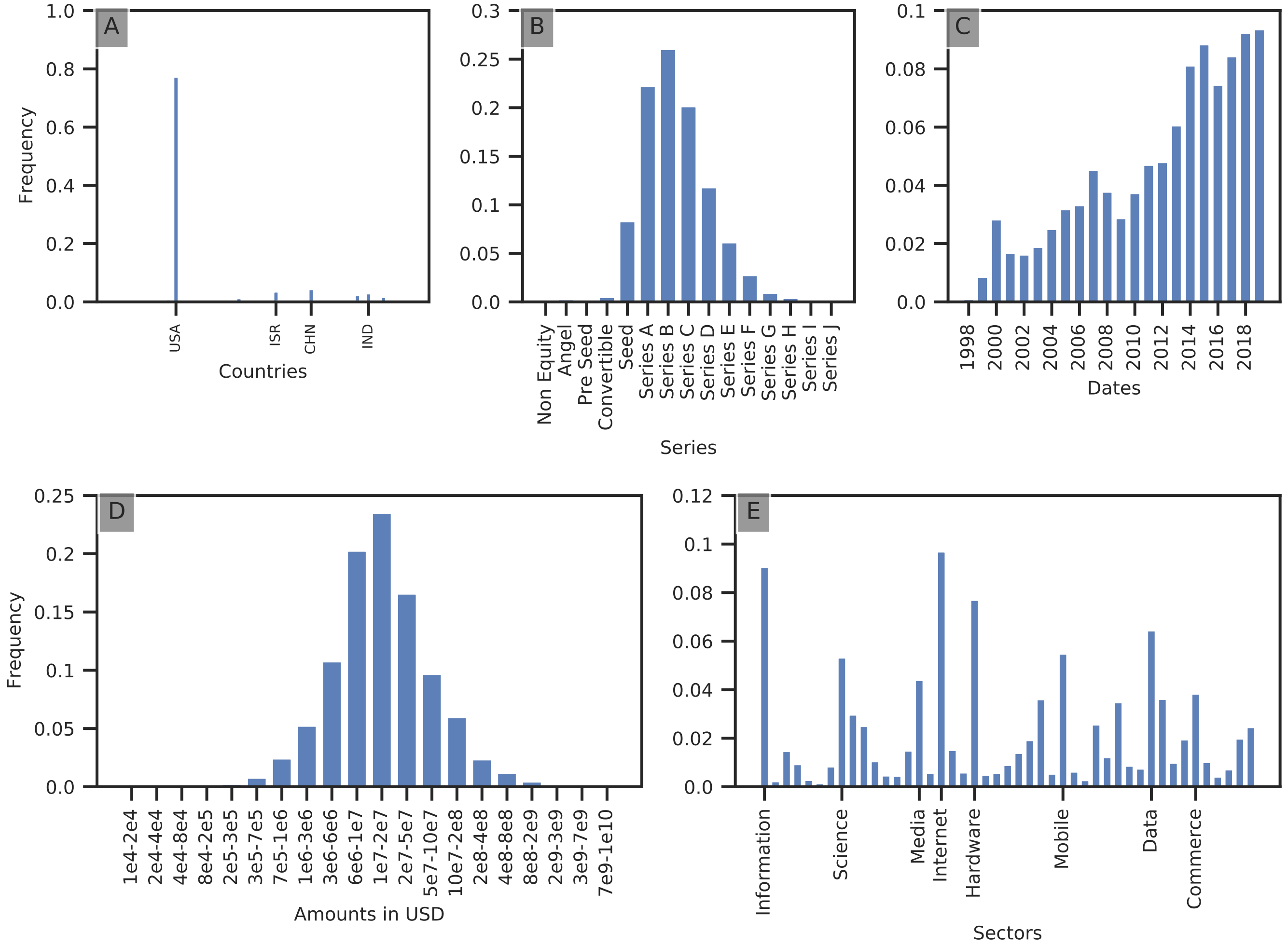

Fig S59. Representative investor of community F0.

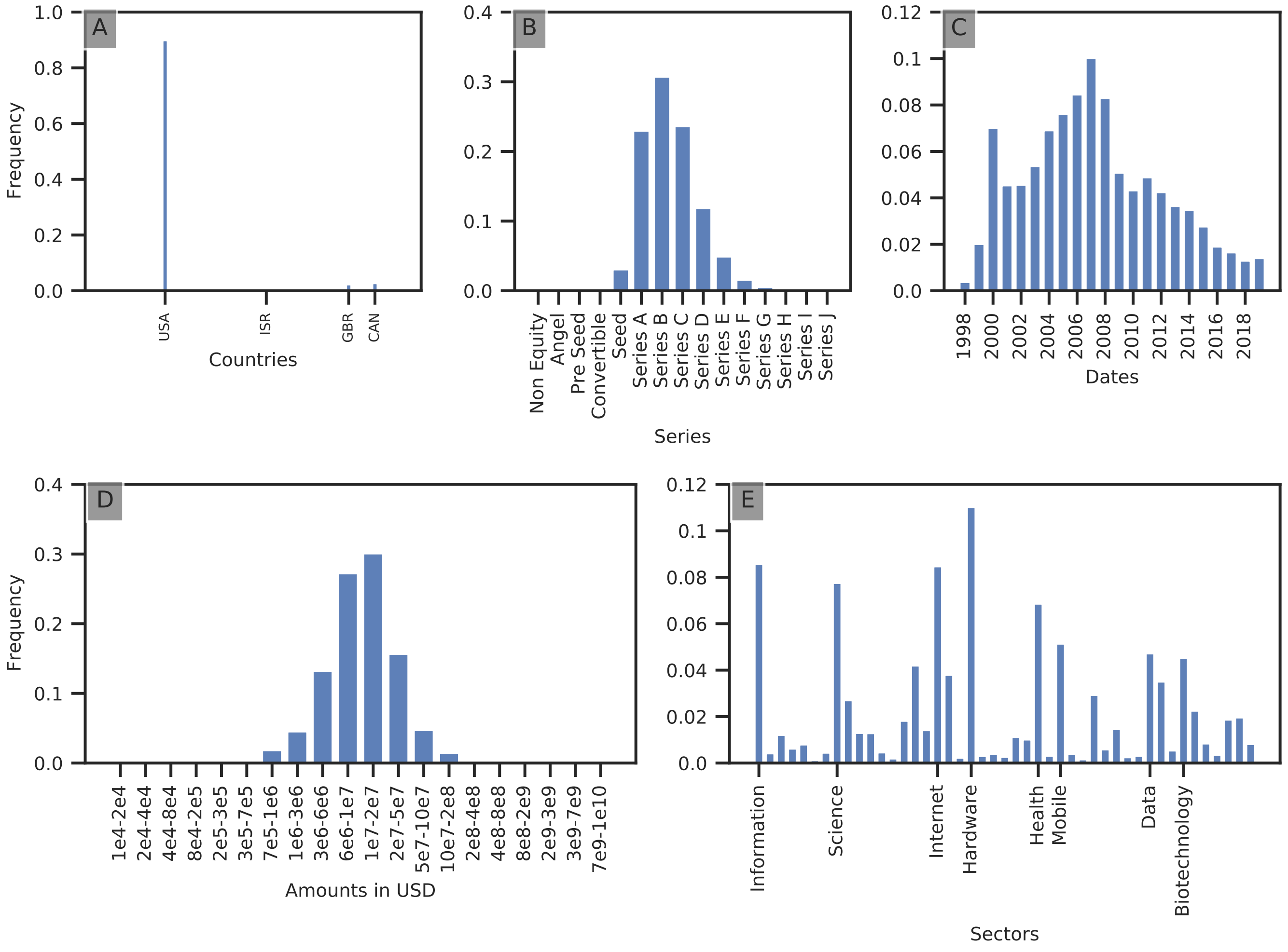

Fig S60. Representative investor of community F1.

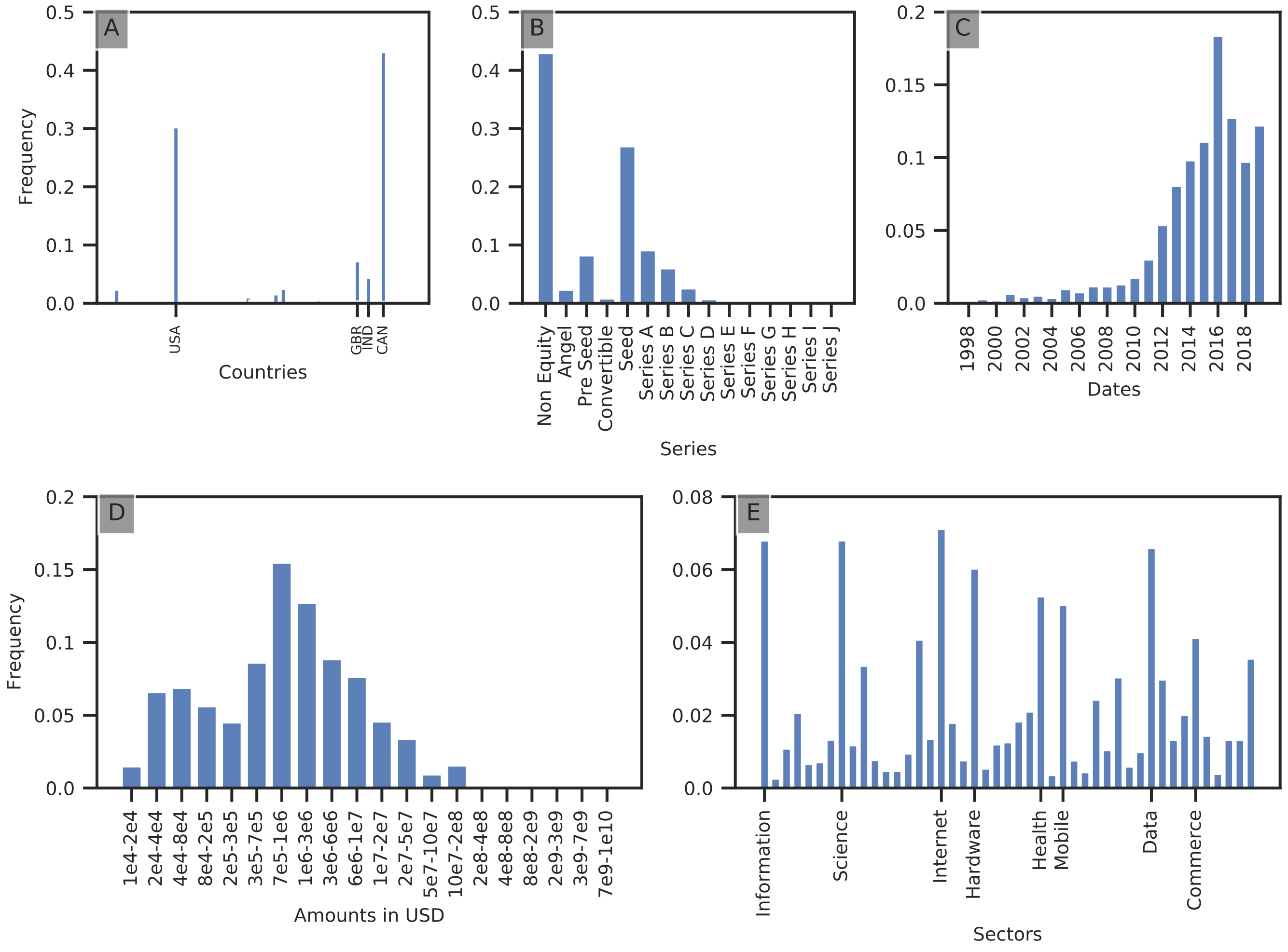

Fig S61. Representative investor of community F2.

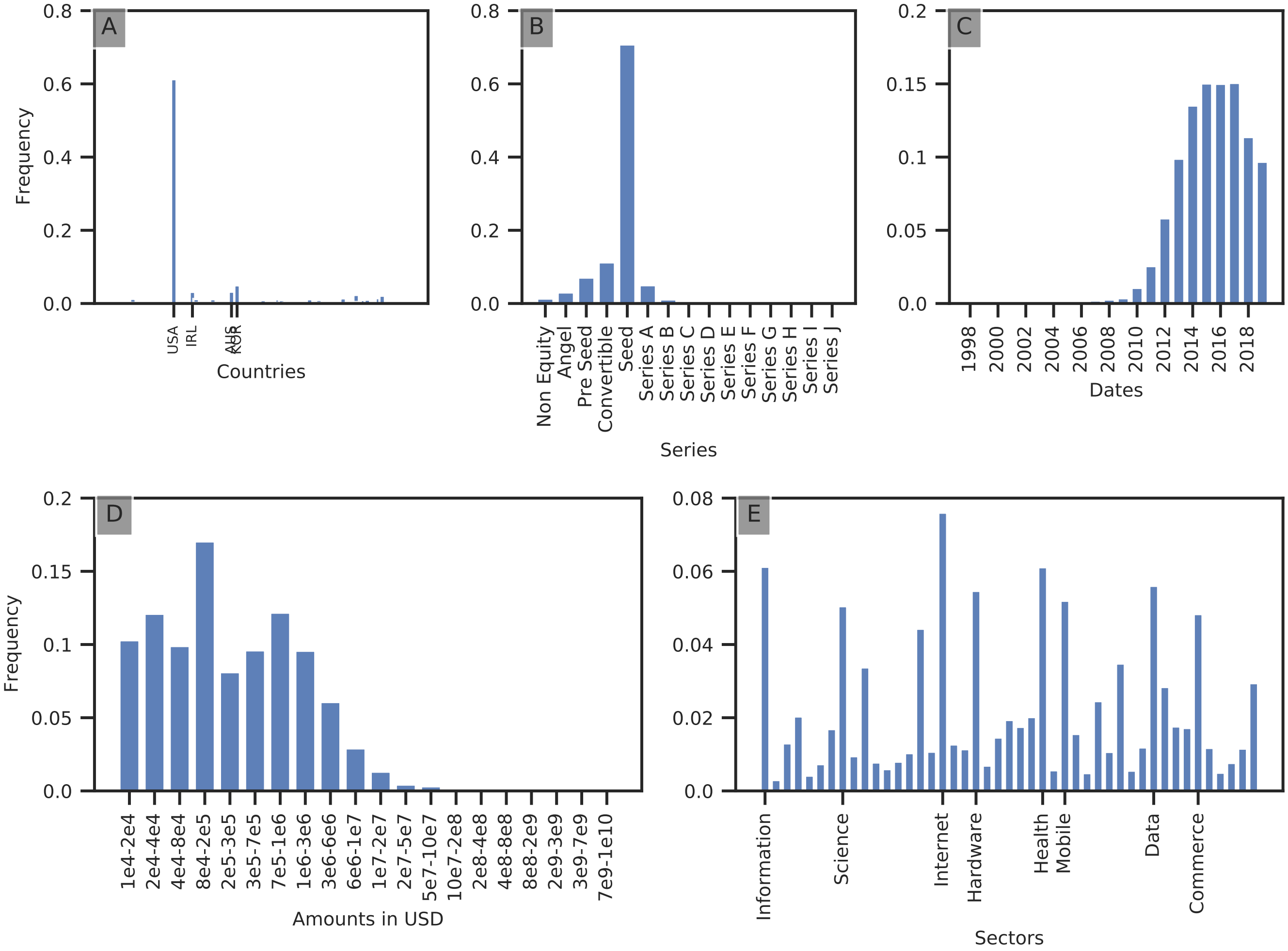

Fig S62. Representative investor of community F3.

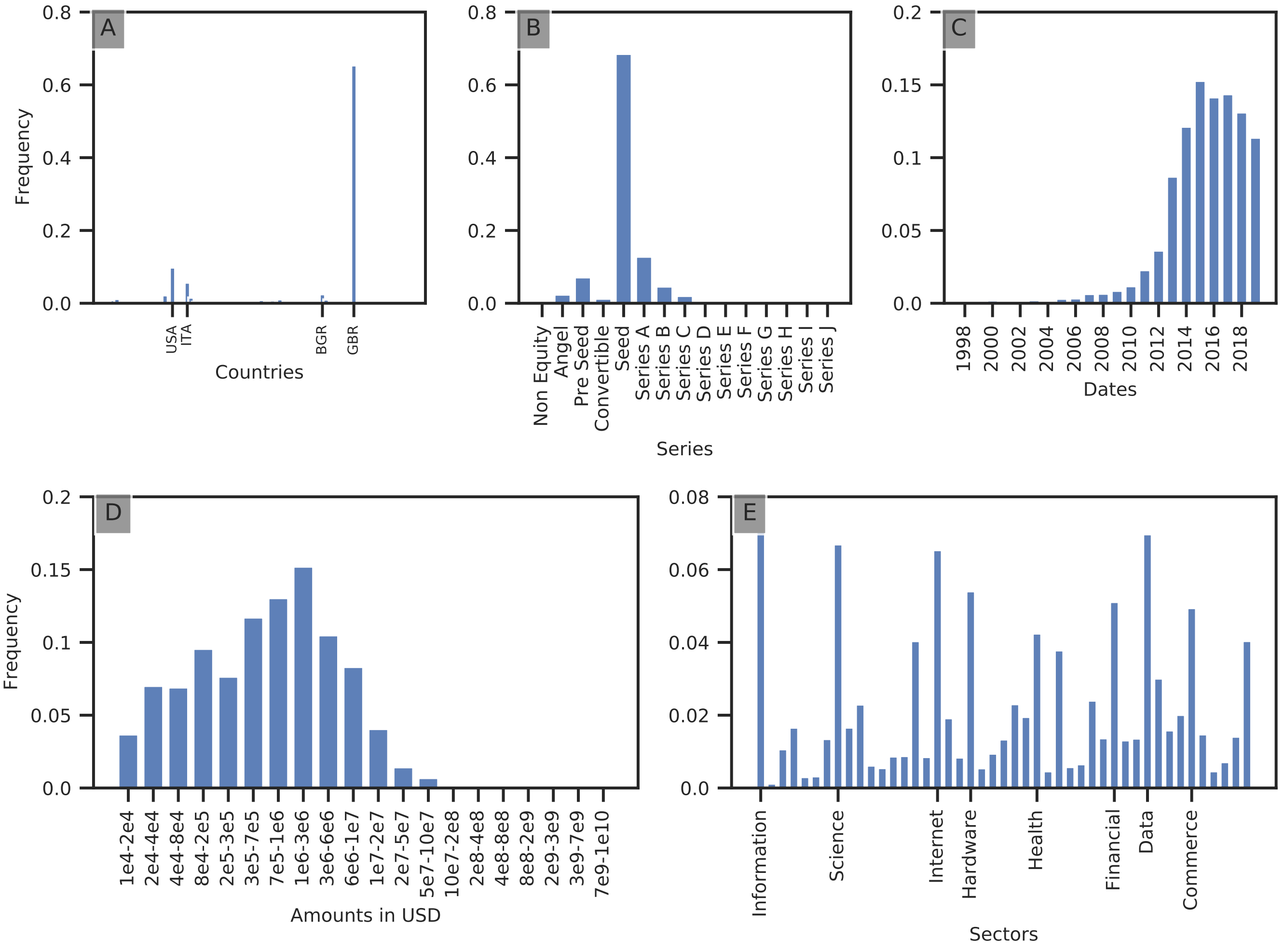

Fig S63. Representative investor of community F4.

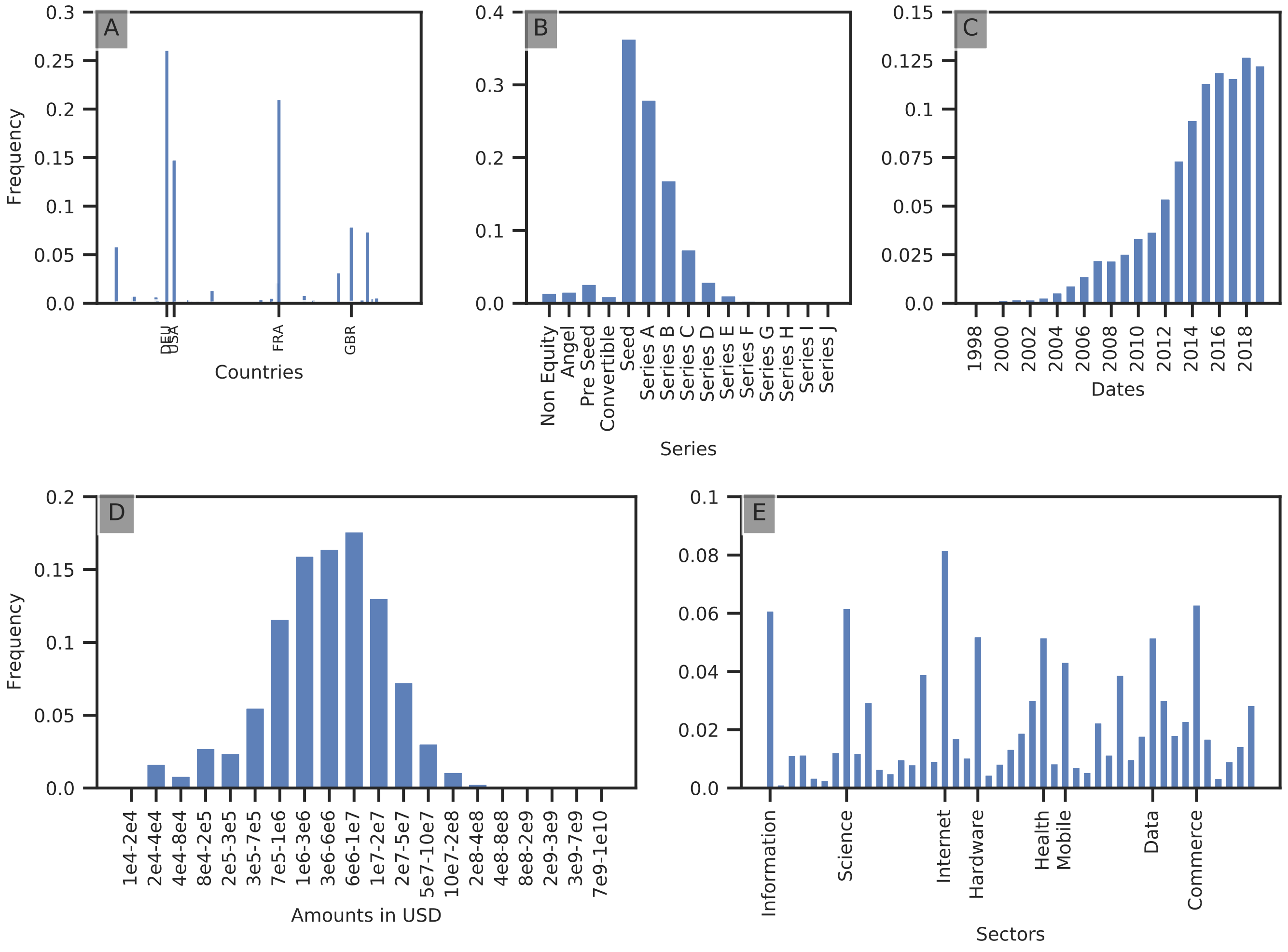

Fig S64. Representative investor of community F5.

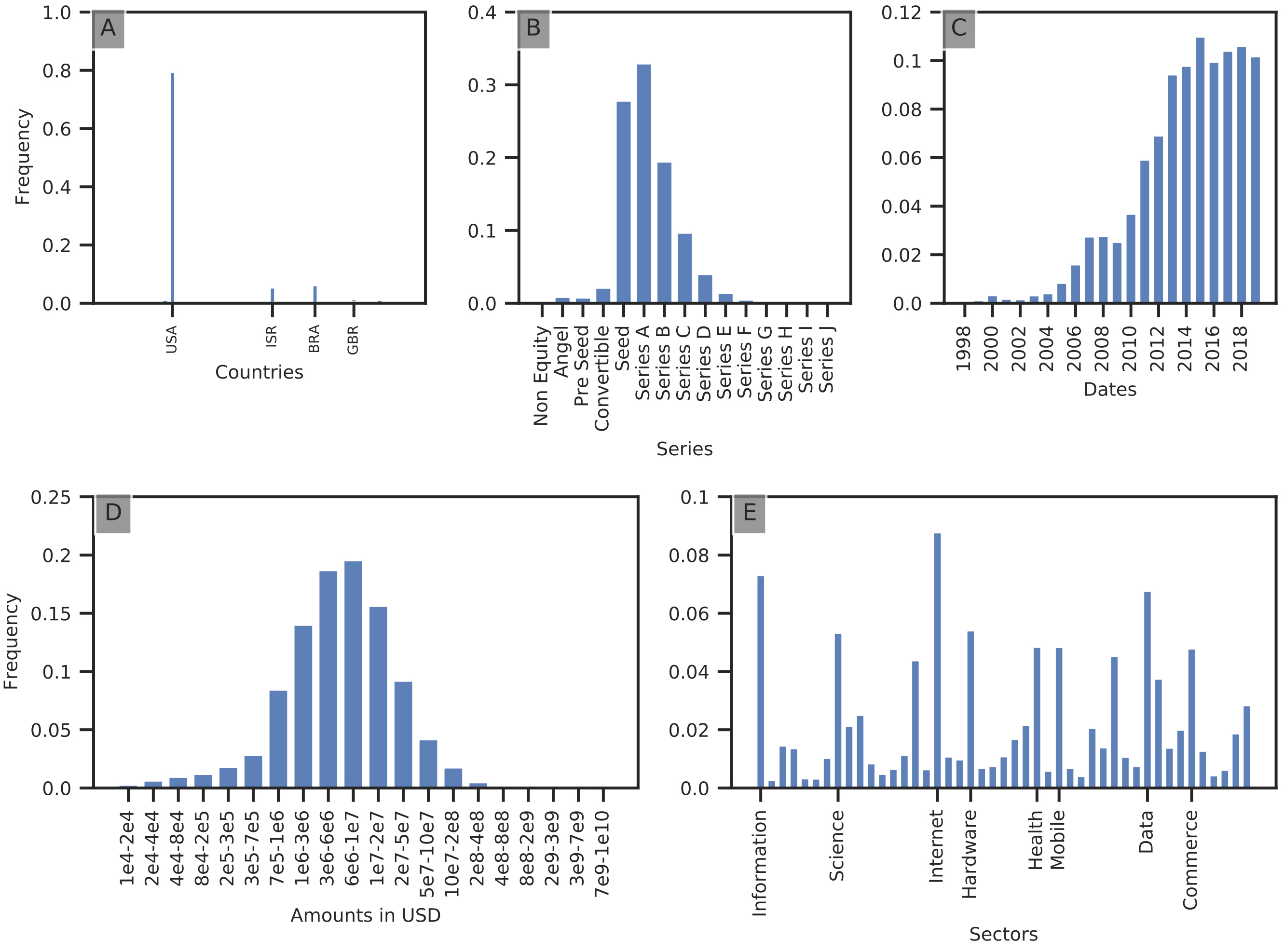

Fig S65. Representative investor of community F6.

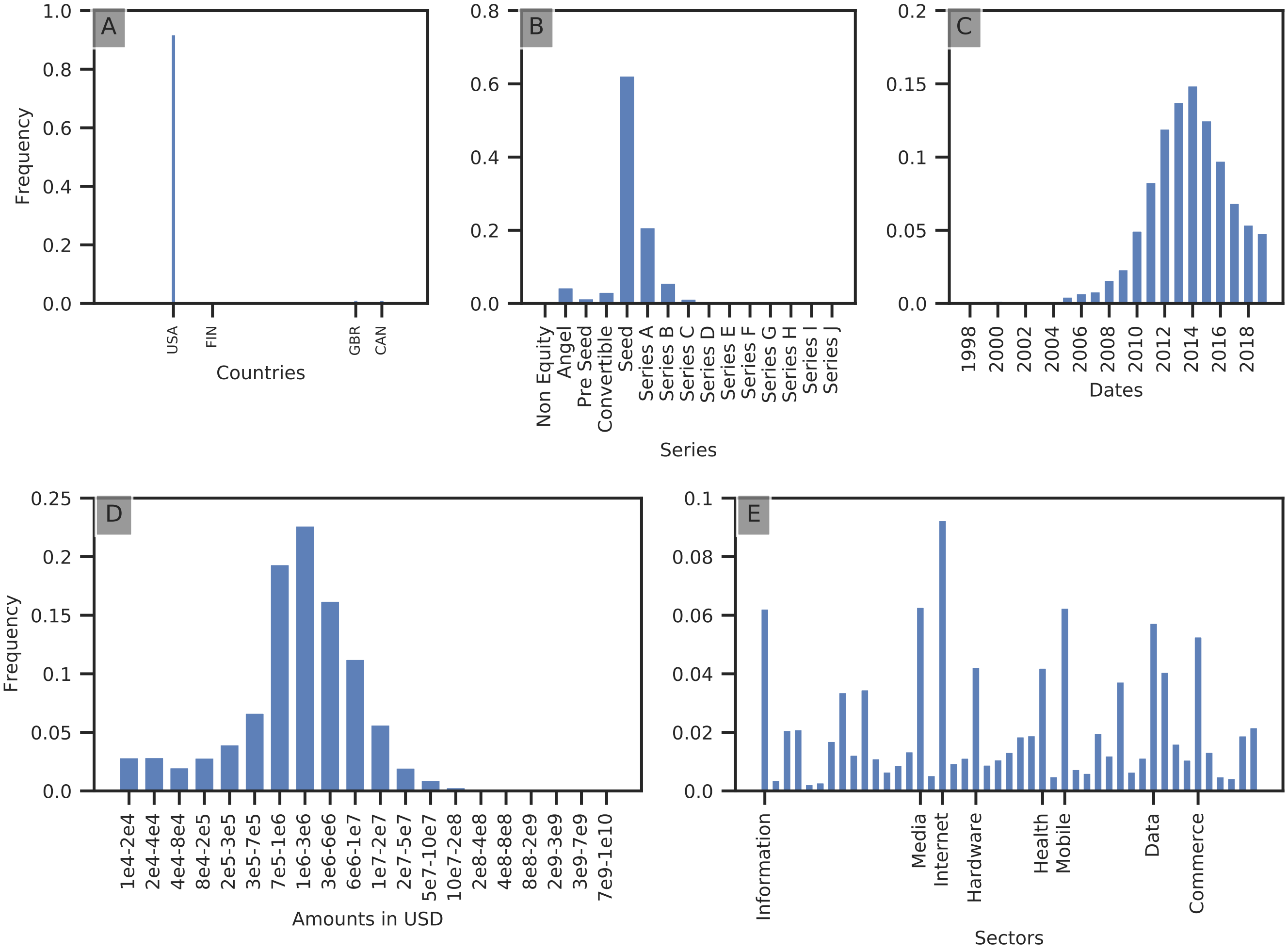

Fig S66. Representative investor of community F7.

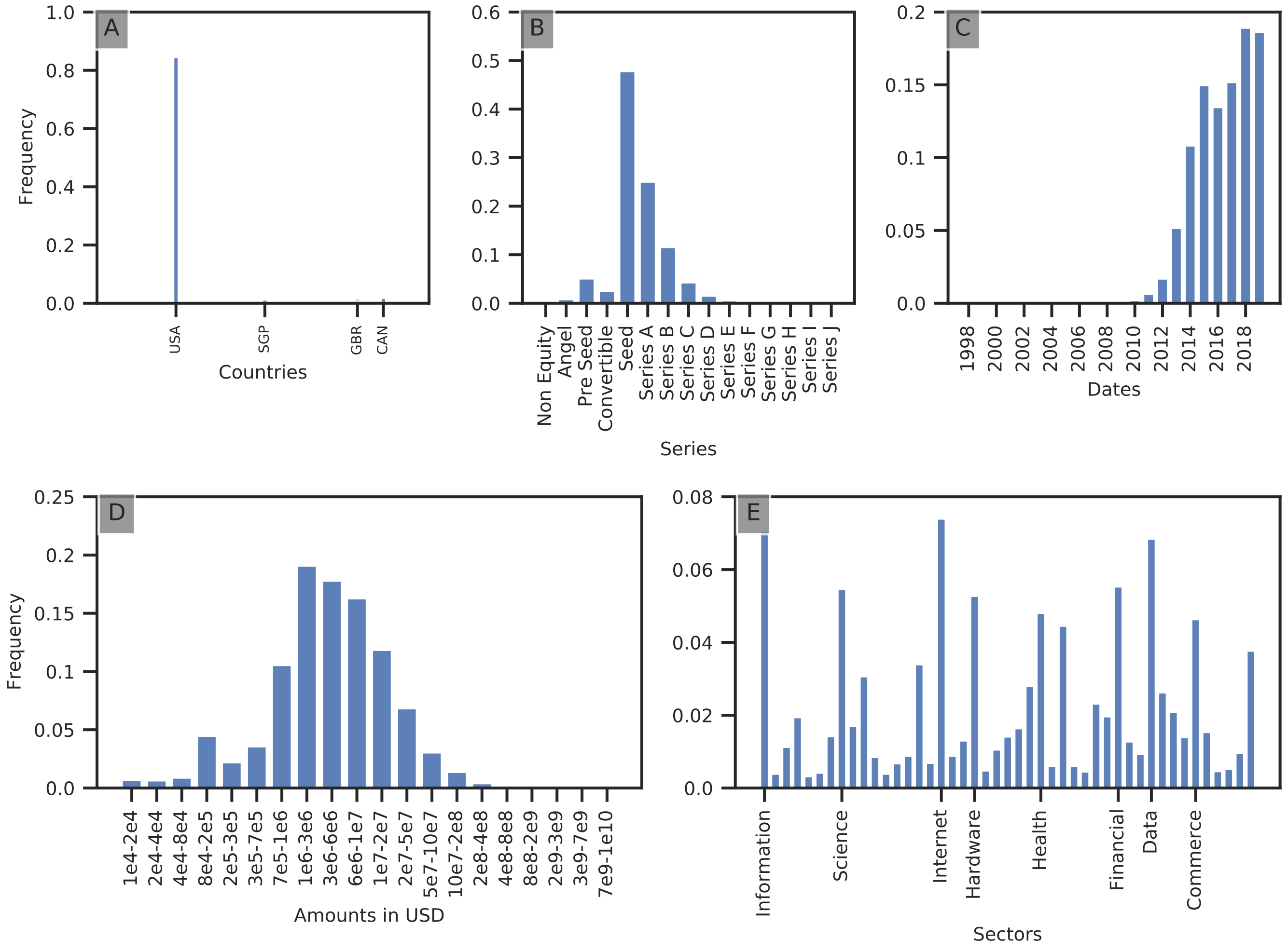

Fig S67. Representative investor of community F8.

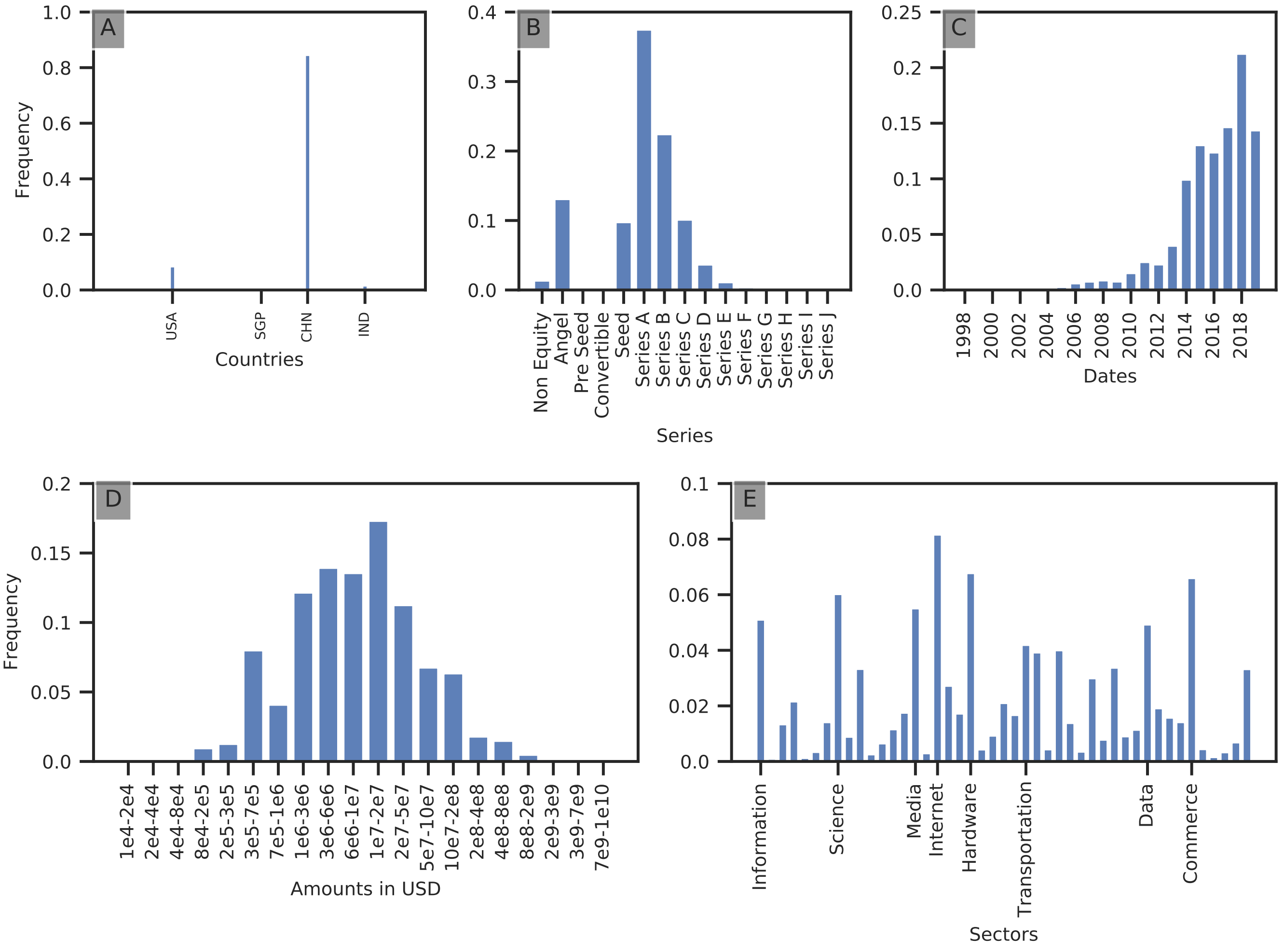

Fig S68. Representative investor of community F9.

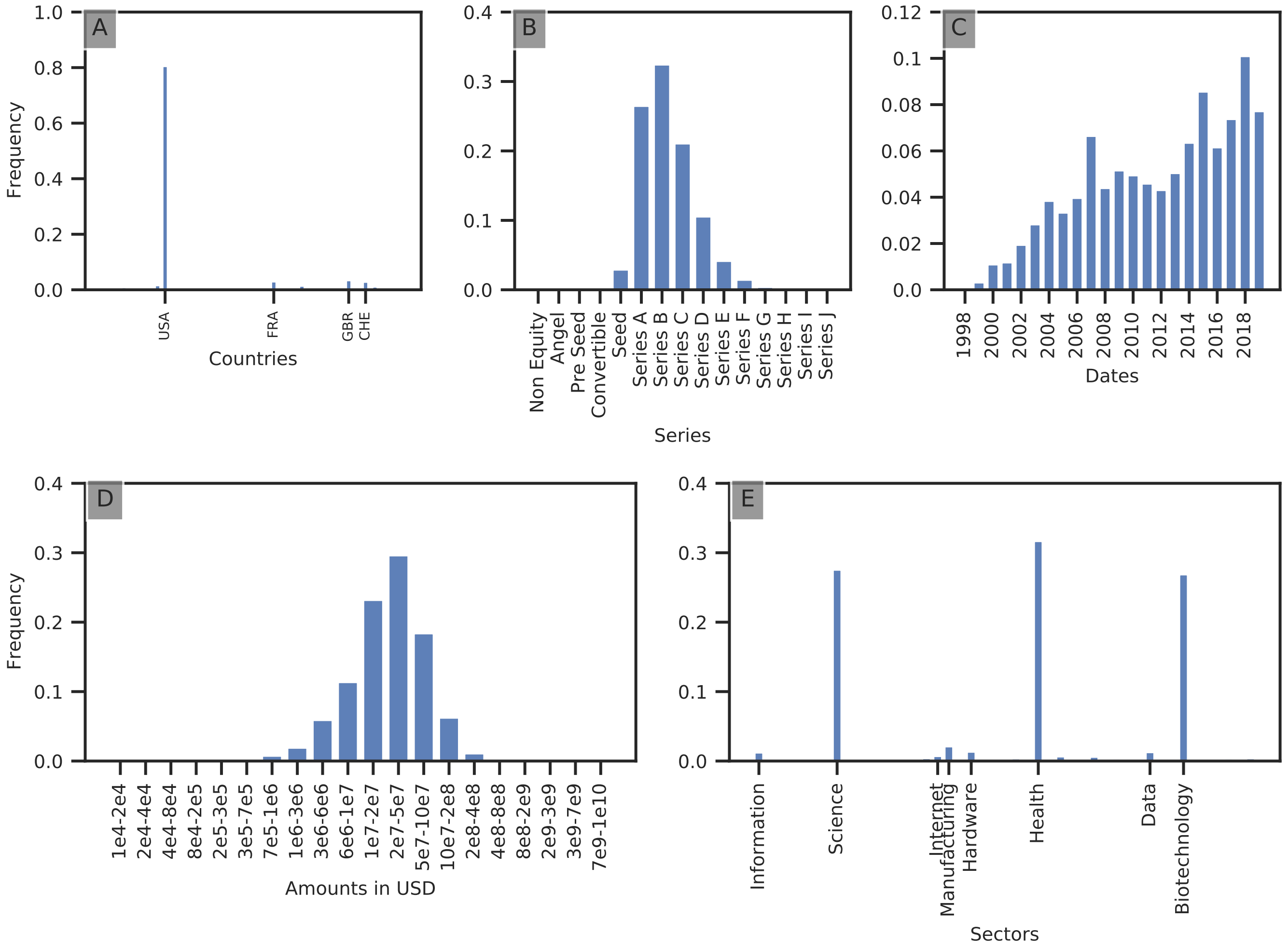

Fig S69. Representative investor of community F10.

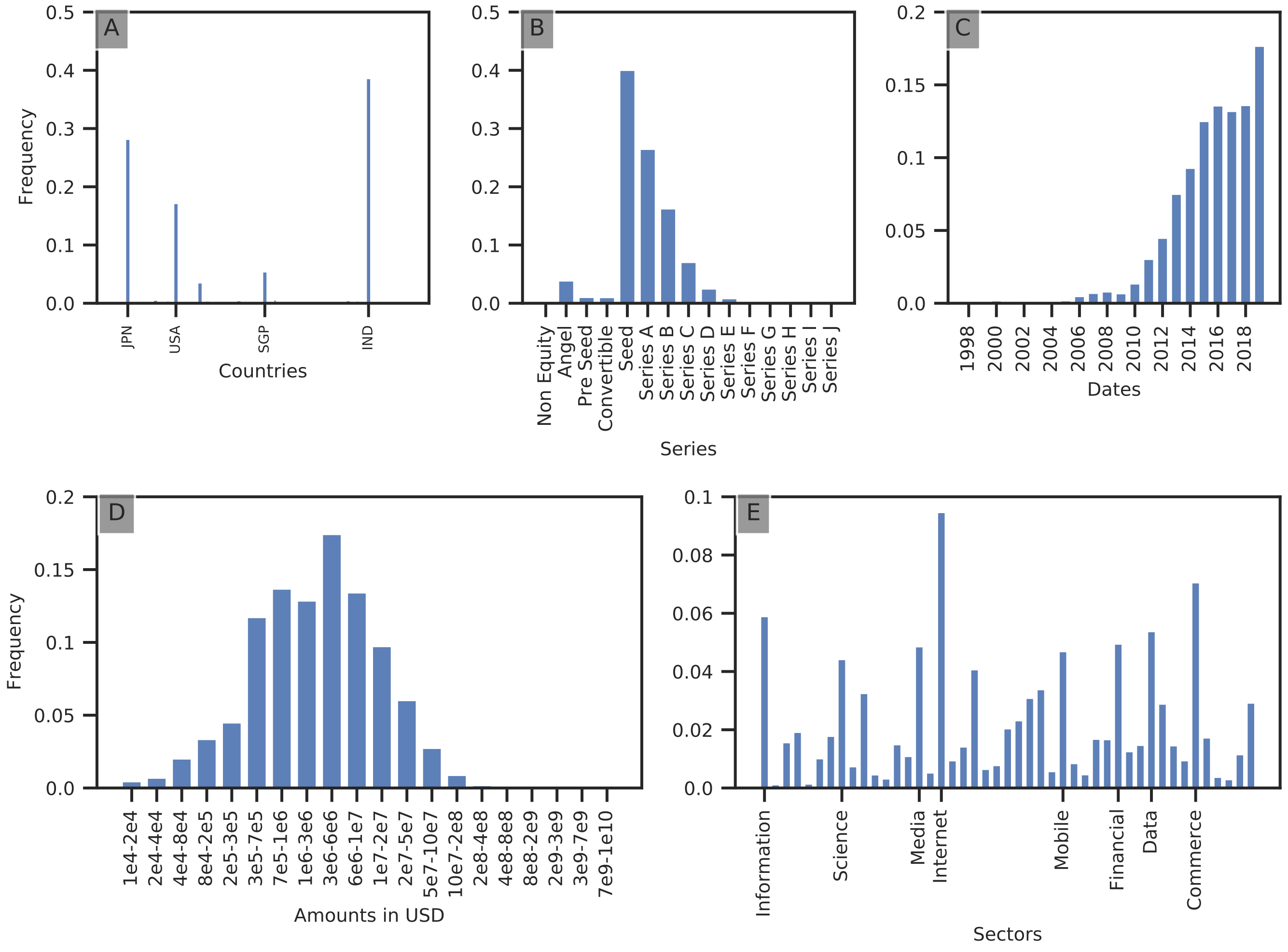

Fig S70. Representative investor of community F11.

Cross-interaction heatmaps of all communities

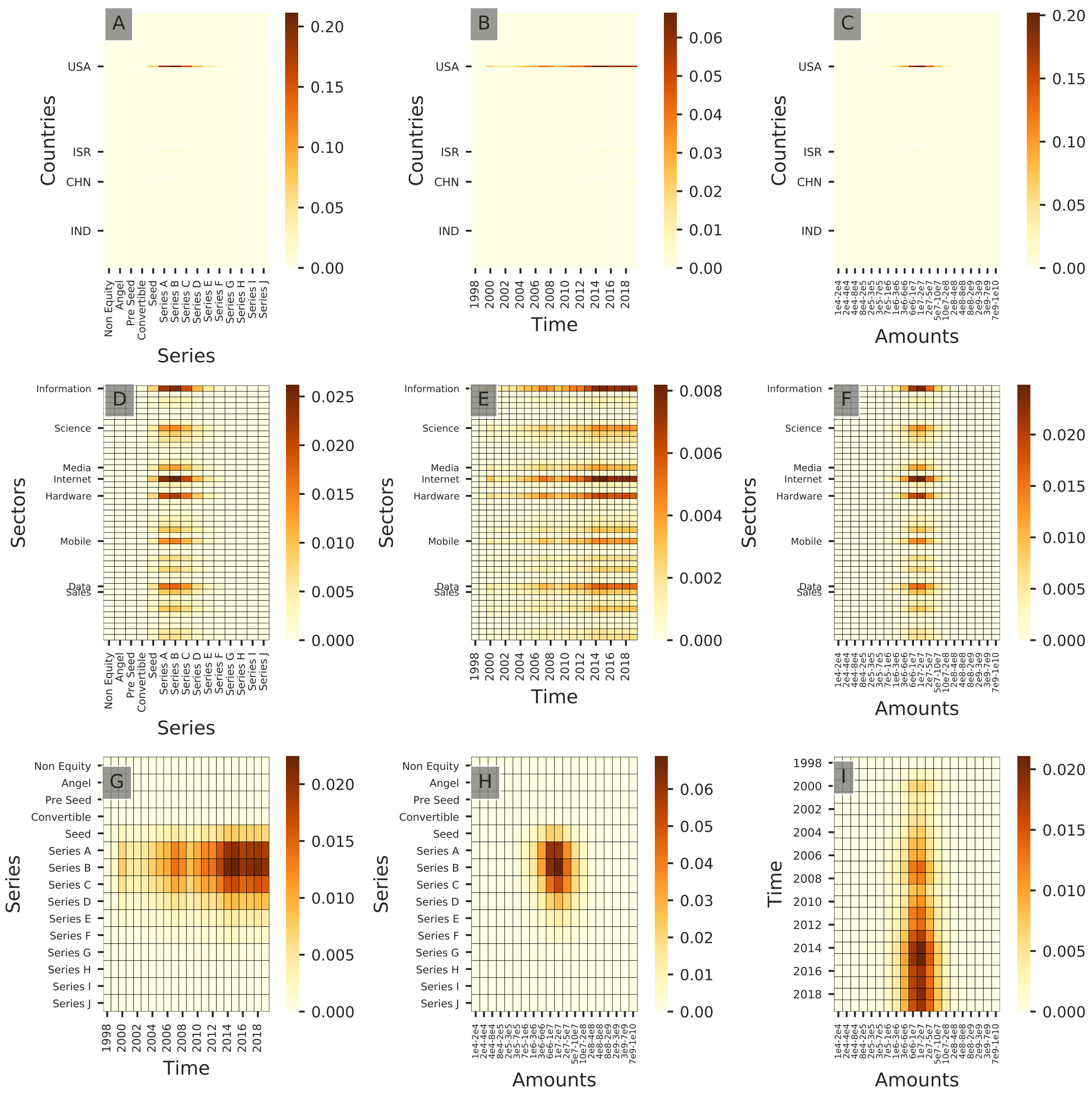

Fig S71. Cross graph interaction heatmap of community A0.

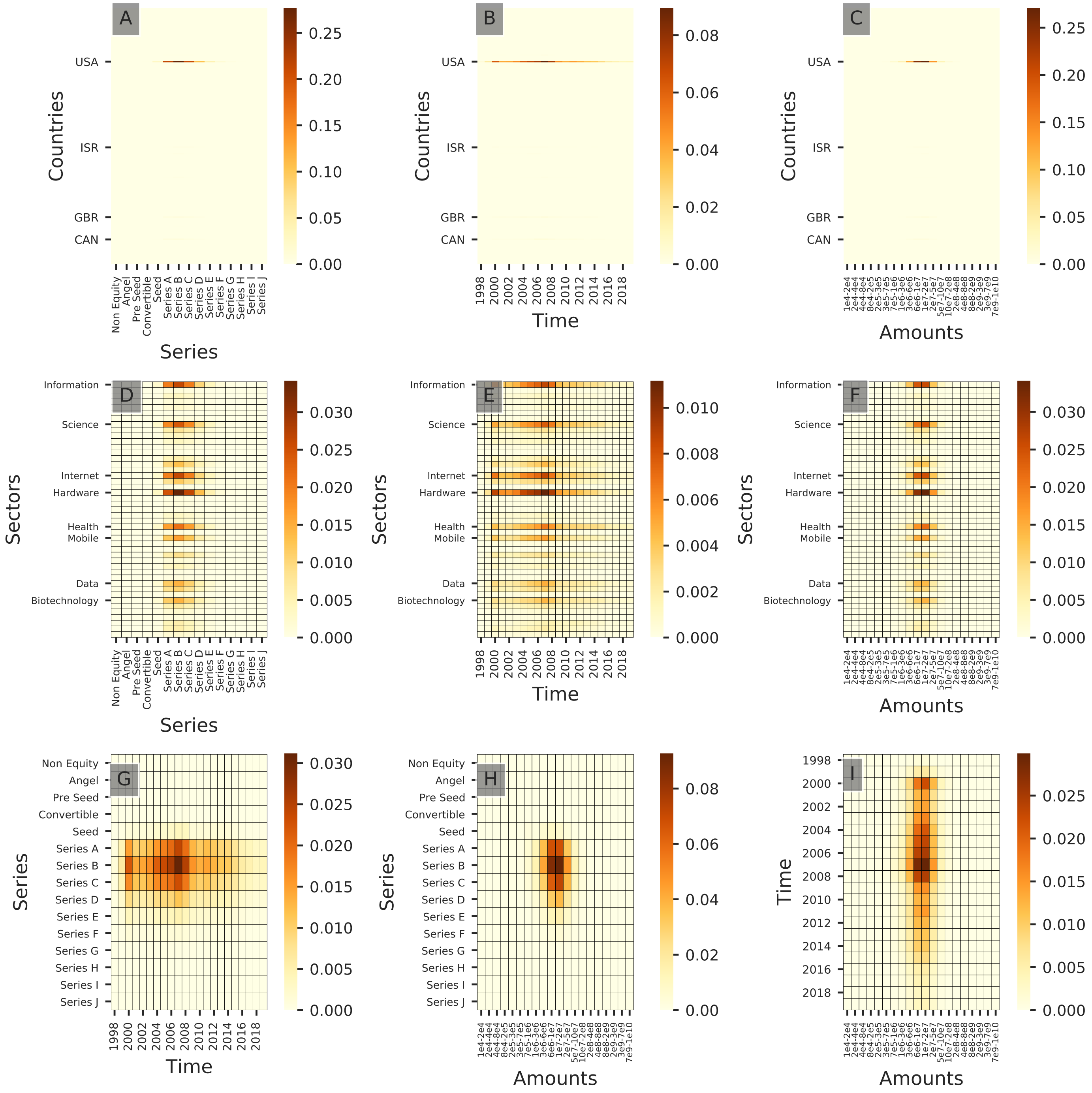

Fig S72. Cross graph interaction heatmap of community A1.

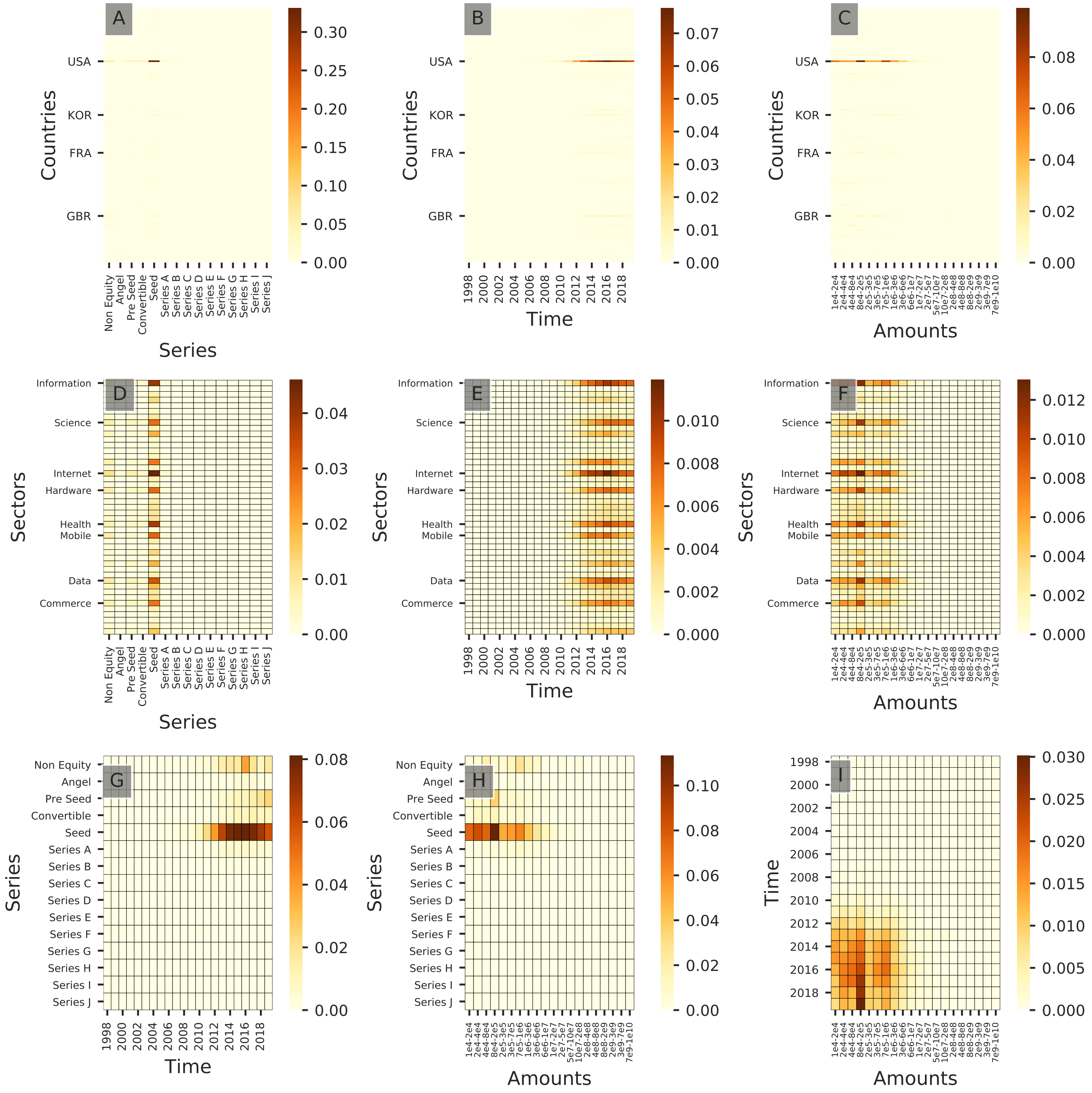

Fig S73. Cross graph interaction heatmap of community A2.

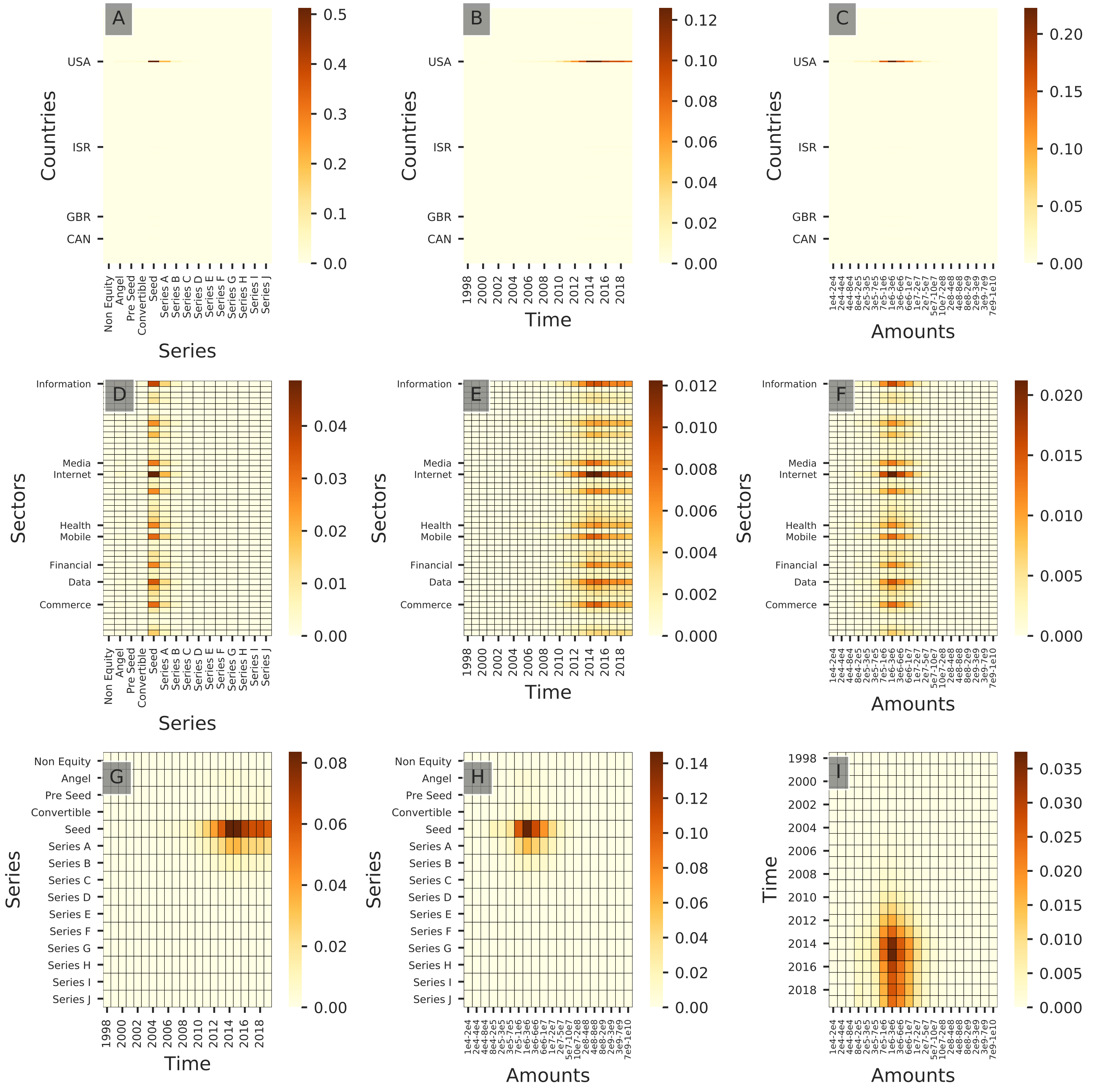

Fig S74. Cross graph interaction heatmap of community A3.

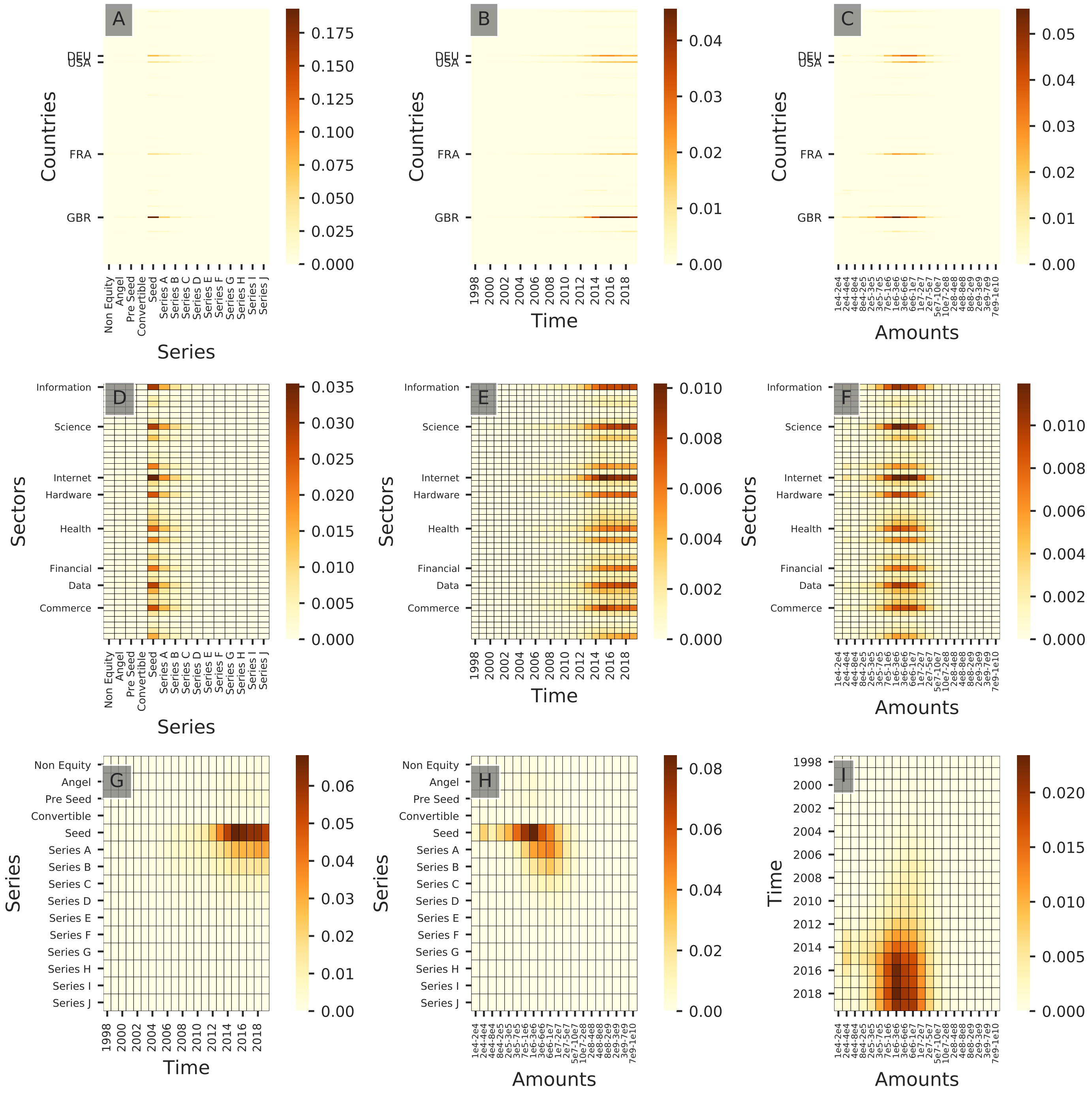

Fig S75. Cross graph interaction heatmap of community A4.

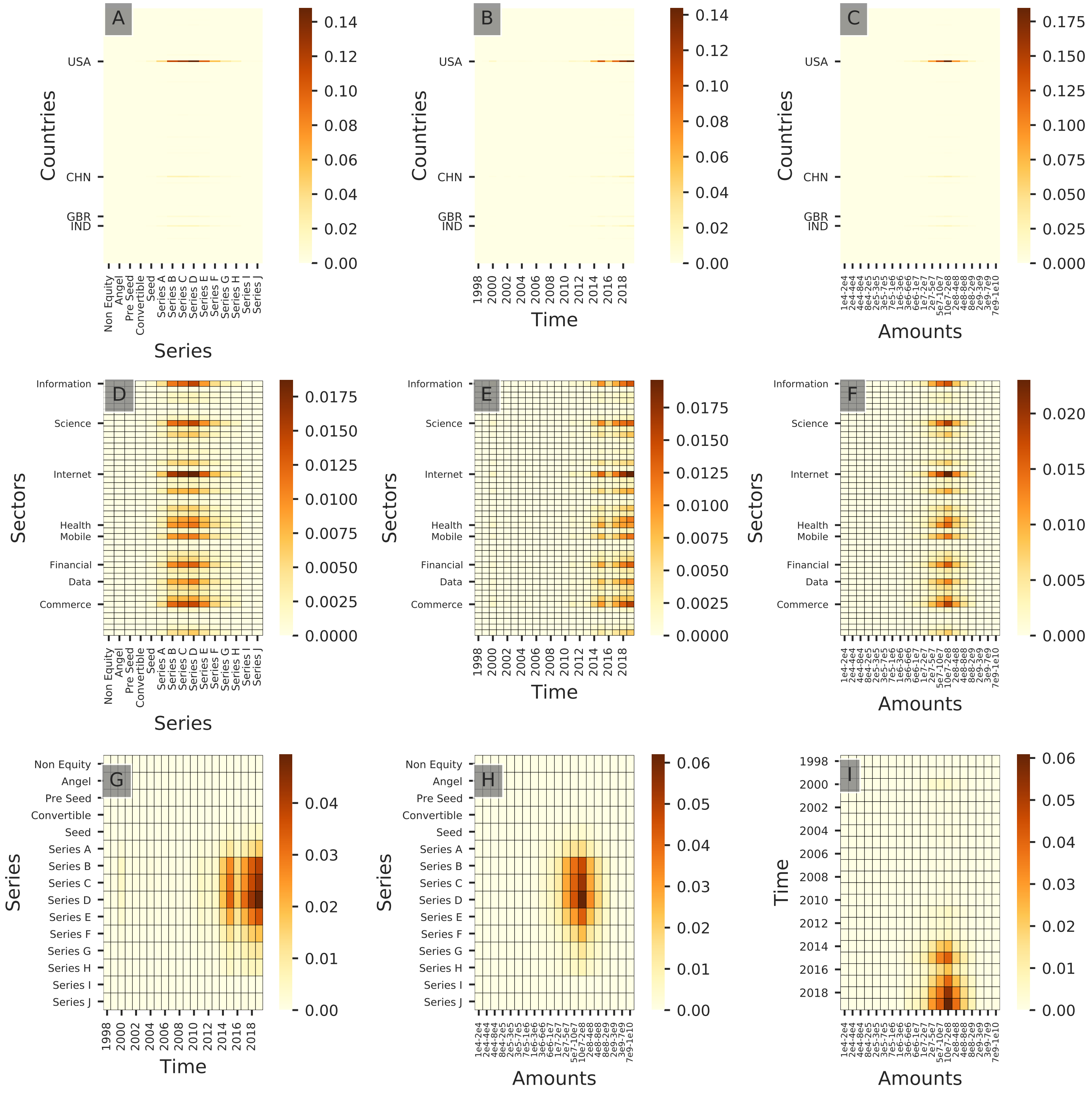

Fig S76. Cross graph interaction heatmap of community A5.

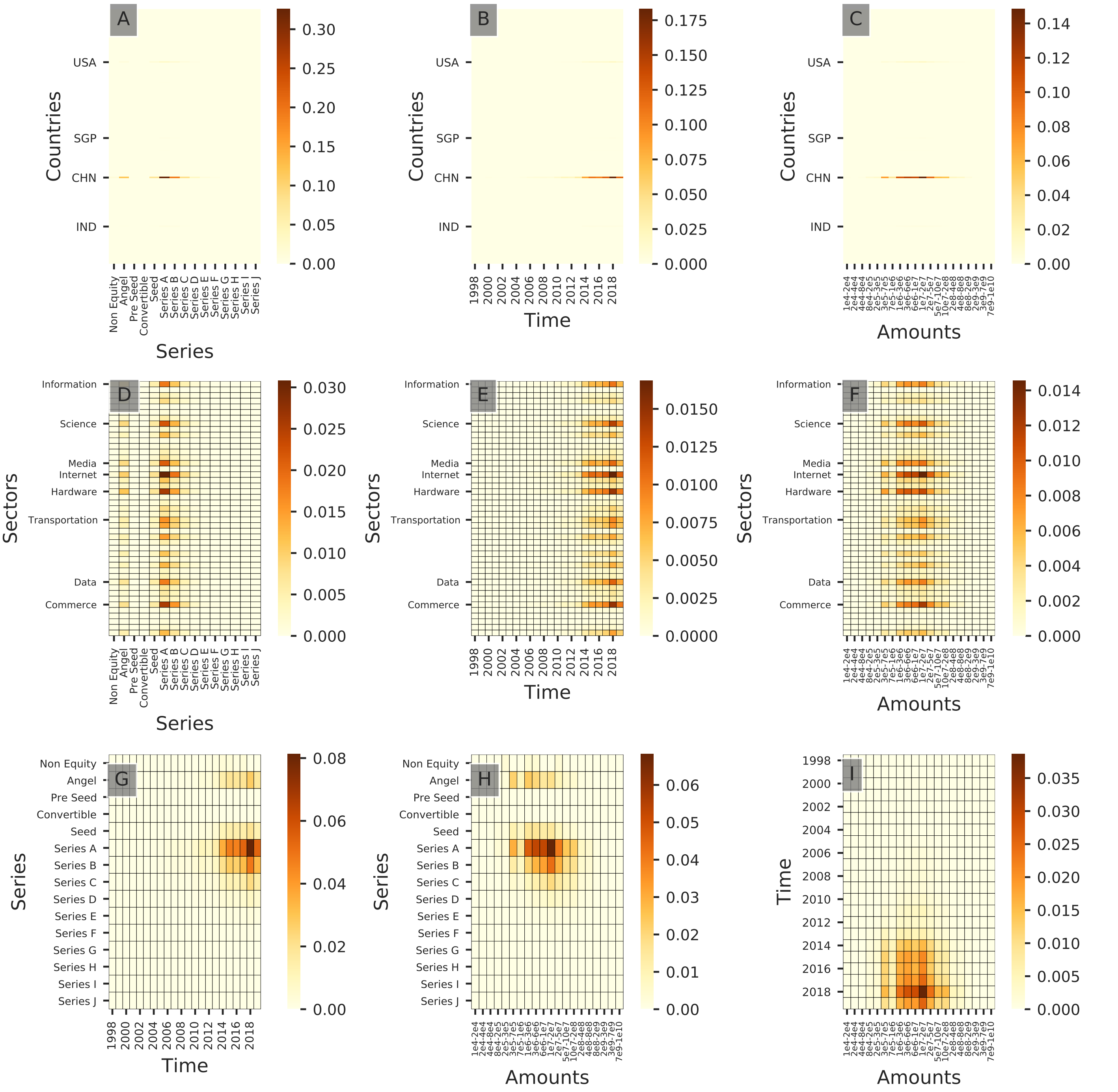

Fig S77. Cross graph interaction heatmap of community A6.

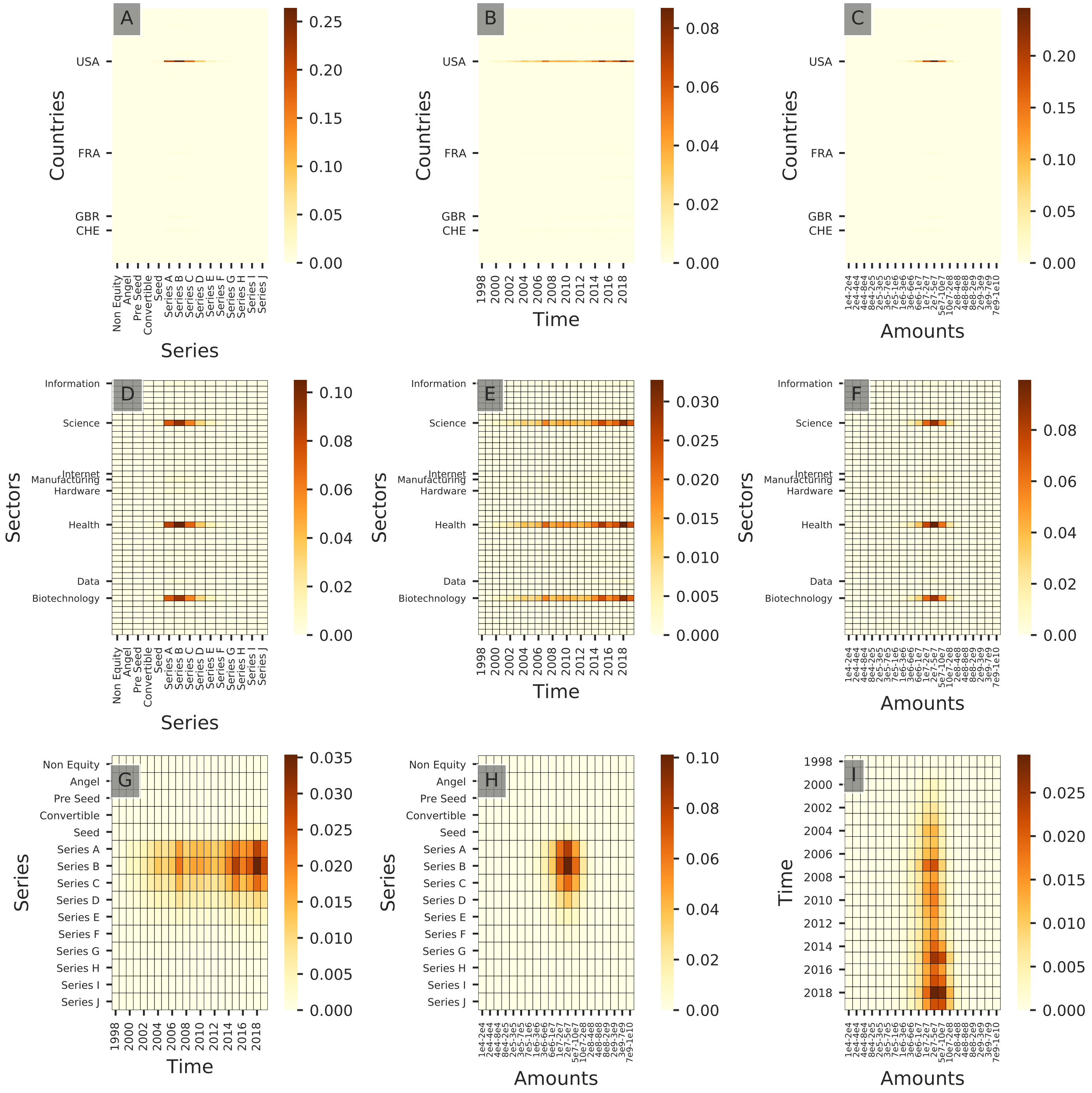

Fig S78. Cross graph interaction heatmap of community A7.

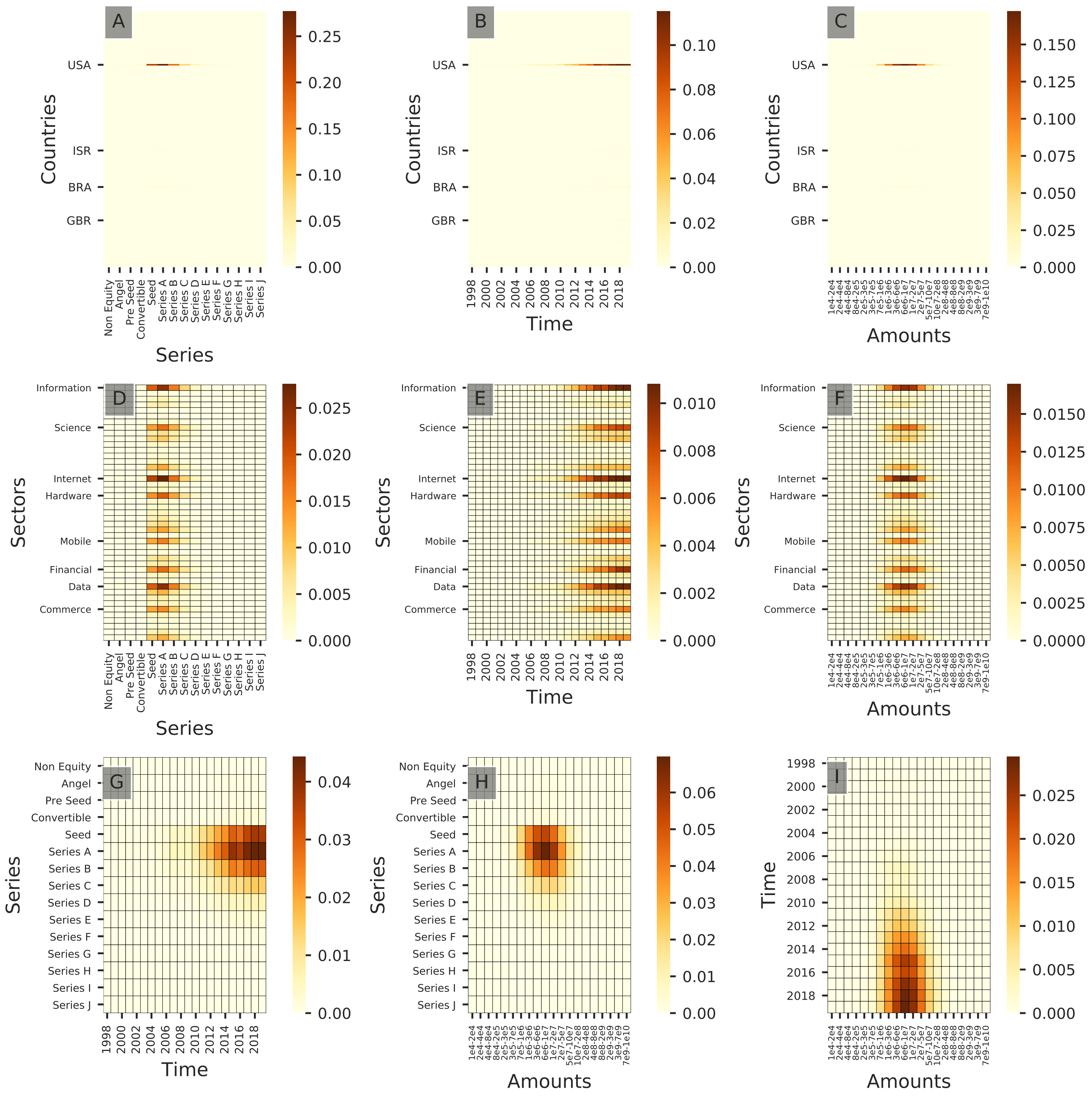

Fig S79. Cross graph interaction heatmap of community A8.

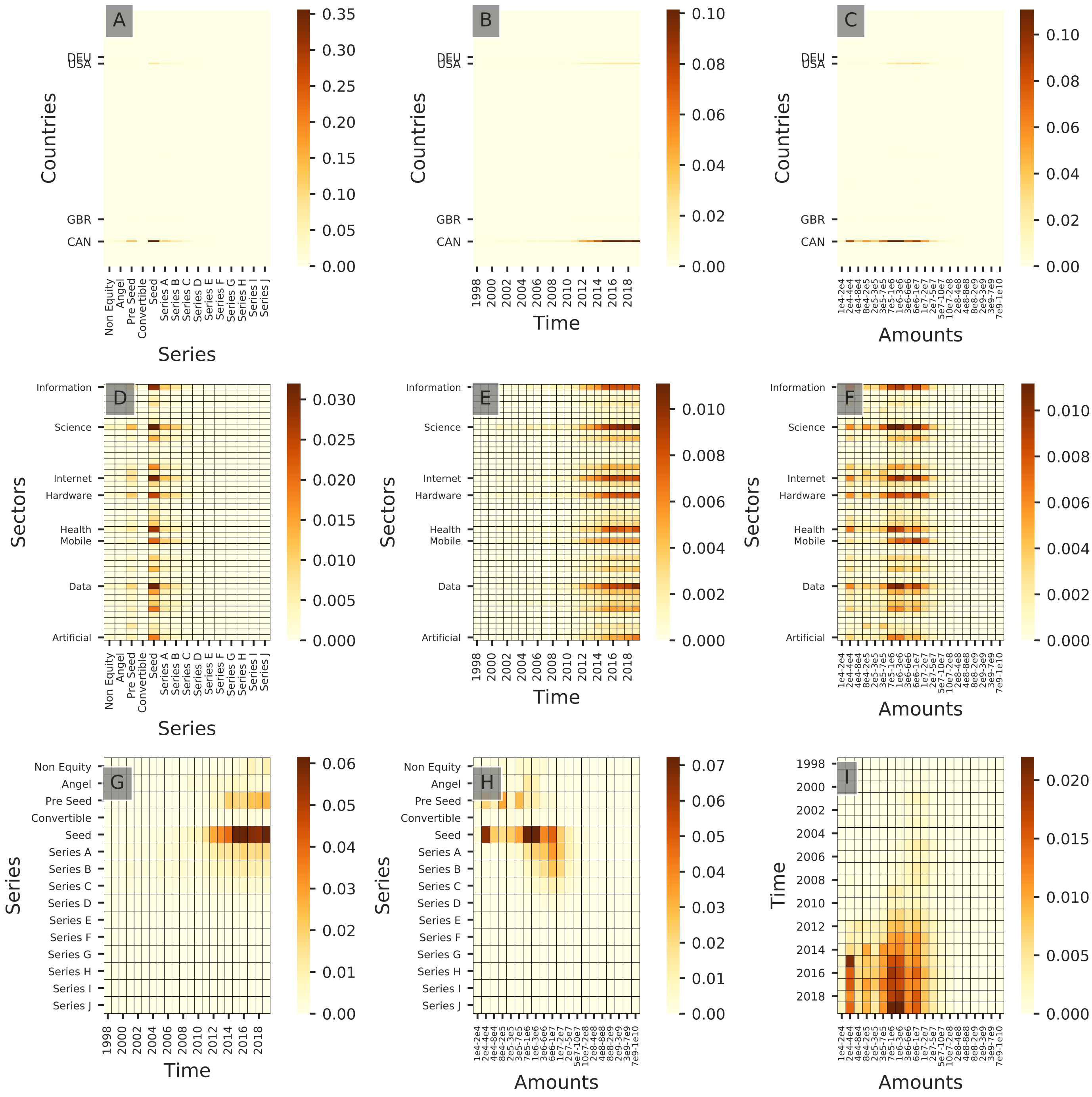

Fig S80. Cross graph interaction heatmap of community A9.

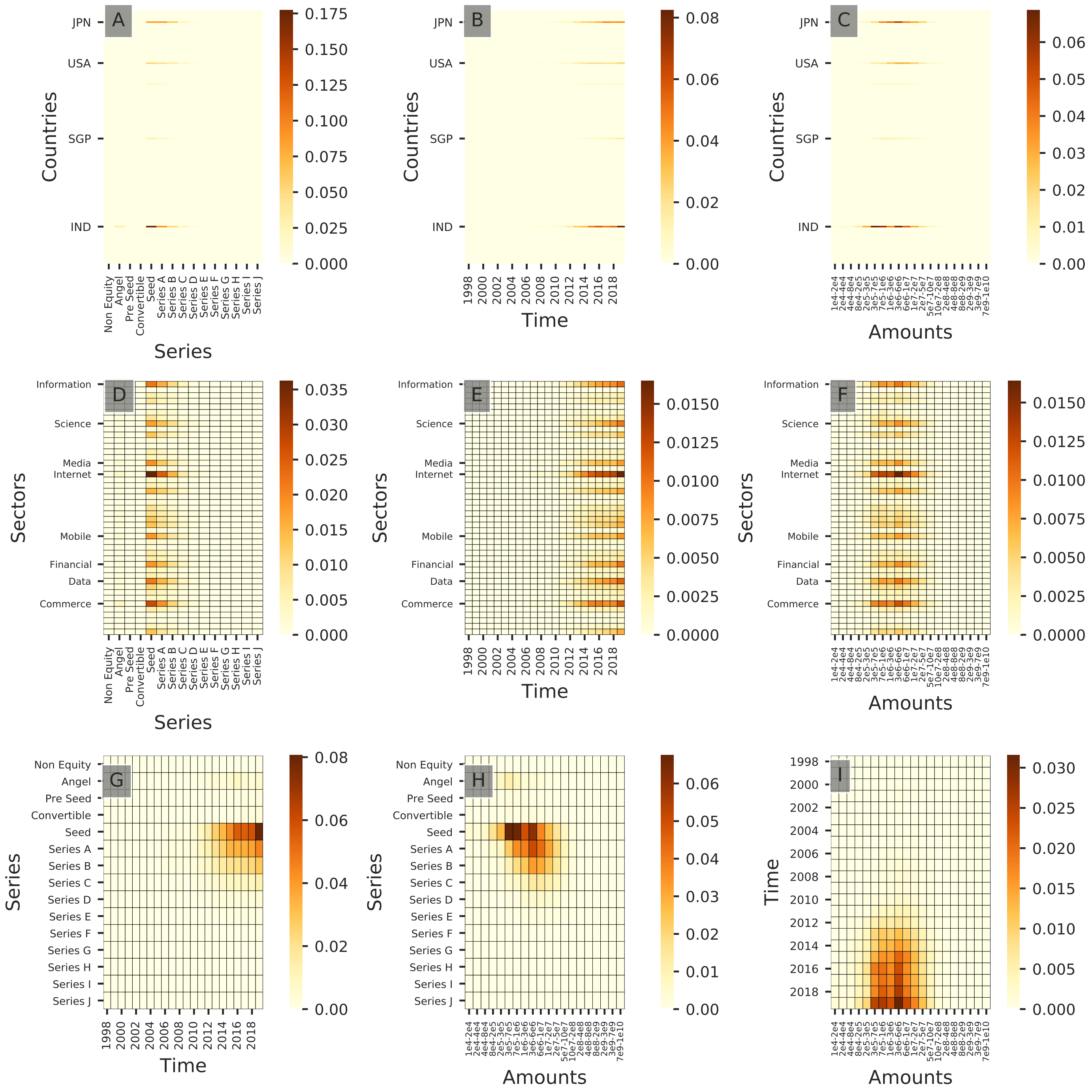

Fig S81. Cross graph interaction heatmap of community A10.

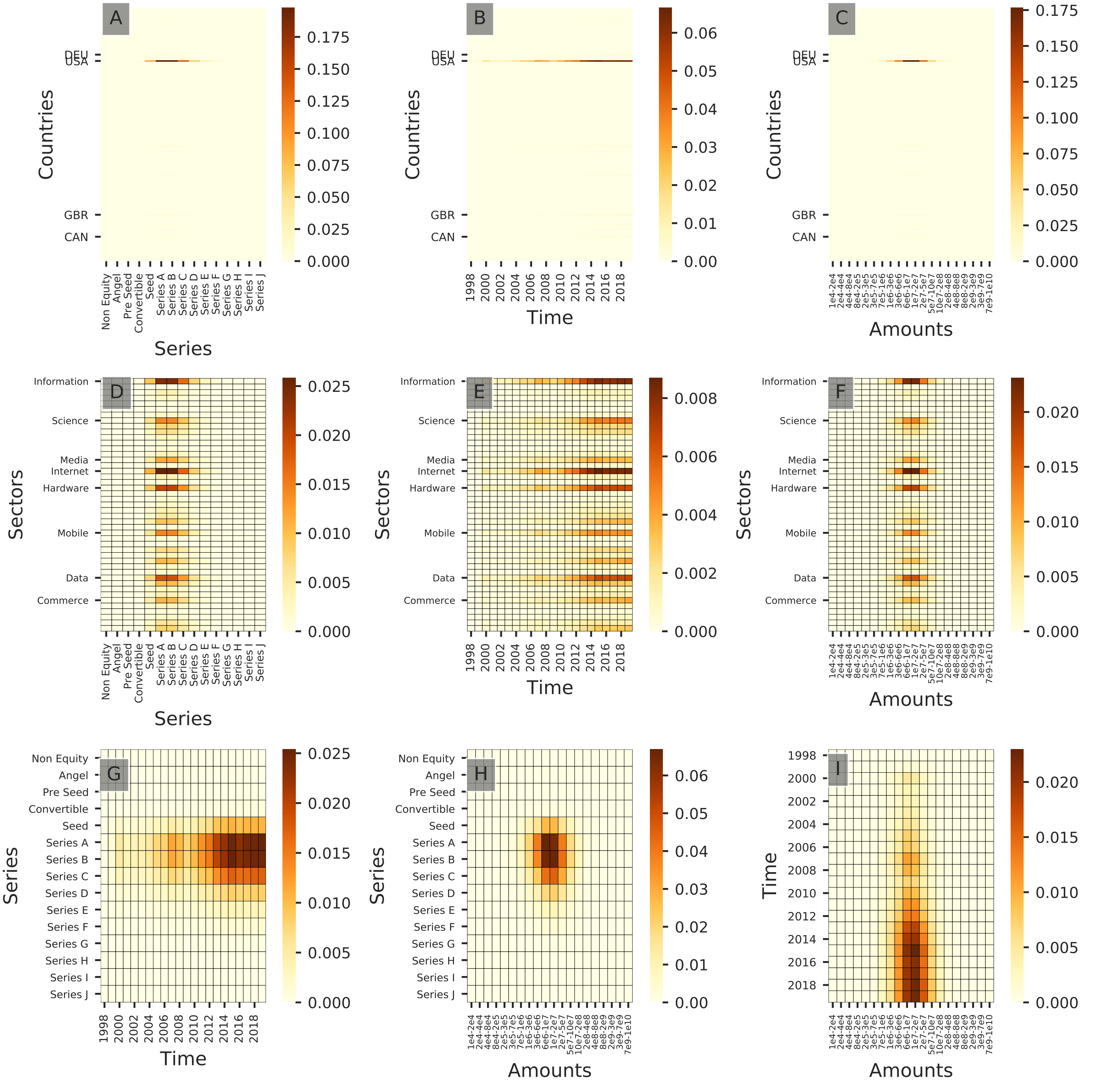

Fig S82. Cross graph interaction heatmap of community B0.

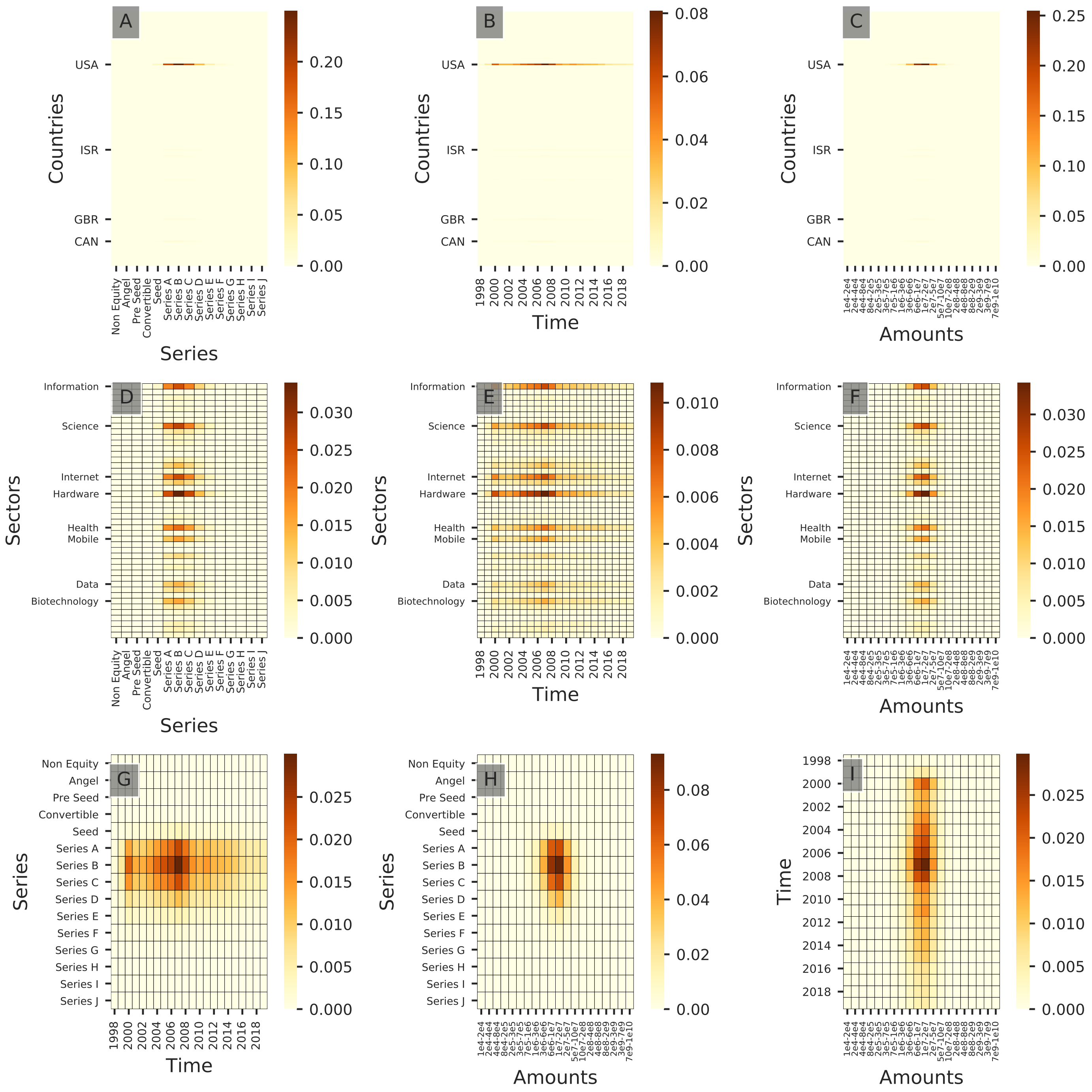

Fig S83. Cross graph interaction heatmap of community B1.

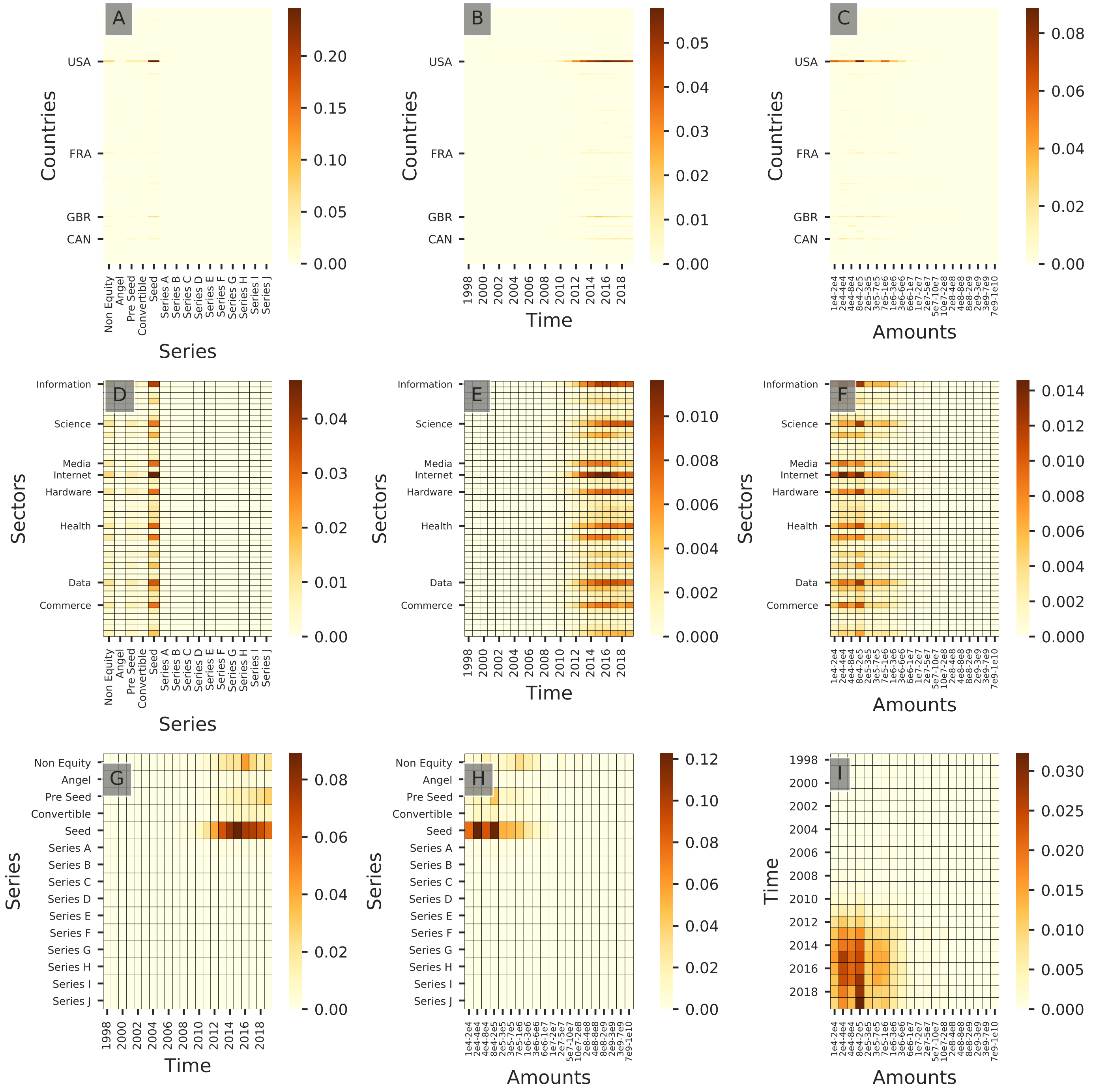

Fig S84. Cross graph interaction heatmap of community B2.

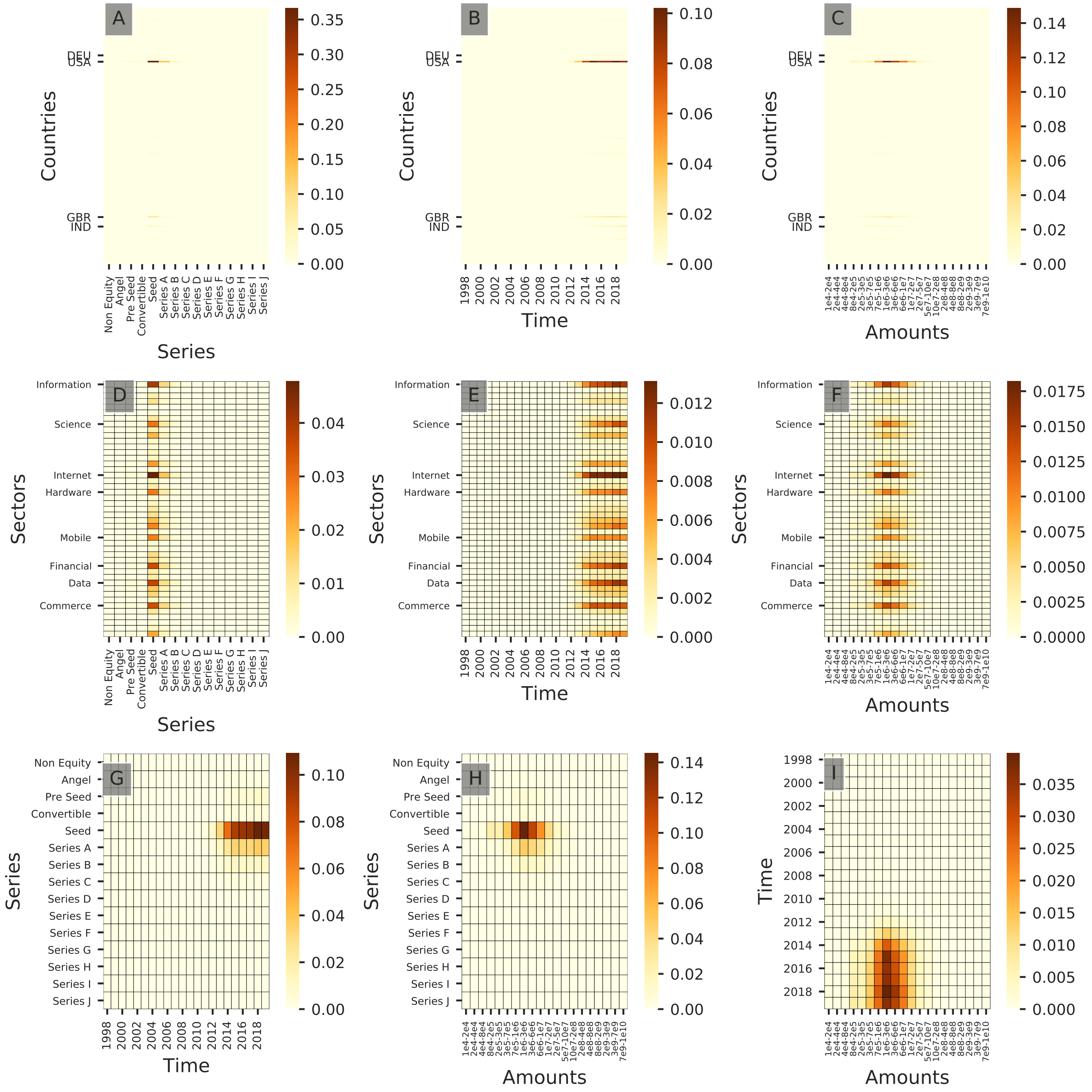

Fig S85. Cross graph interaction heatmap of community B3.

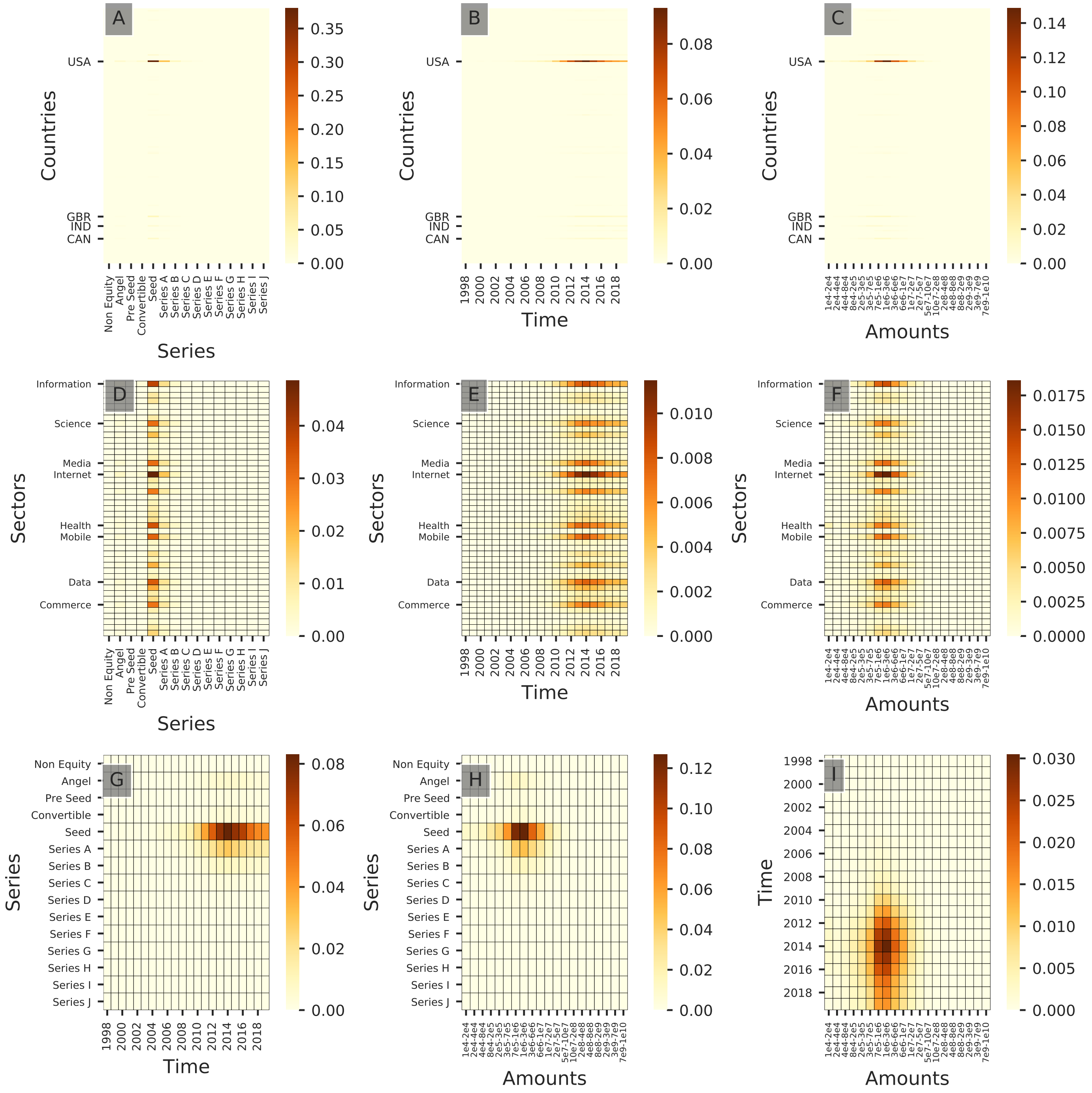

Fig S86. Cross graph interaction heatmap of community B4.

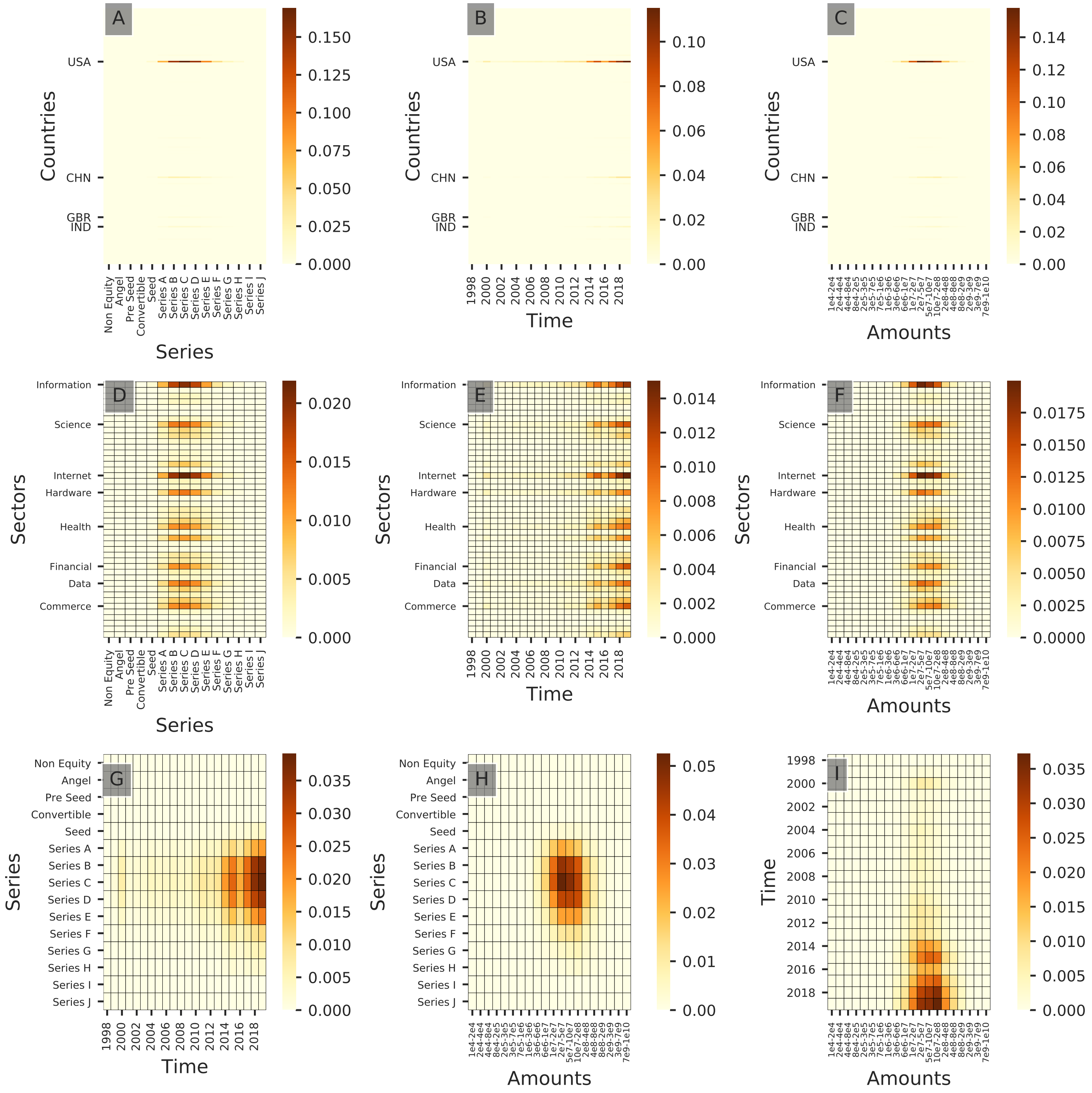

Fig S87. Cross graph interaction heatmap of community B5.

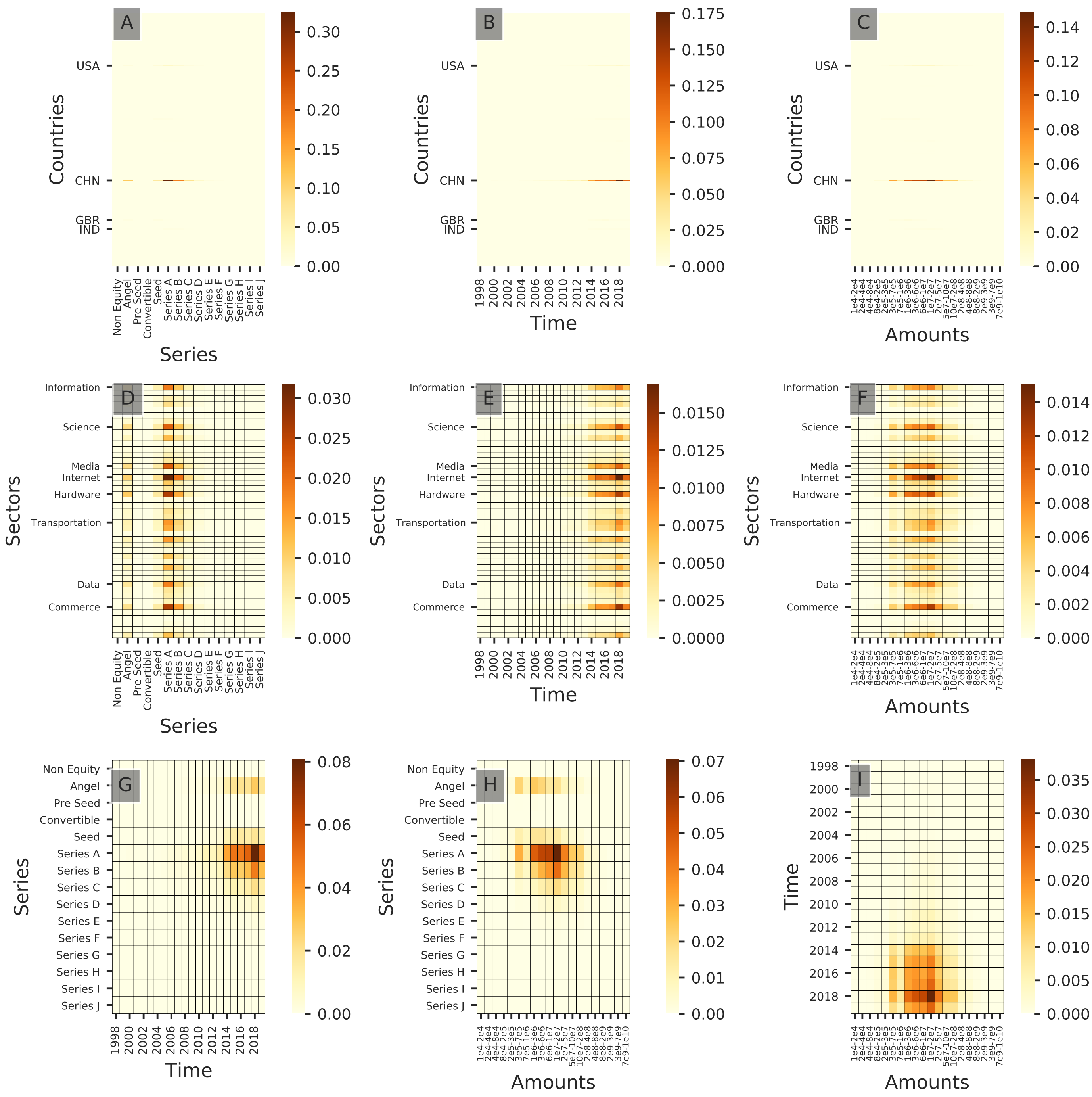

Fig S88. Cross graph interaction heatmap of community B6.

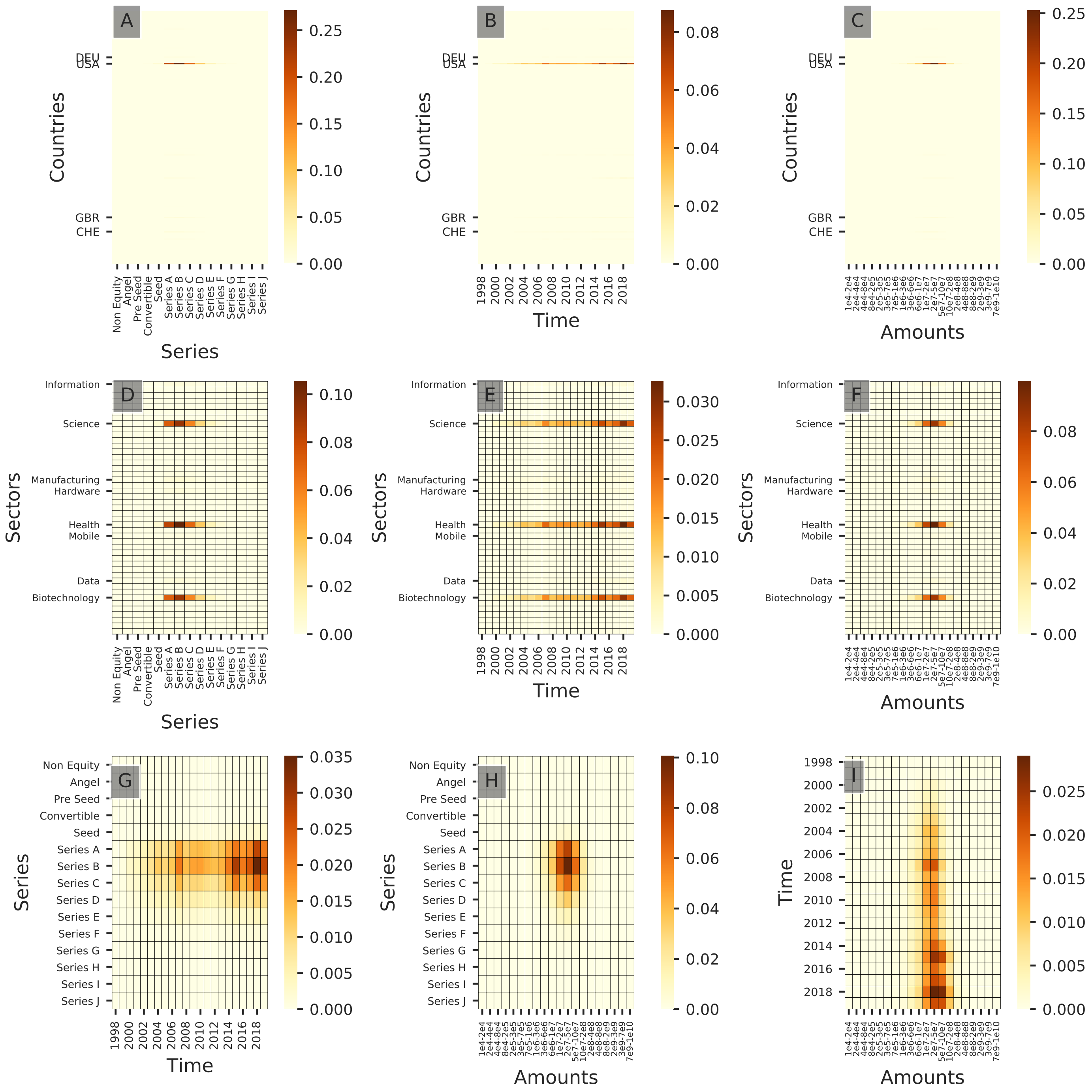

Fig S89. Cross graph interaction heatmap of community B7.

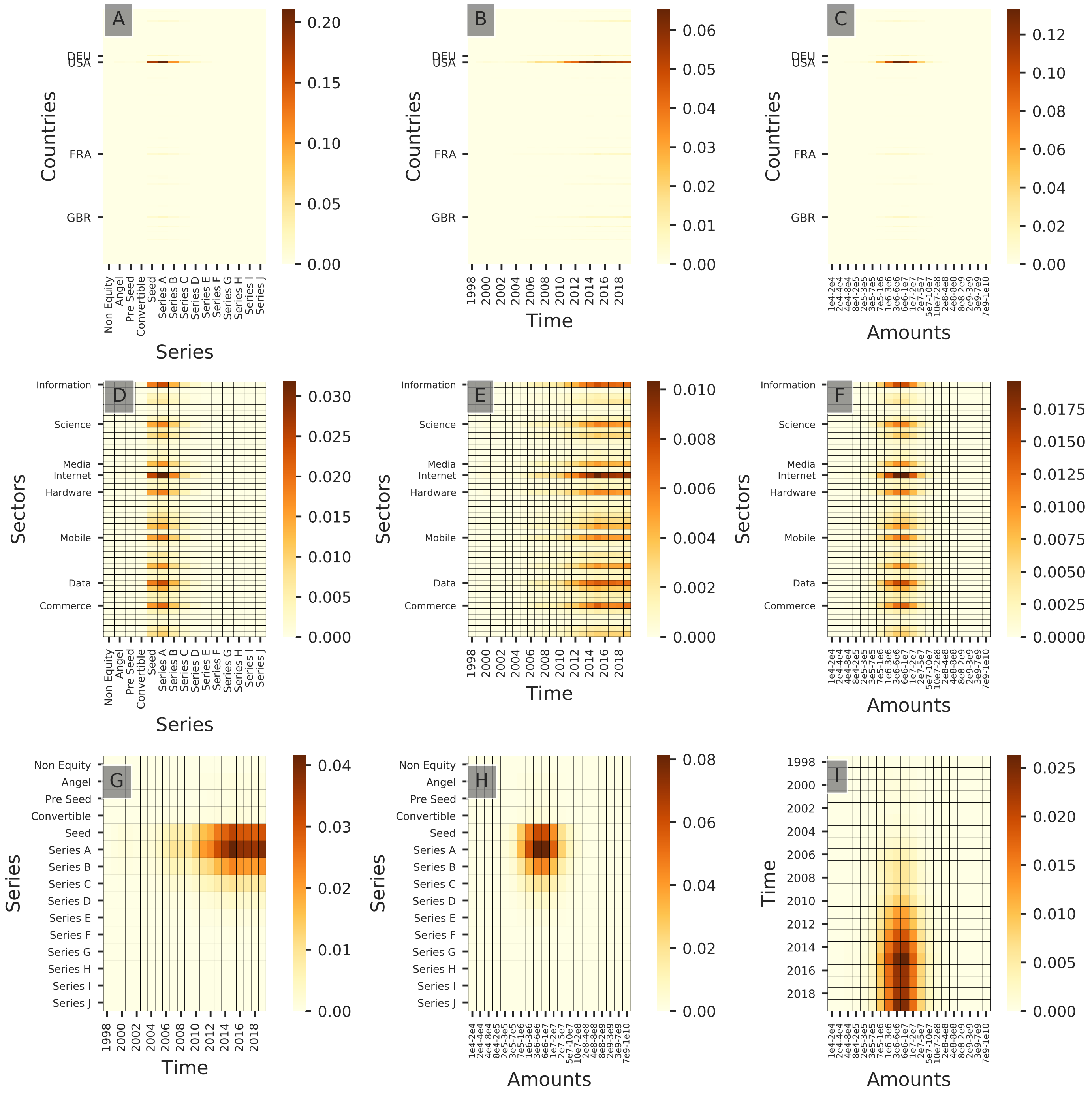

Fig S90. Cross graph interaction heatmap of community B8.

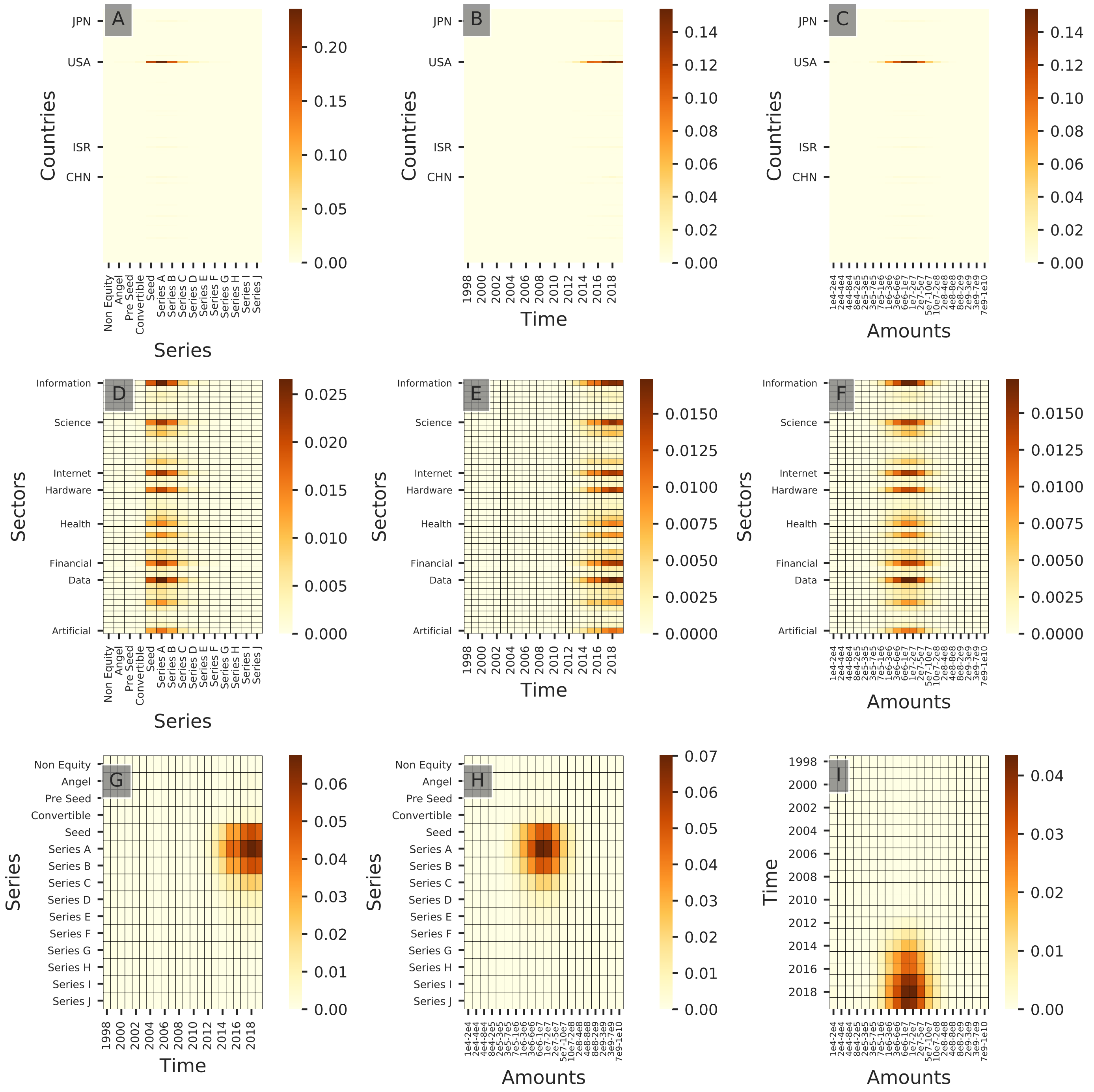

Fig S91. Cross graph interaction heatmap of community B9.

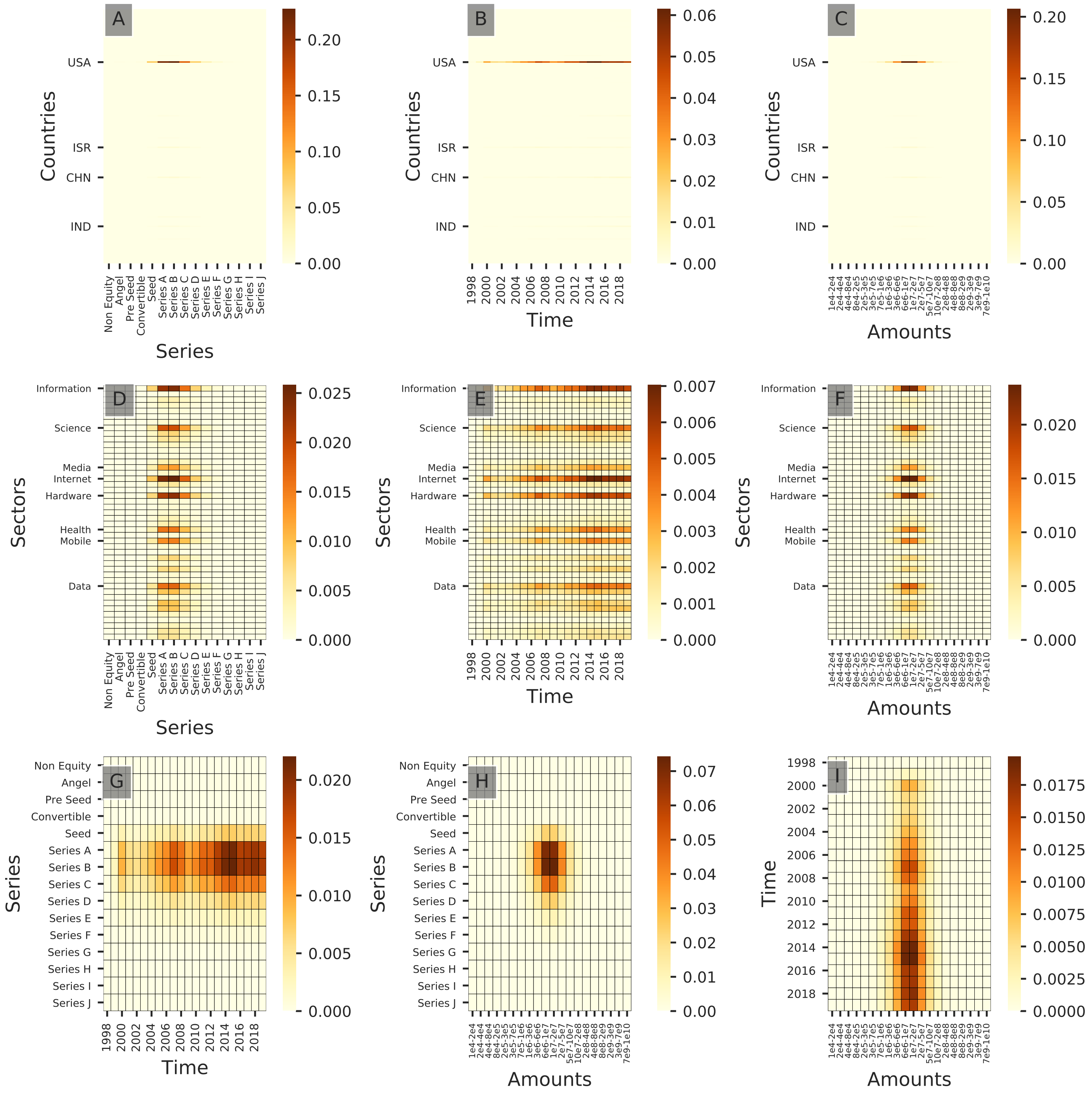

Fig S92. Cross graph interaction heatmap of community C0.

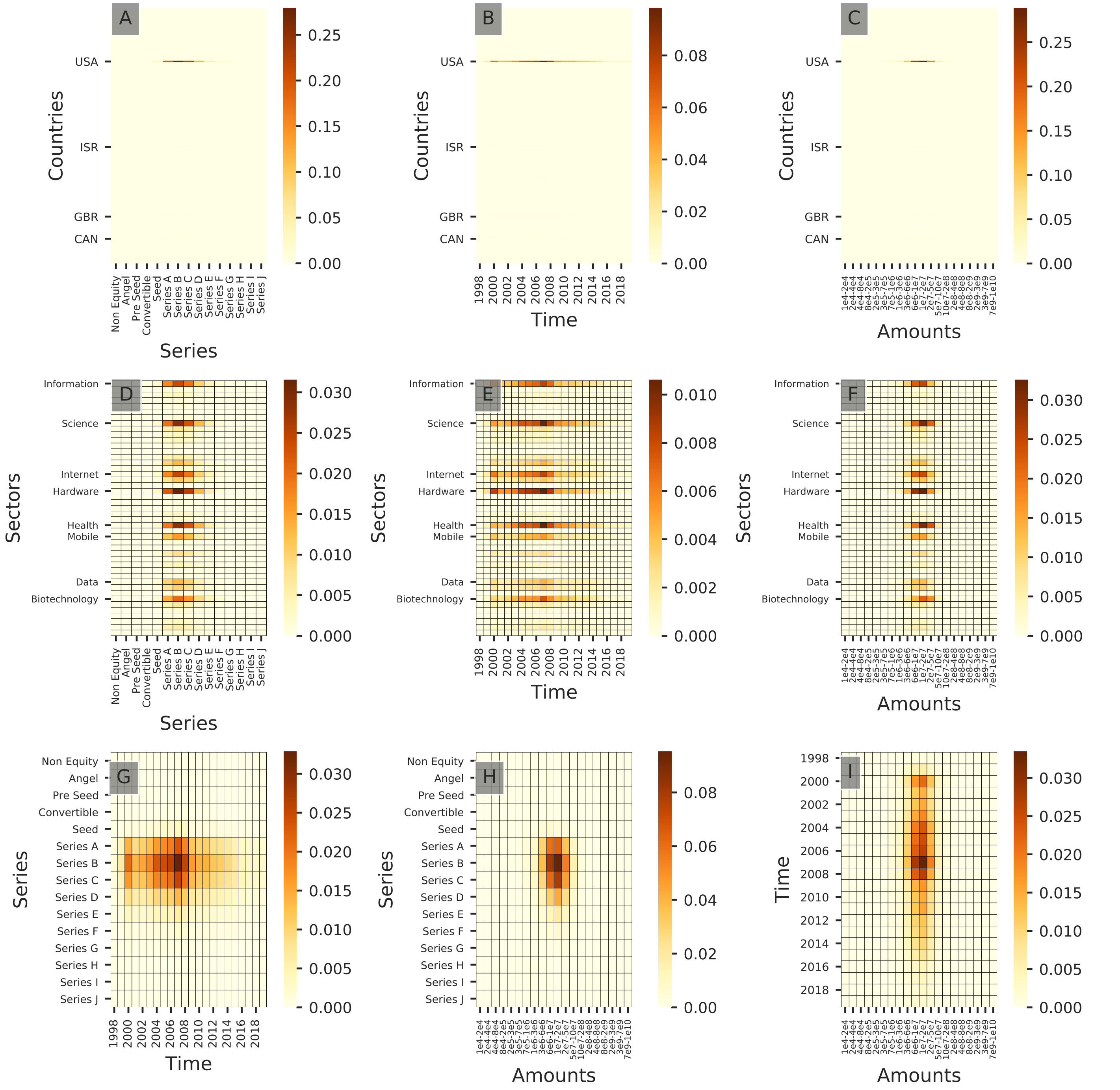

Fig S93. Cross graph interaction heatmap of community C1.

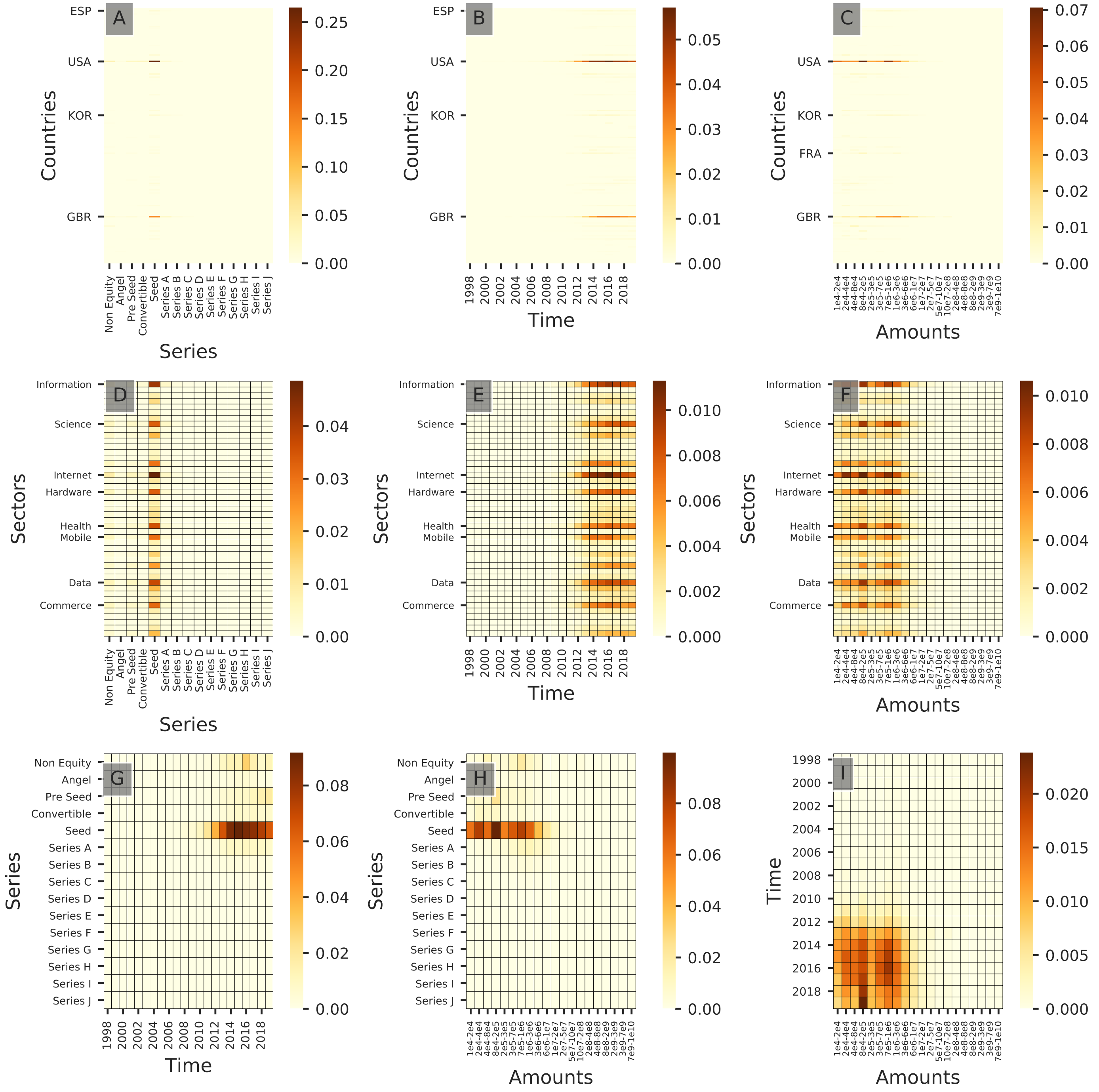

Fig S94. Cross graph interaction heatmap of community C2.

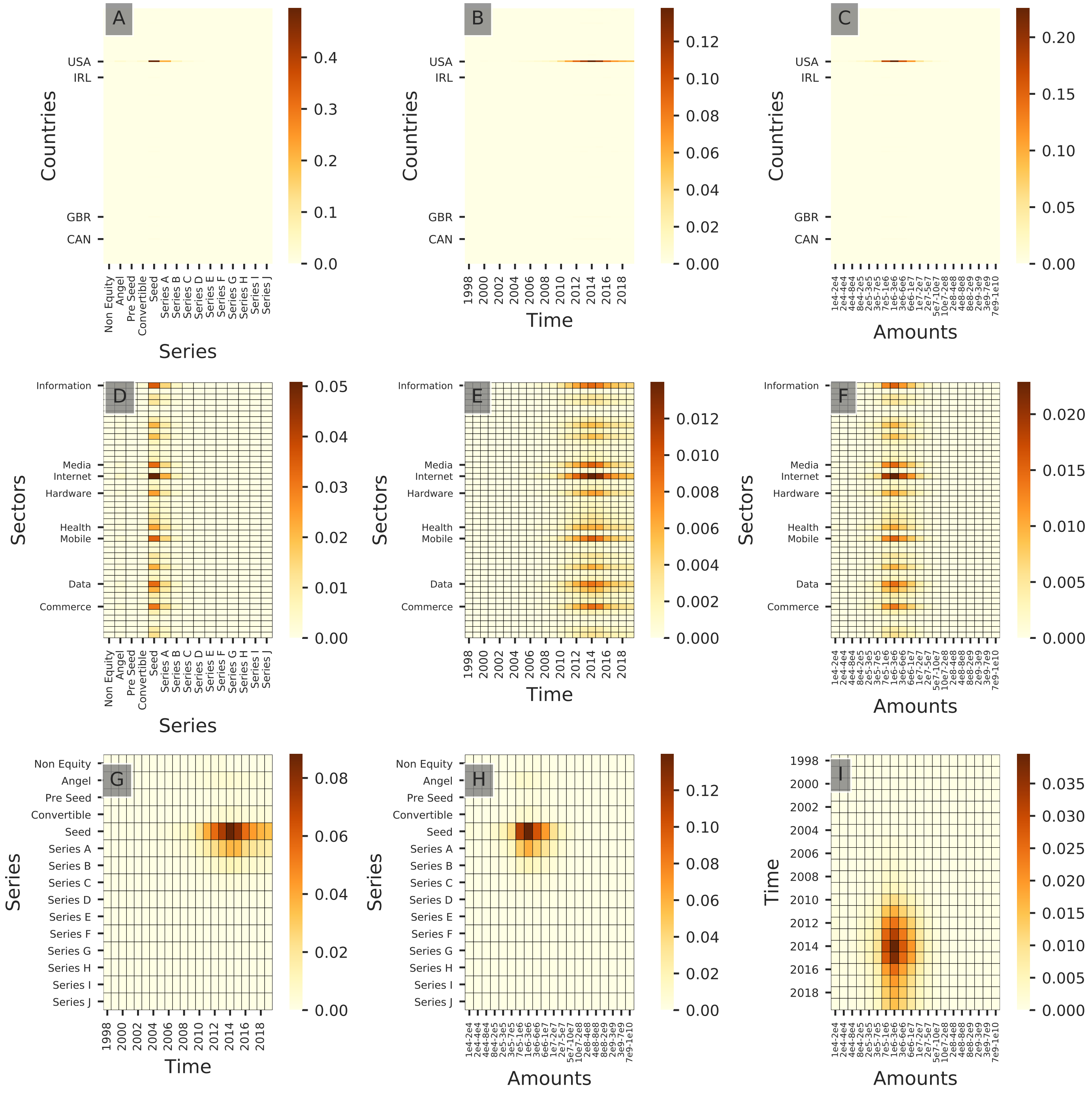

Fig S95. Cross graph interaction heatmap of community C3.

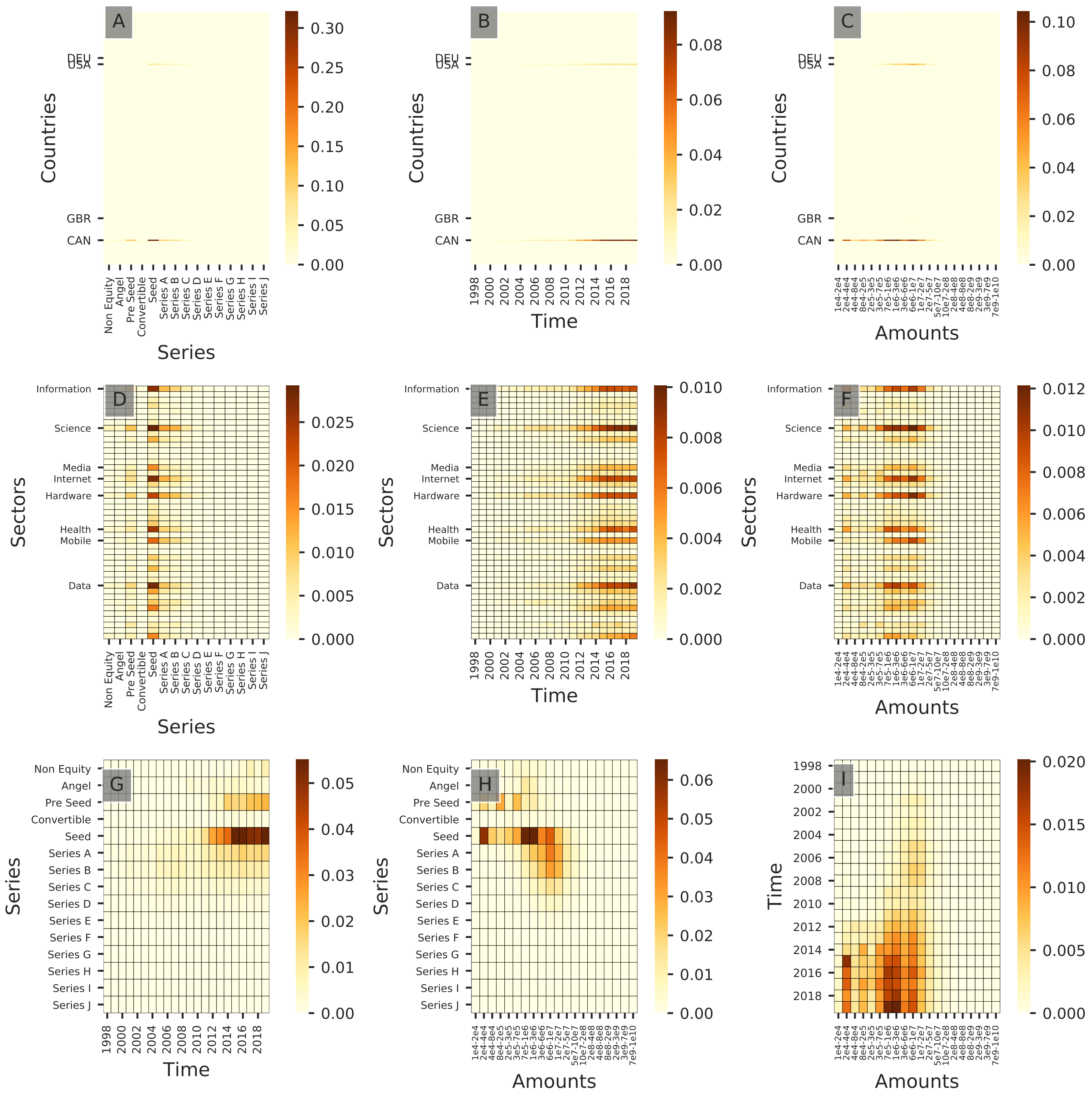

Fig S96. Cross graph interaction heatmap of community C4.

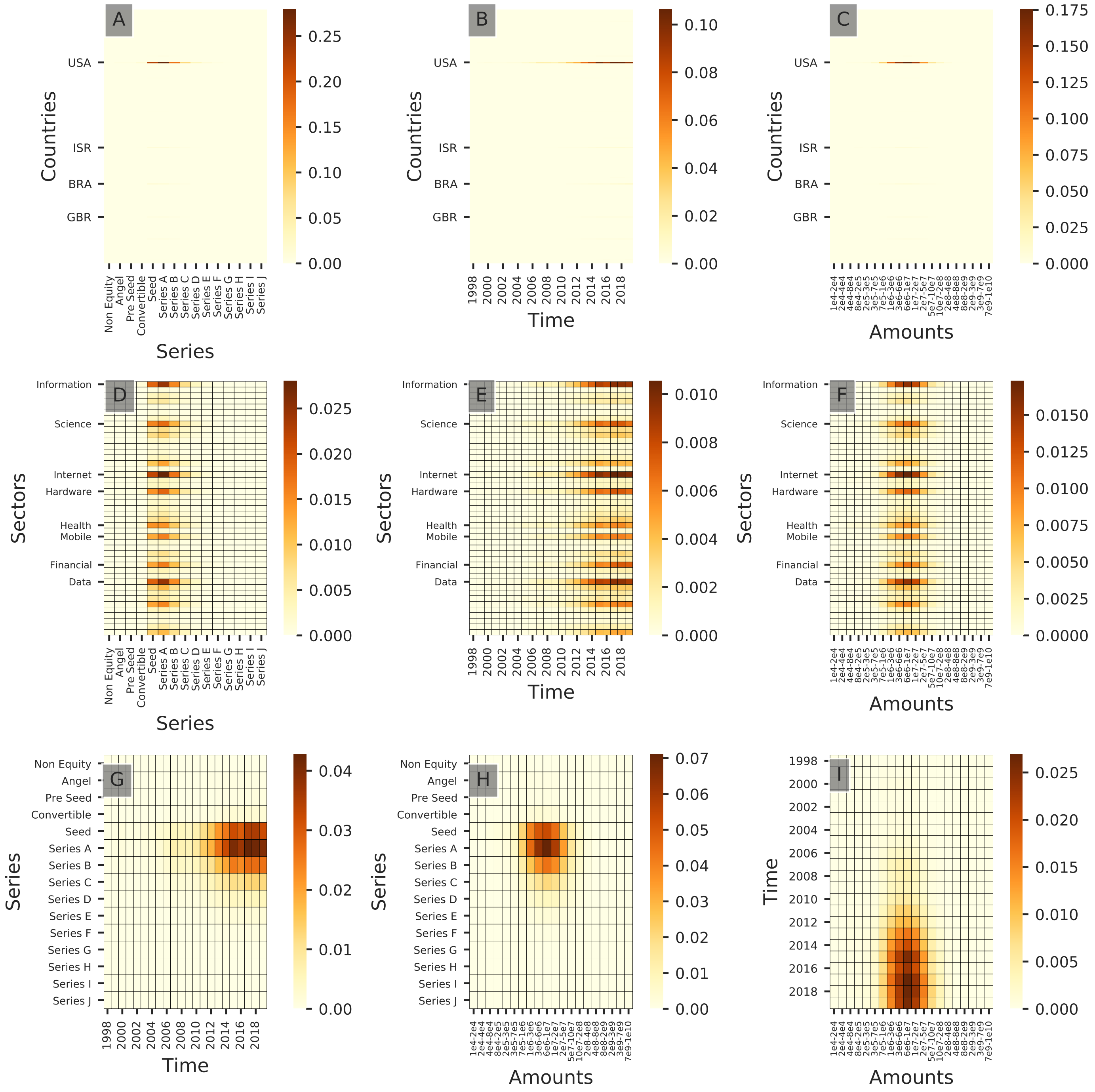

Fig S97. Cross graph interaction heatmap of community C5.

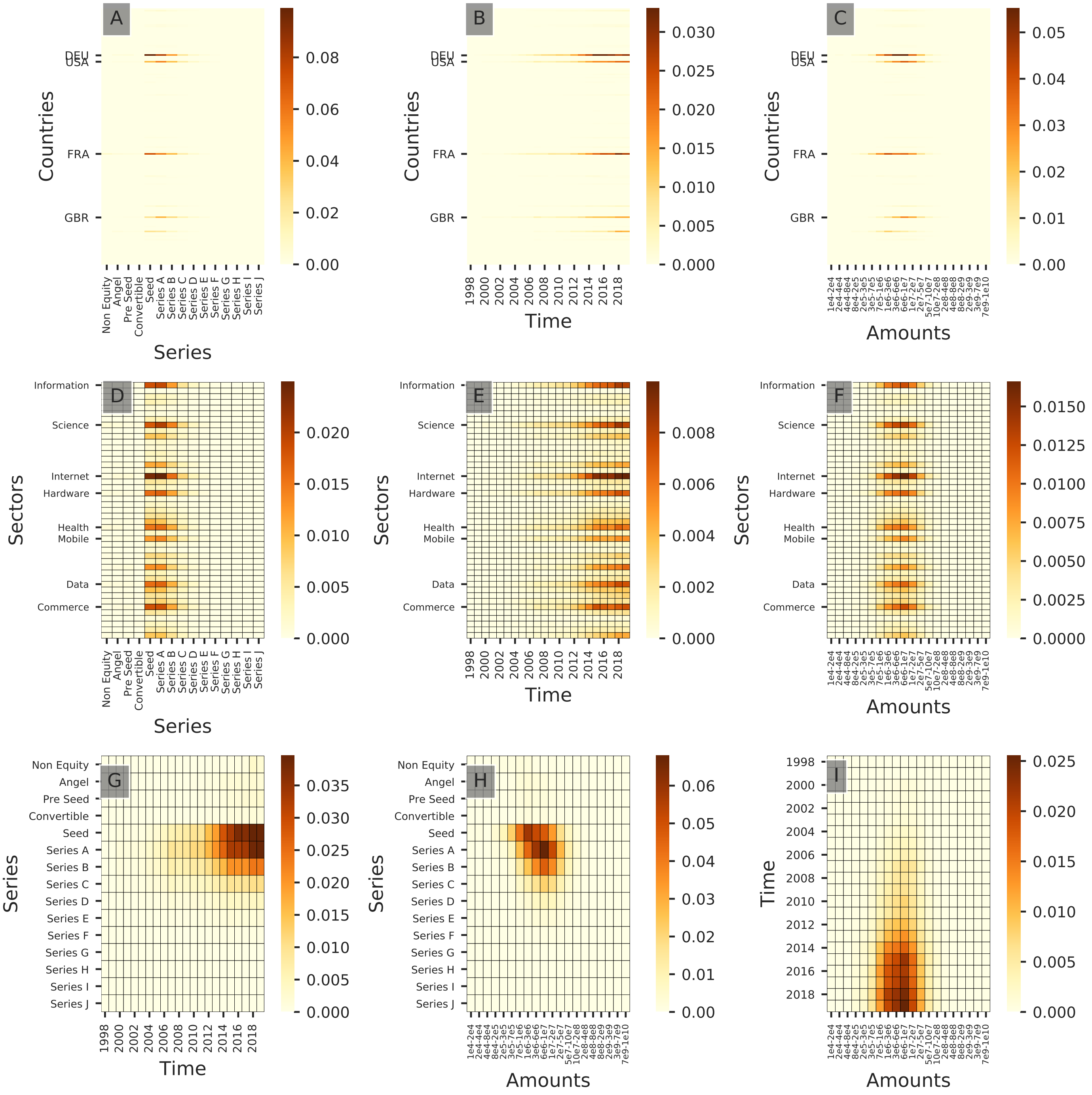

Fig S98. Cross graph interaction heatmap of community C6.

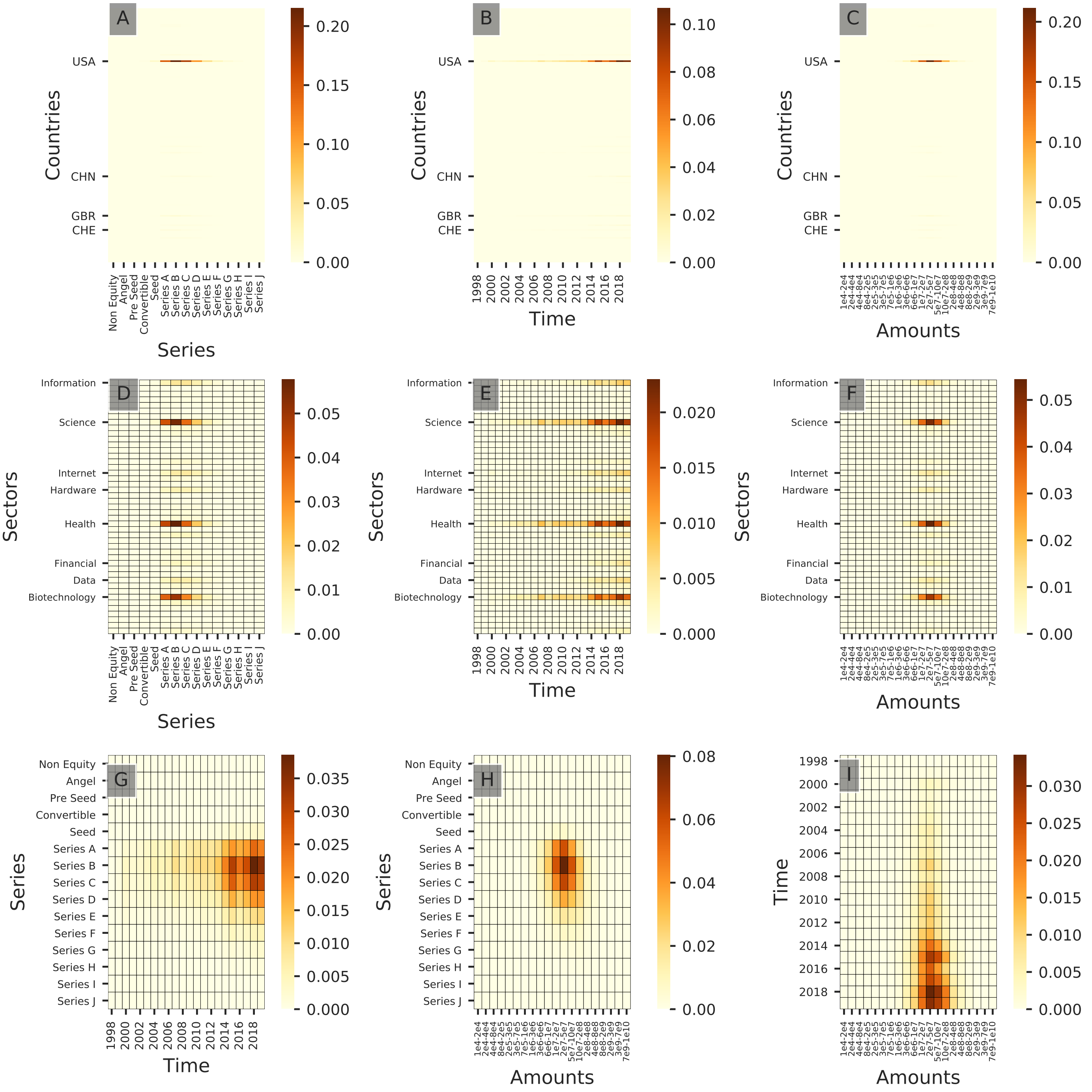

Fig S99. Cross graph interaction heatmap of community C7.

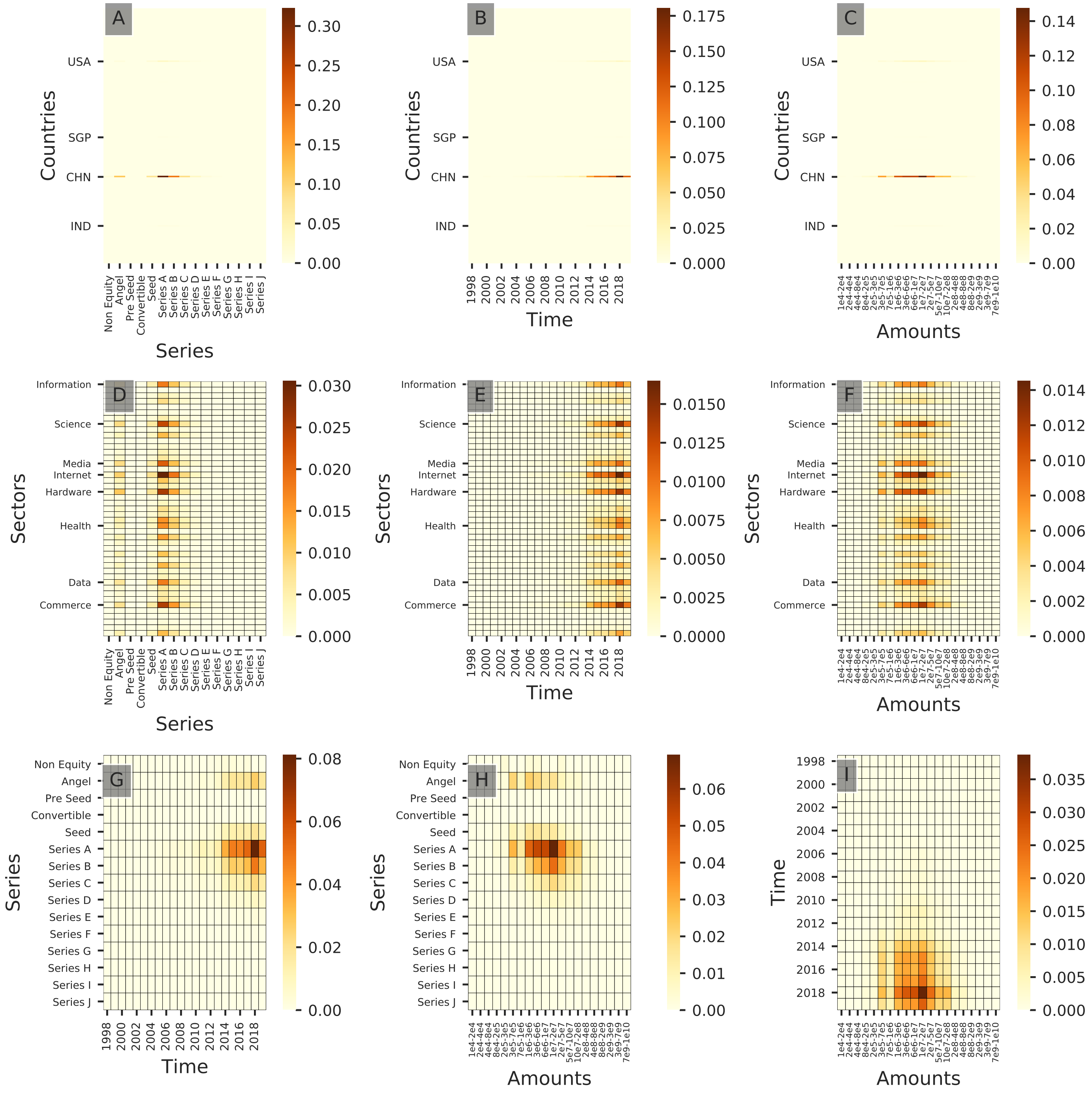

Fig S100. Cross graph interaction heatmap of community C8.

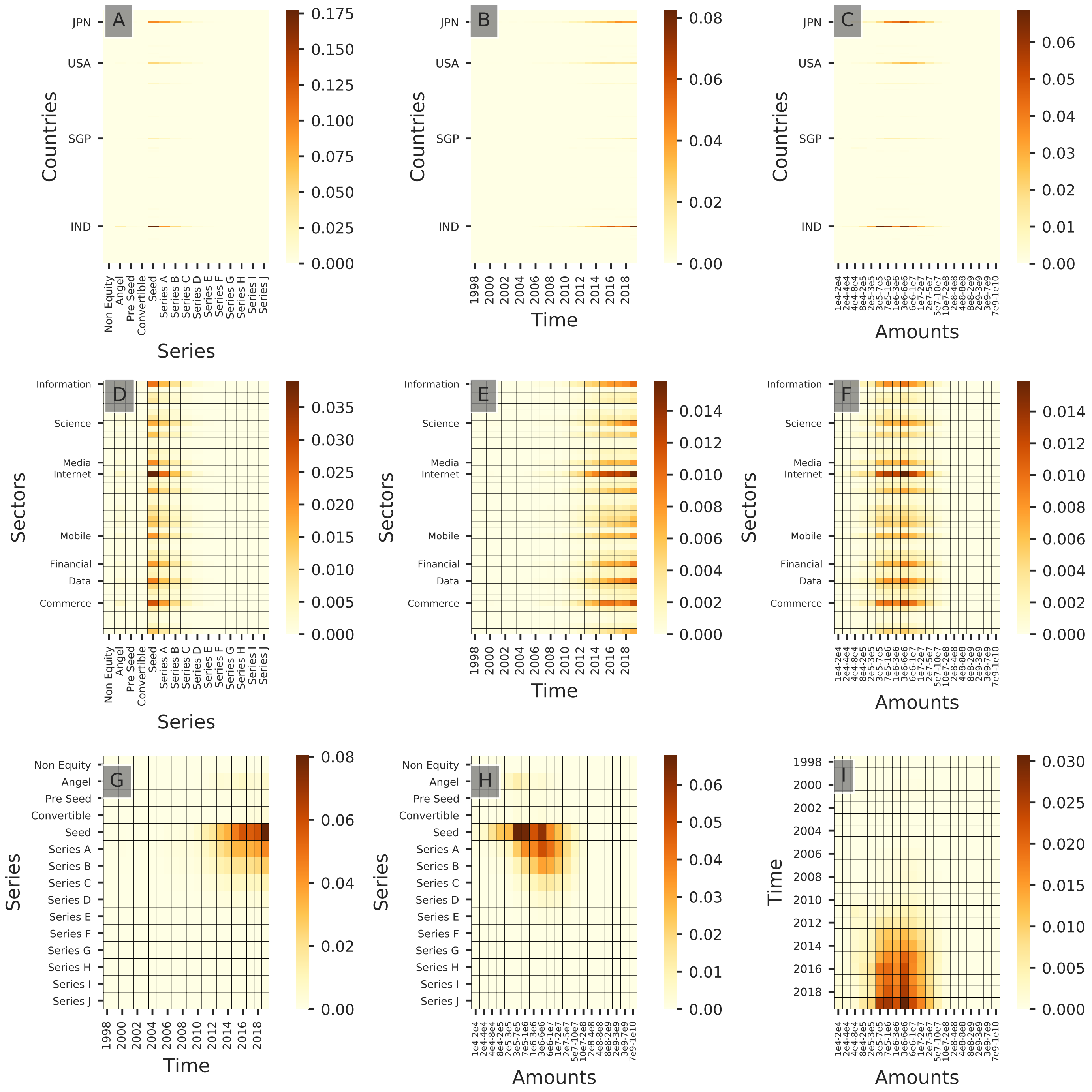

Fig S101. Cross graph interaction heatmap of community C9.

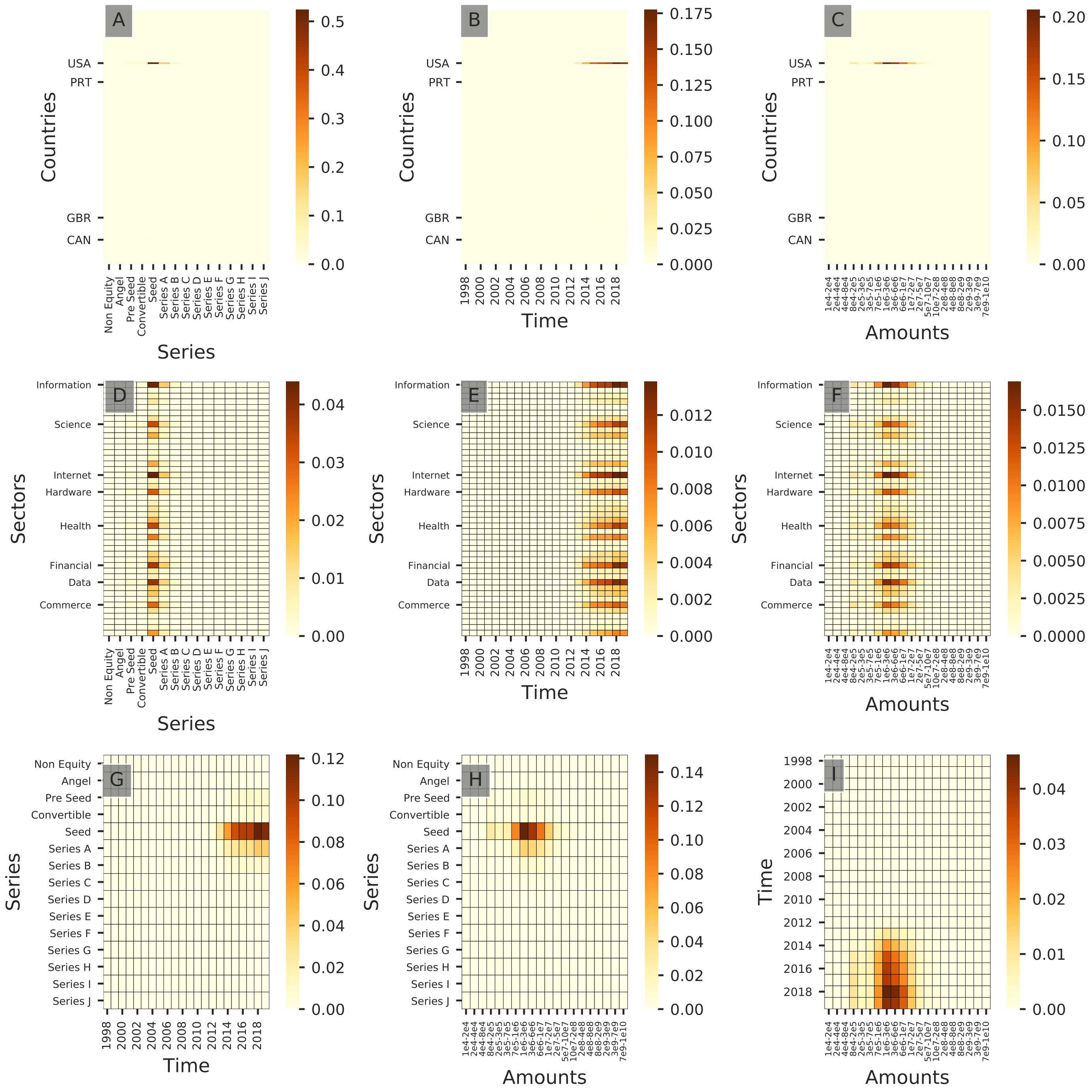

Fig S102. Cross graph interaction heatmap of community C10.

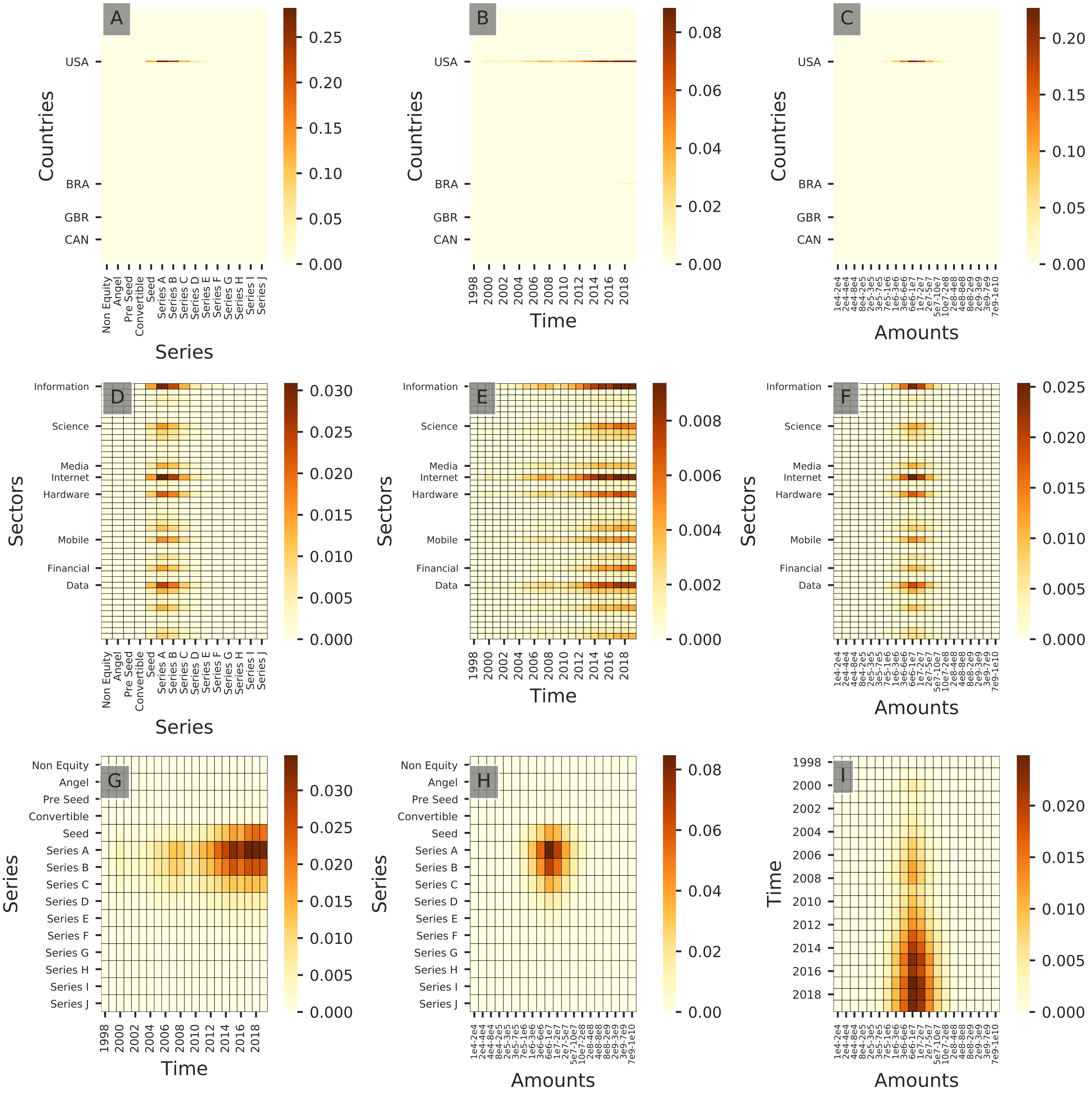

Fig S103. Cross graph interaction heatmap of community D0.

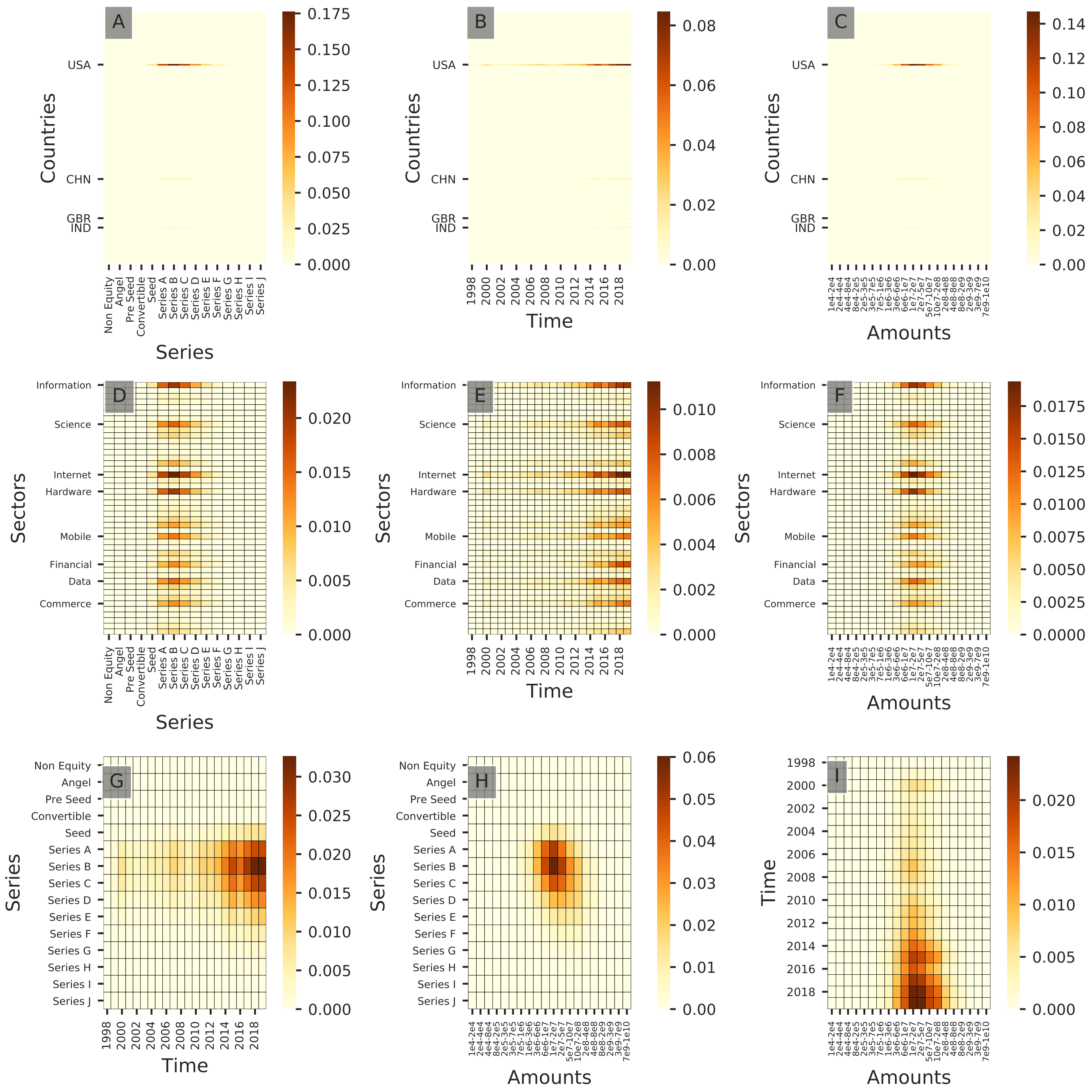

Fig S104. Cross graph interaction heatmap of community D1.

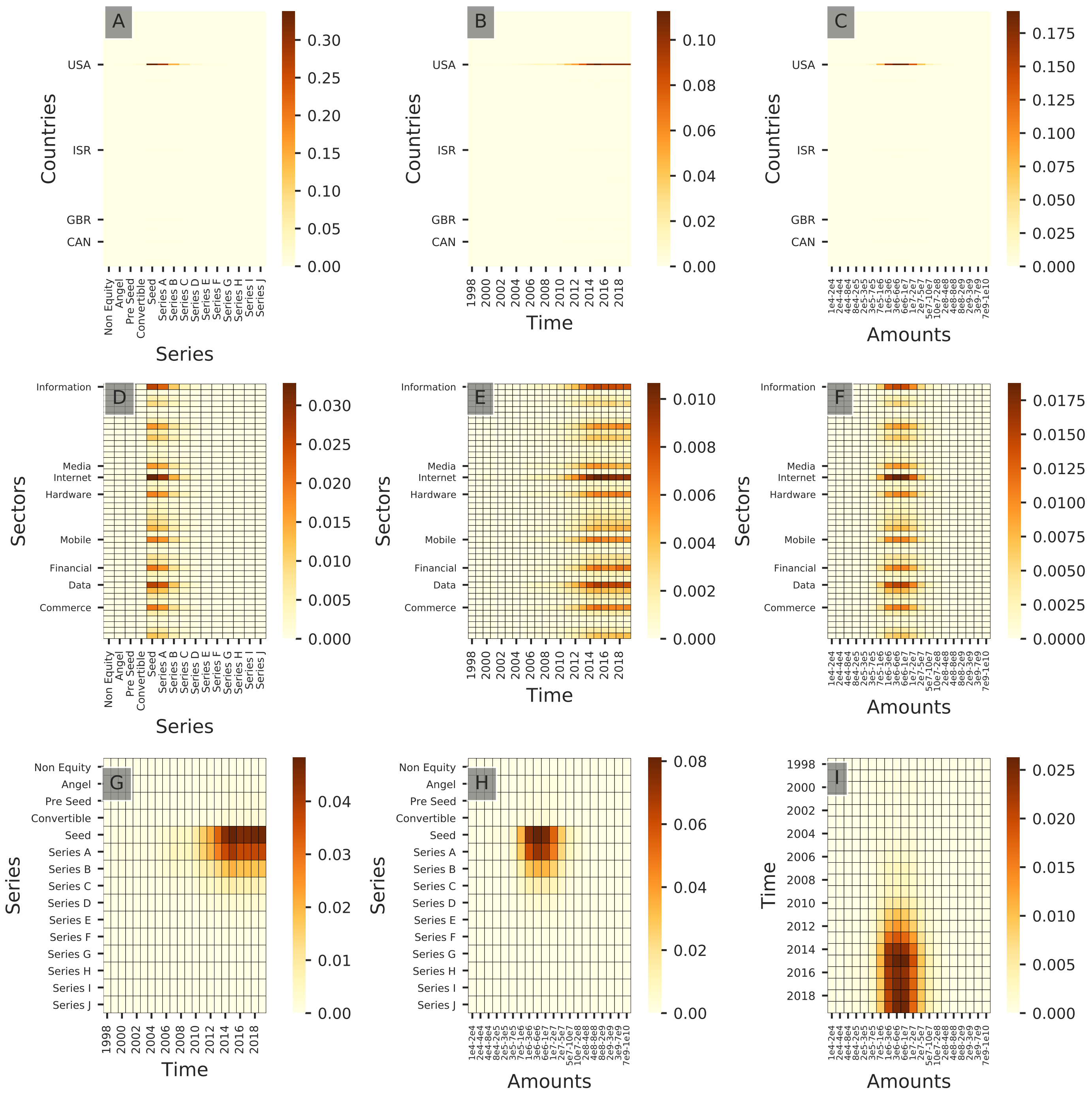

Fig S105. Cross graph interaction heatmap of community D2.

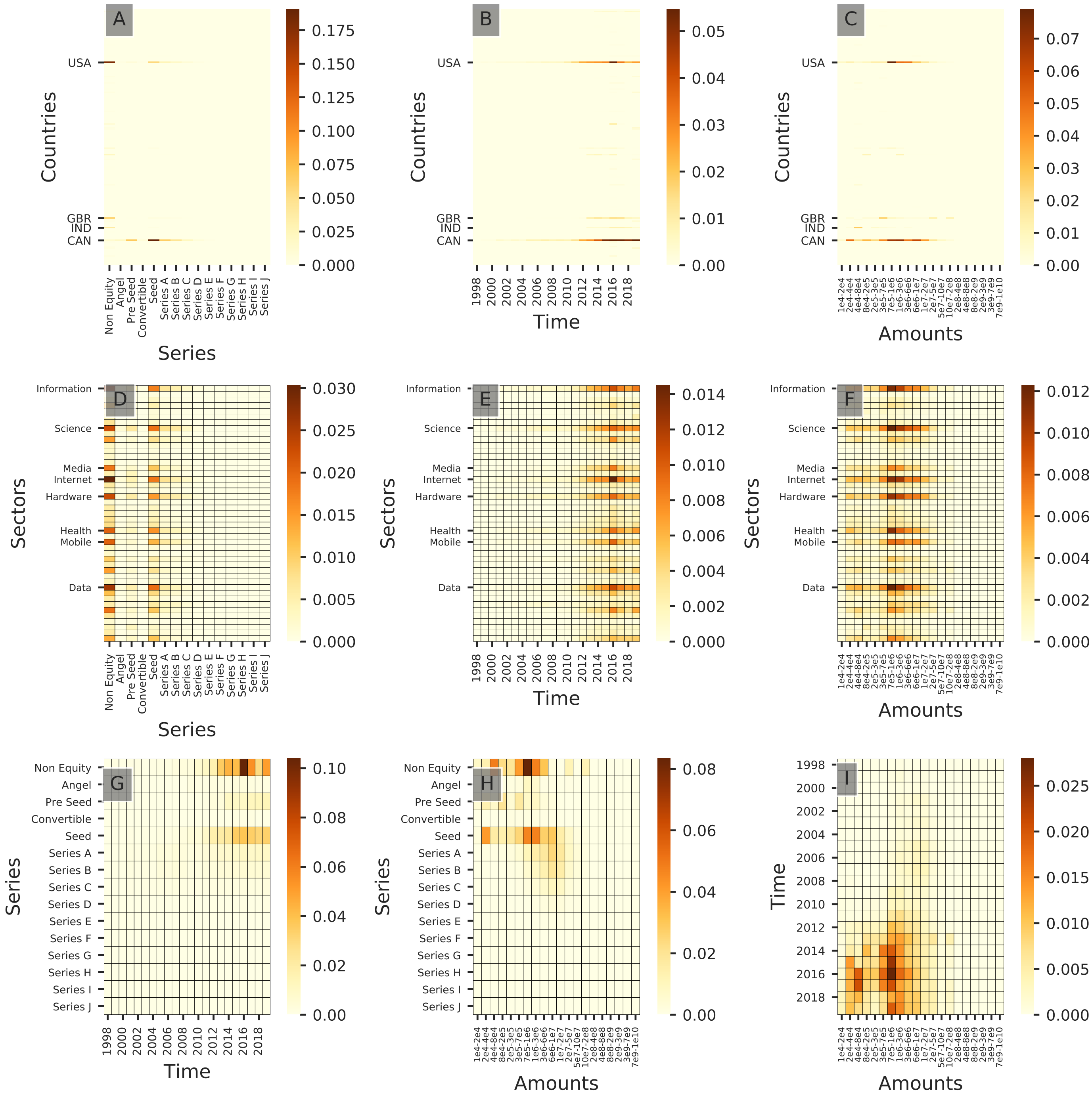

Fig S106. Cross graph interaction heatmap of community D3.

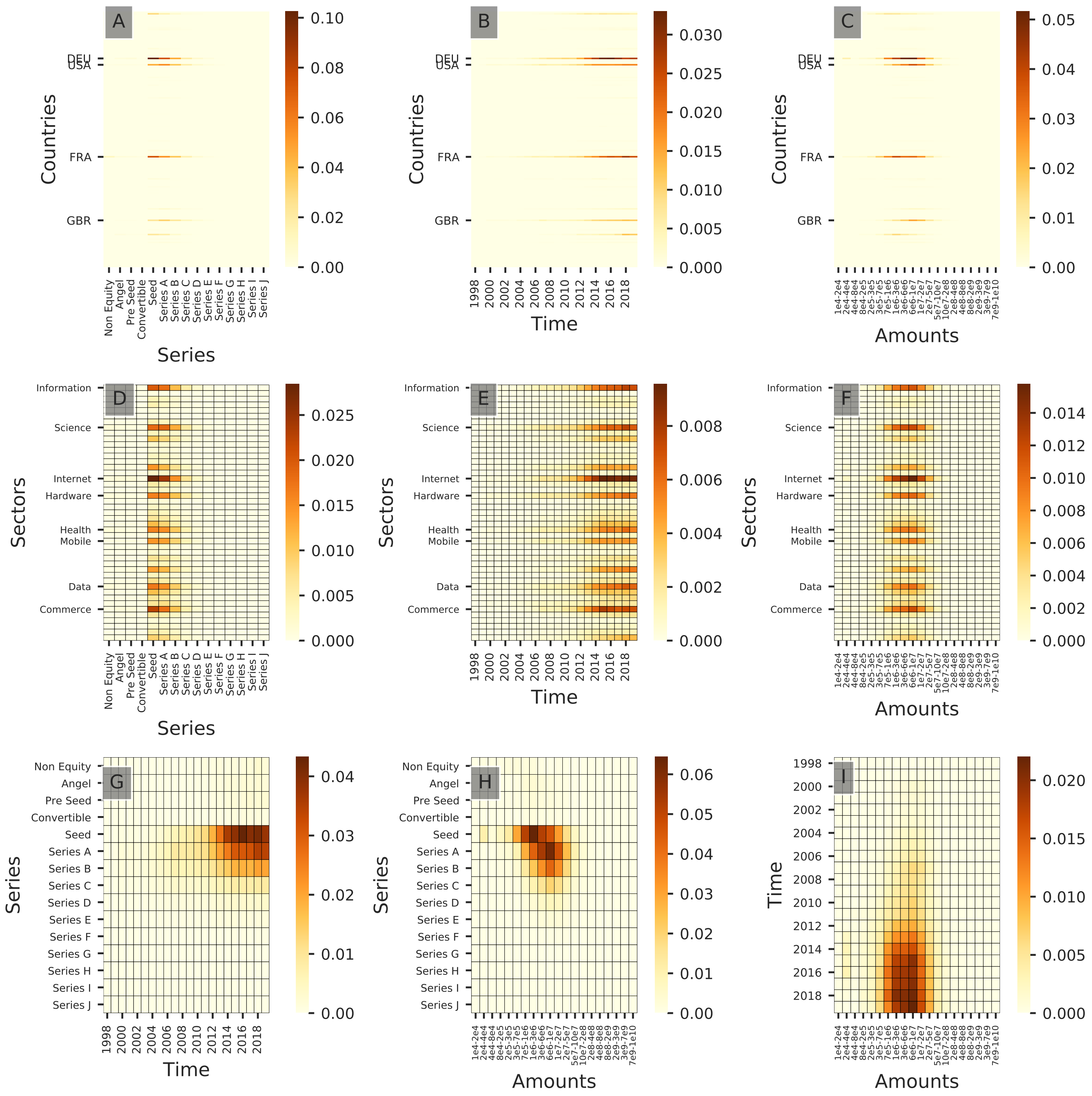

Fig S107. Cross graph interaction heatmap of community D4.

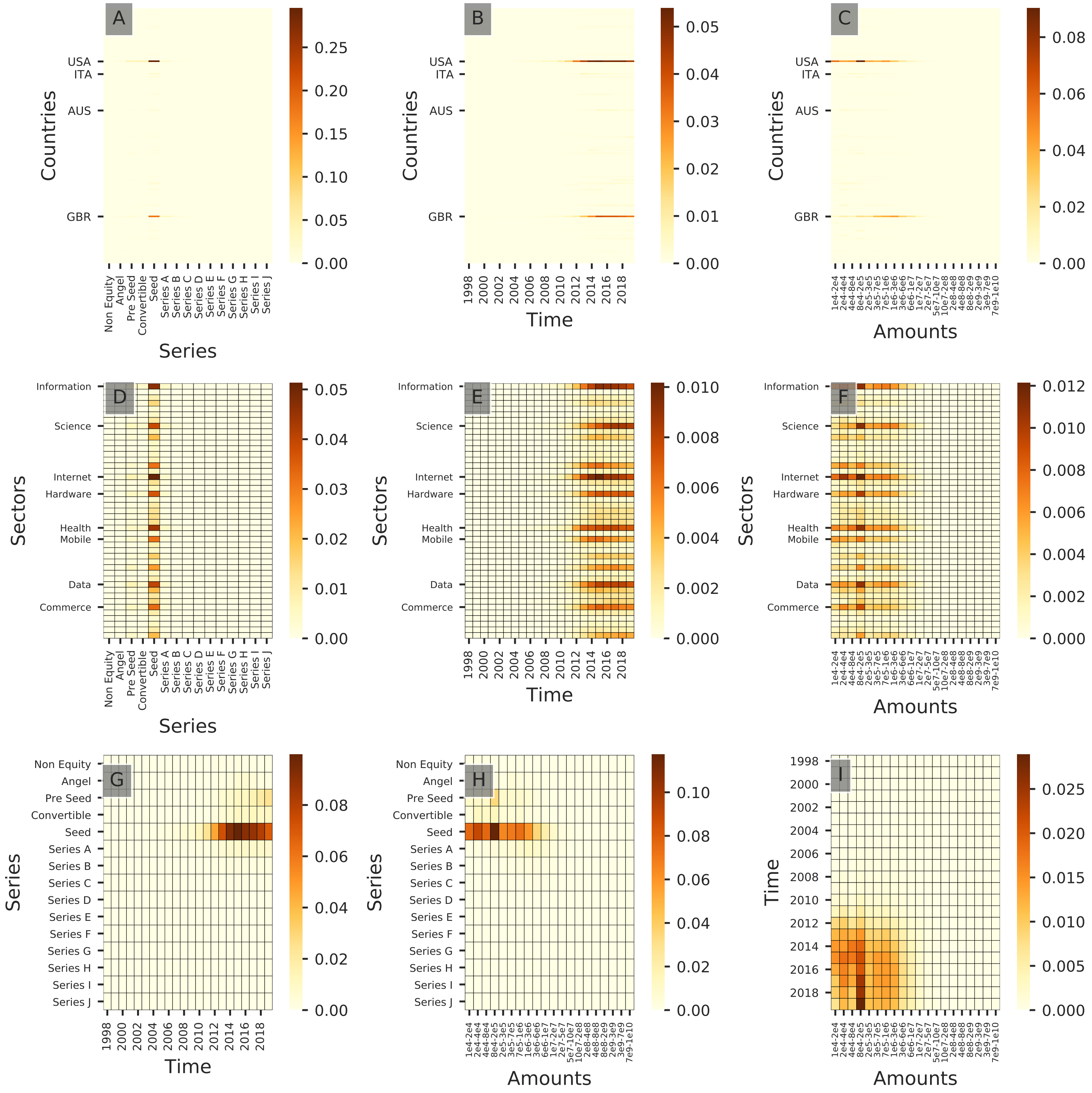

Fig S108. Cross graph interaction heatmap of community D5.

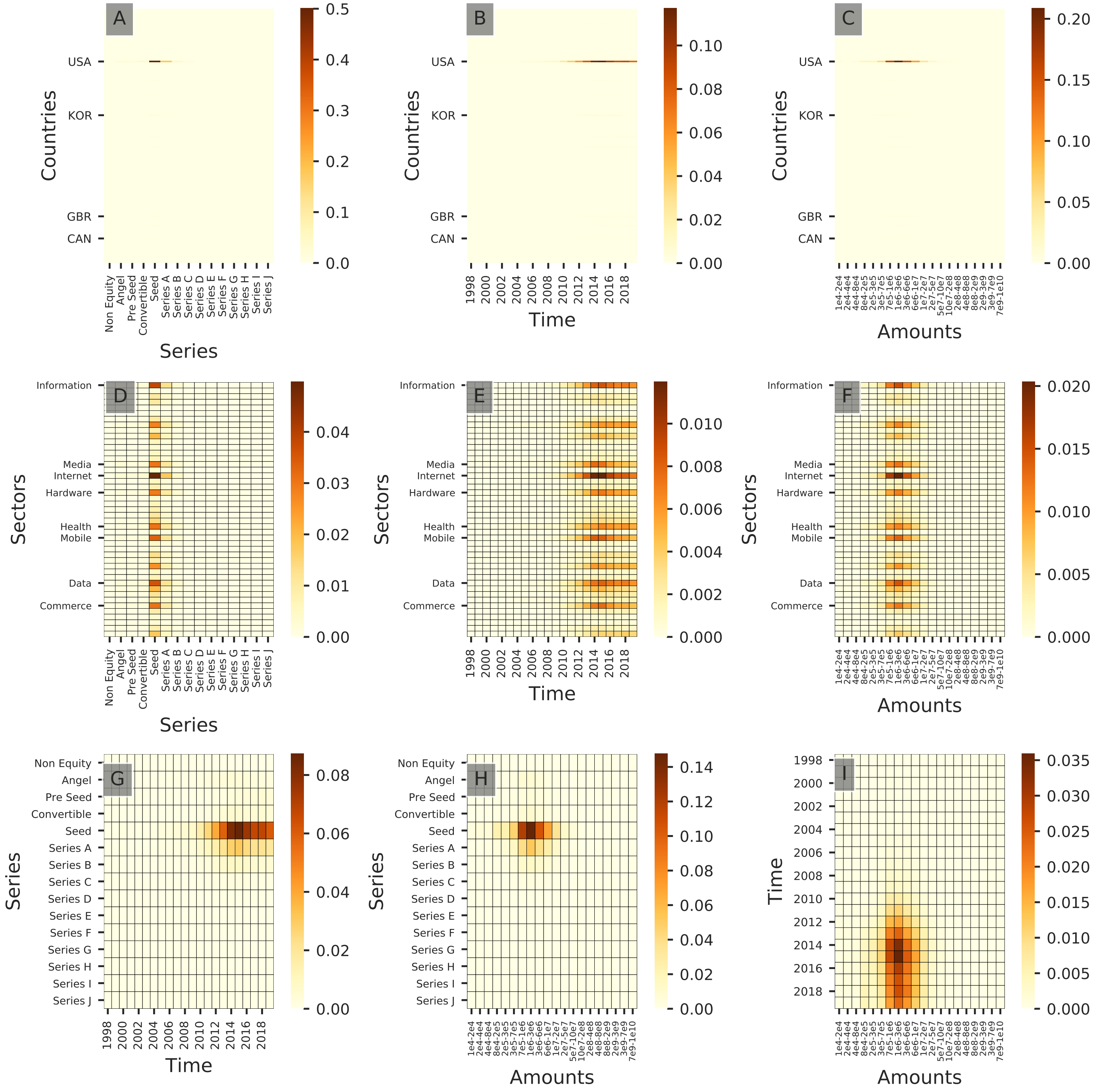

Fig S109. Cross graph interaction heatmap of community D6.

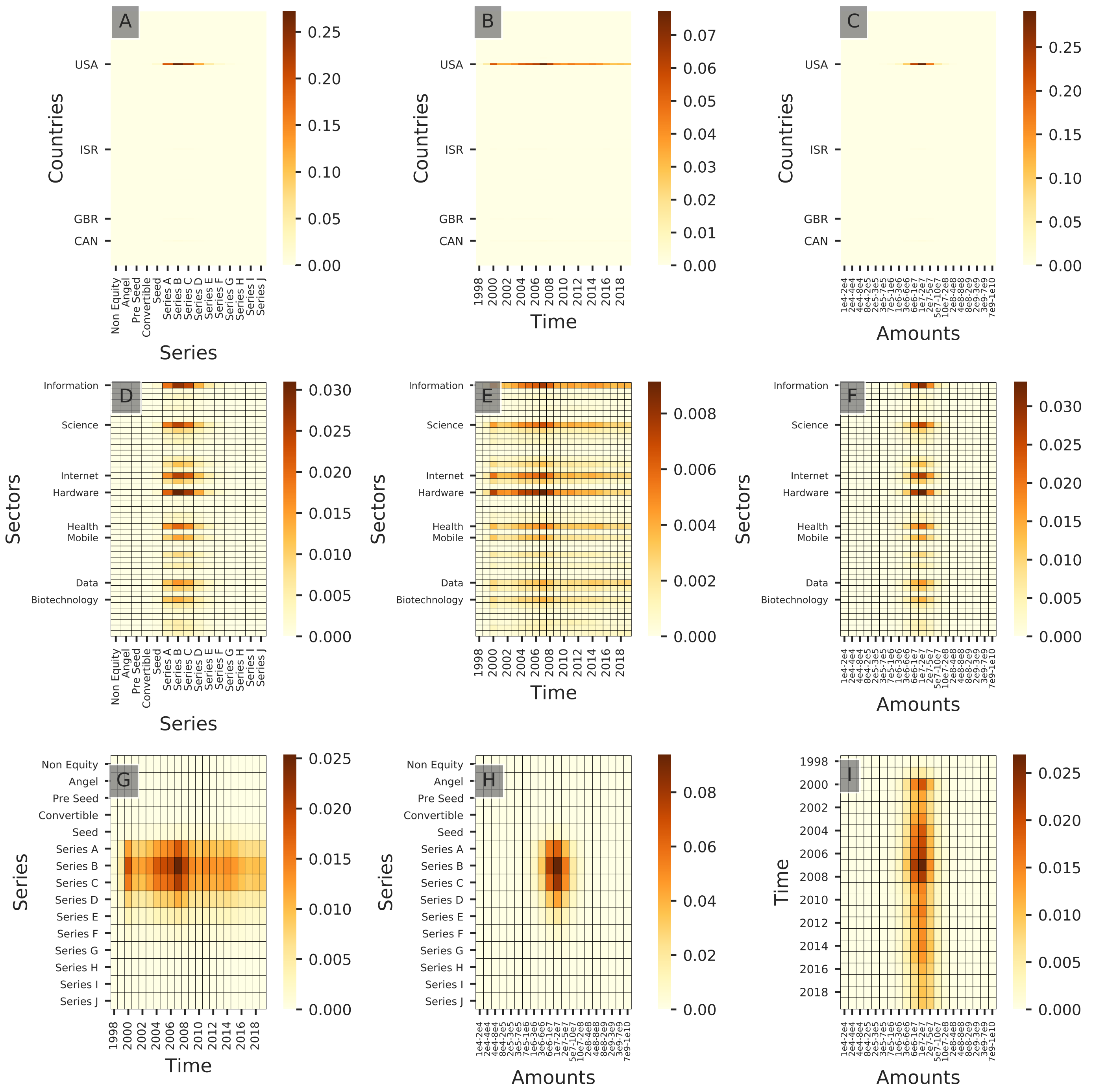

Fig S110. Cross graph interaction heatmap of community D7.

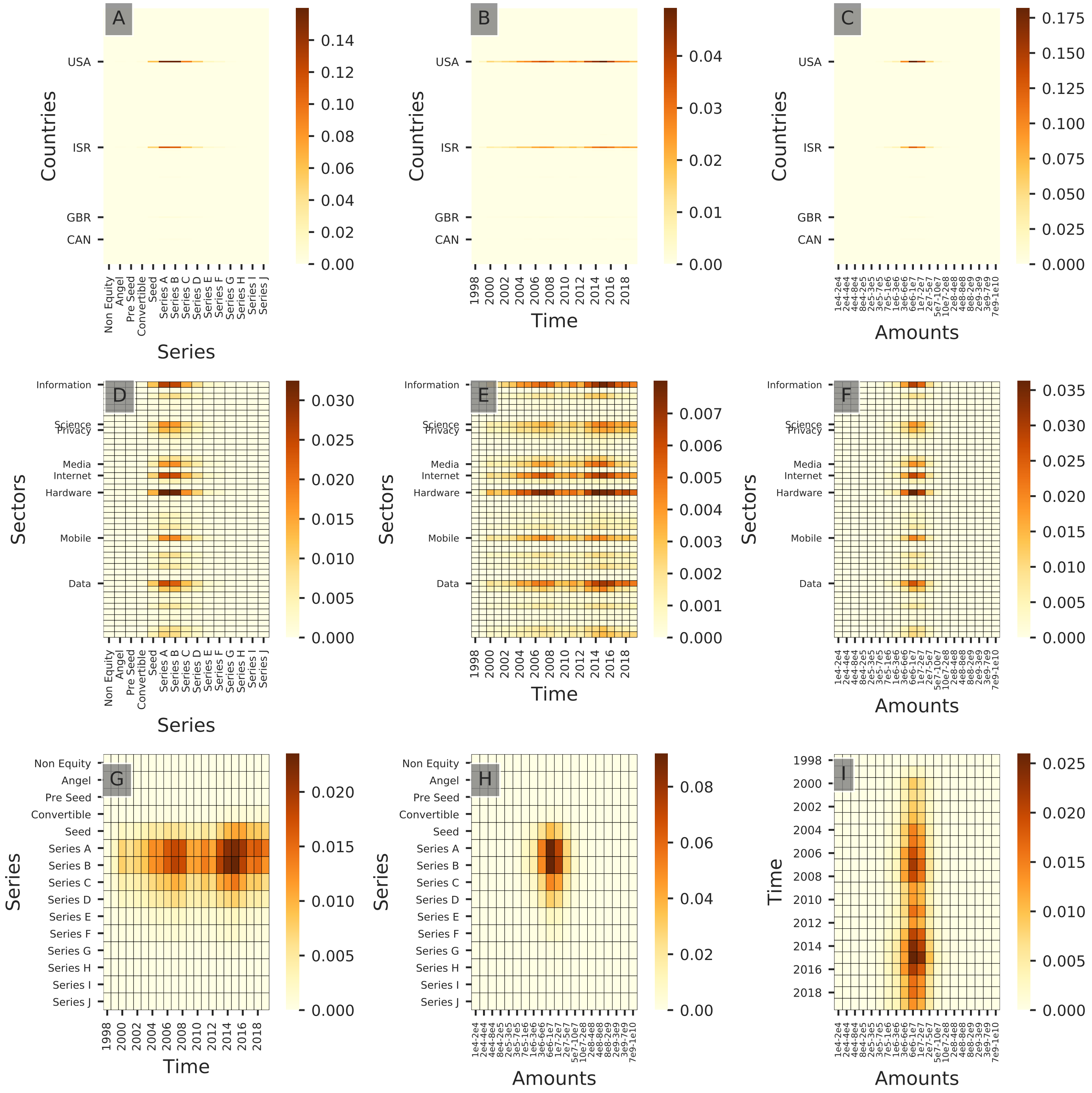

Fig S111. Cross graph interaction heatmap of community D8.

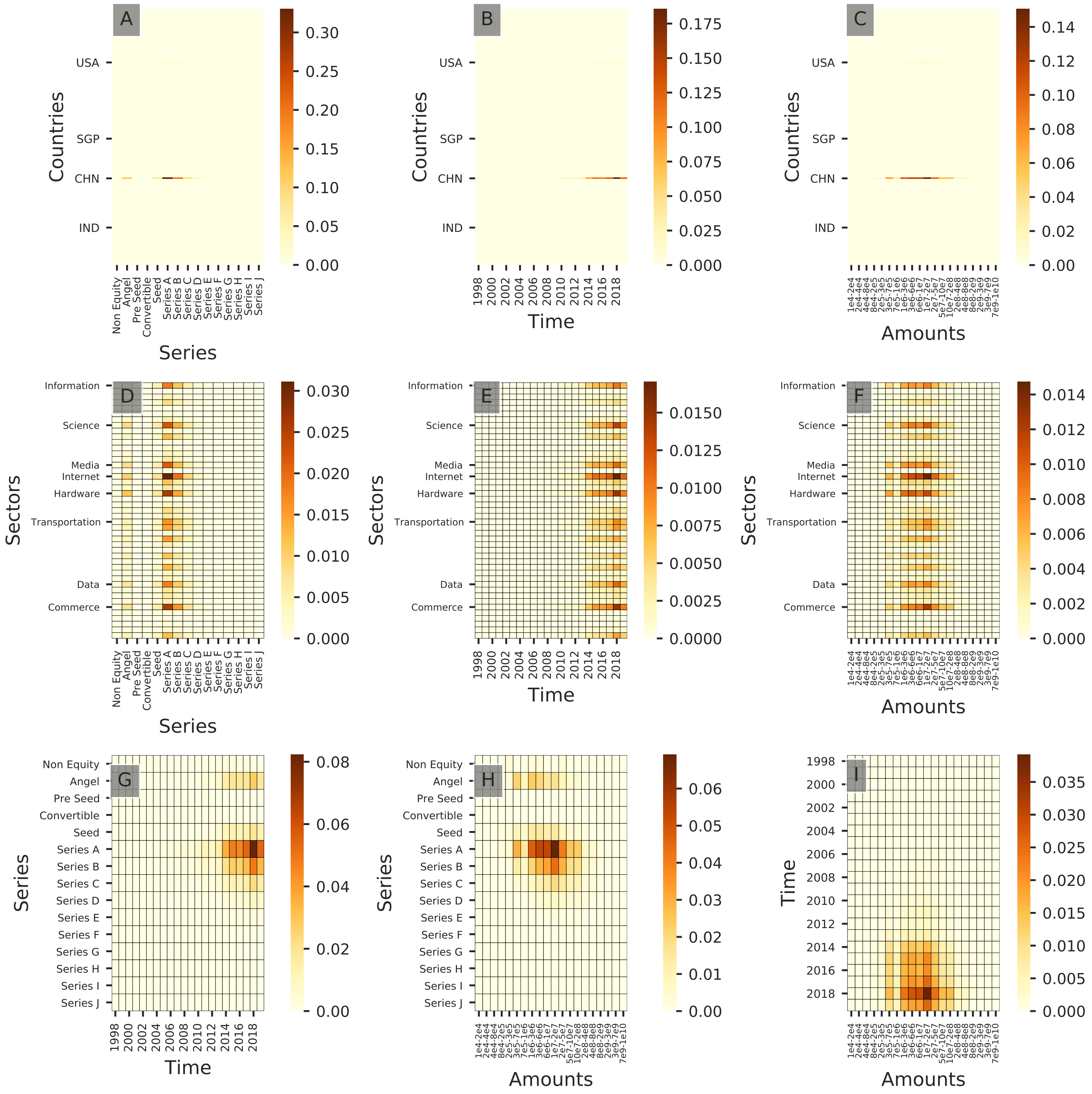

Fig S112. Cross graph interaction heatmap of community D9.

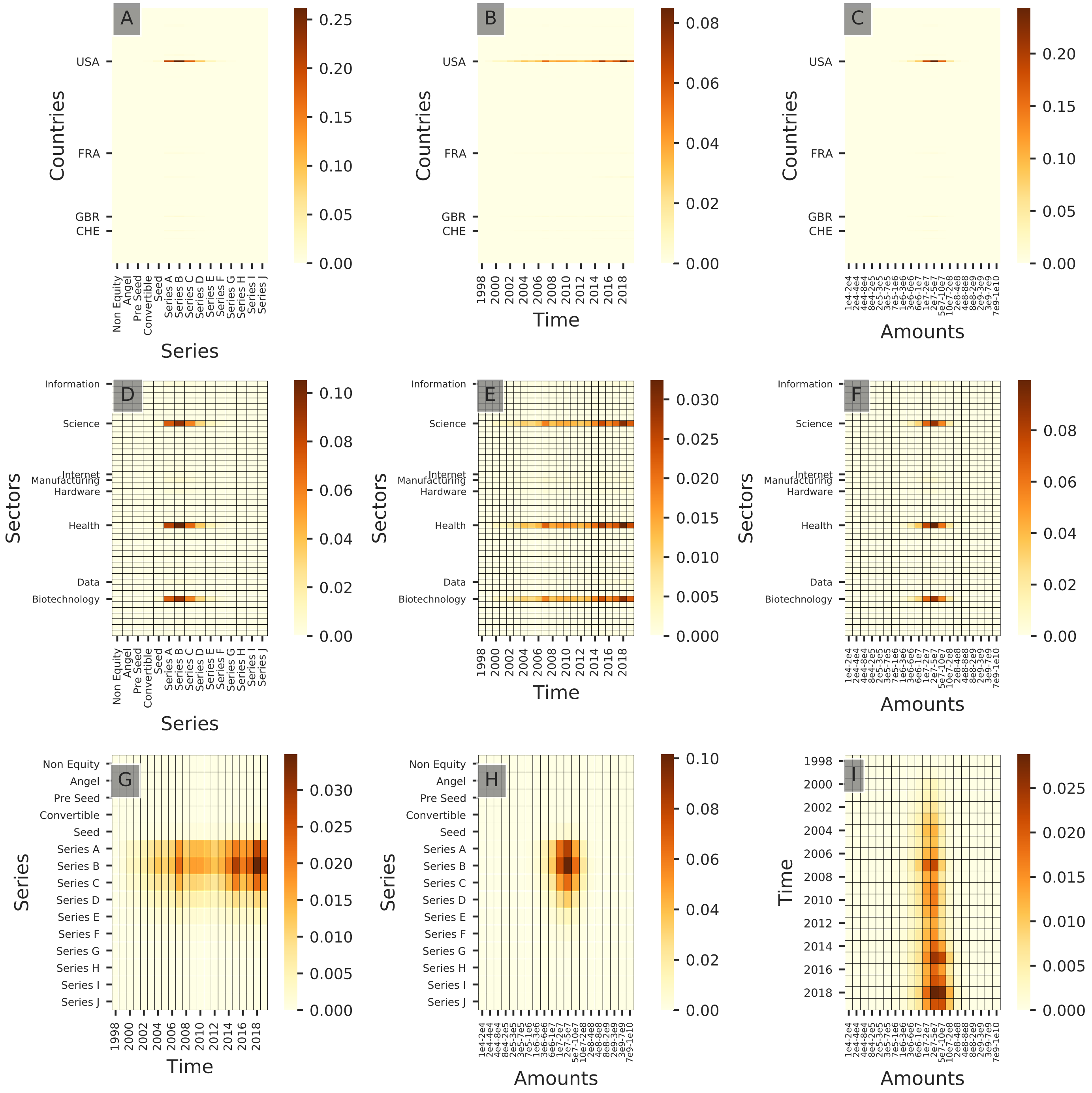

Fig S113. Cross graph interaction heatmap of community D10.

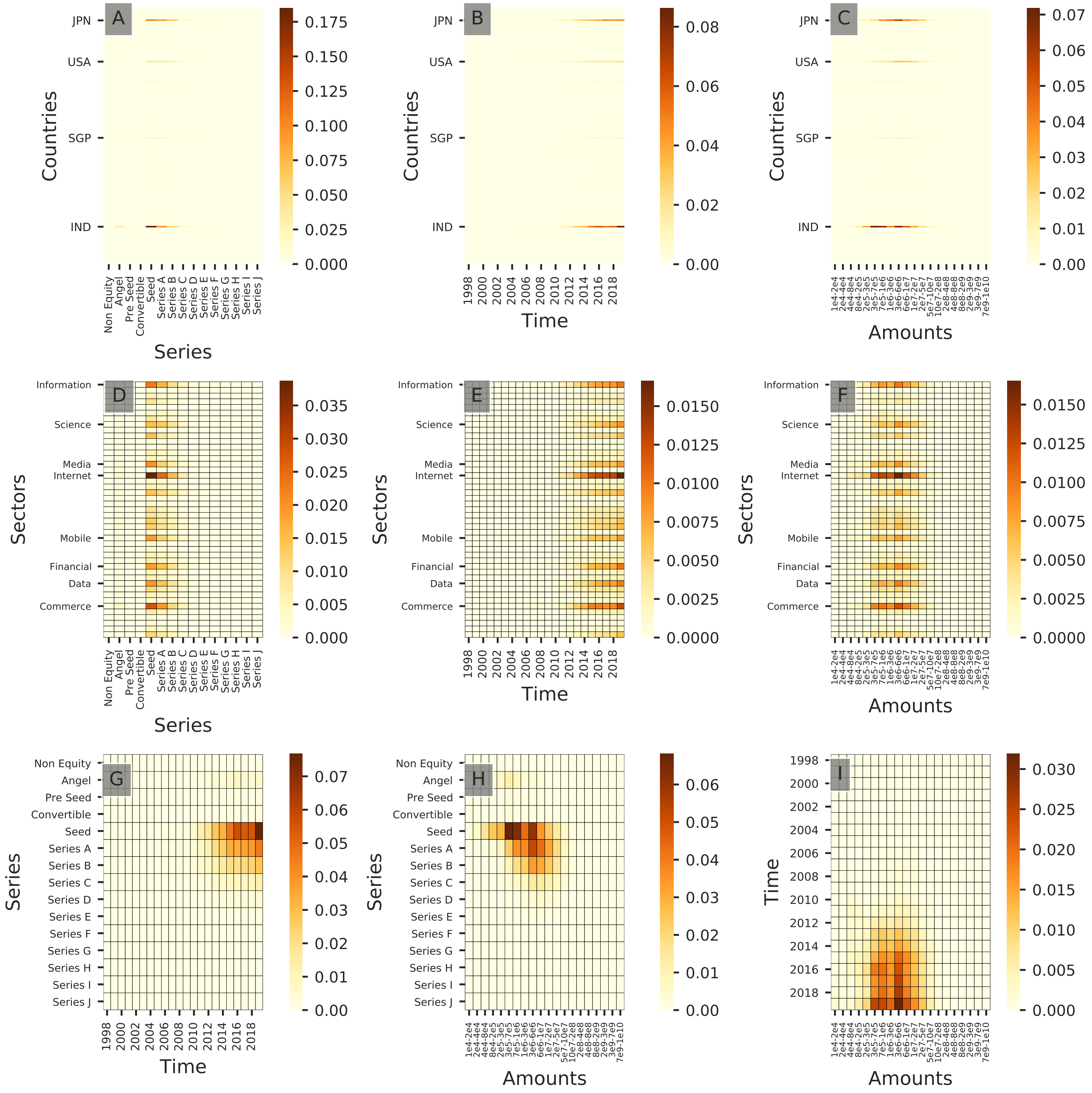

Fig S114. Cross graph interaction heatmap of community D11.

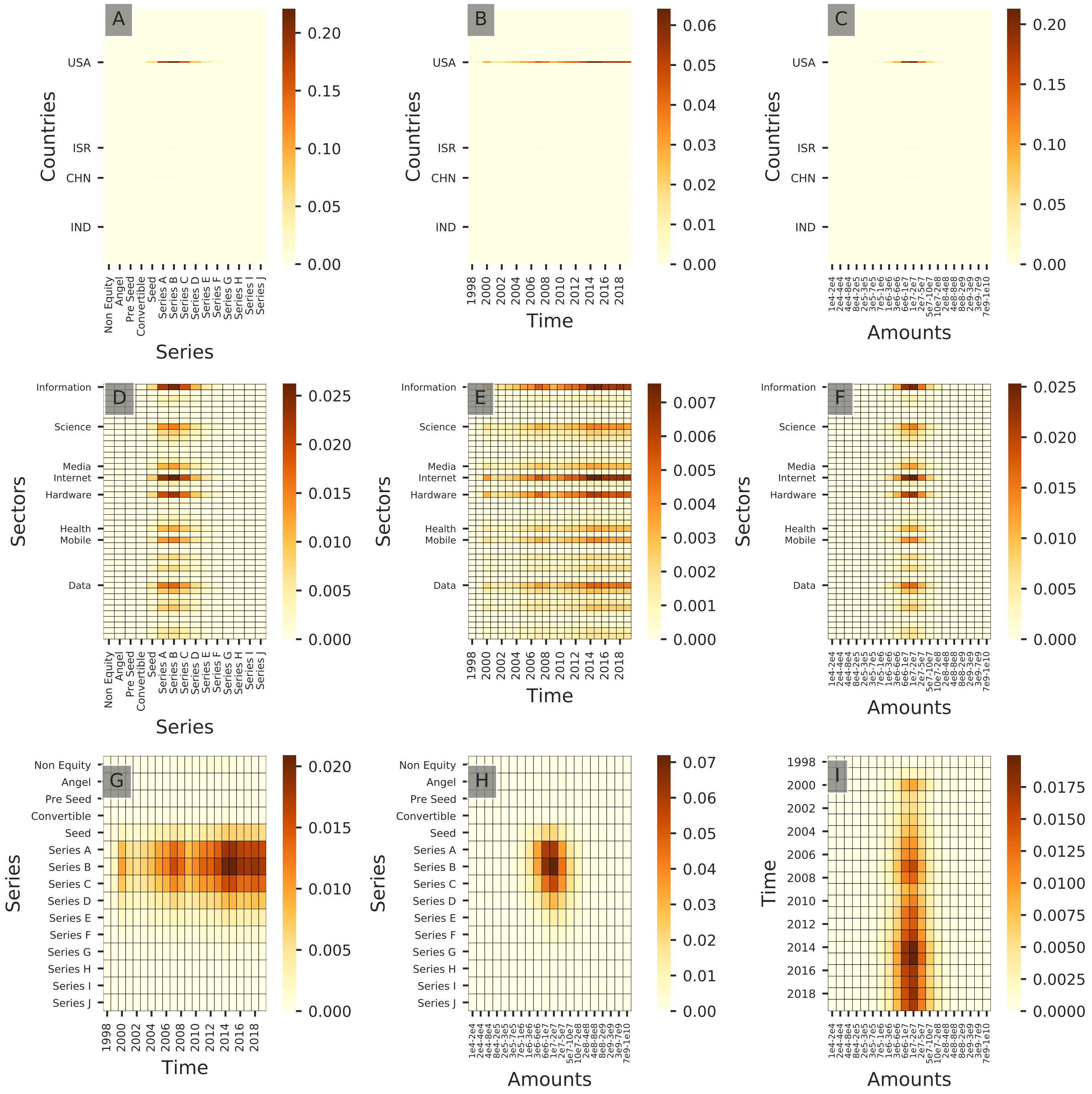

Fig S115. Cross graph interaction heatmap of community E0.

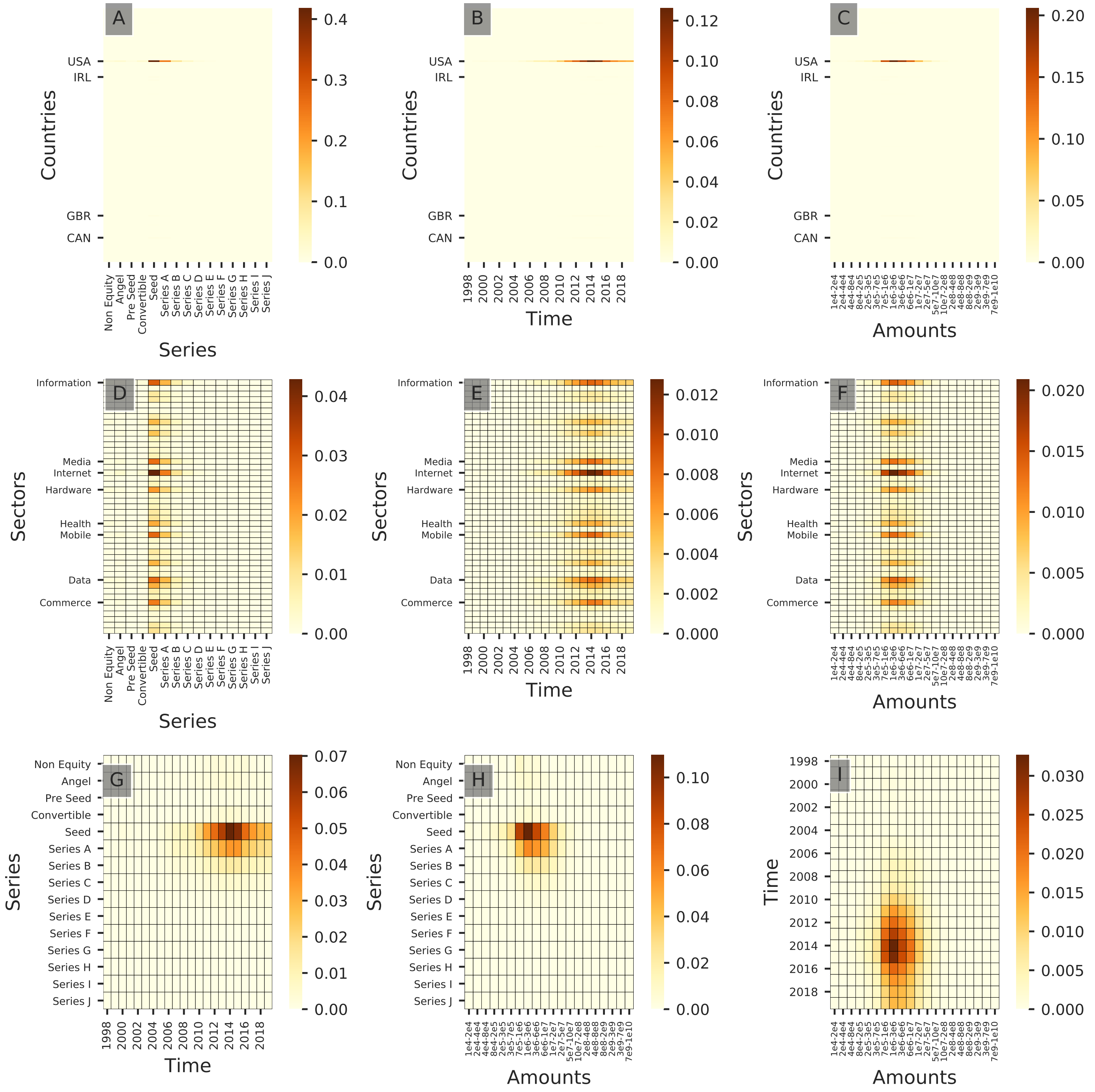

Fig S116. Cross graph interaction heatmap of community E1.

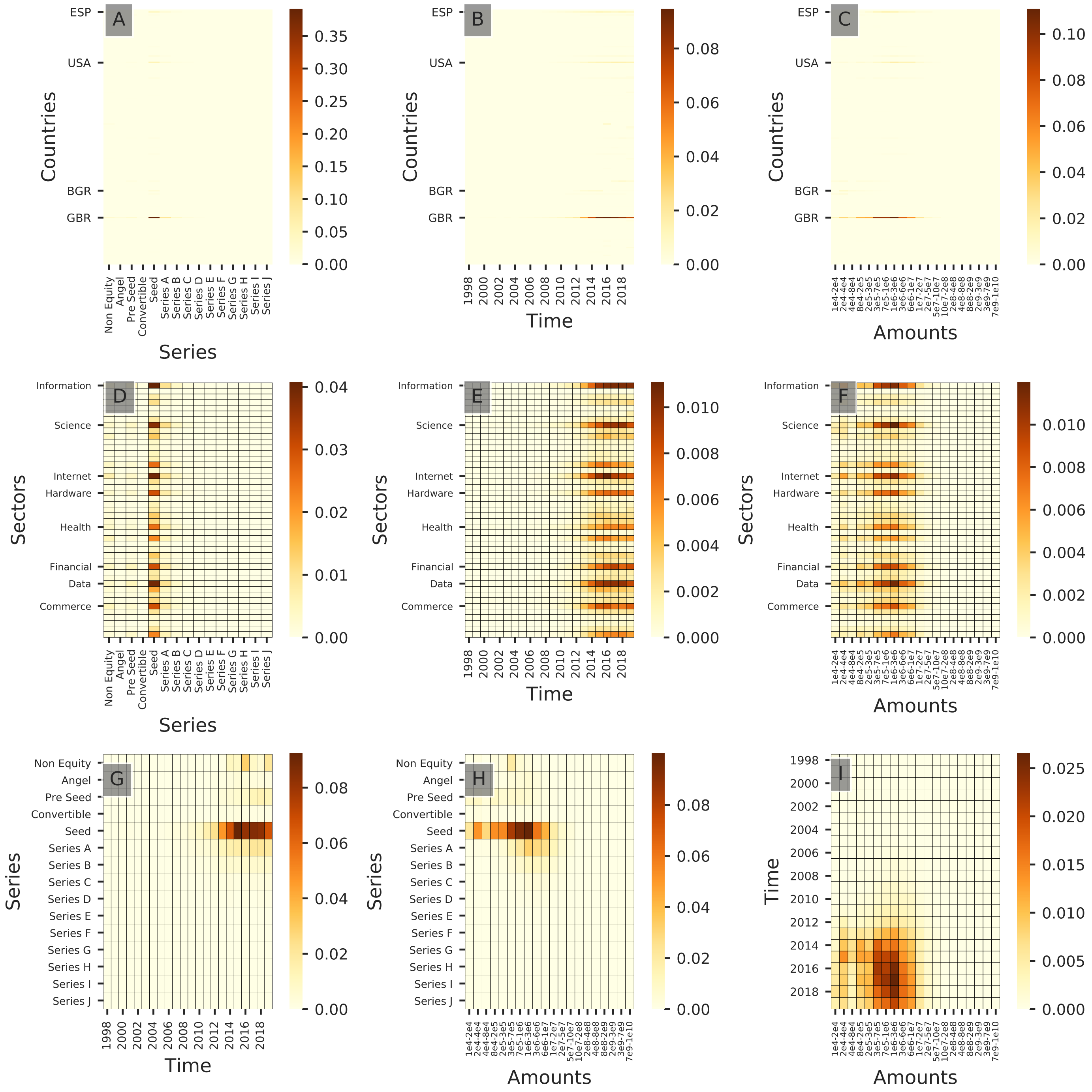

Fig S117. Cross graph interaction heatmap of community E2.

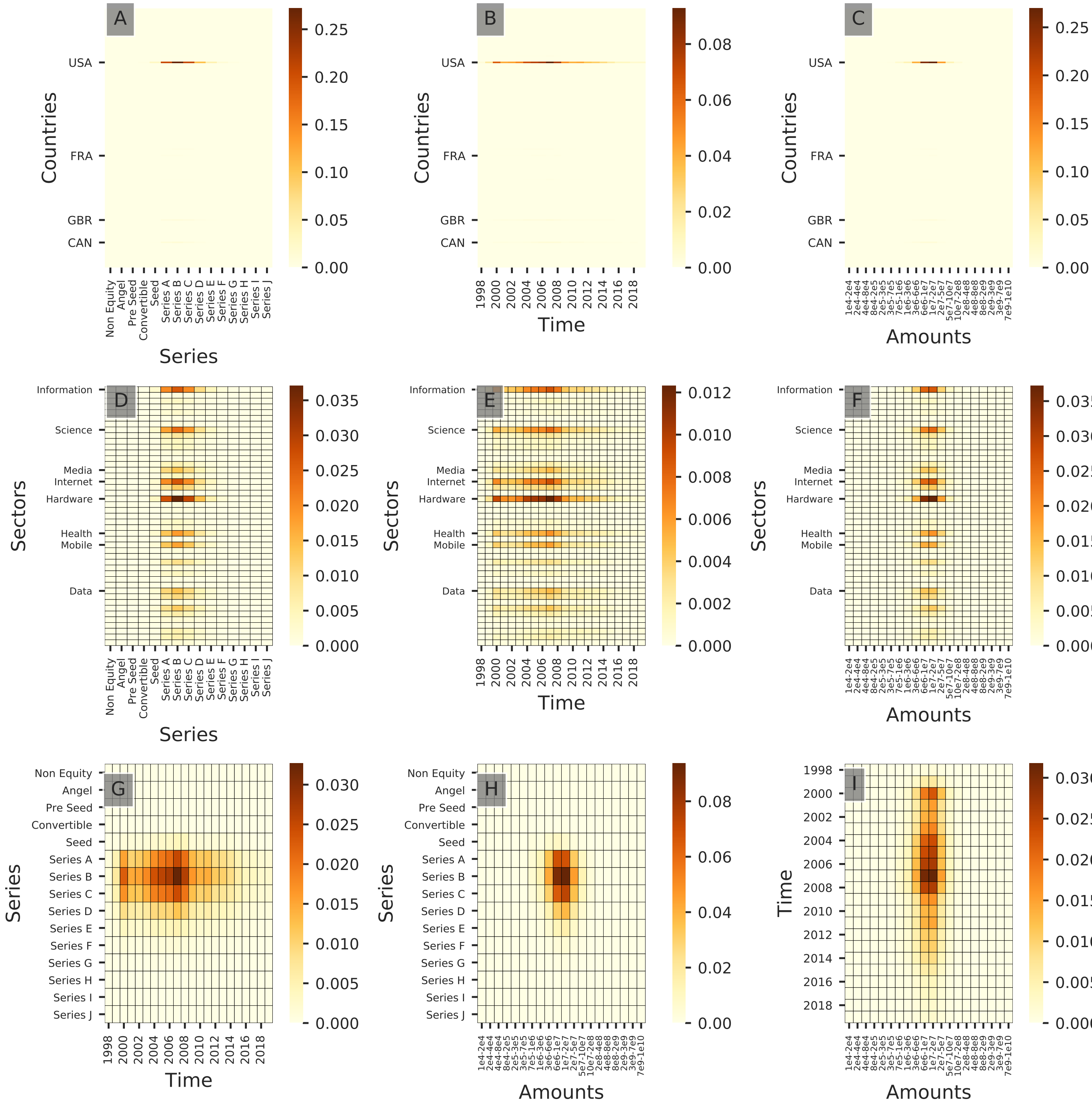

Fig S118. Cross graph interaction heatmap of community E3.

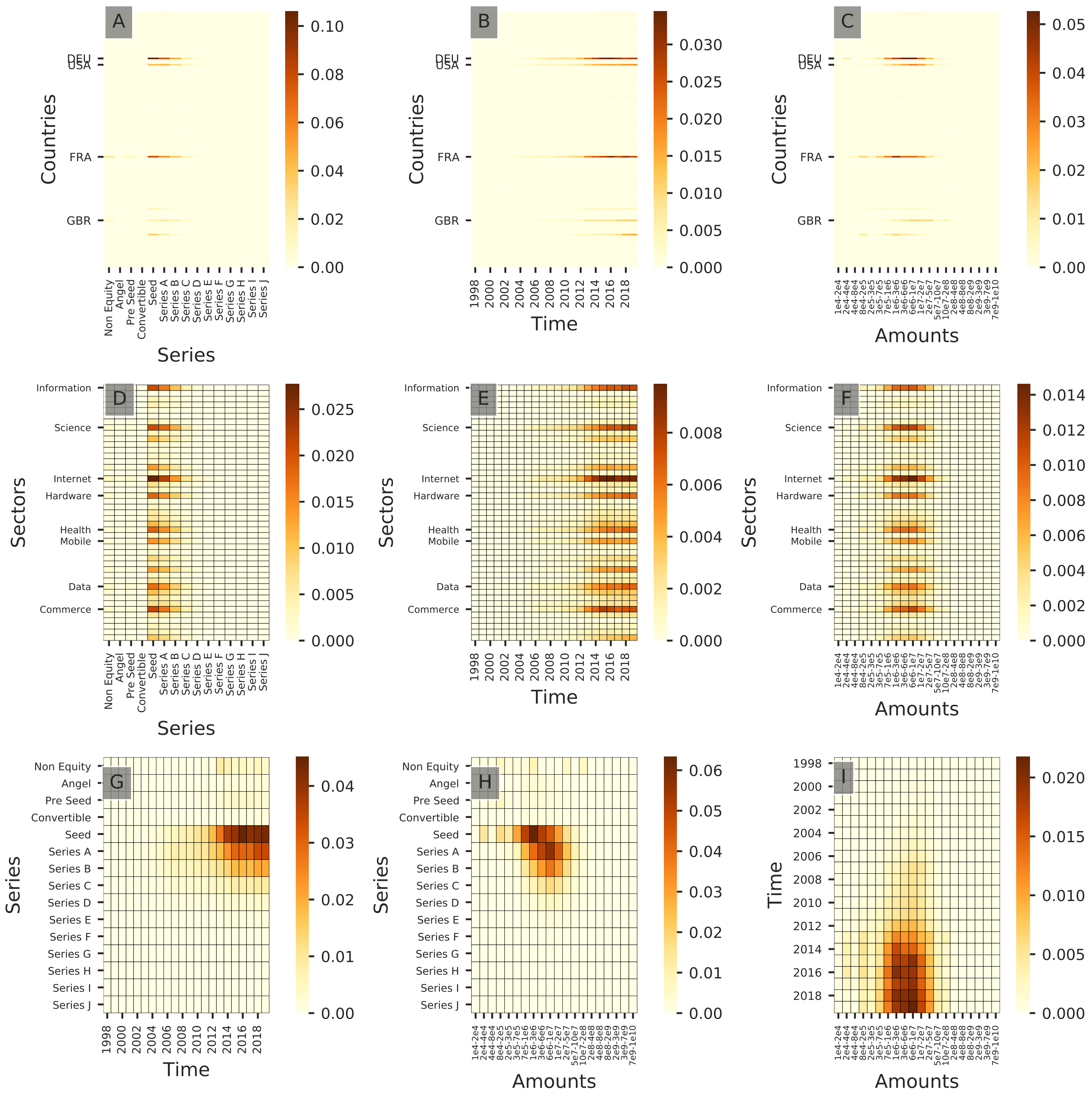

Fig S119. Cross graph interaction heatmap of community E4.

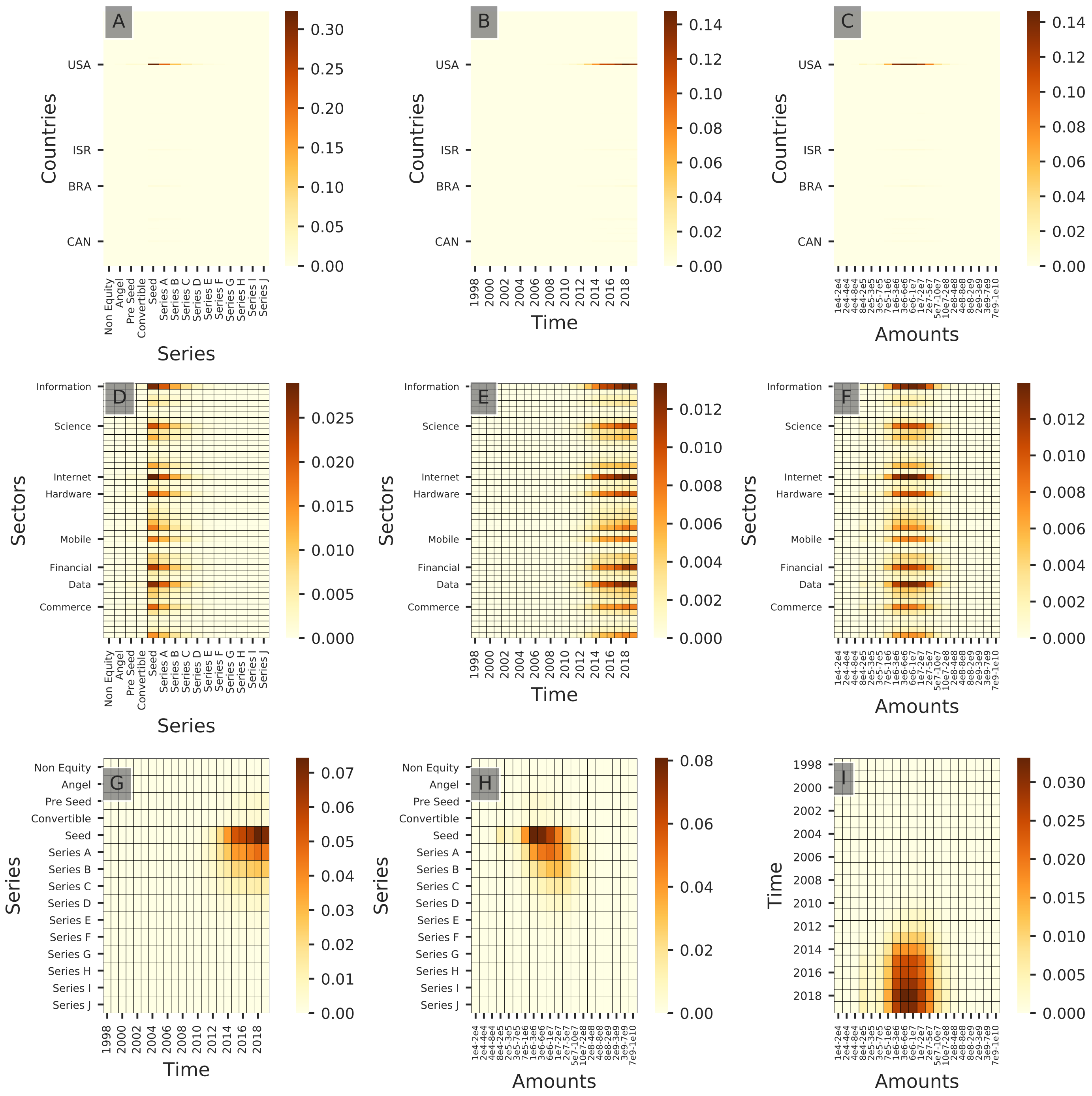

Fig S120. Cross graph interaction heatmap of community E5.

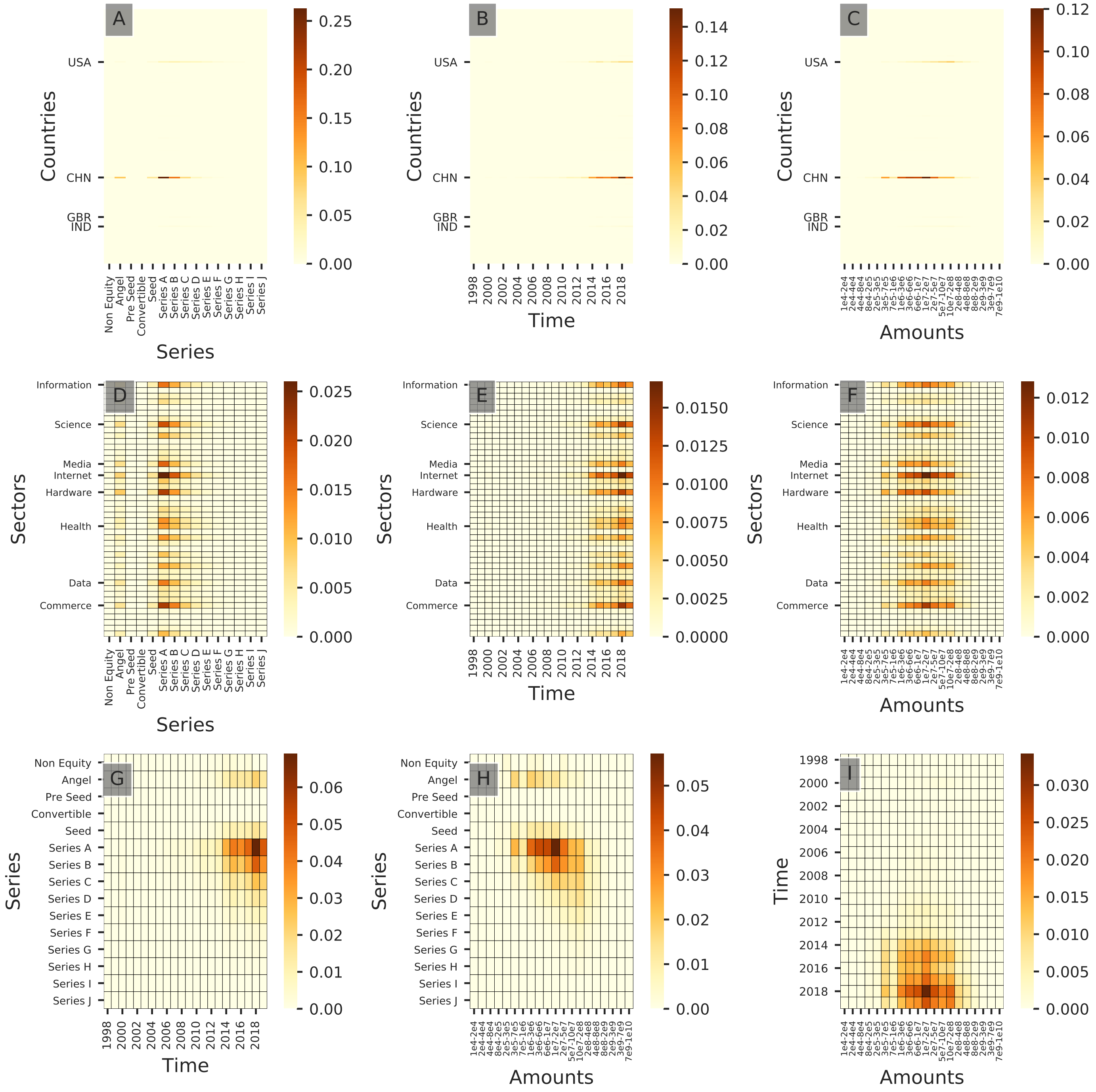

Fig S121. Cross graph interaction heatmap of community E6.

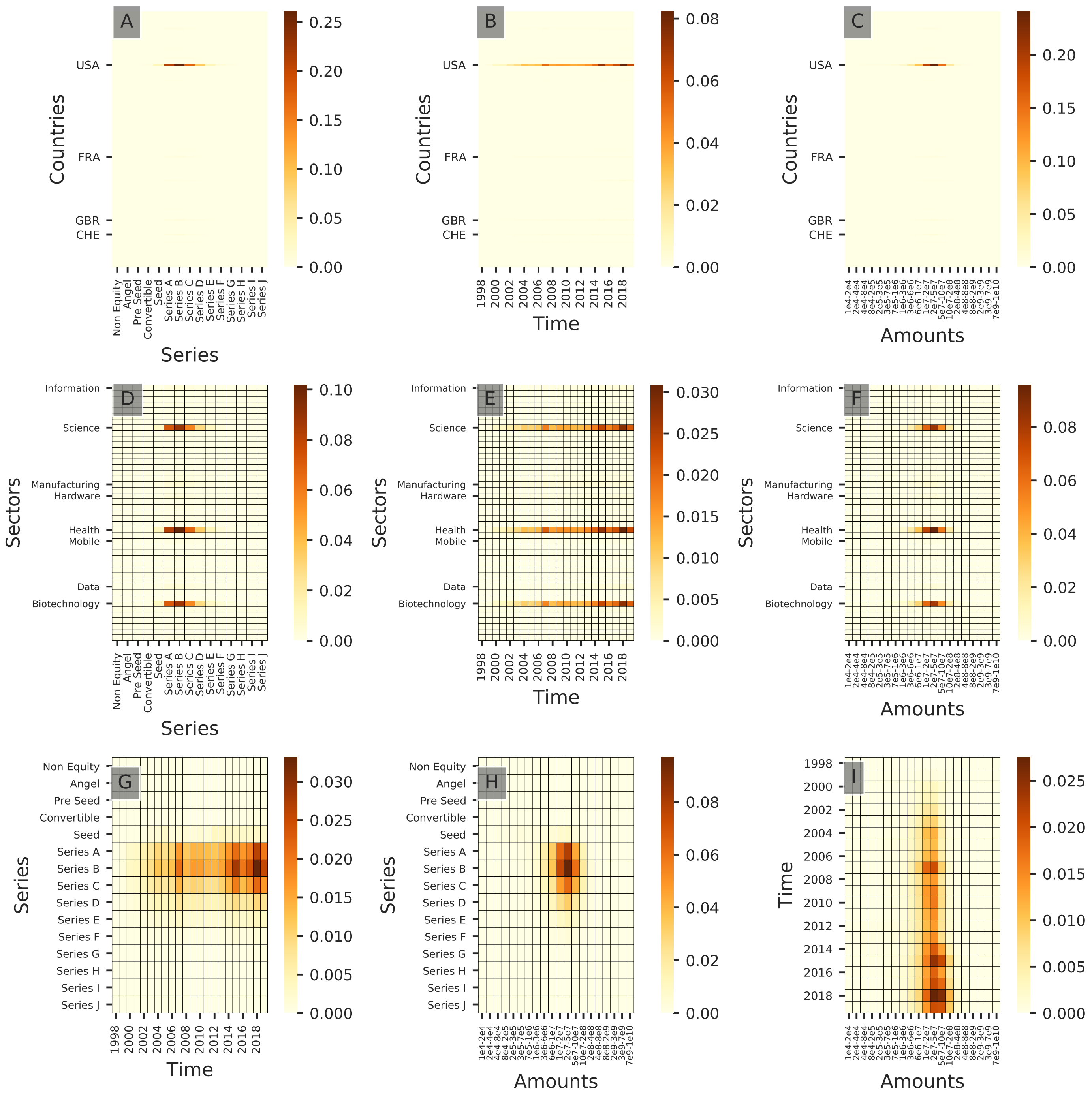

Fig S122. Cross graph interaction heatmap of community E7.

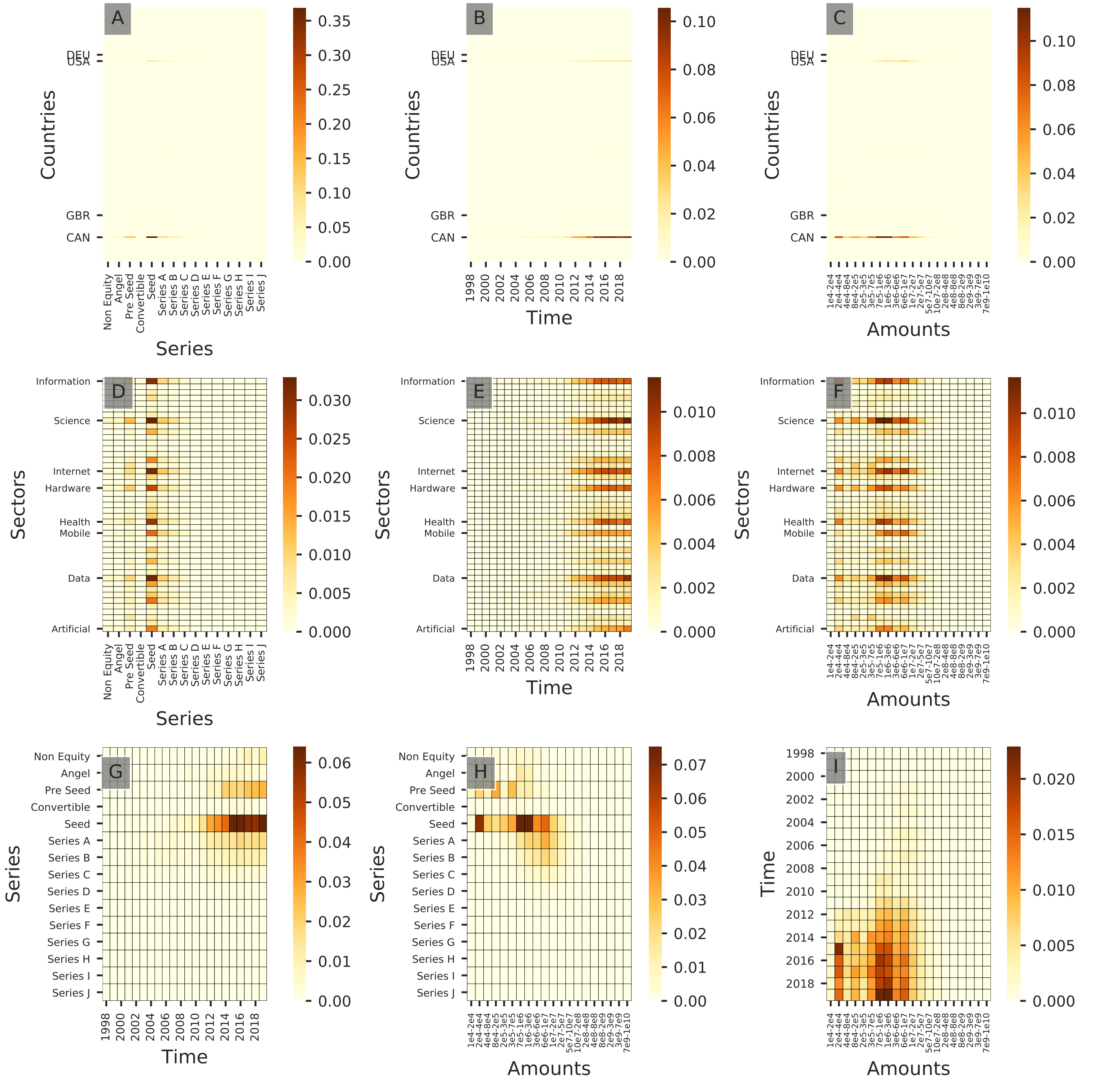

Fig S123. Cross graph interaction heatmap of community E8.

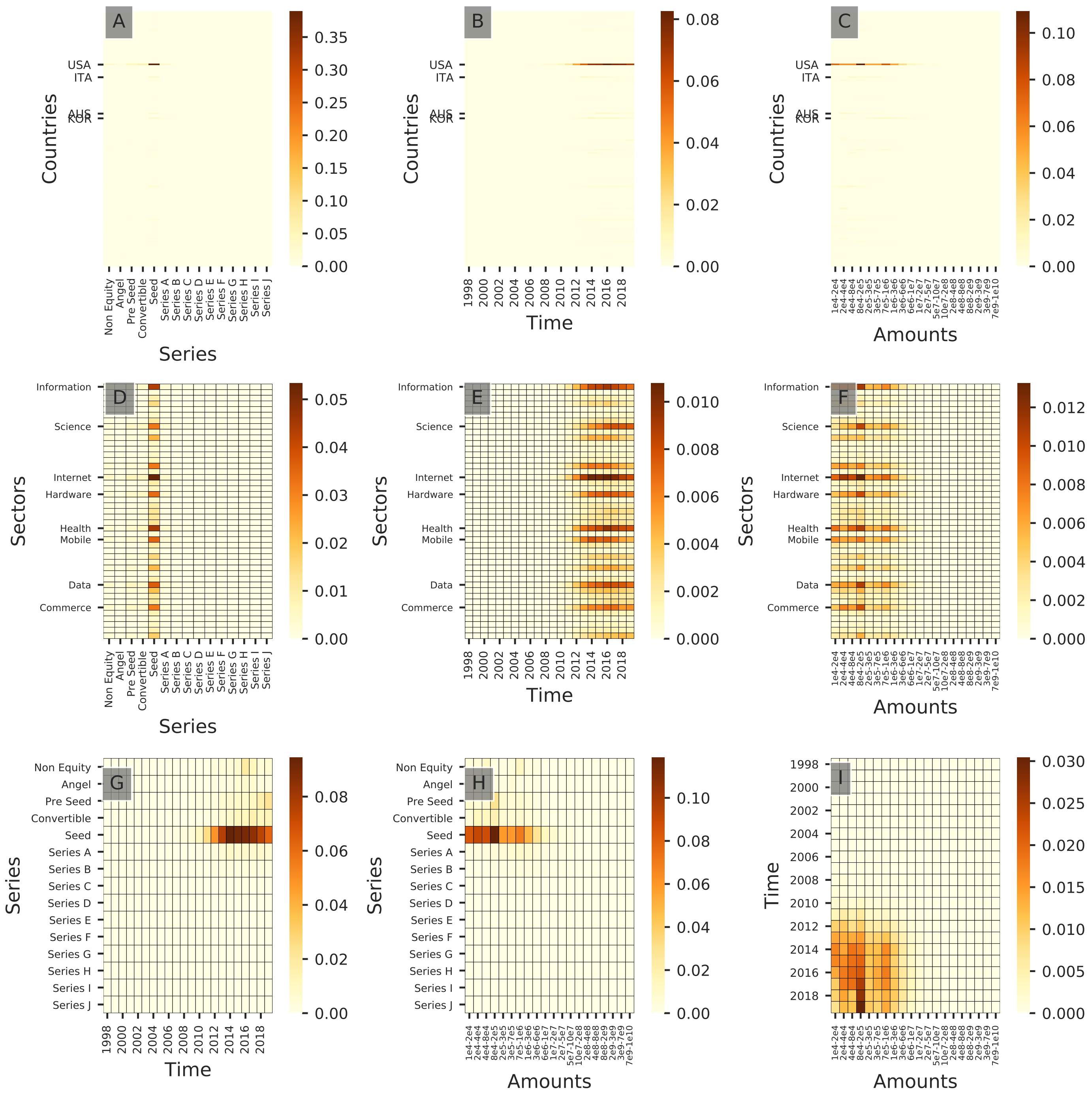

Fig S124. Cross graph interaction heatmap of community E9.

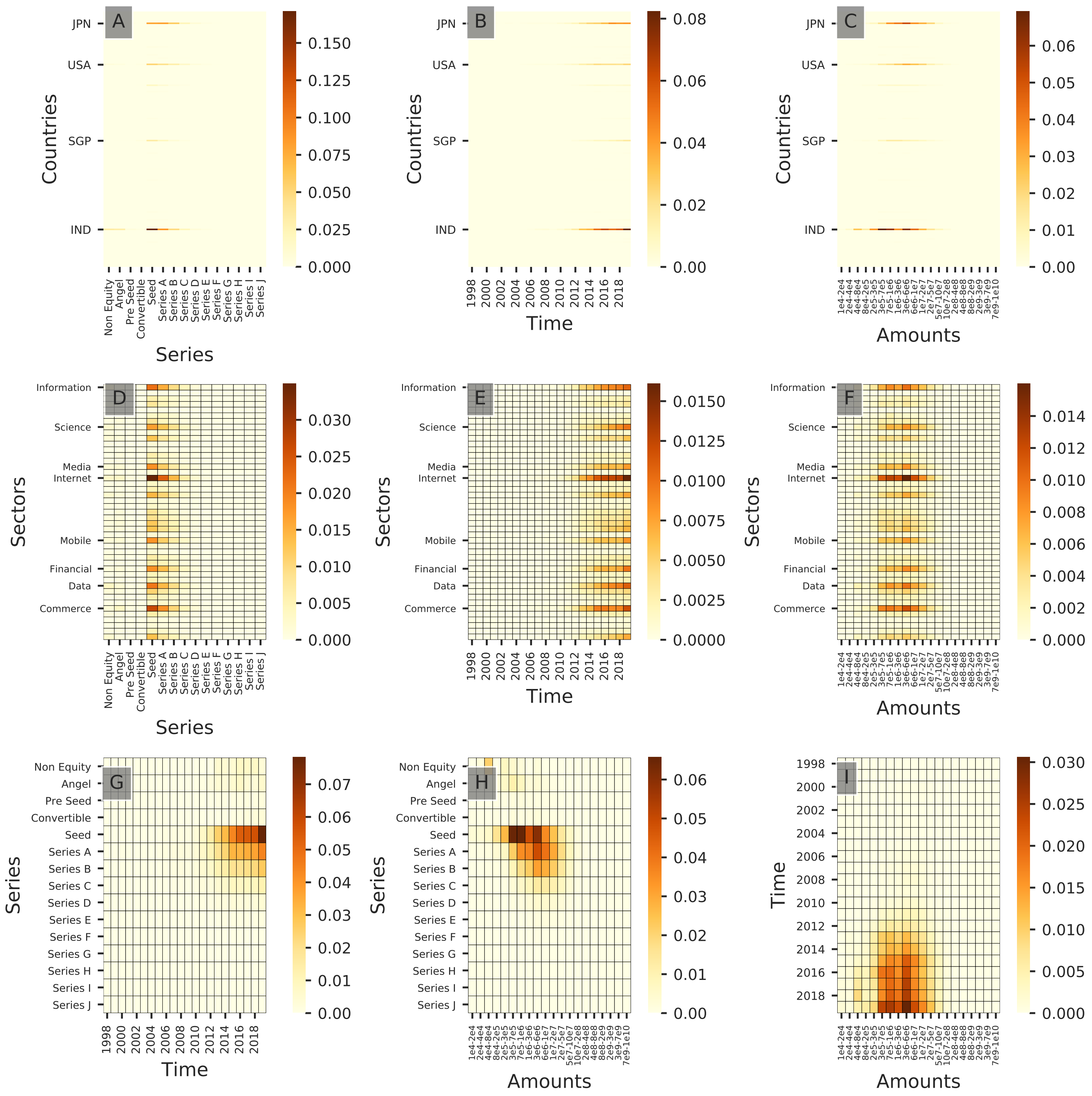

Fig S125. Cross graph interaction heatmap of community E10.

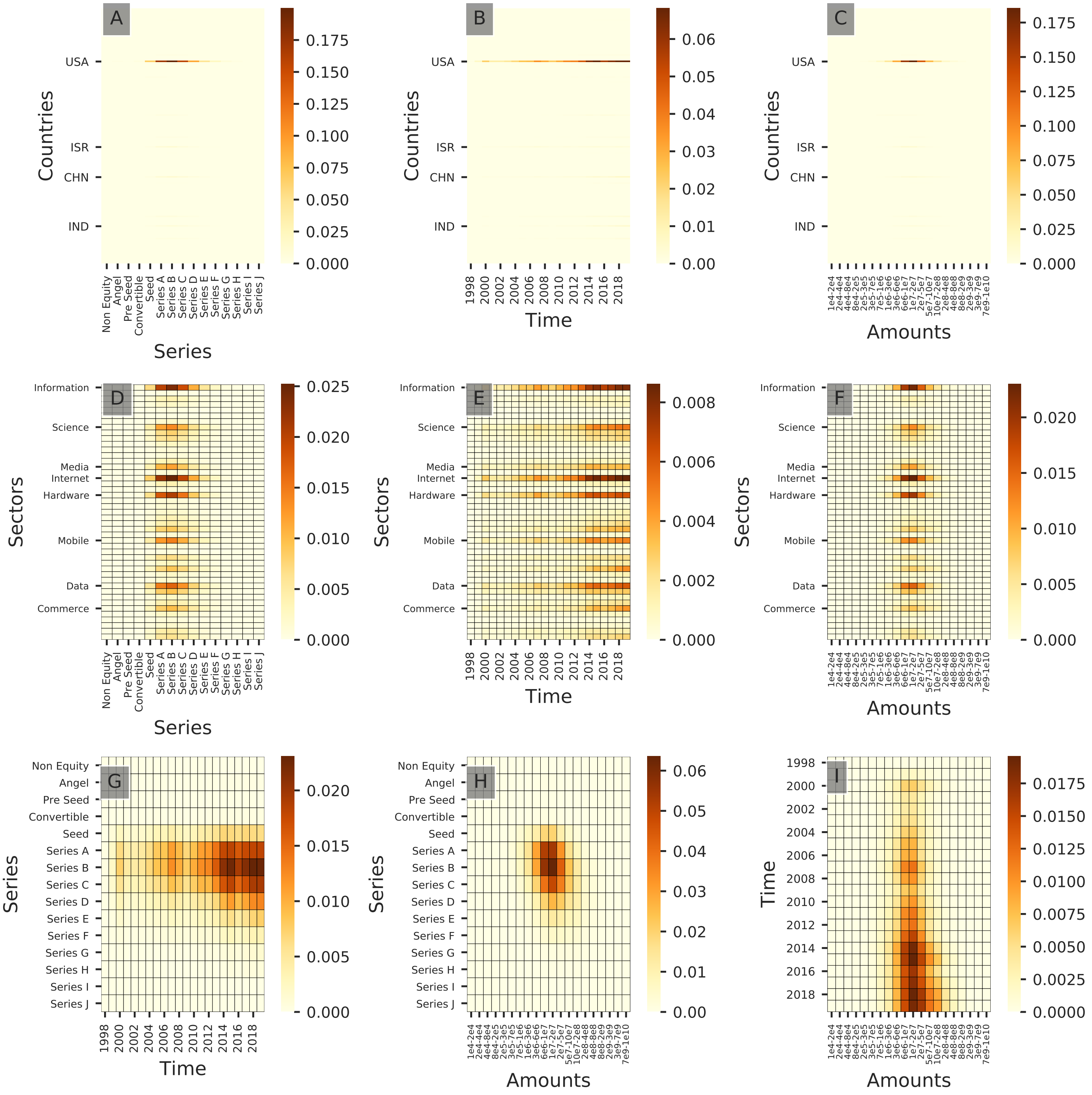

Fig S126. Cross graph interaction heatmap of community F0.

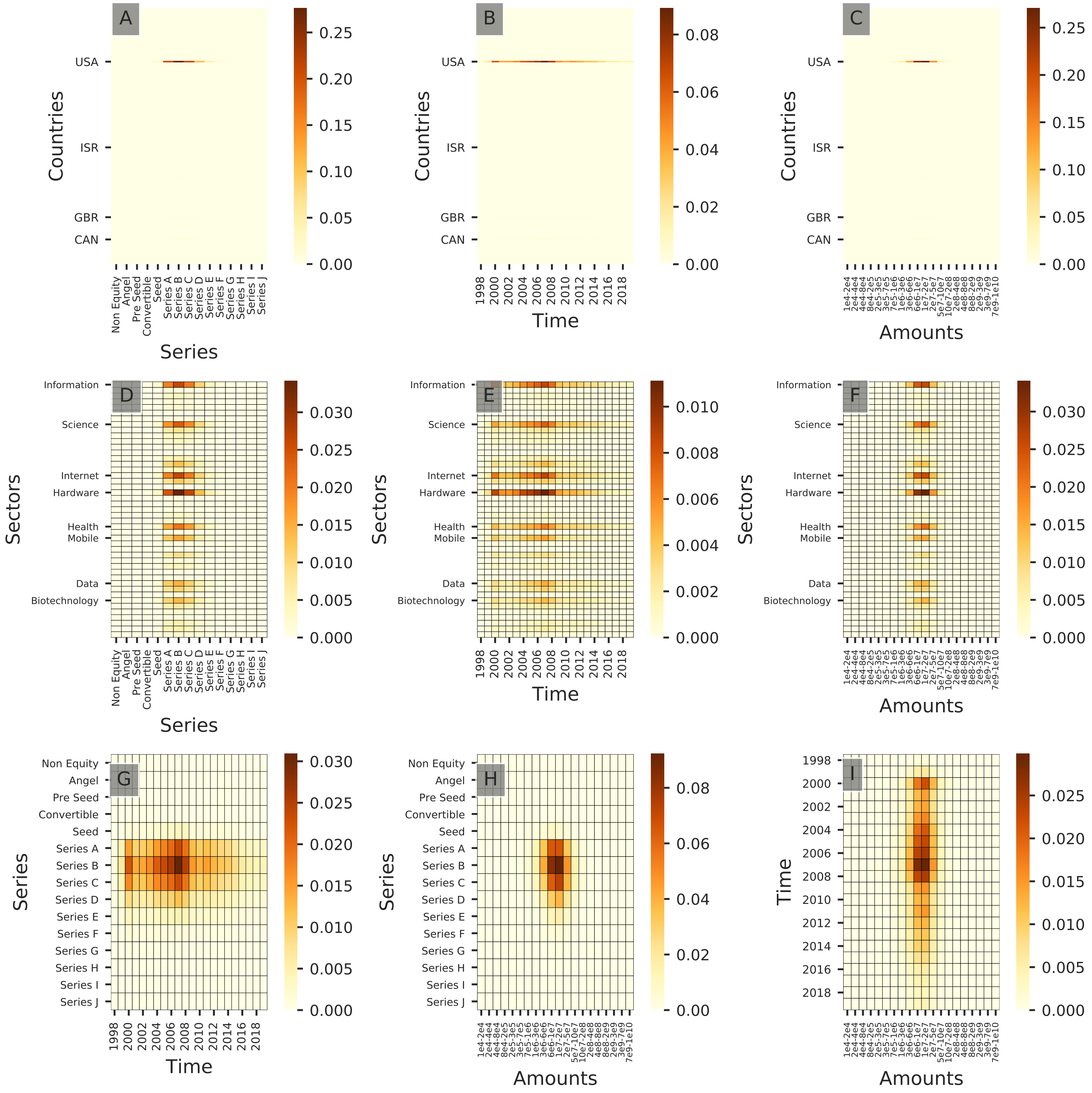

Fig S127. Cross graph interaction heatmap of community F1.

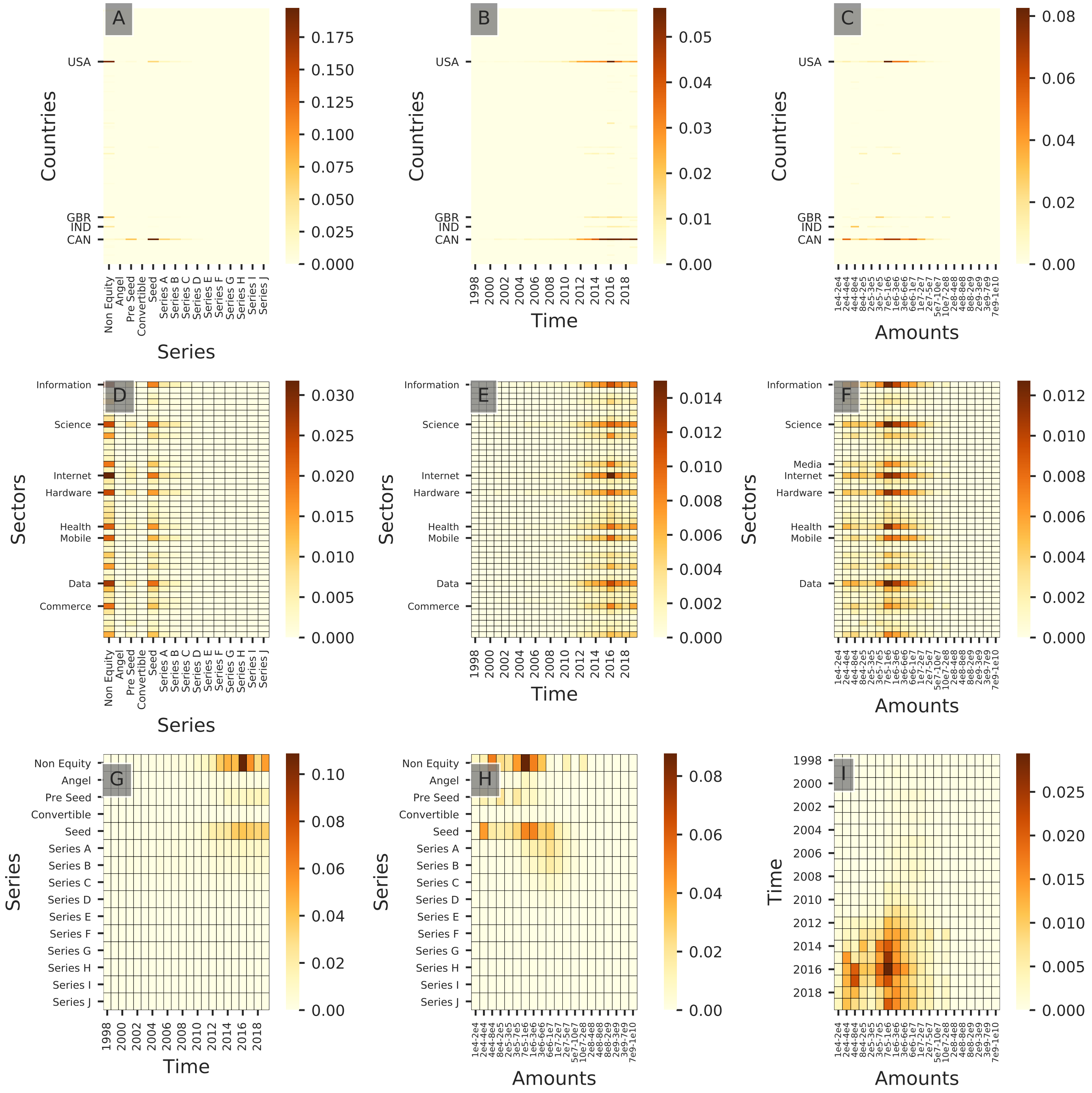

Fig S128. Cross graph interaction heatmap of community F2.

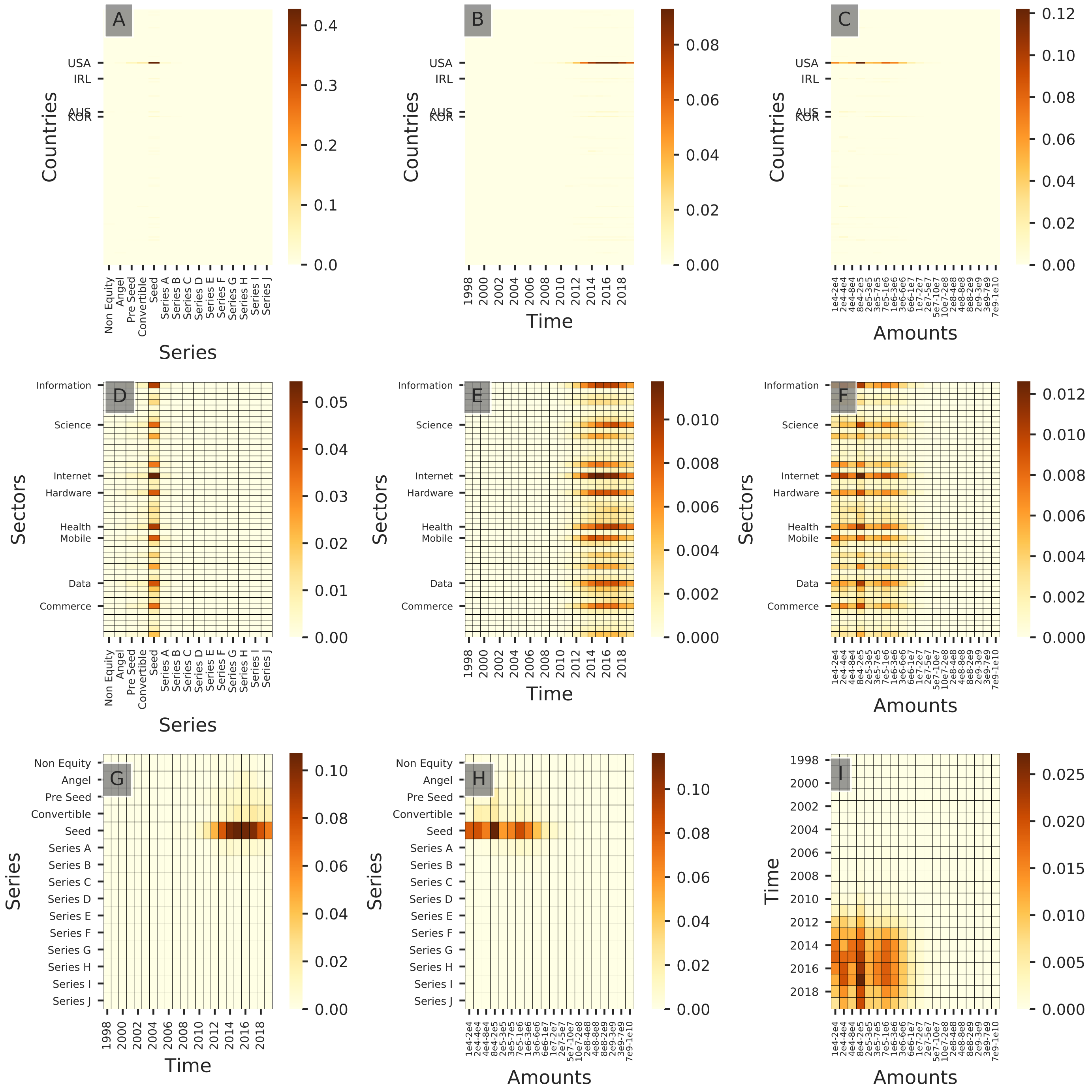

Fig S129. Cross graph interaction heatmap of community F3.

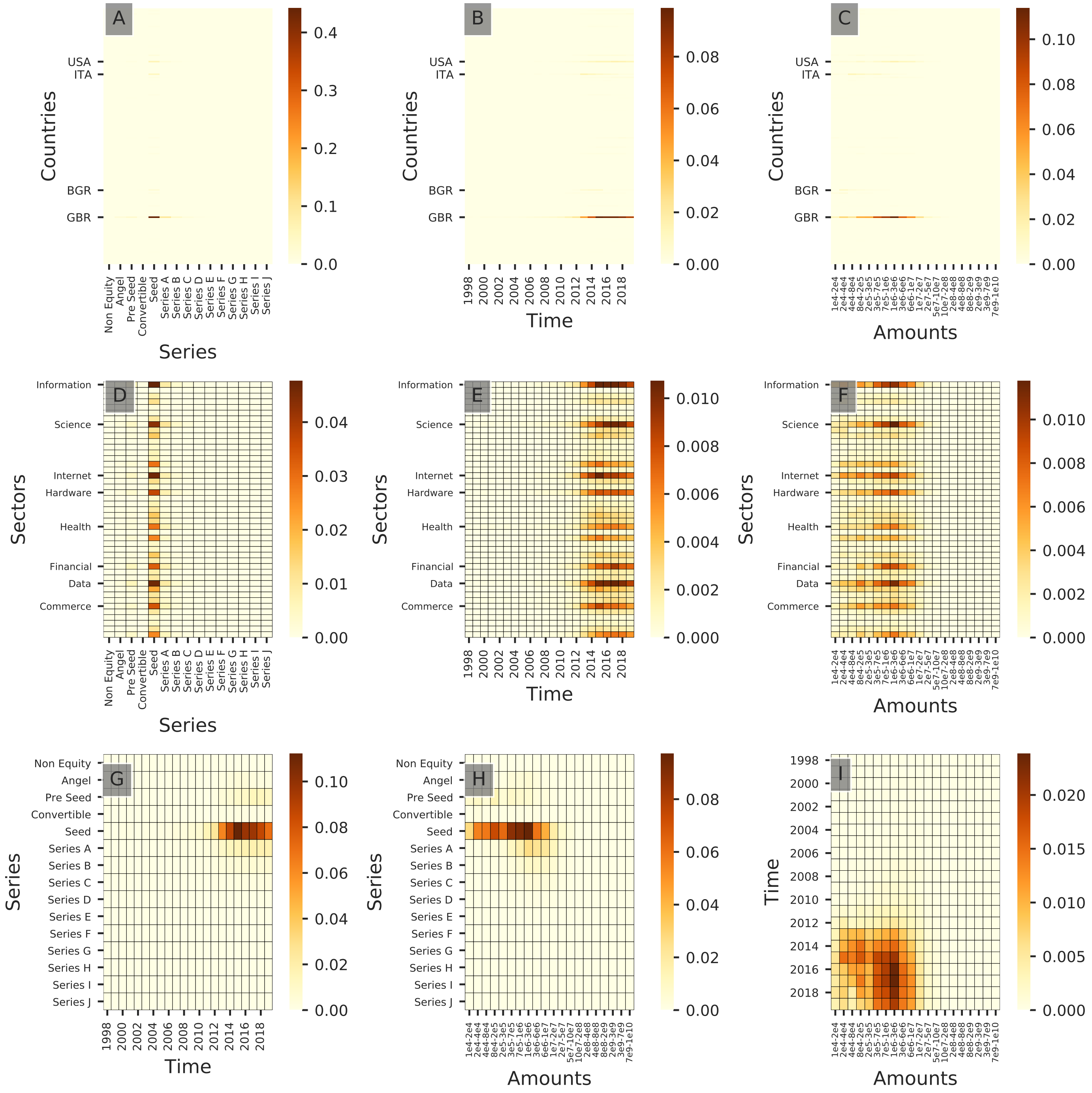

Fig S130. Cross graph interaction heatmap of community F4.

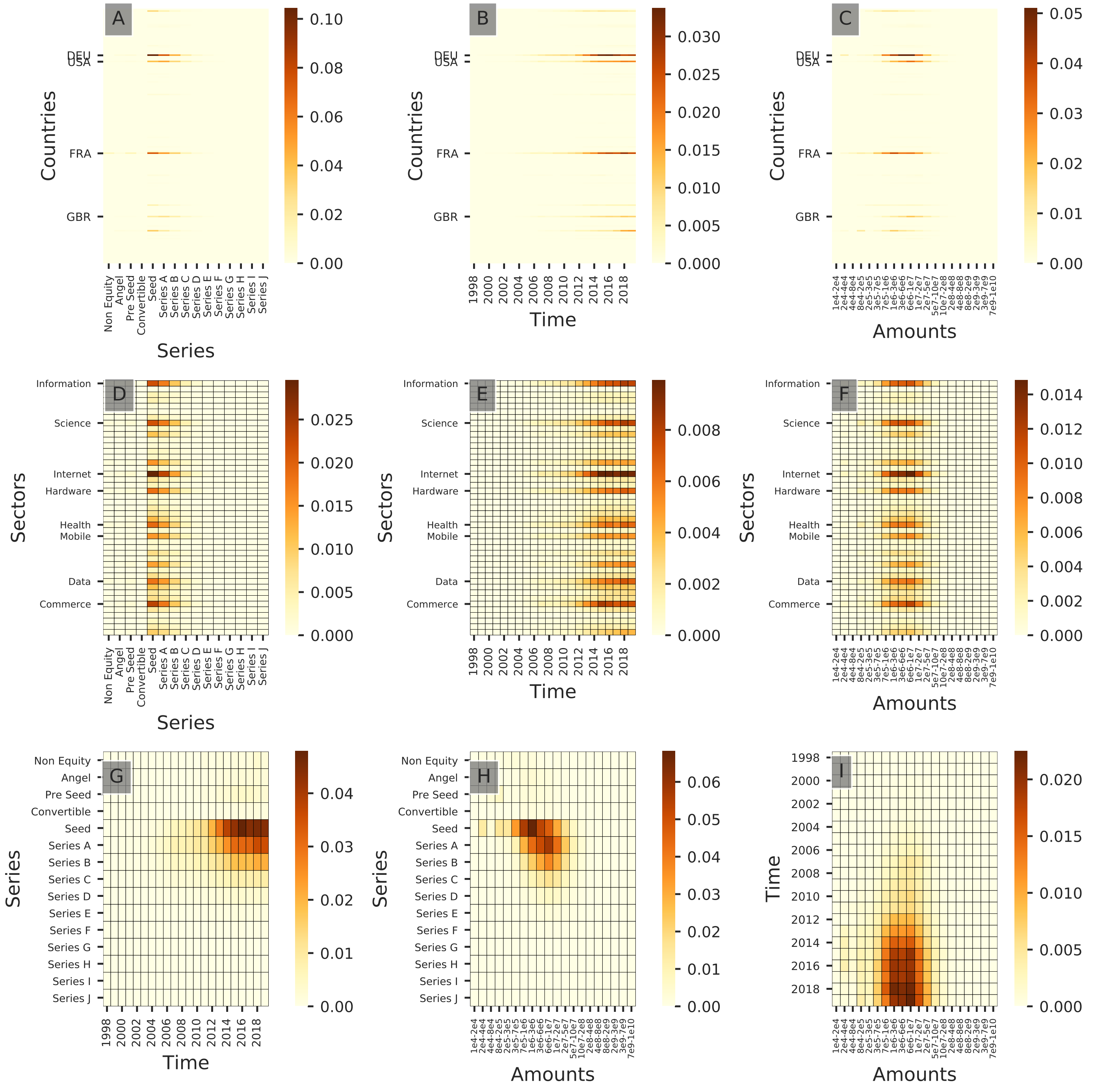

Fig S131. Cross graph interaction heatmap of community F5.

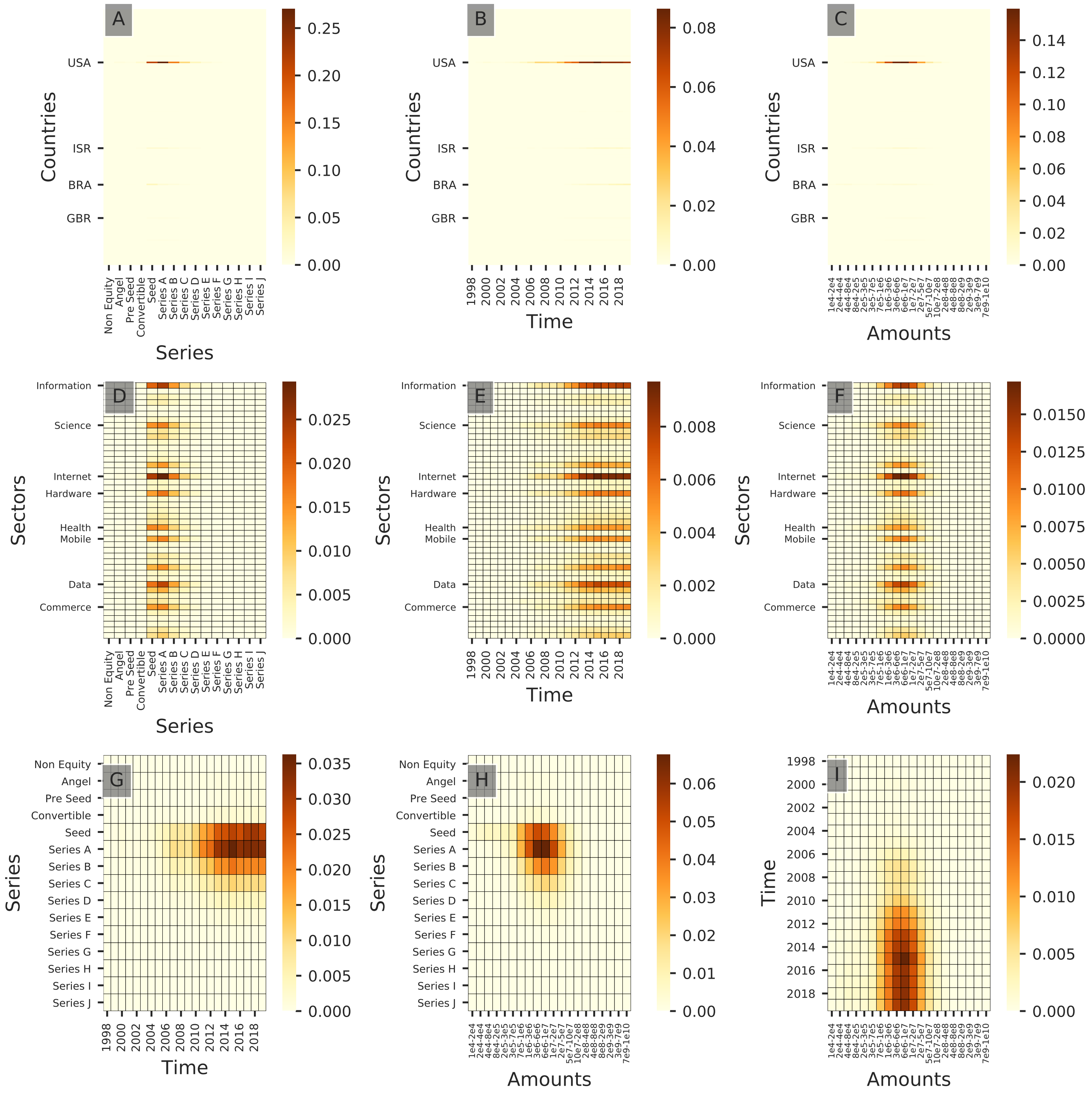

Fig S132. Cross graph interaction heatmap of community F6.

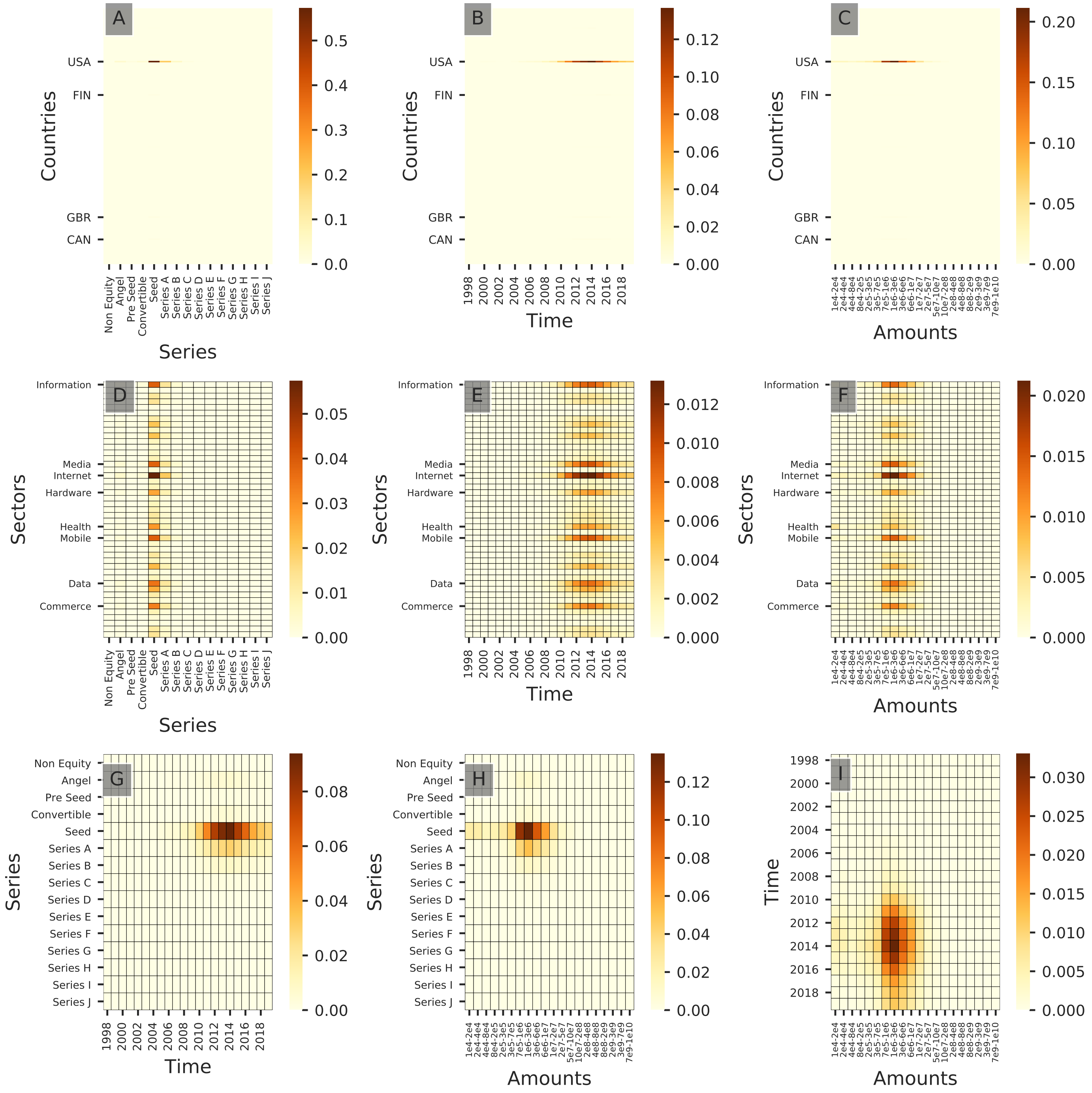

Fig S133. Cross graph interaction heatmap of community F7.

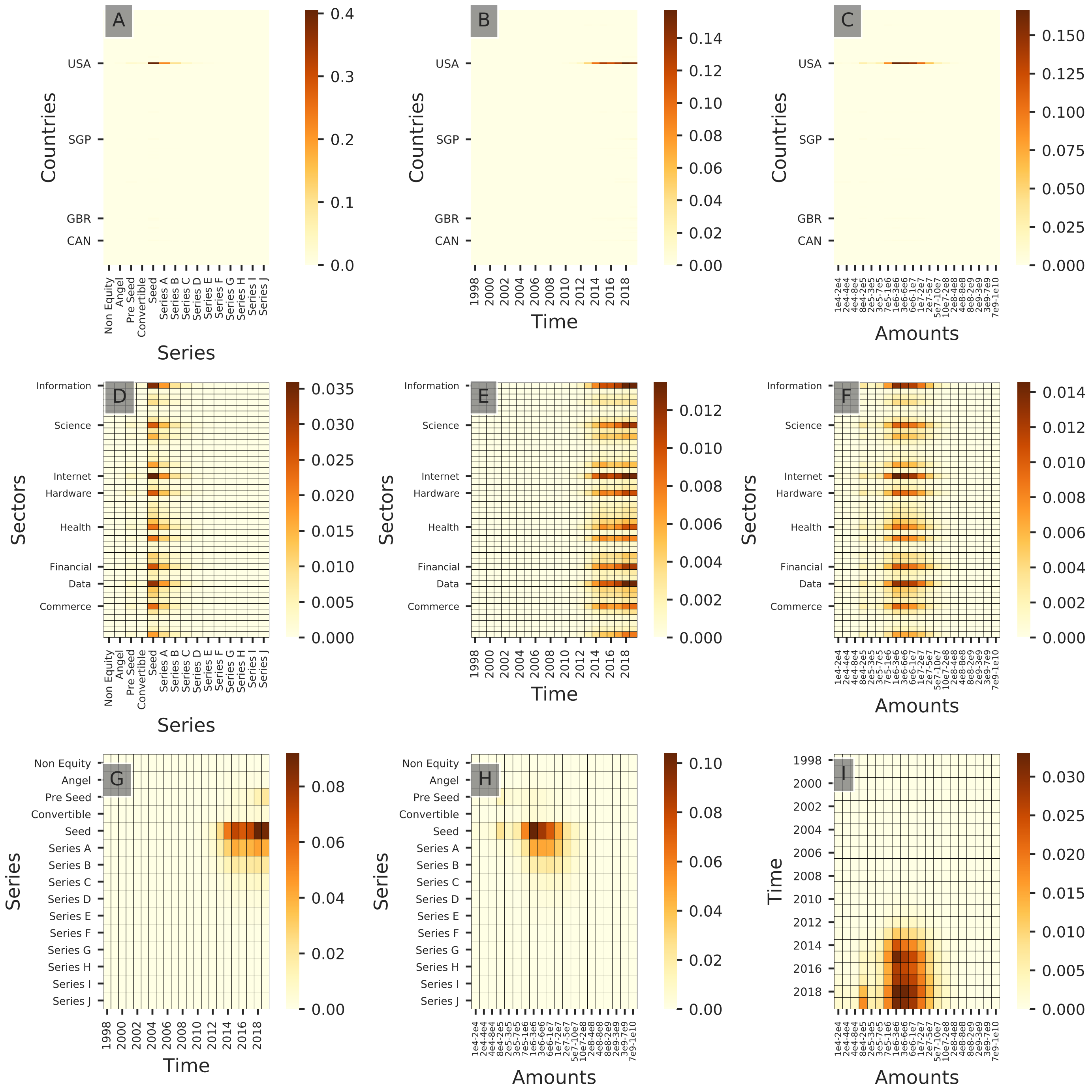

Fig S134. Cross graph interaction heatmap of community F8.

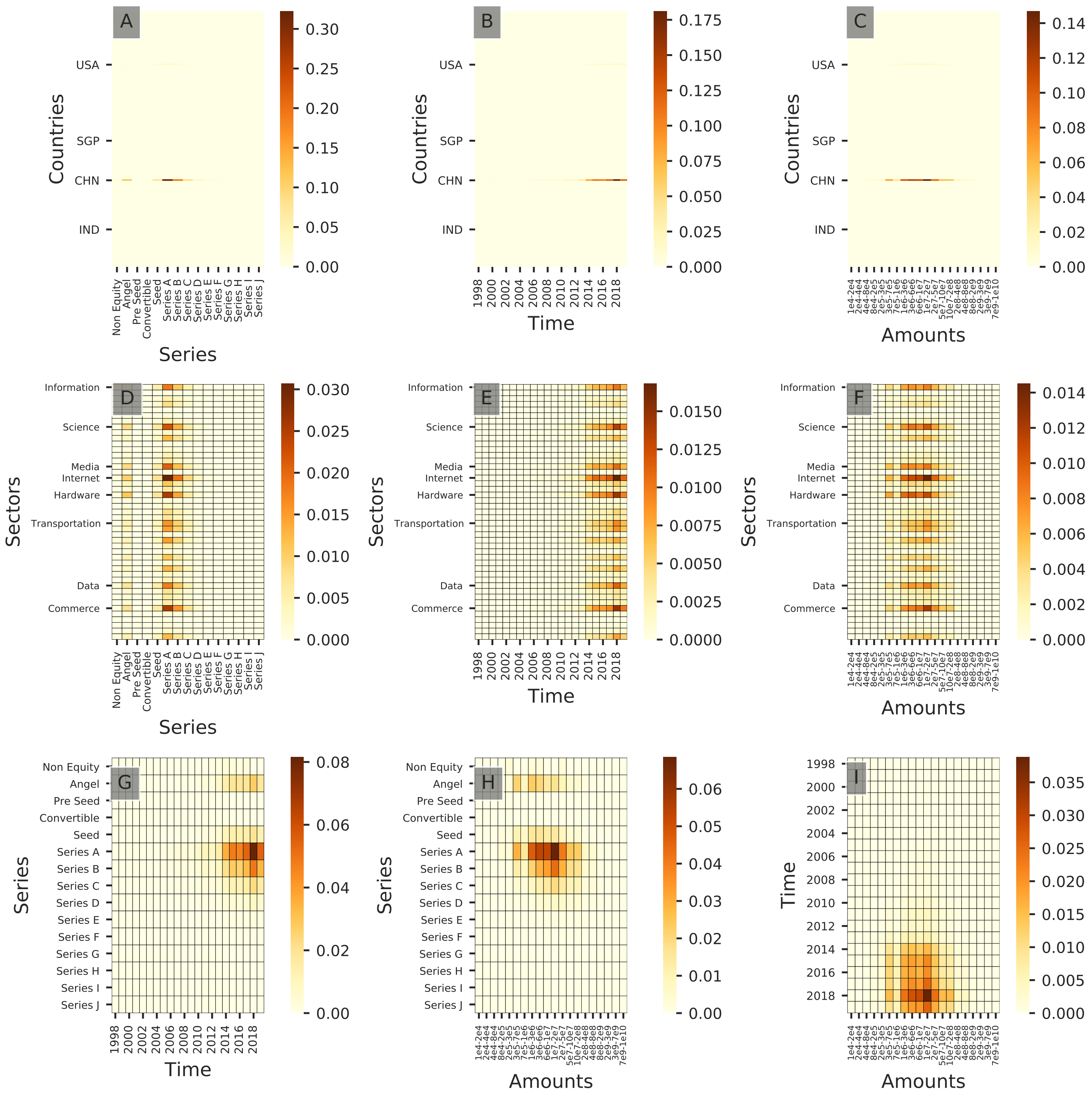

Fig S135. Cross graph interaction heatmap of community F9.

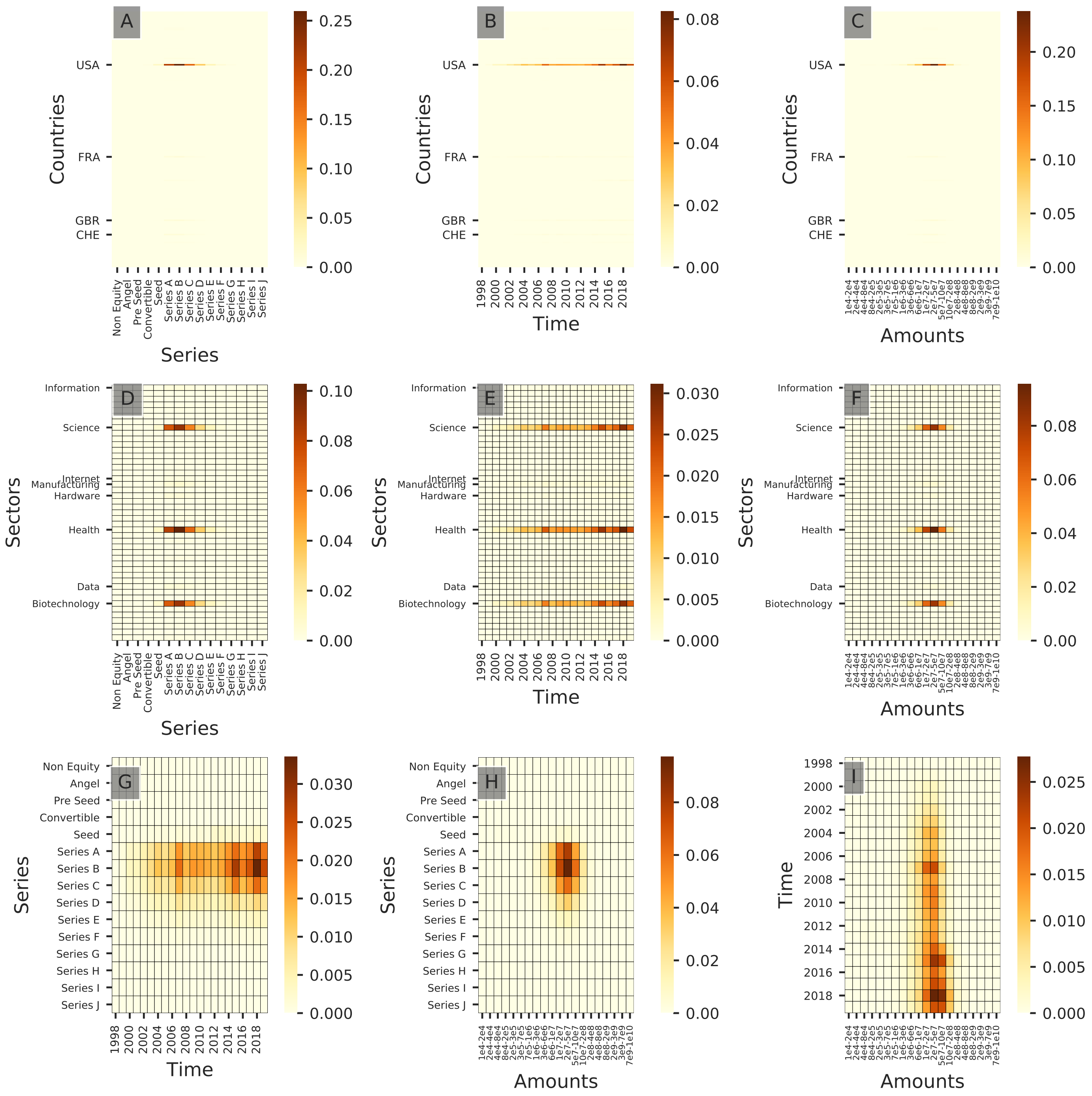

Fig S136. Cross graph interaction heatmap of community F10.

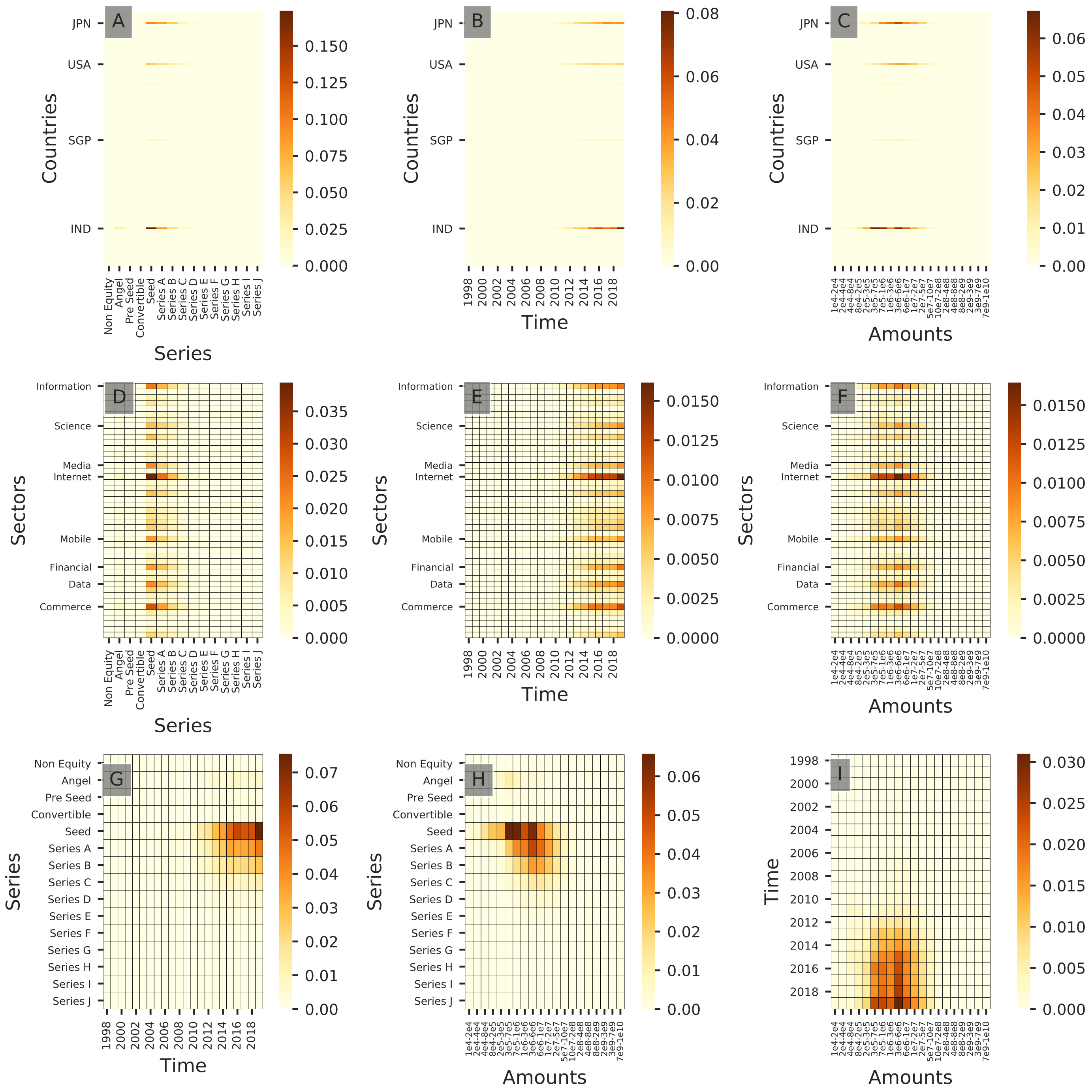

Fig S137. Cross graph interaction heatmap of community F11.

Temporal sector evolution of communities in the complete clustering

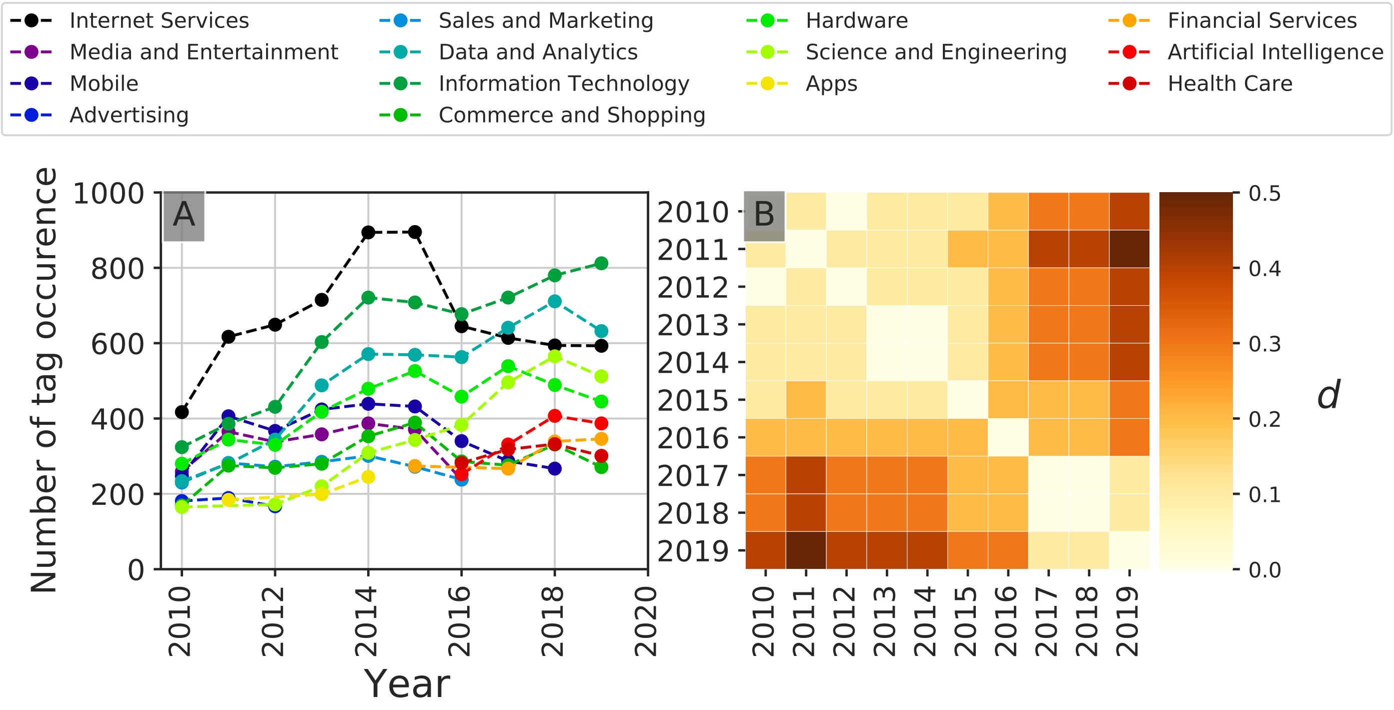

Fig S138. Temporal evolution of the investment patterns of community A0.

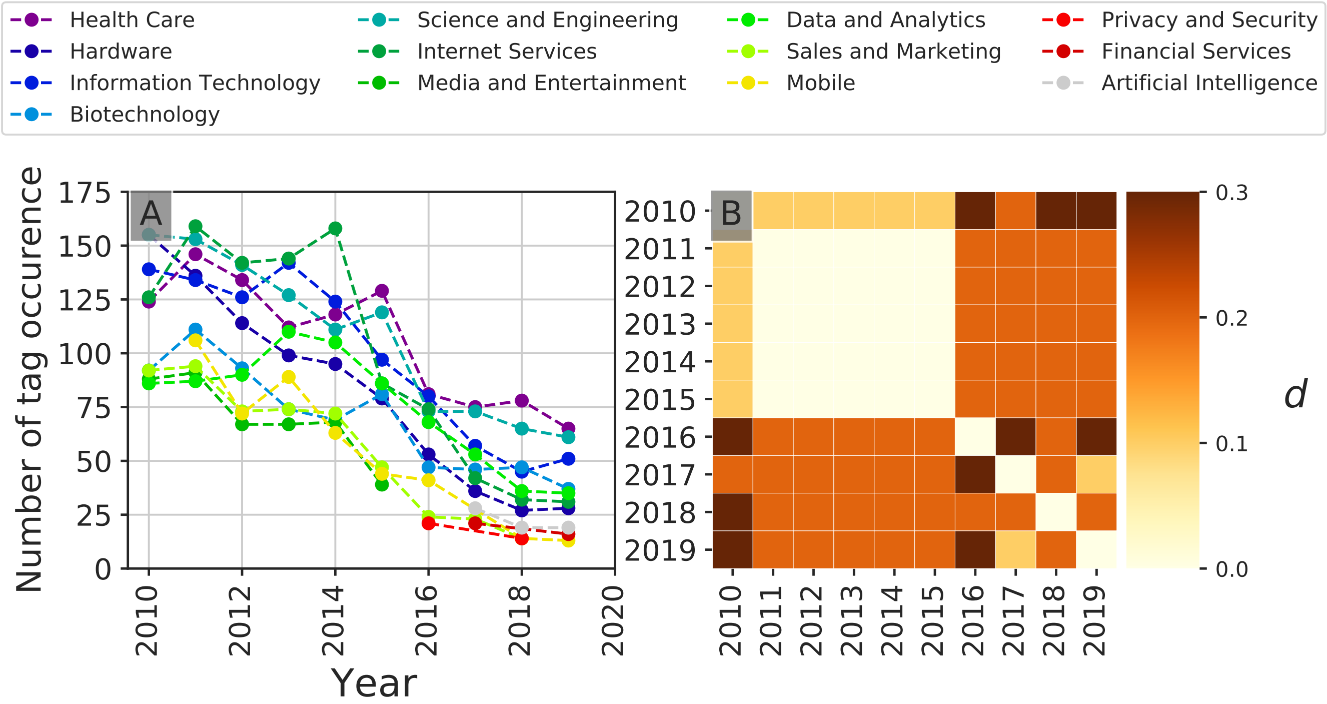

Fig S139. Temporal evolution of the investment patterns of community A1.

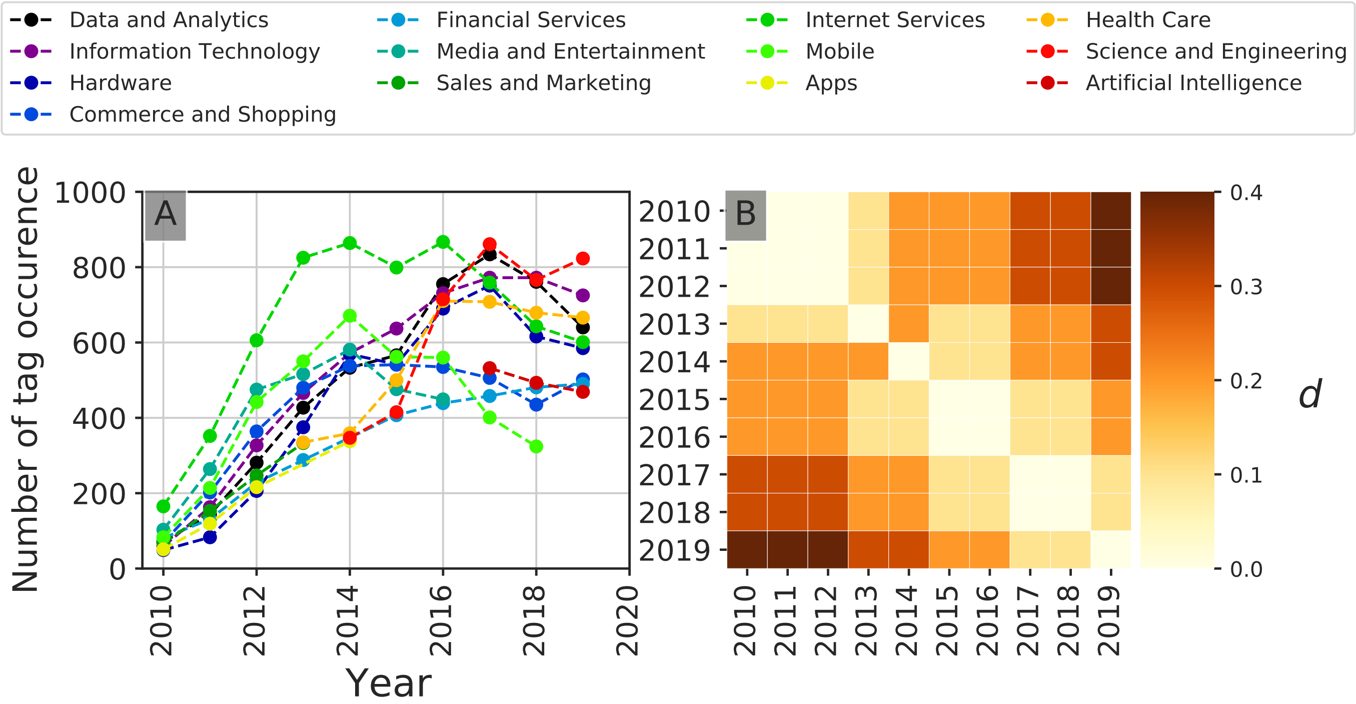

Fig S140. Temporal evolution of the investment patterns of community A2.

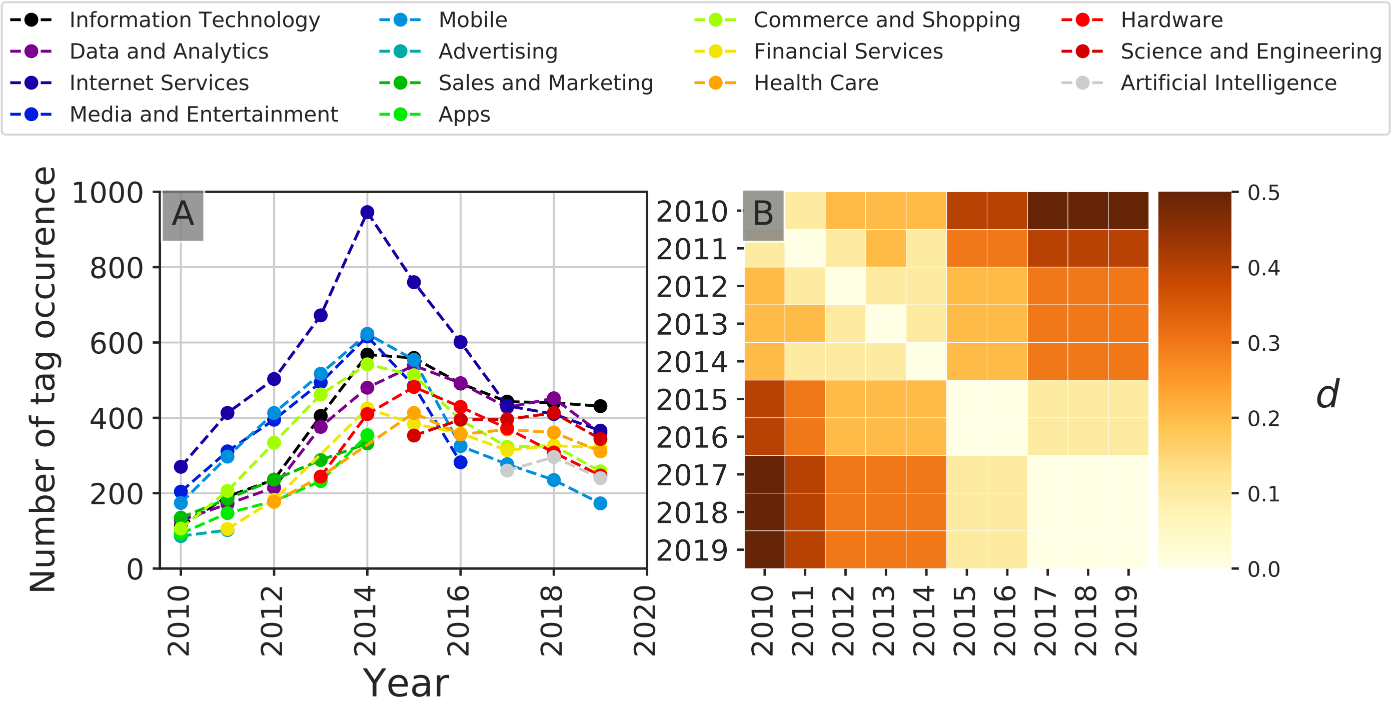

Fig S141. Temporal evolution of the investment patterns of community A3.

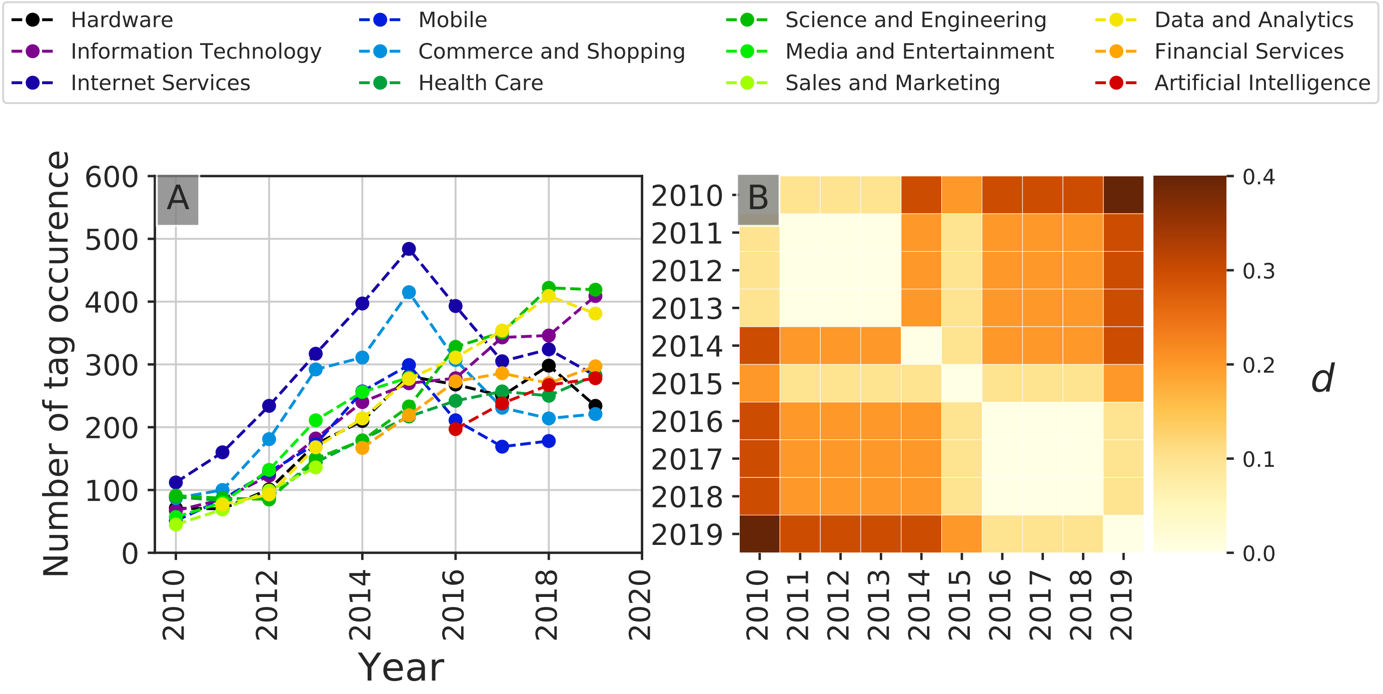

Fig S142. Temporal evolution of the investment patterns of community A4.

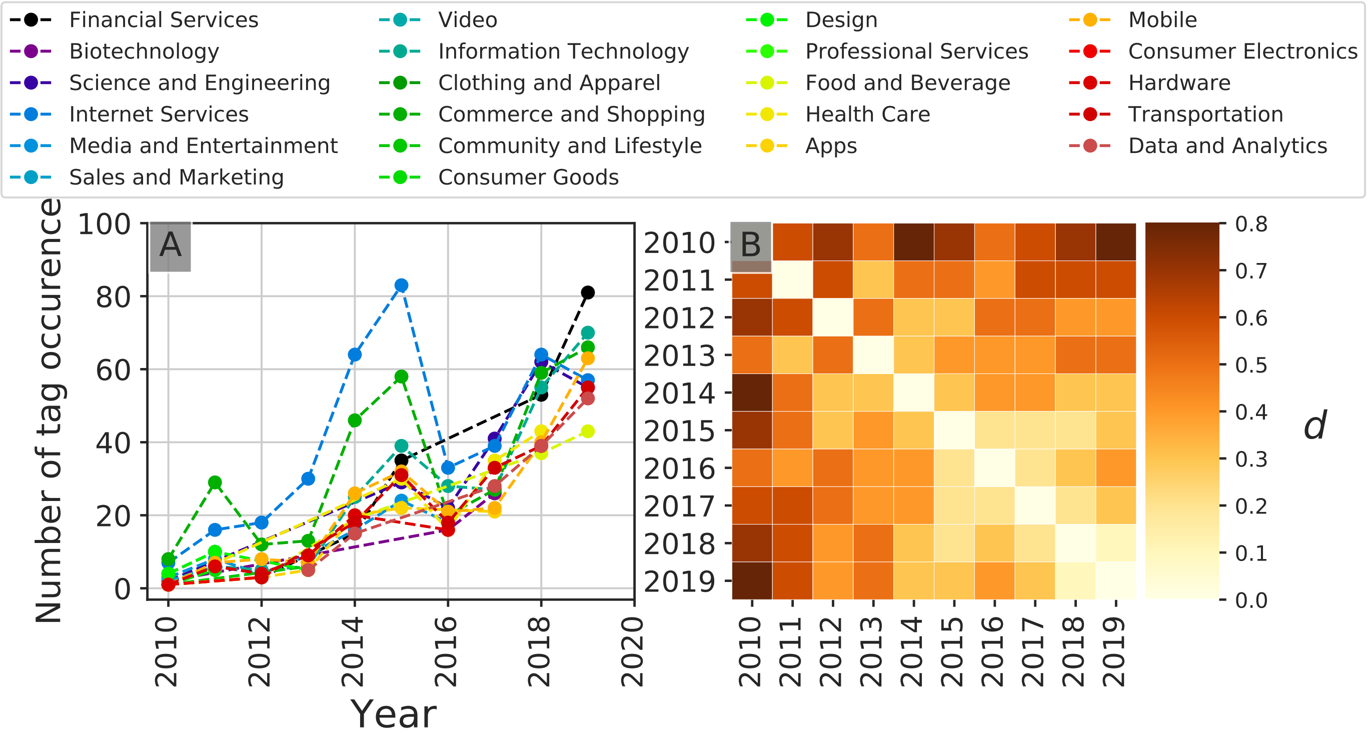

Fig S143. Temporal evolution of the investment patterns of community A5.

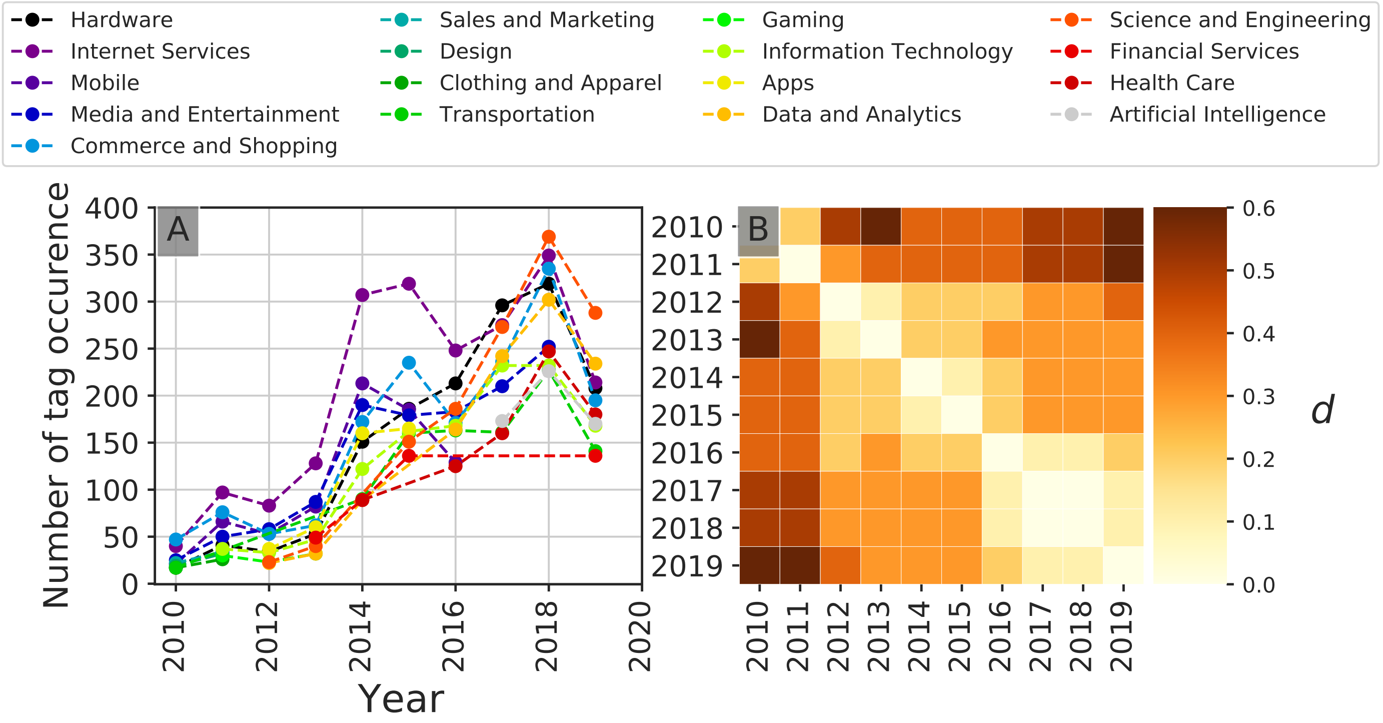

Fig S144. Temporal evolution of the investment patterns of community A6.

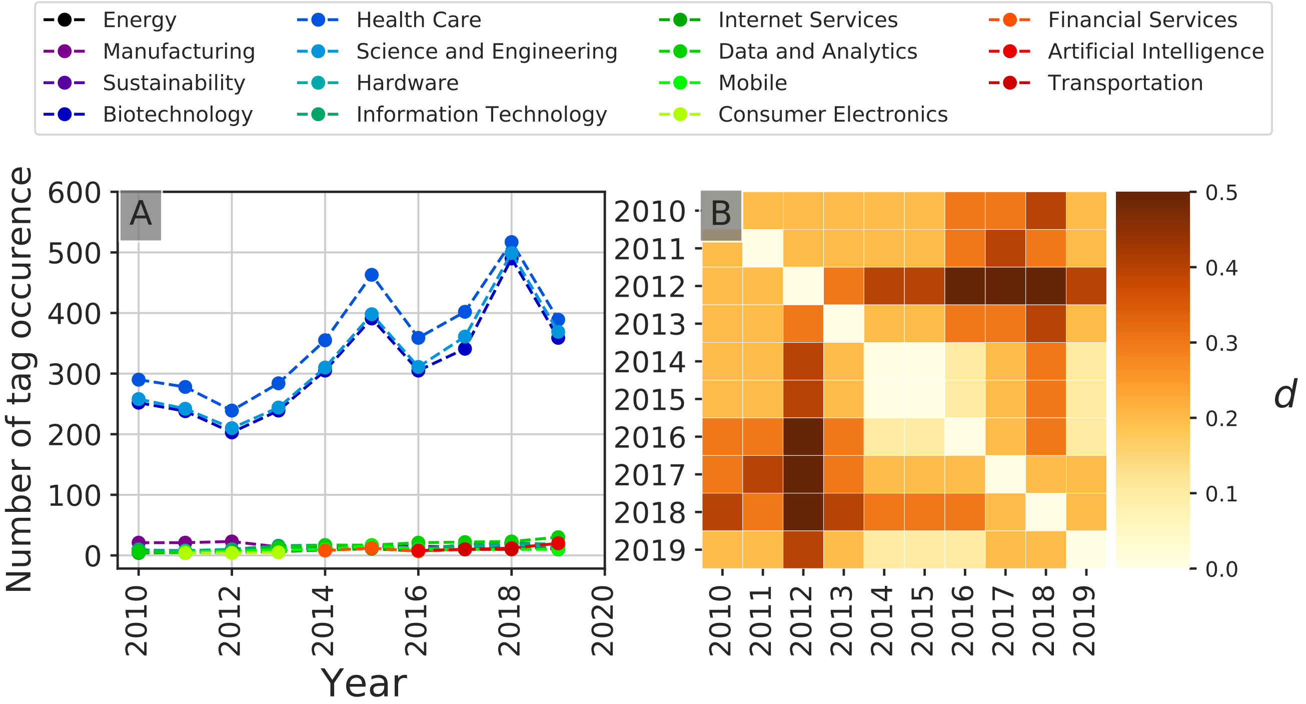

Fig S145. Temporal evolution of the investment patterns of community A7.

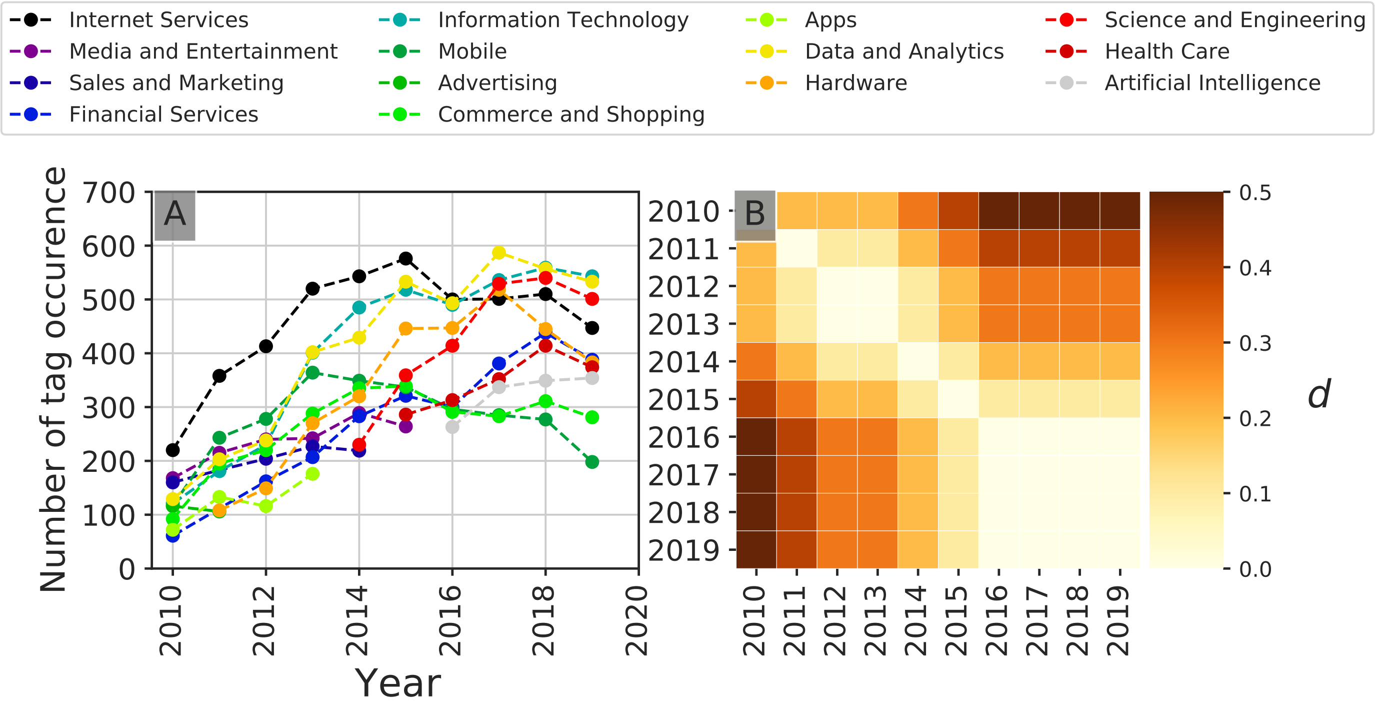

Fig S146. Temporal evolution of the investment patterns of community A8.

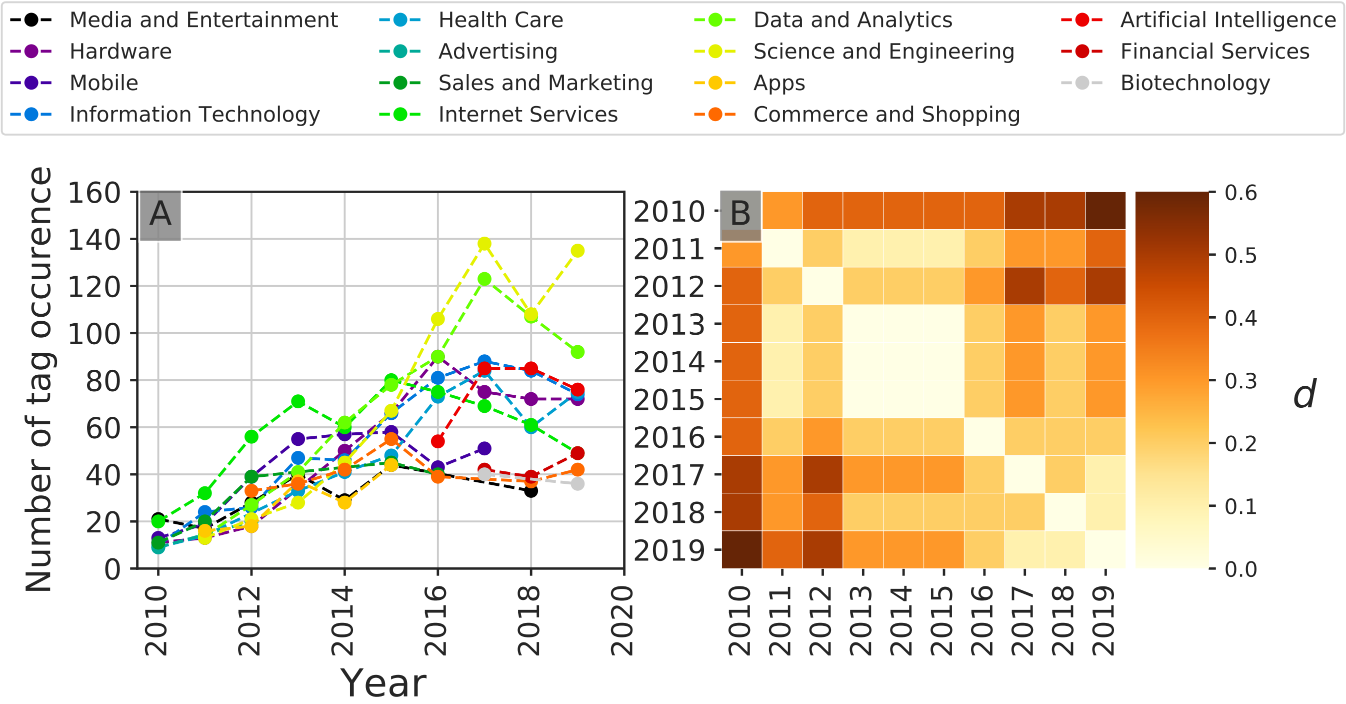

Fig S147. Temporal evolution of the investment patterns of community A9.

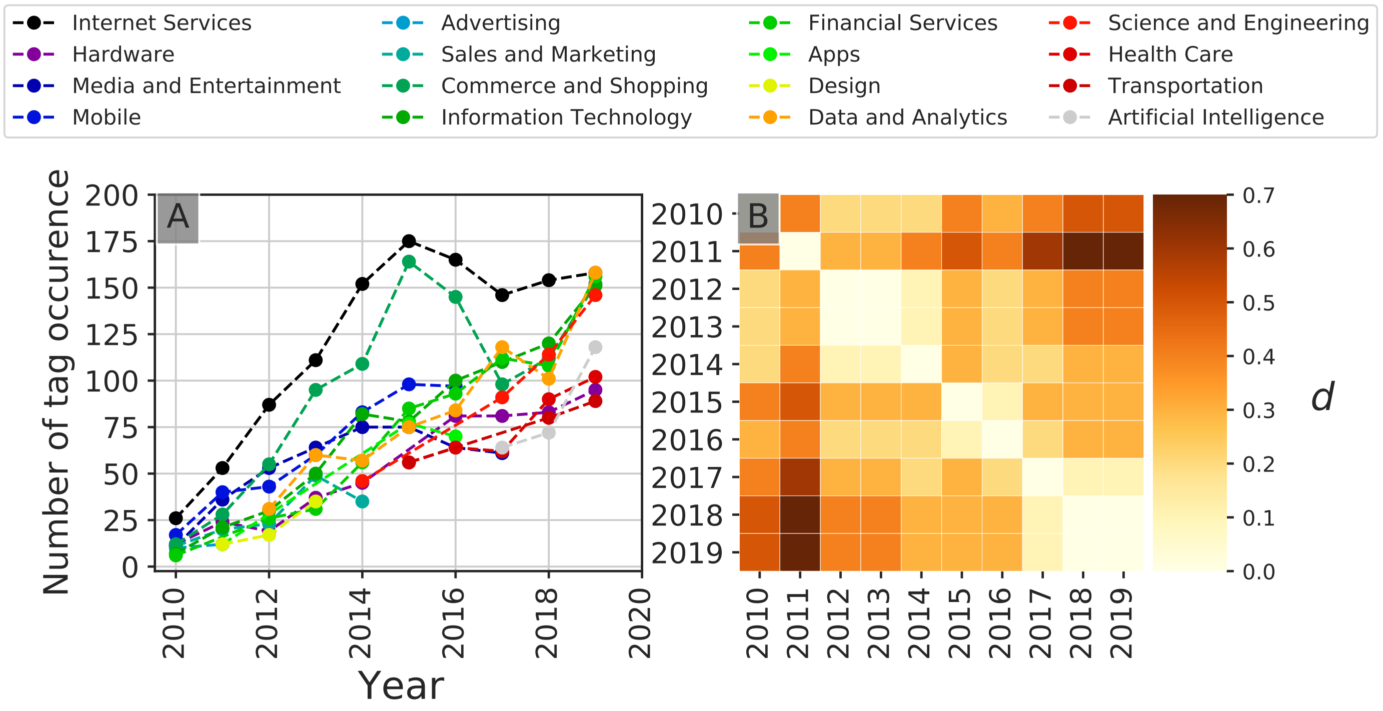

Fig S148. Temporal evolution of the investment patterns of community A10.

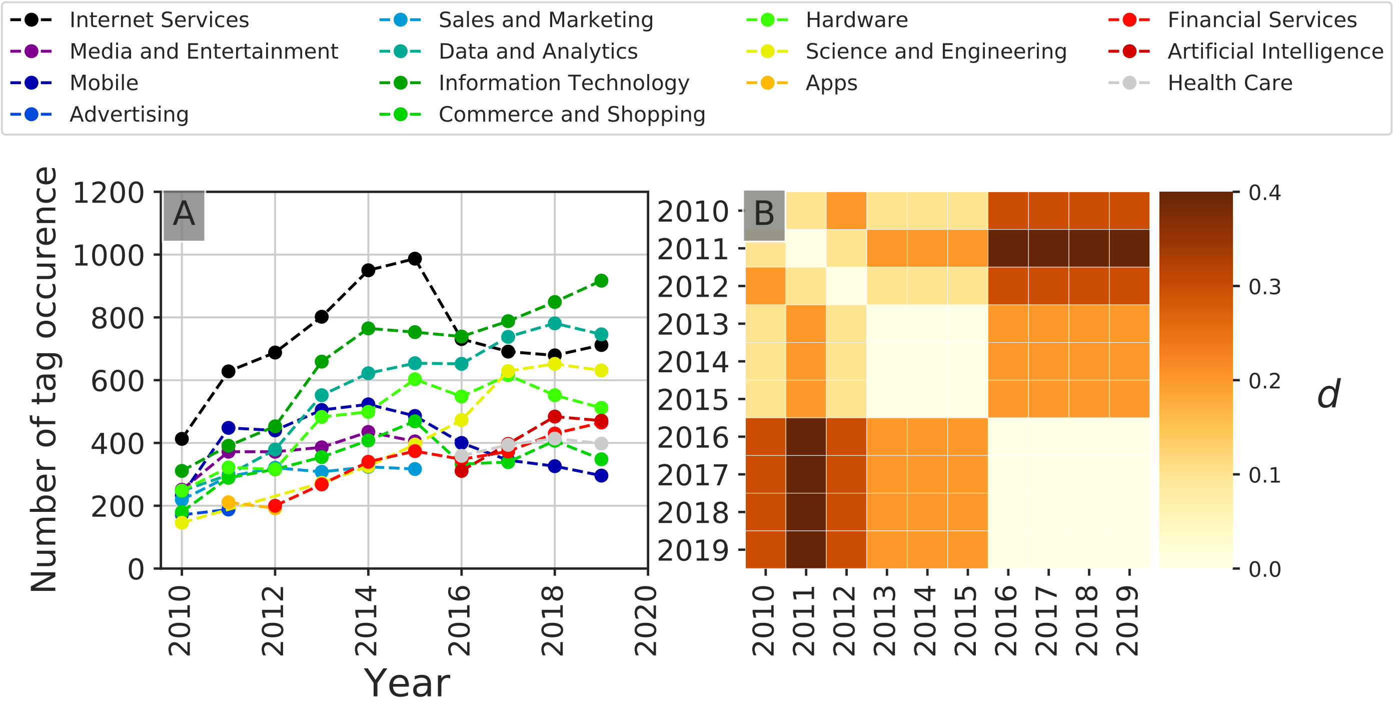

Fig S149. Temporal evolution of the investment patterns of community B0.

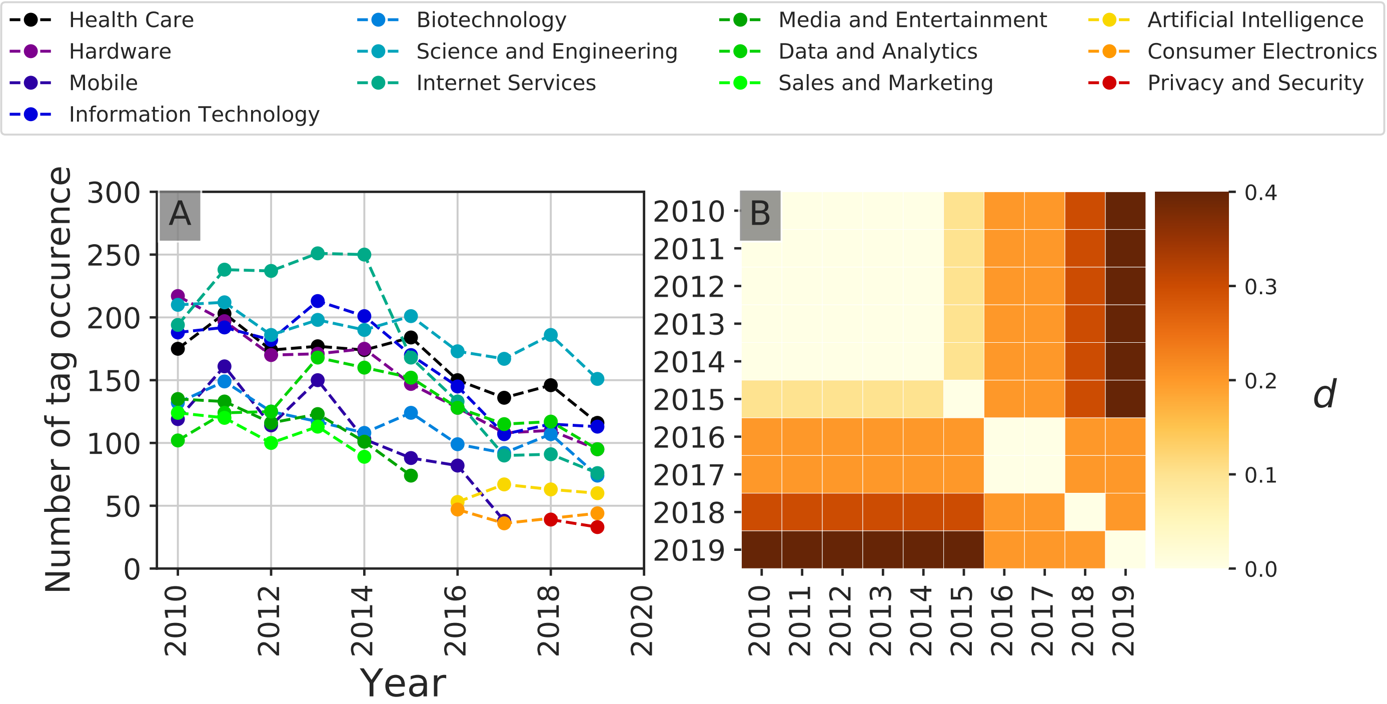

Fig S150. Temporal evolution of the investment patterns of community B1.

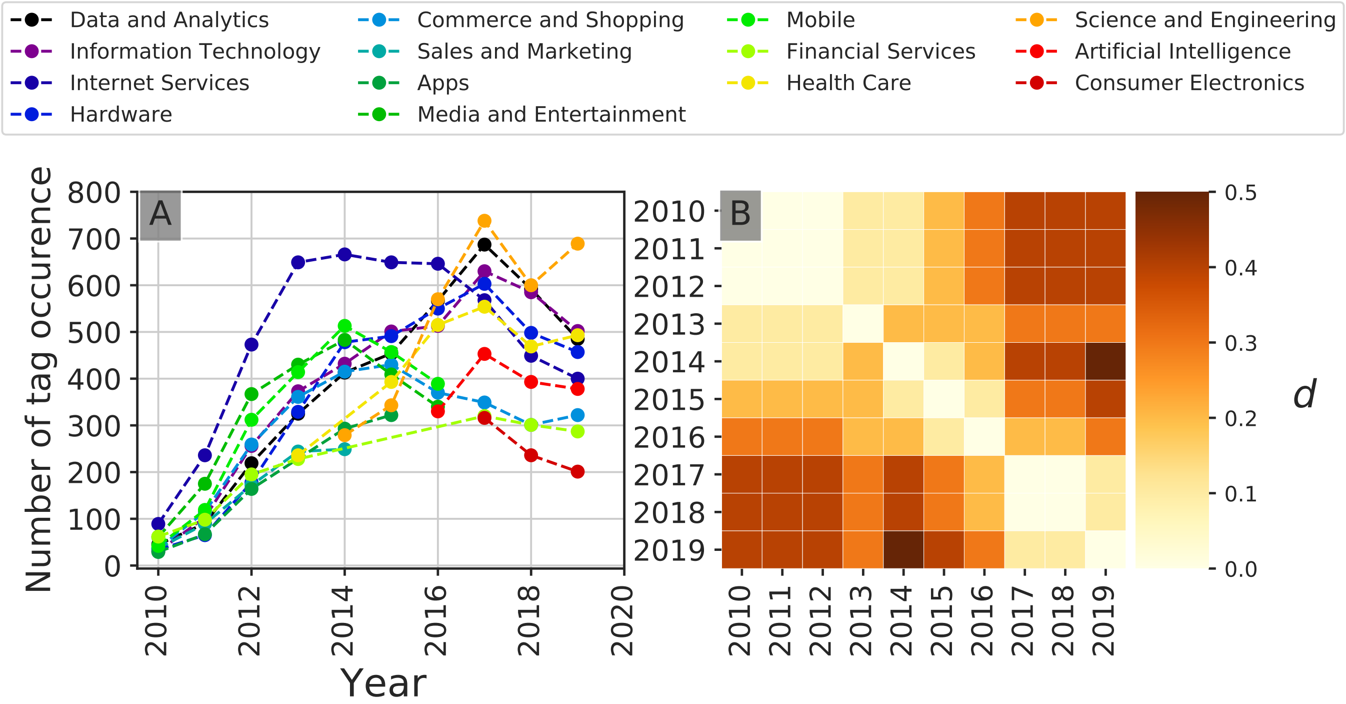

Fig S151. Temporal evolution of the investment patterns of community B2.

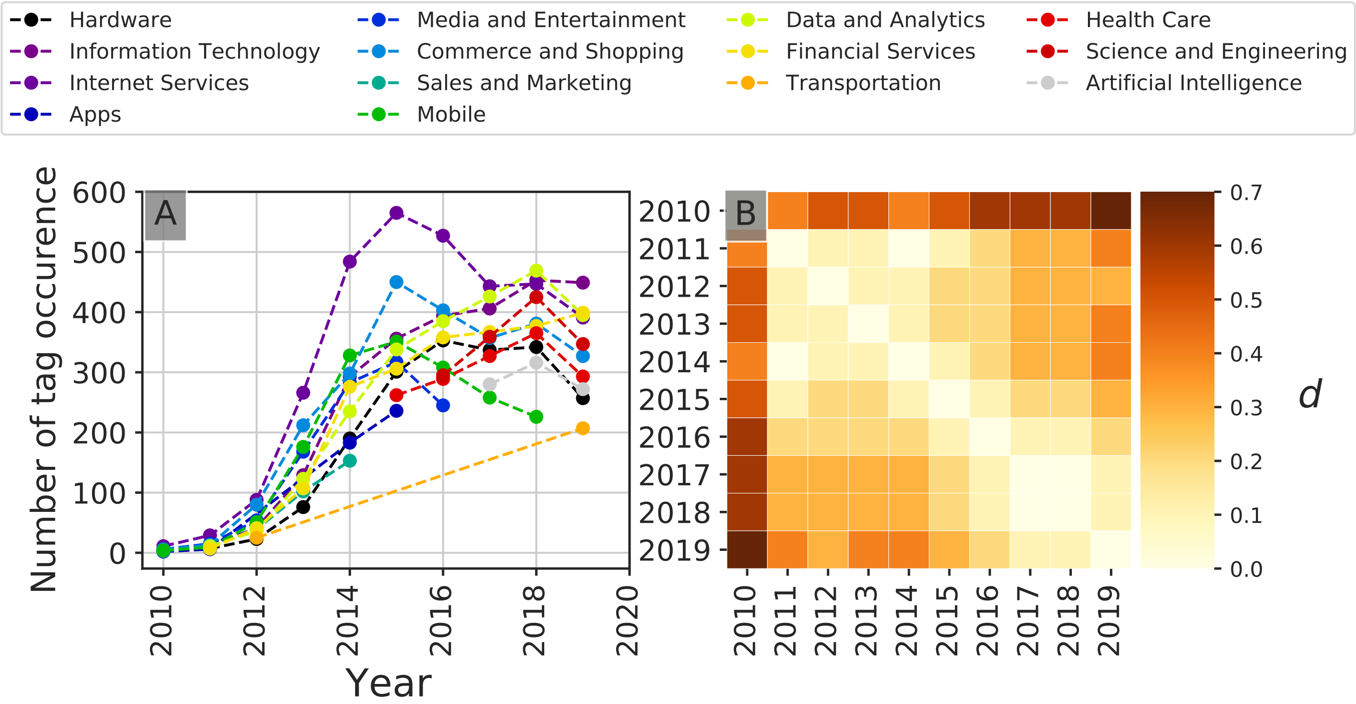

Fig S152. Temporal evolution of the investment patterns of community B3.

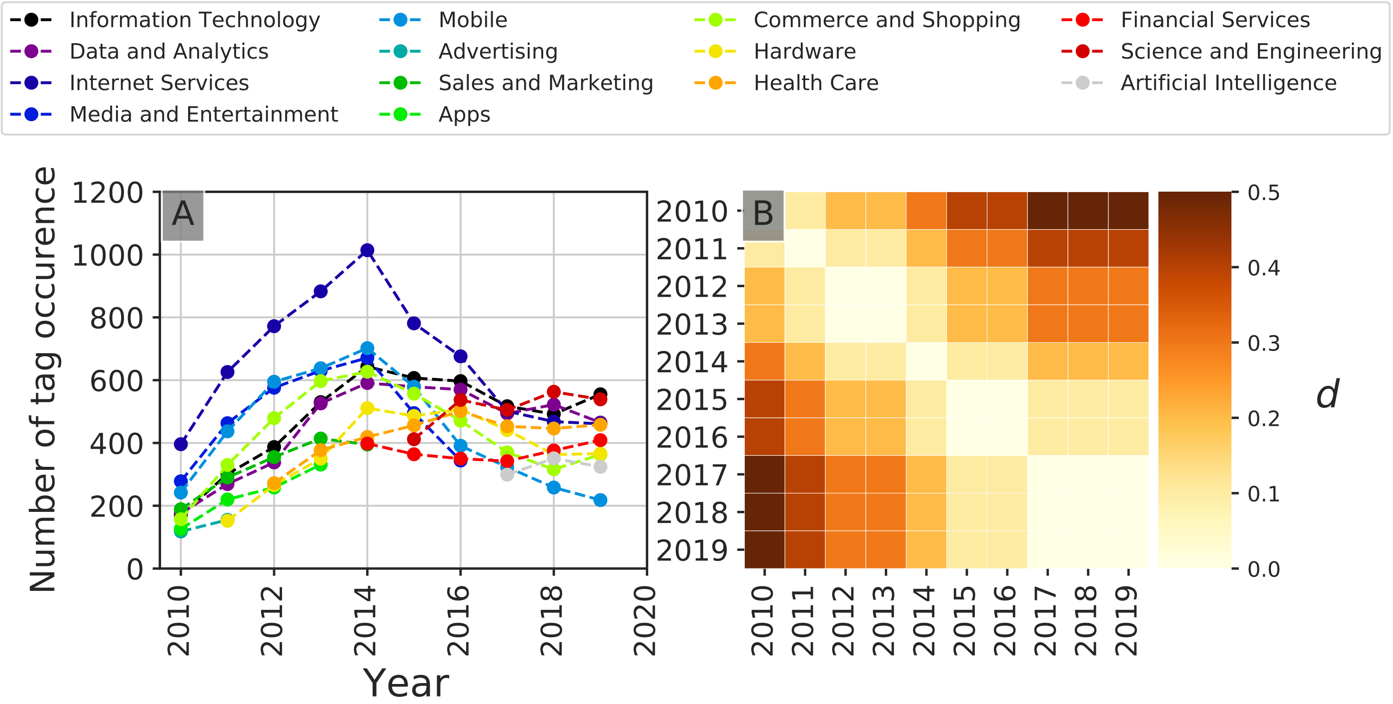

Fig S153. Temporal evolution of the investment patterns of community B4.

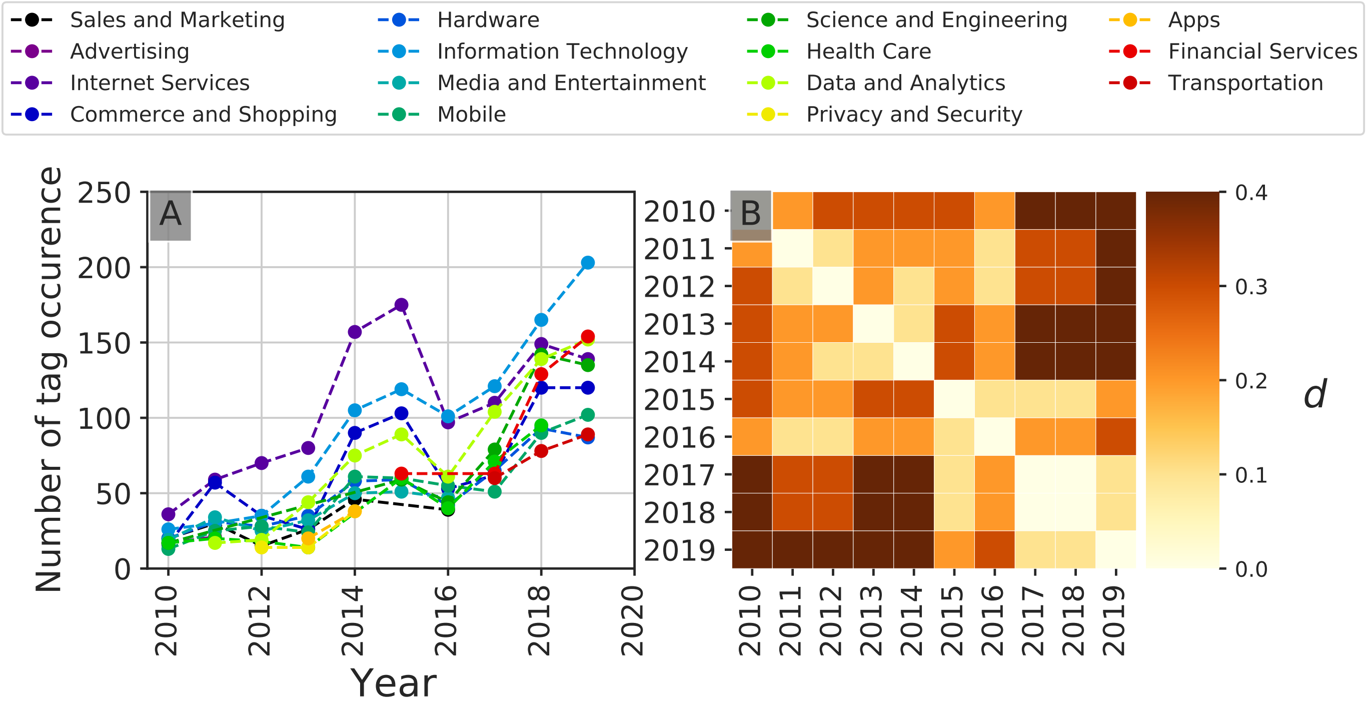

Fig S154. Temporal evolution of the investment patterns of community B5.

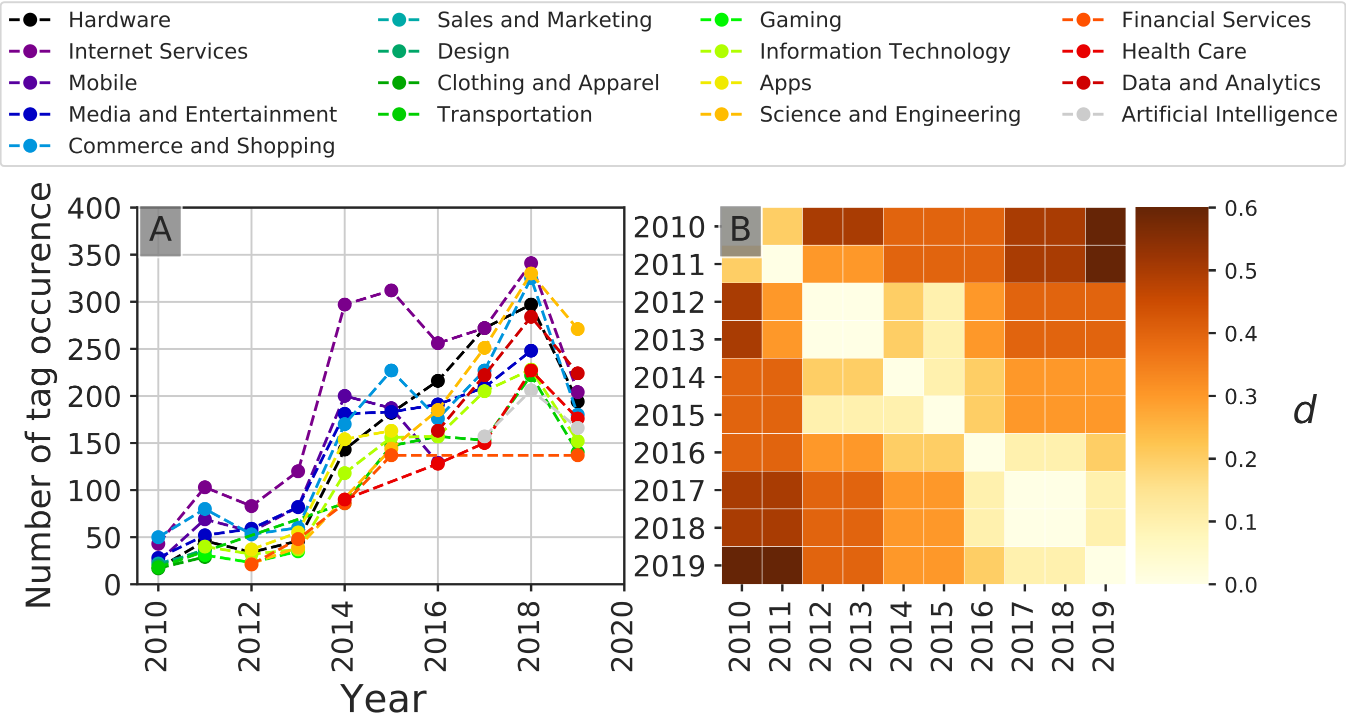

Fig S155. Temporal evolution of the investment patterns of community B6.

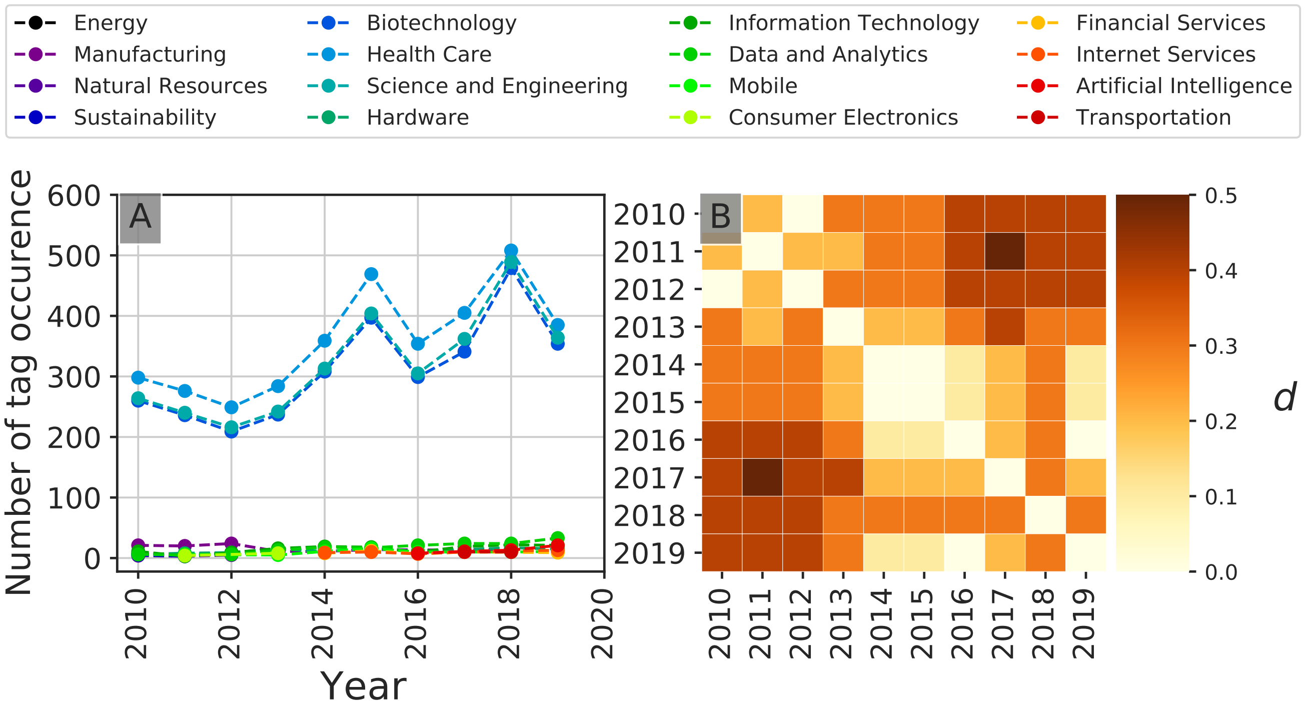

Fig S156. Temporal evolution of the investment patterns of community B7.

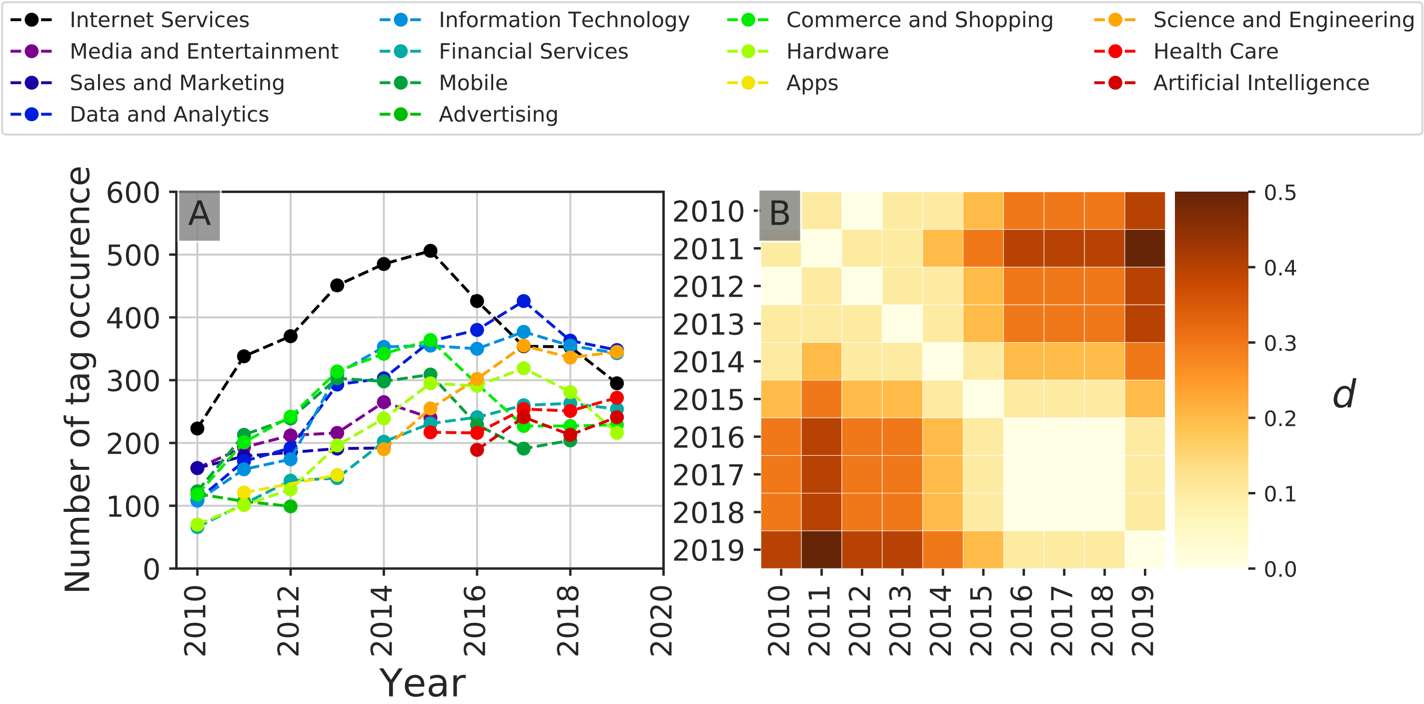

Fig S157. Temporal evolution of the investment patterns of community B8.

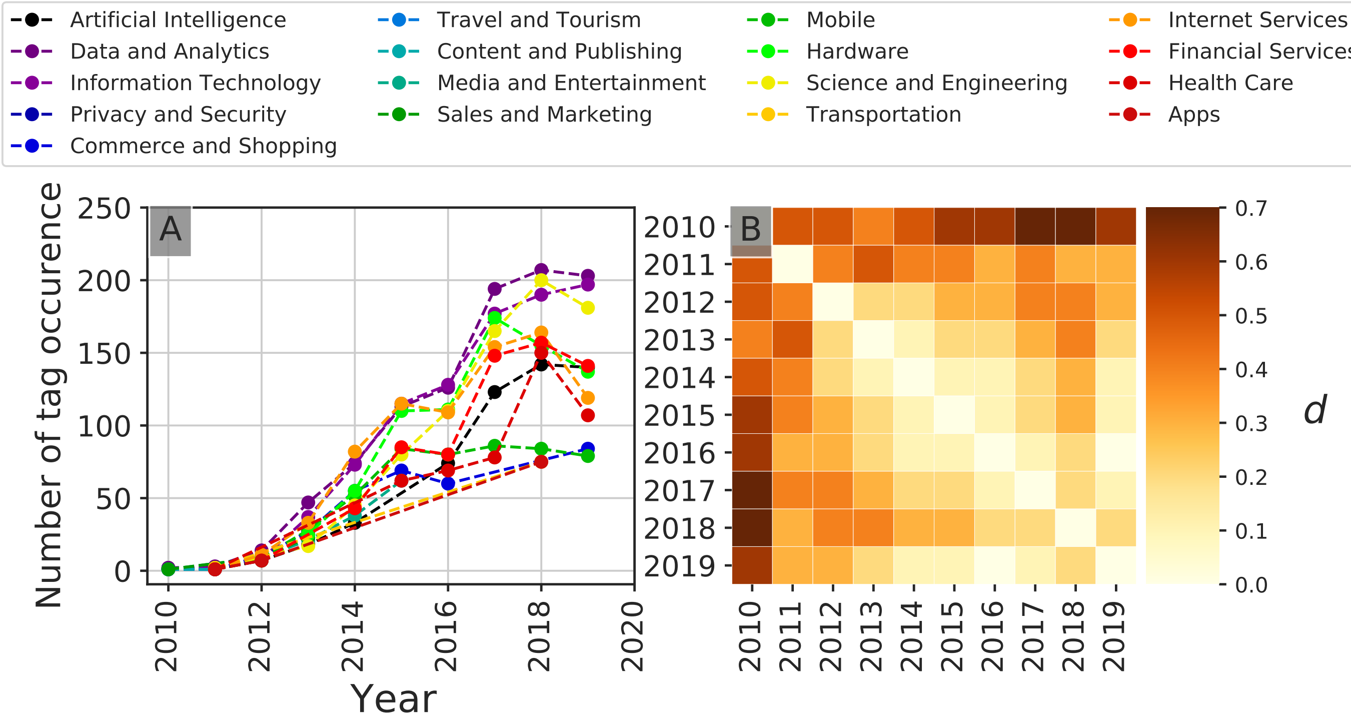

Fig S158. Temporal evolution of the investment patterns of community B9.

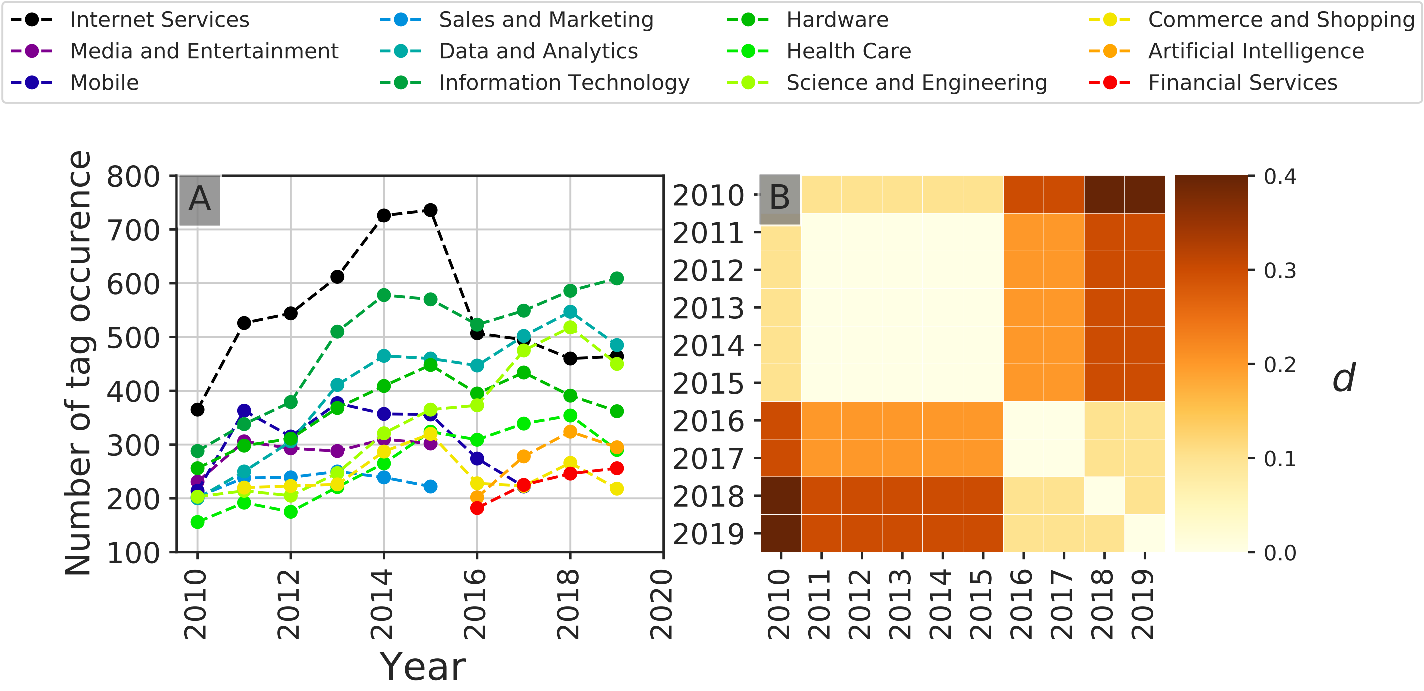

Fig S159. Temporal evolution of the investment patterns of community C0.

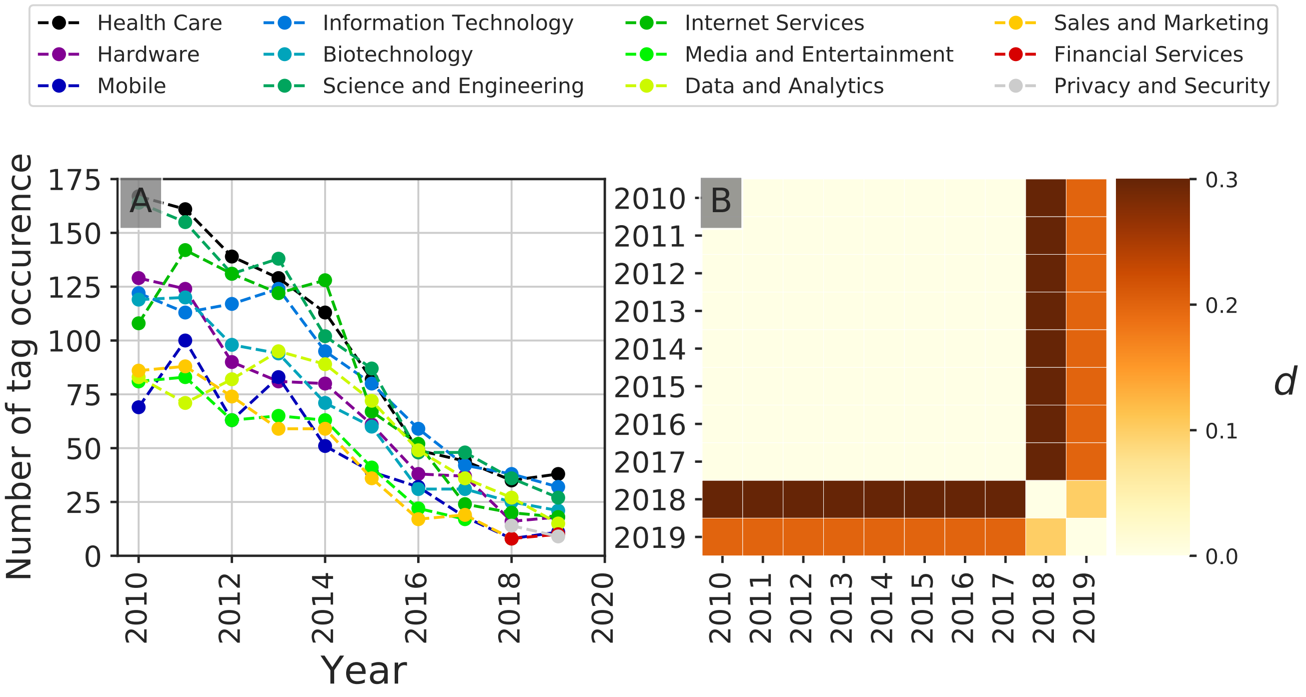

Fig S160. Temporal evolution of the investment patterns of community C1.

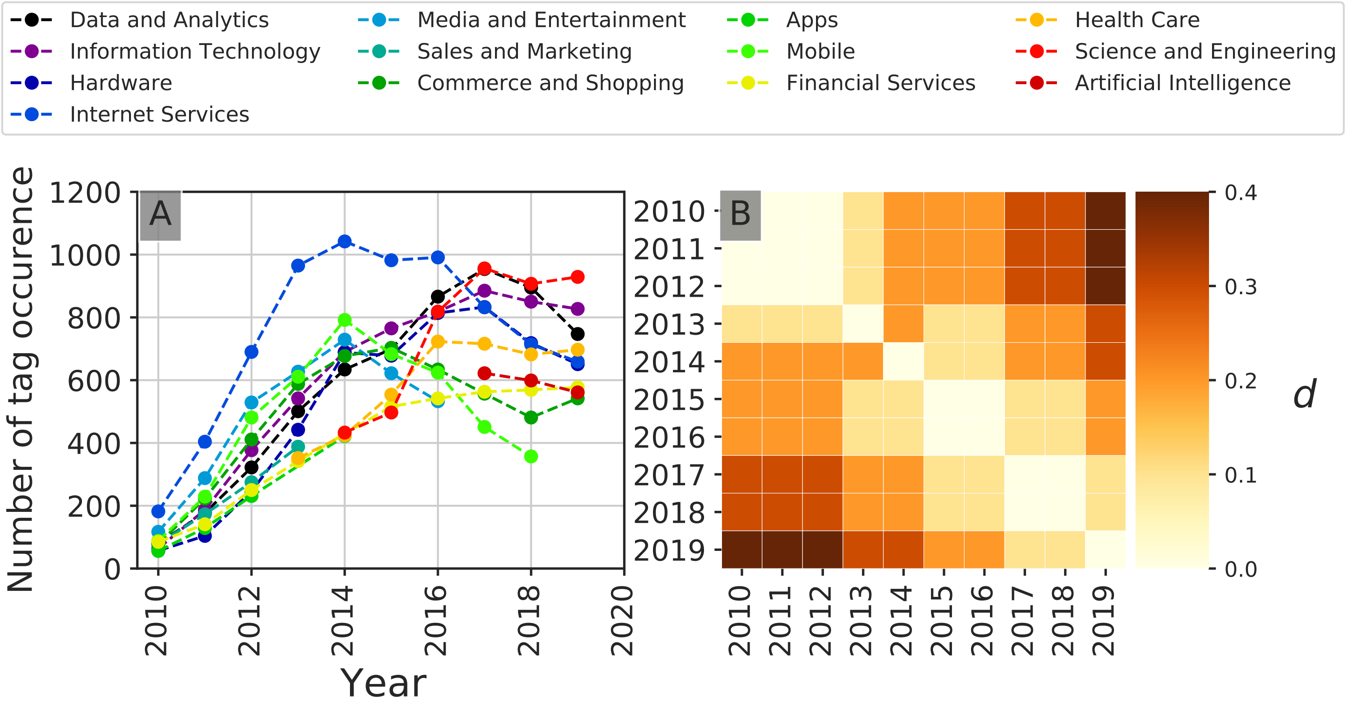

Fig S161. Temporal evolution of the investment patterns of community C2.

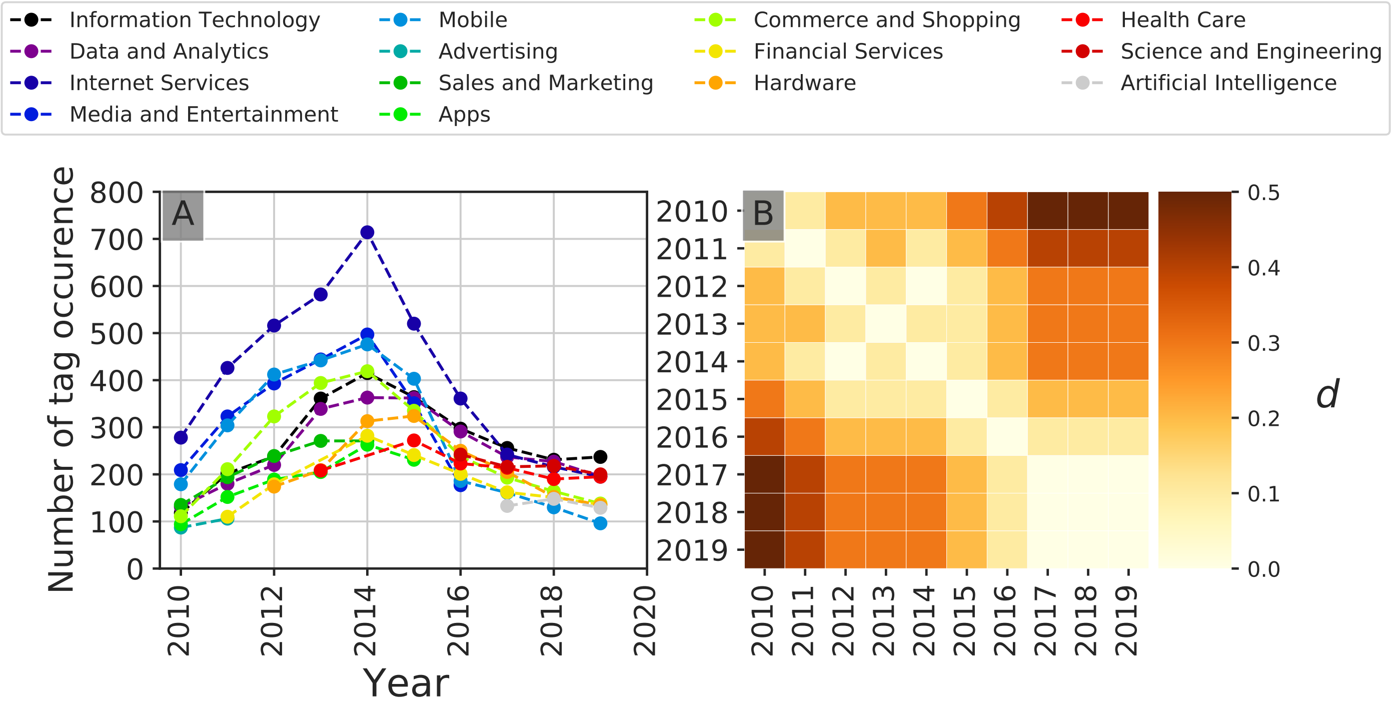

Fig S162. Temporal evolution of the investment patterns of community C3.

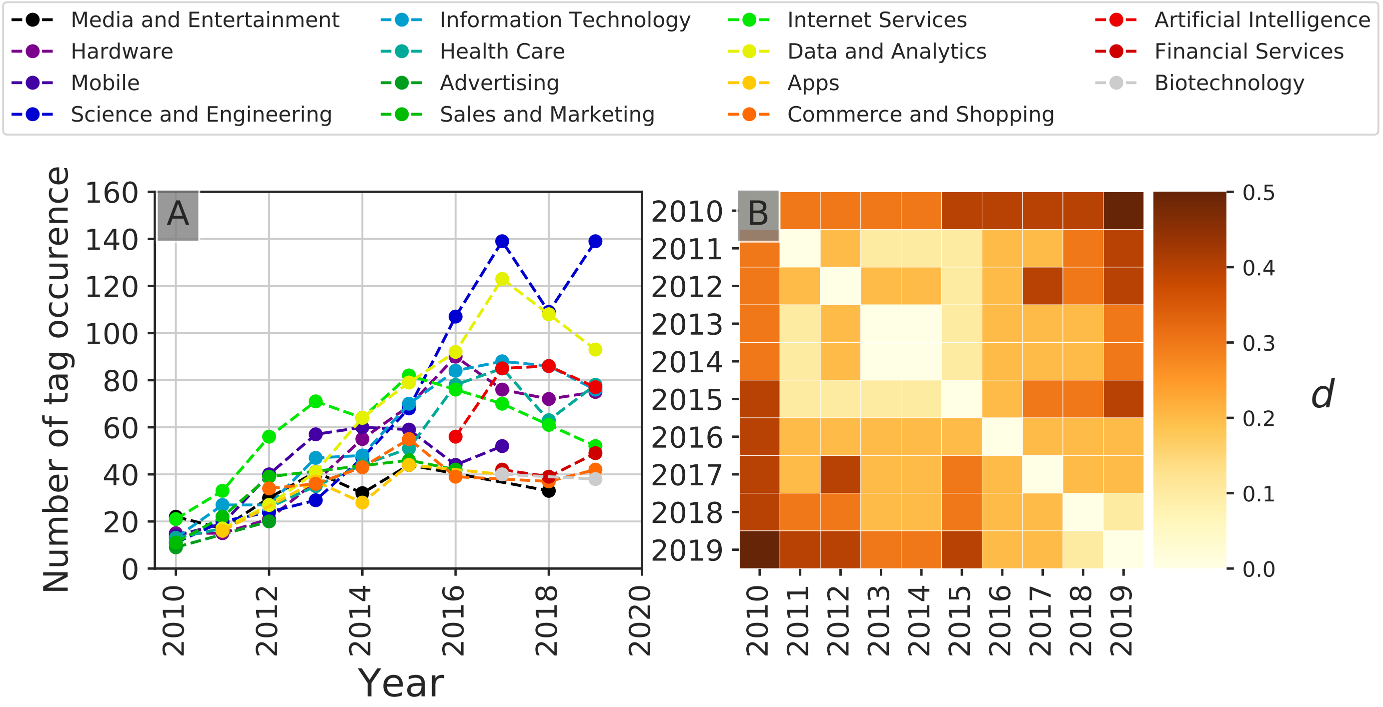

Fig S163. Temporal evolution of the investment patterns of community C4.

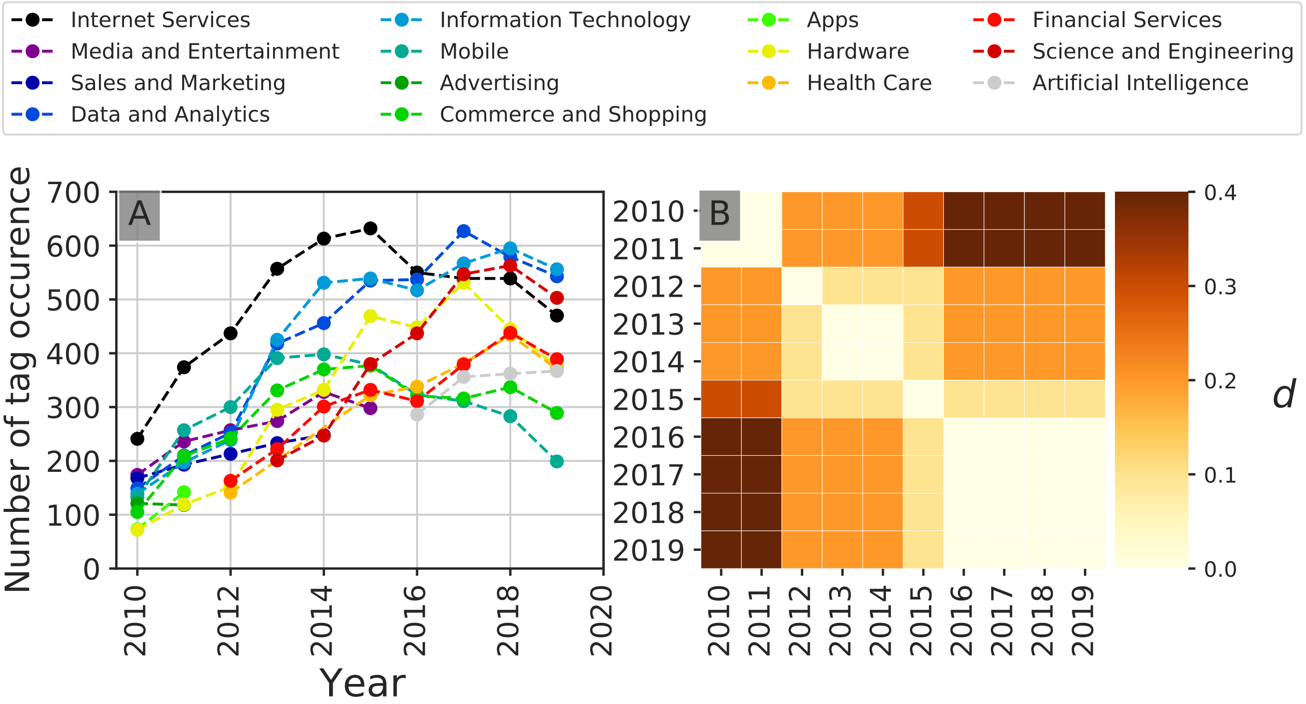

Fig S164. Temporal evolution of the investment patterns of community C5.

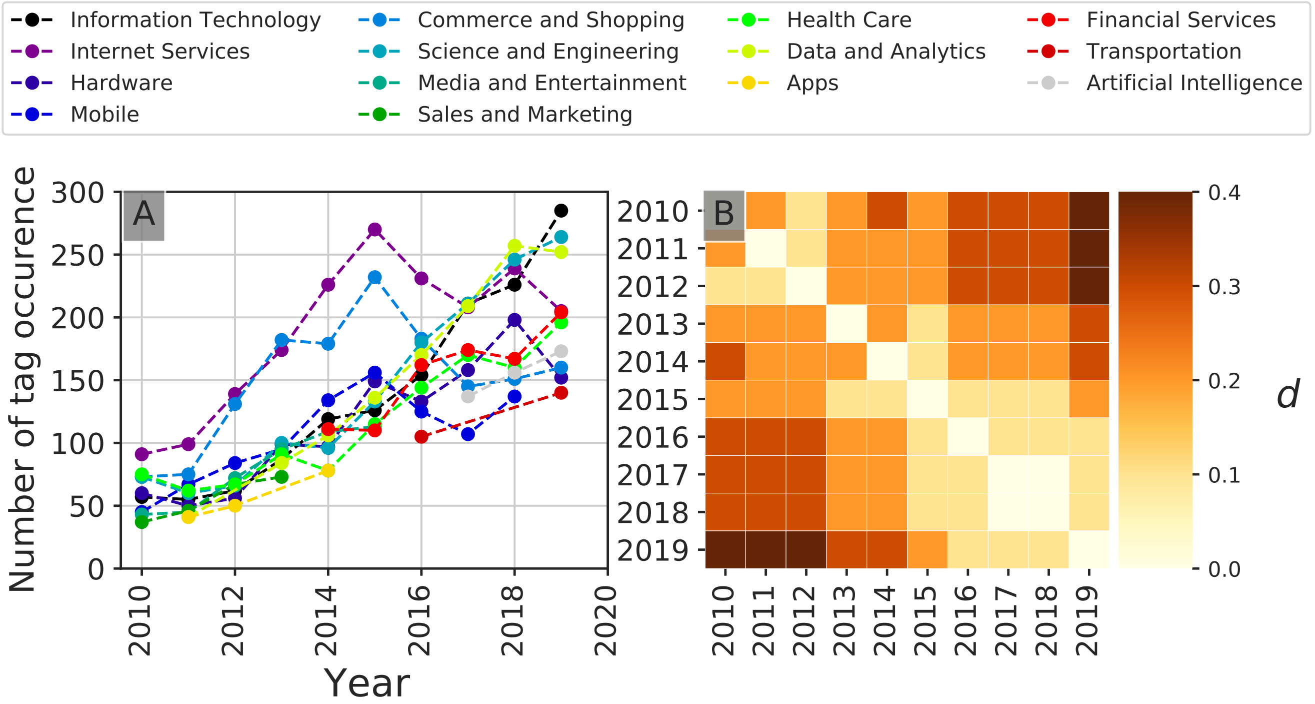

Fig S165. Temporal evolution of the investment patterns of community C6.

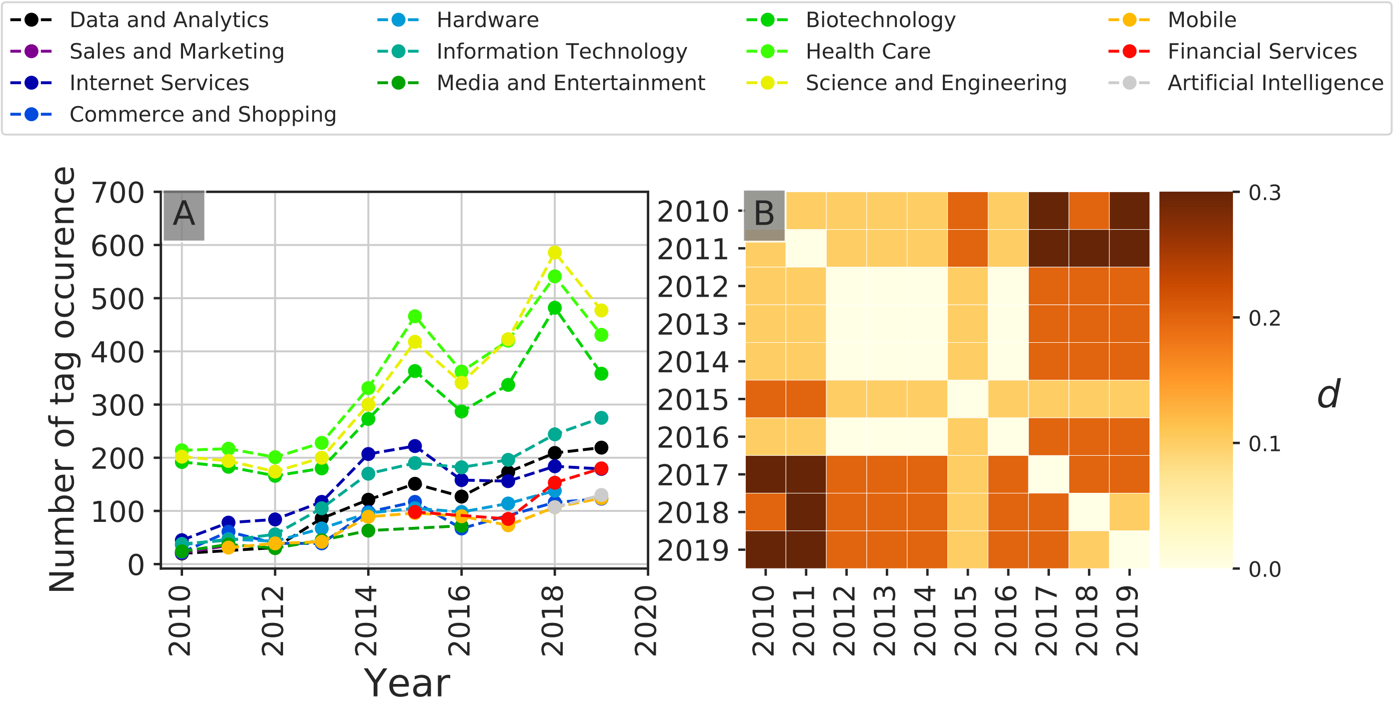

Fig S166. Temporal evolution of the investment patterns of community C7.

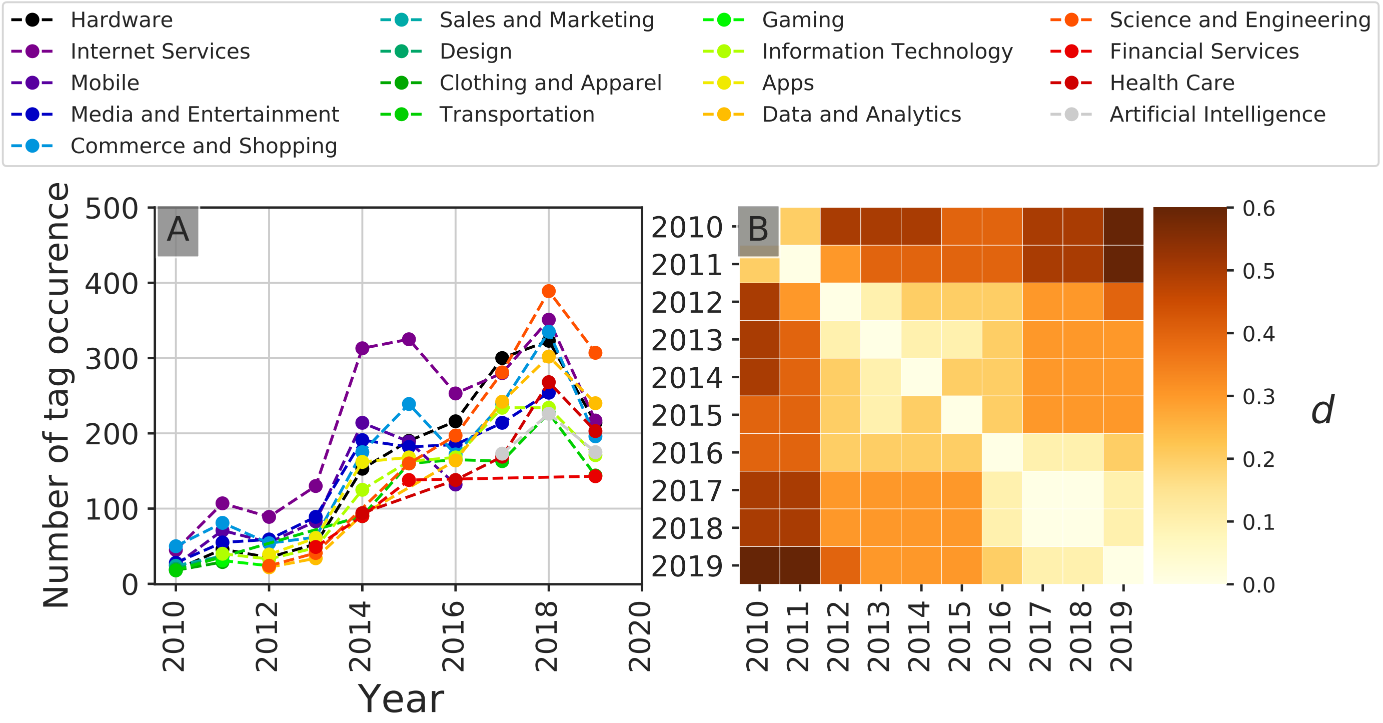

Fig S167. Temporal evolution of the investment patterns of community C8.

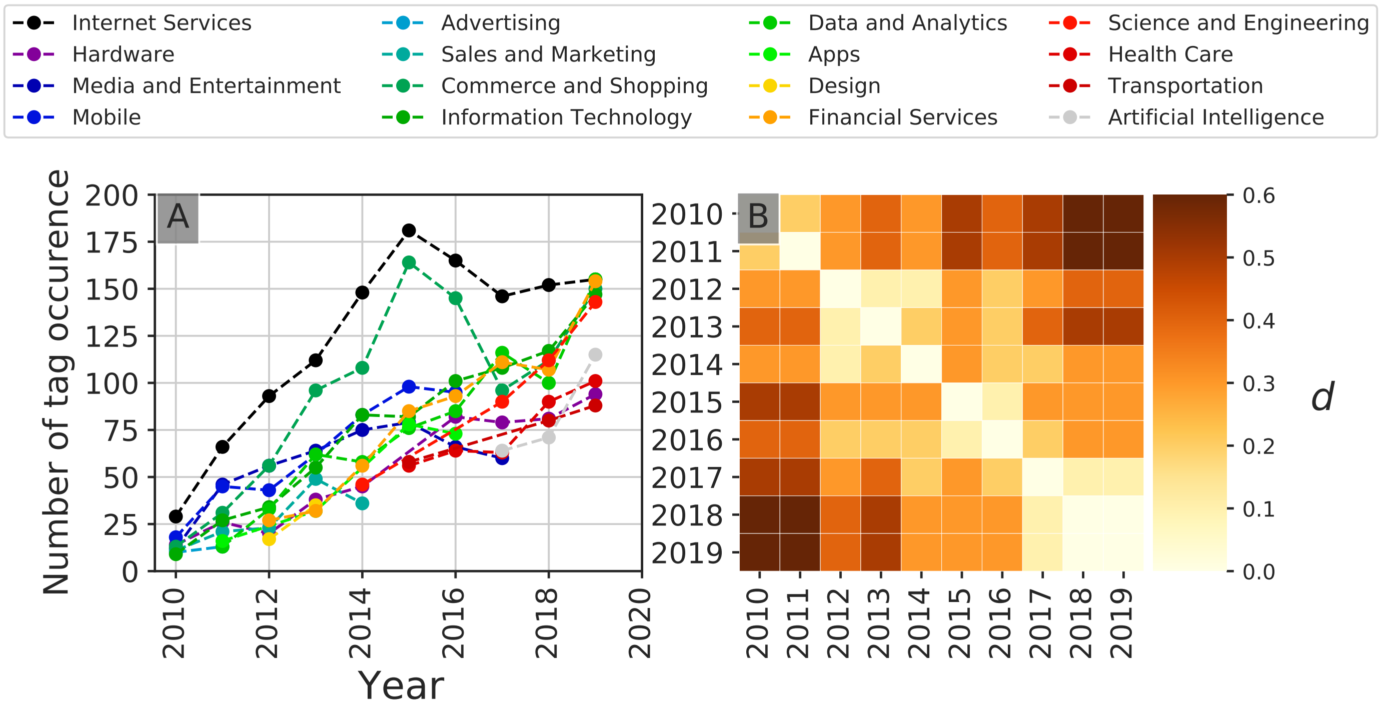

Fig S168. Temporal evolution of the investment patterns of community C9.

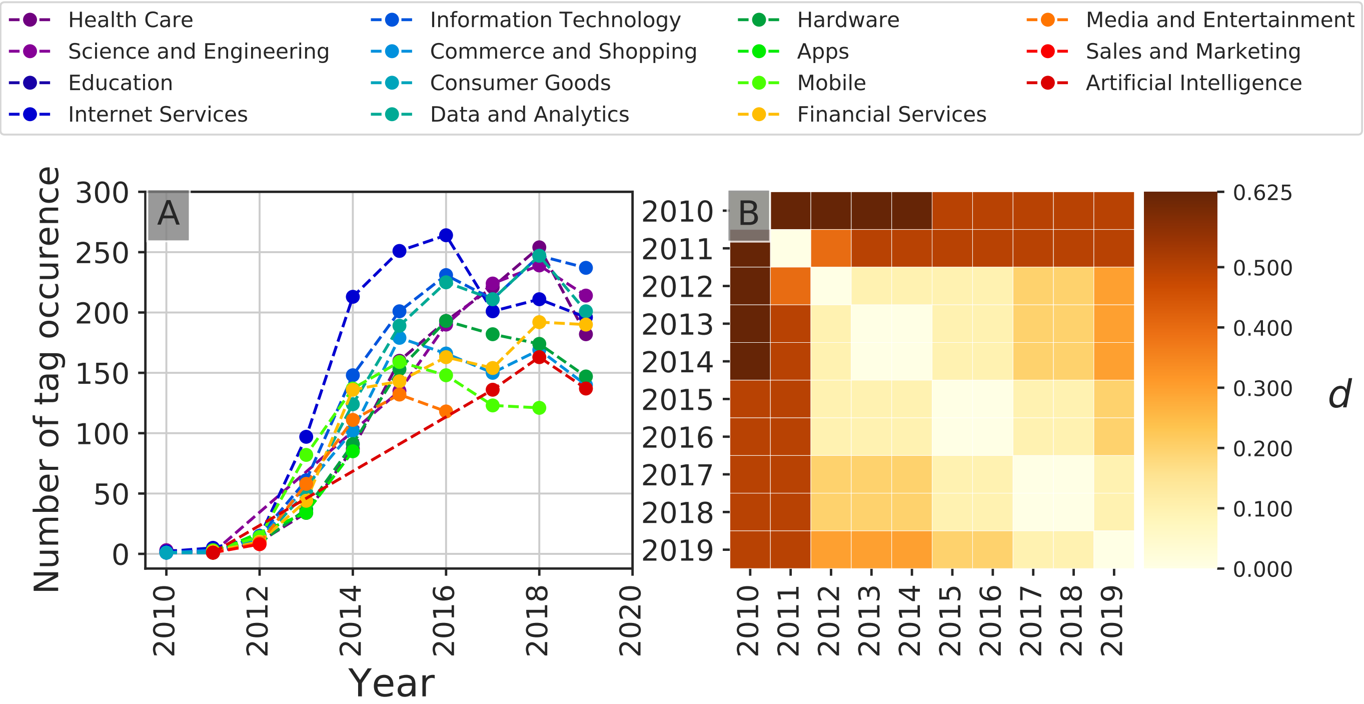

Fig S169. Temporal evolution of the investment patterns of community C10.

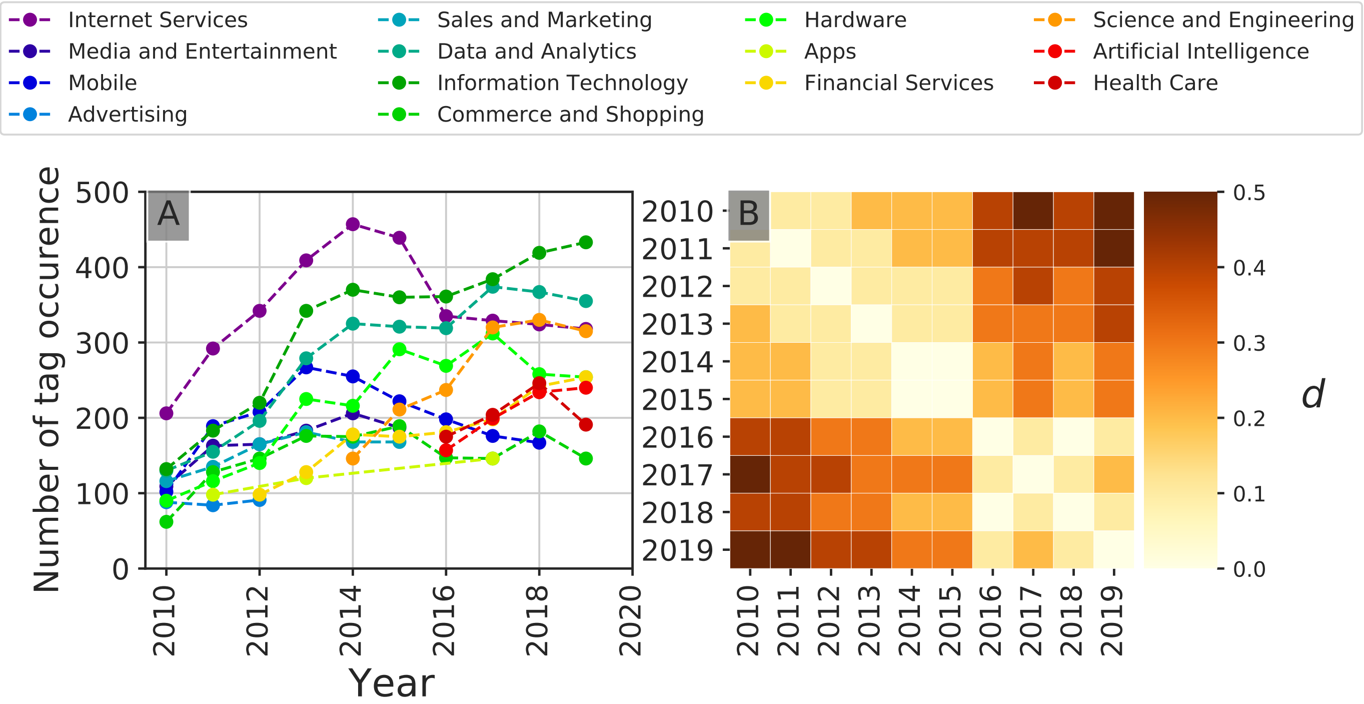

Fig S170. Temporal evolution of the investment patterns of community D0.

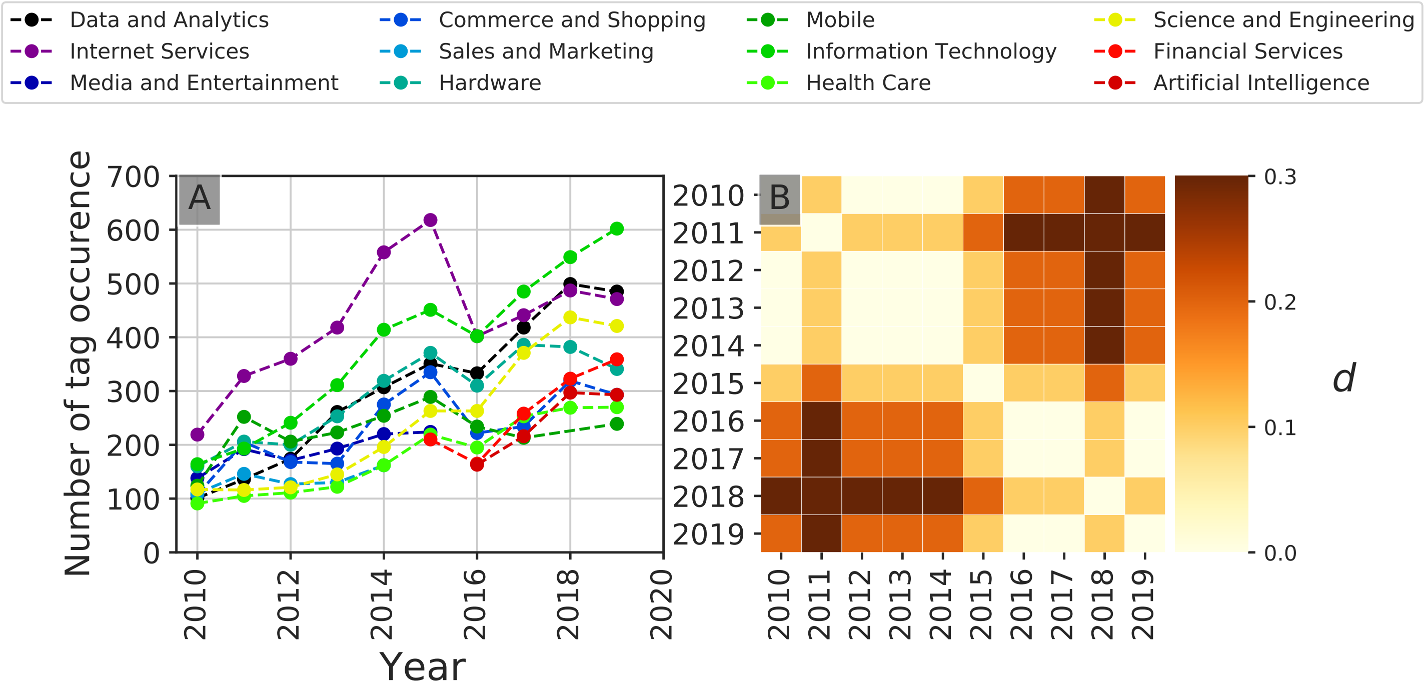

Fig S171. Temporal evolution of the investment patterns of community D1.

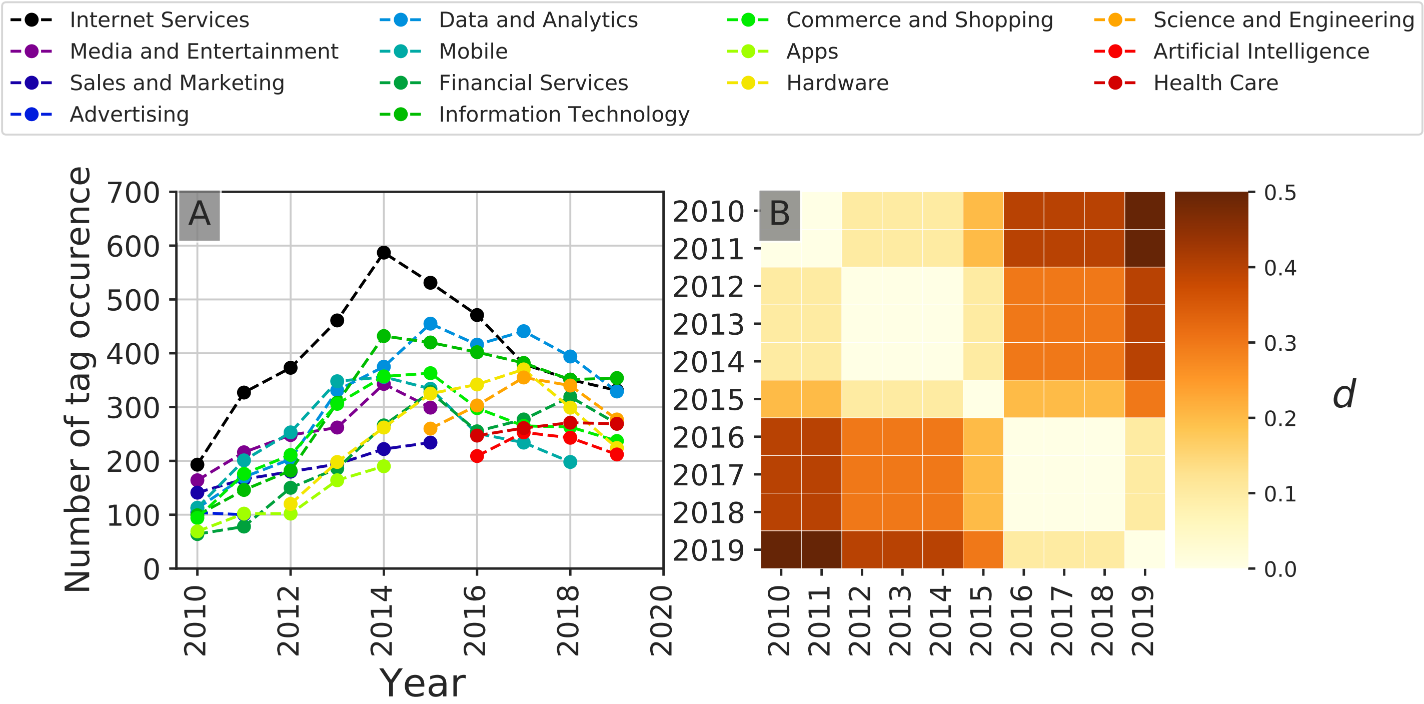

Fig S172. Temporal evolution of the investment patterns of community D2.

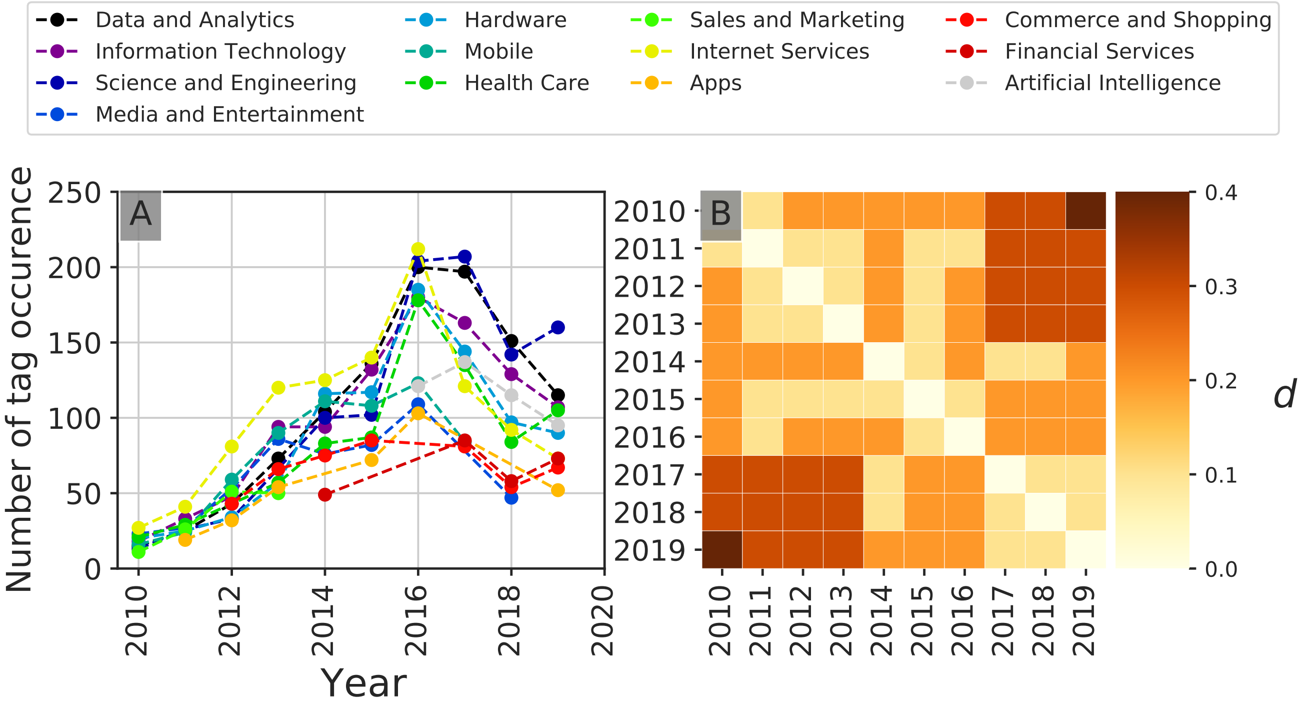

Fig S173. Temporal evolution of the investment patterns of community D3.

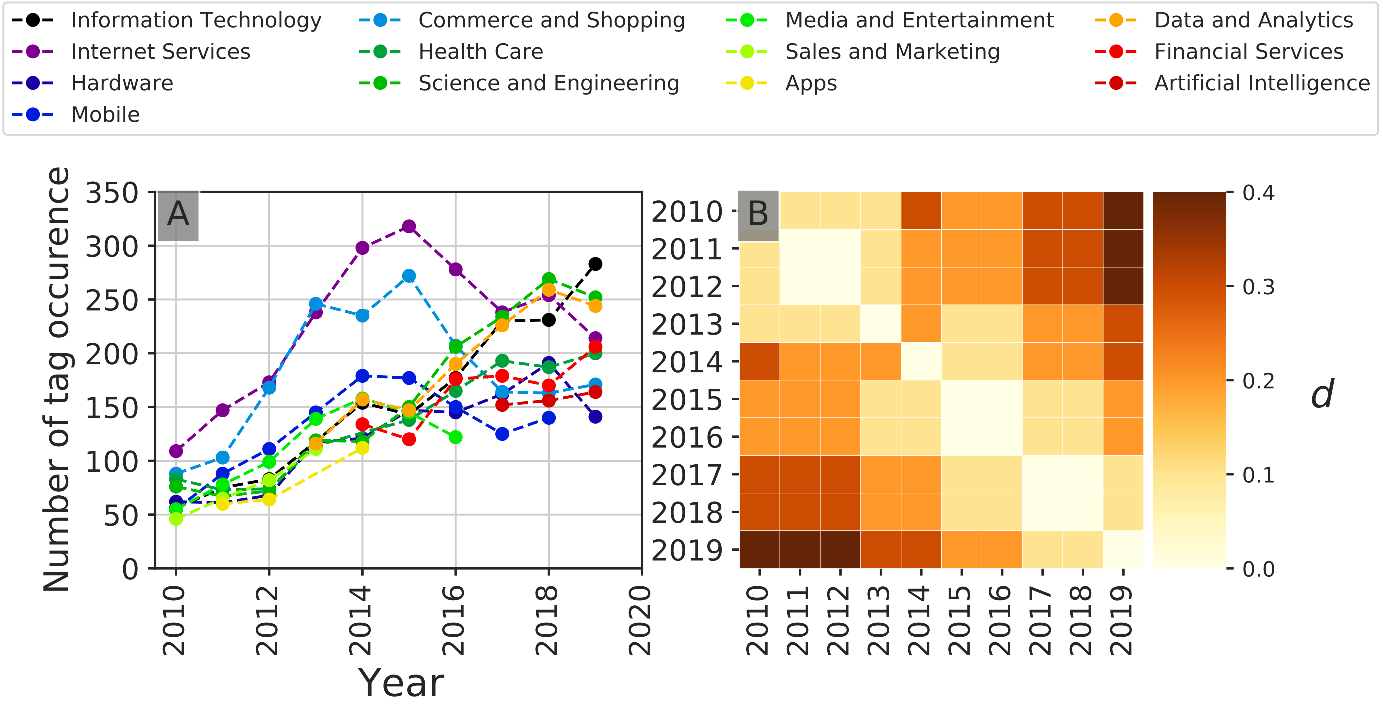

Fig S174. Temporal evolution of the investment patterns of community D4.

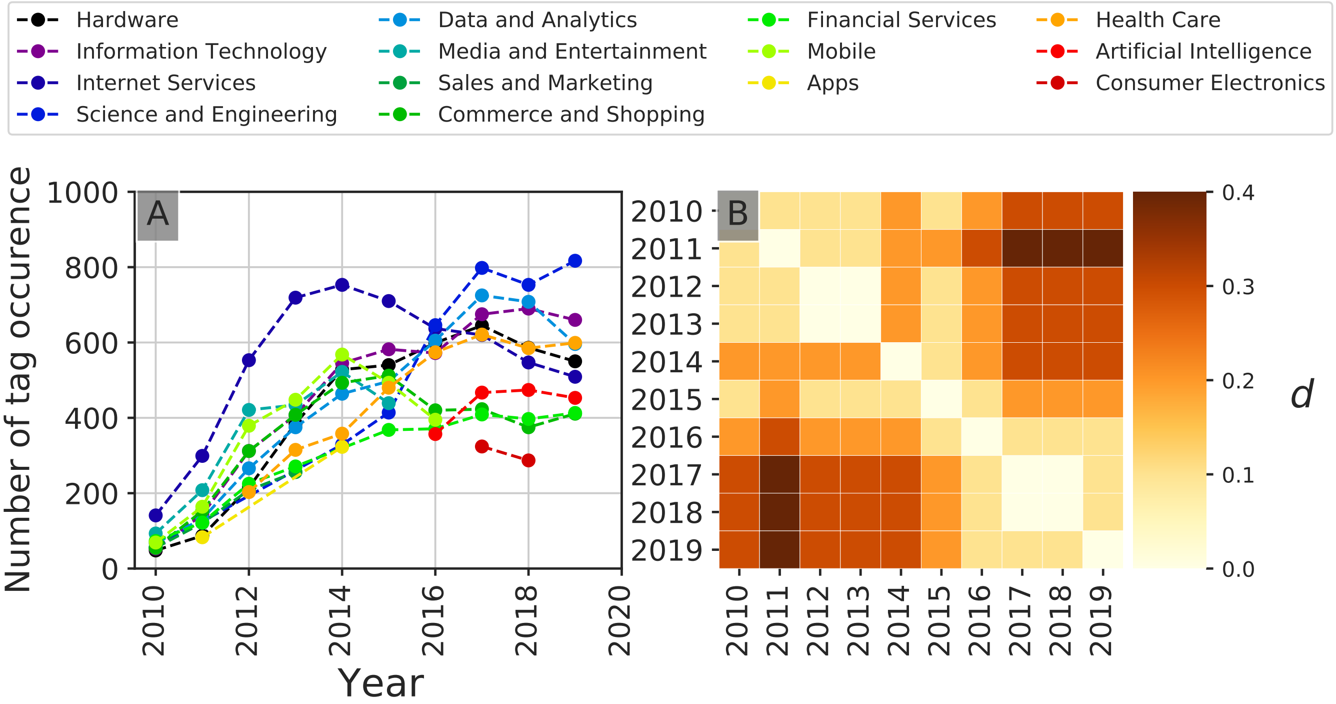

Fig S175. Temporal evolution of the investment patterns of community D5.

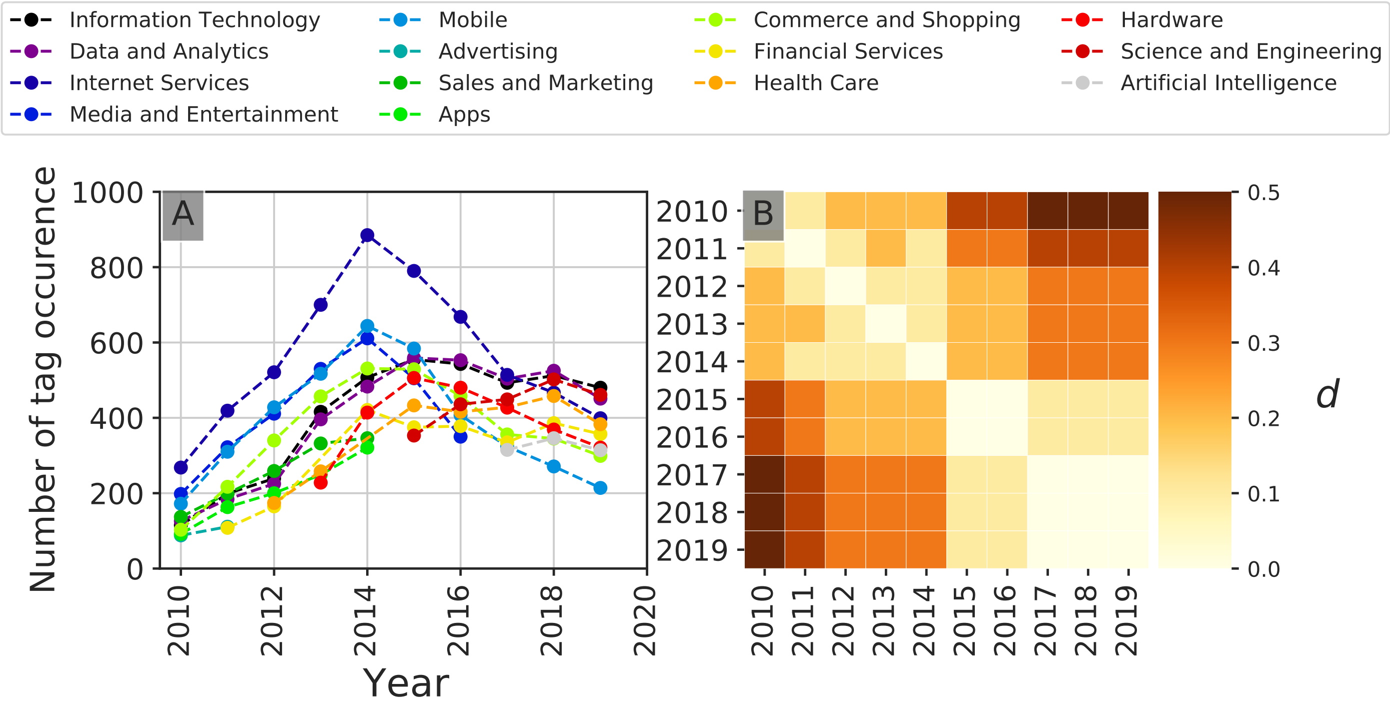

Fig S176. Temporal evolution of the investment patterns of community D6.

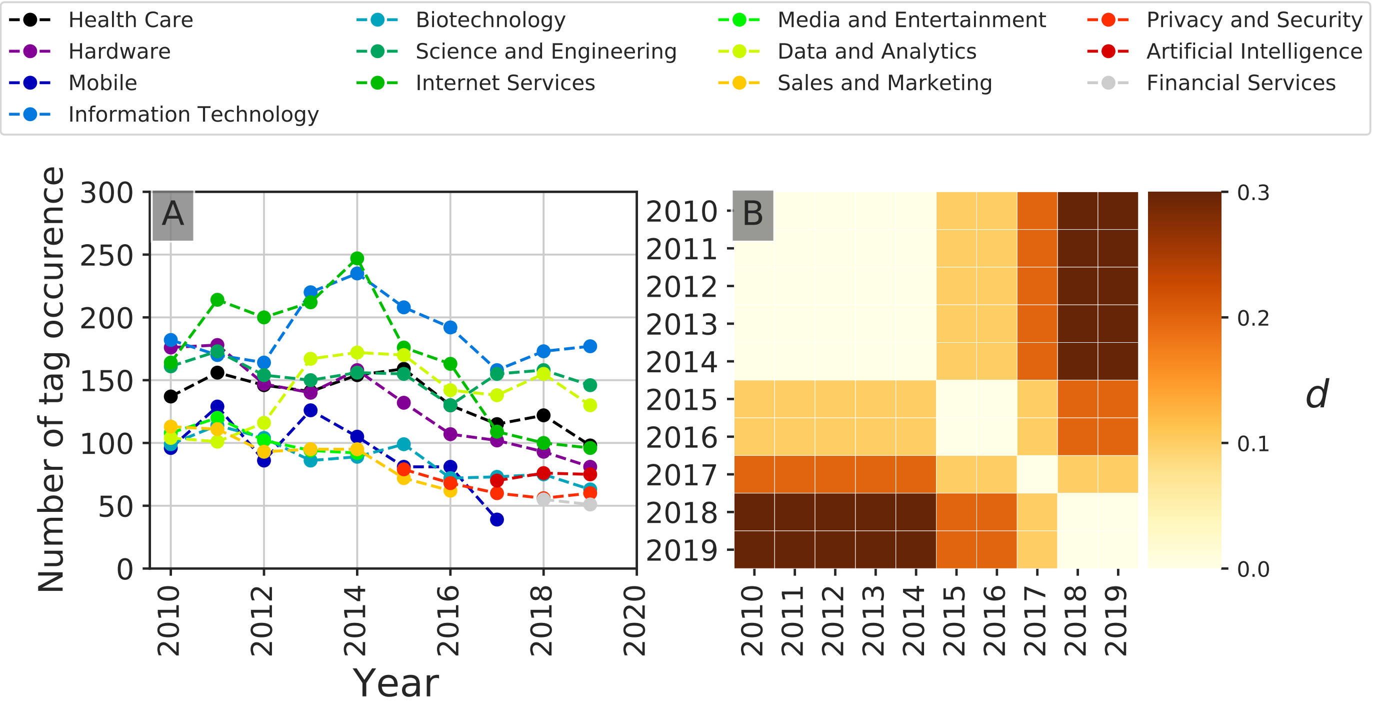

Fig S177. Temporal evolution of the investment patterns of community D7.

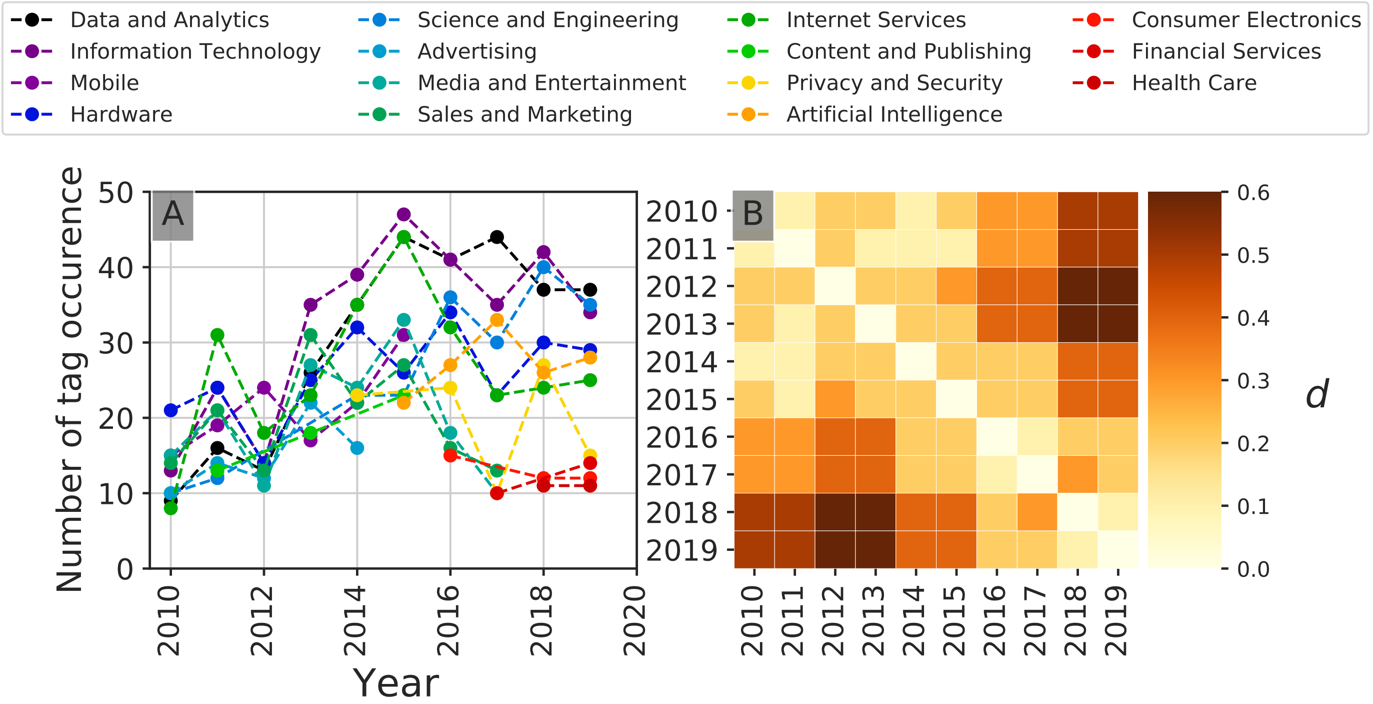

Fig S178. Temporal evolution of the investment patterns of community D8.

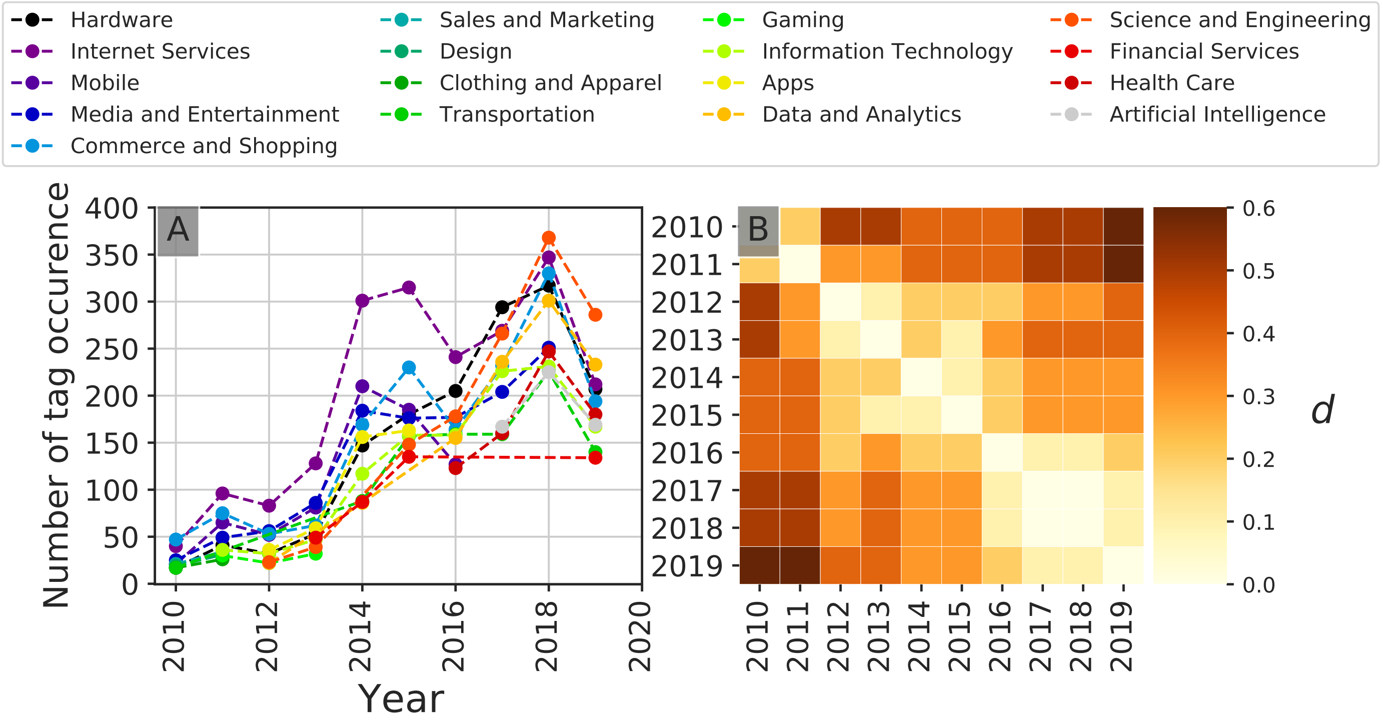

Fig S179. Temporal evolution of the investment patterns of community D9.

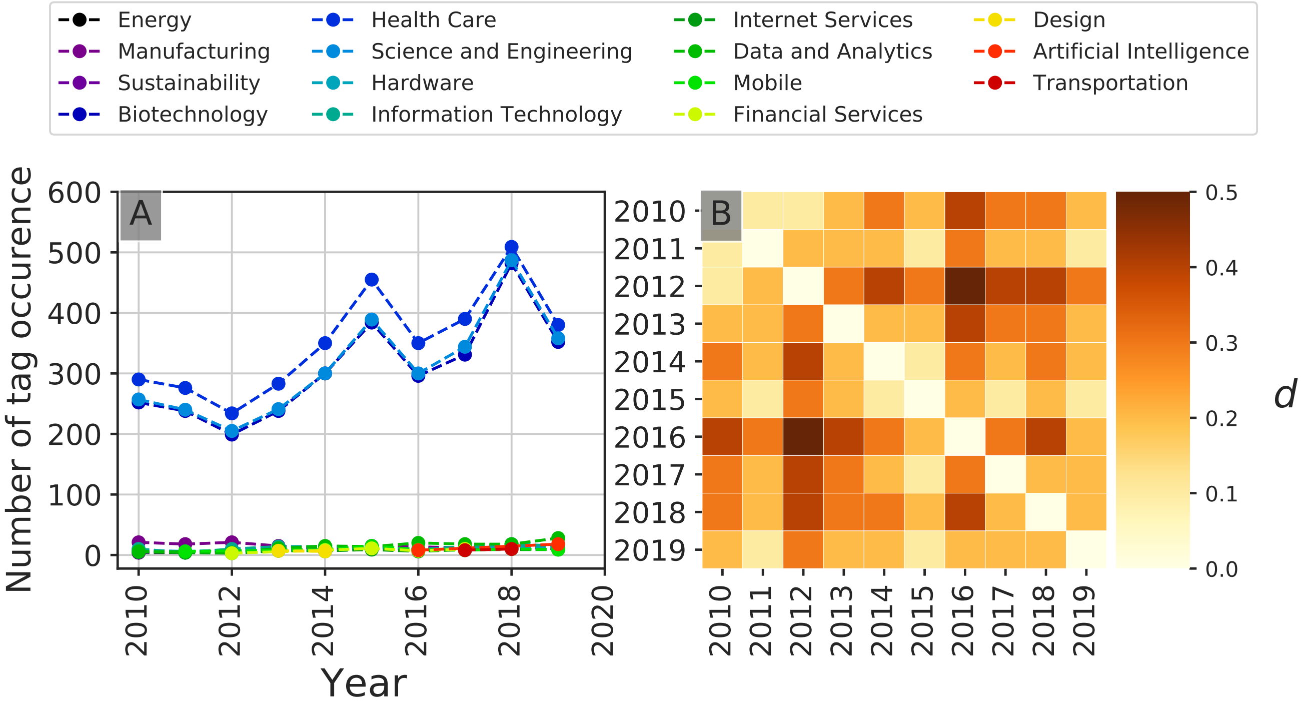

Fig S180. Temporal evolution of the investment patterns of community D10.

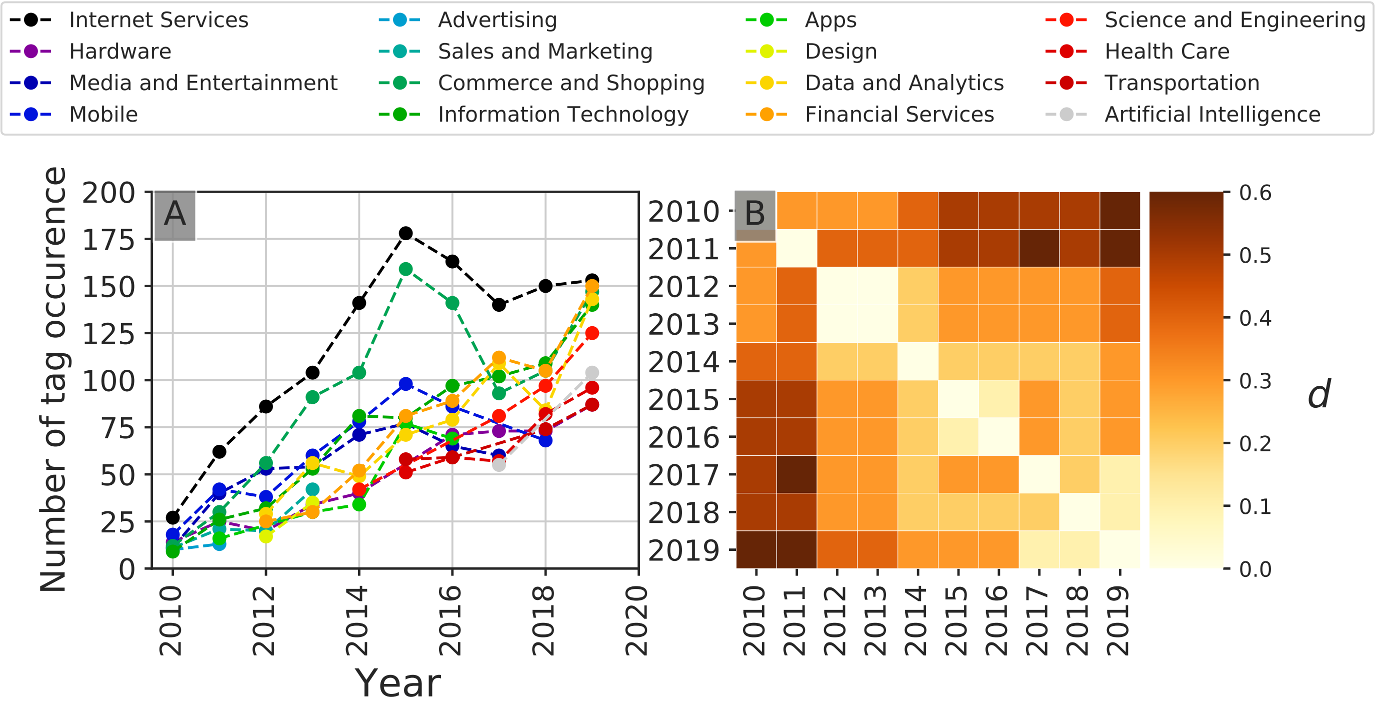

Fig S181. Temporal evolution of the investment patterns of community D11.

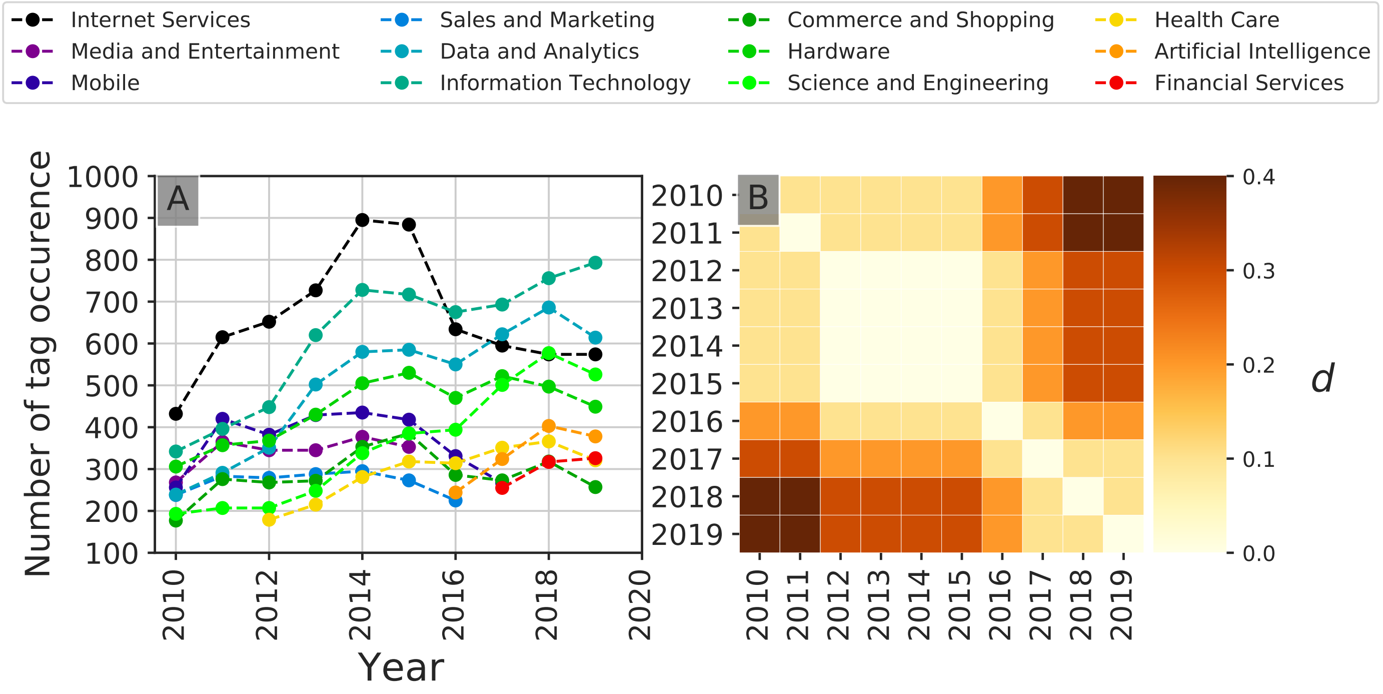

Fig S182. Temporal evolution of the investment patterns of community E0.

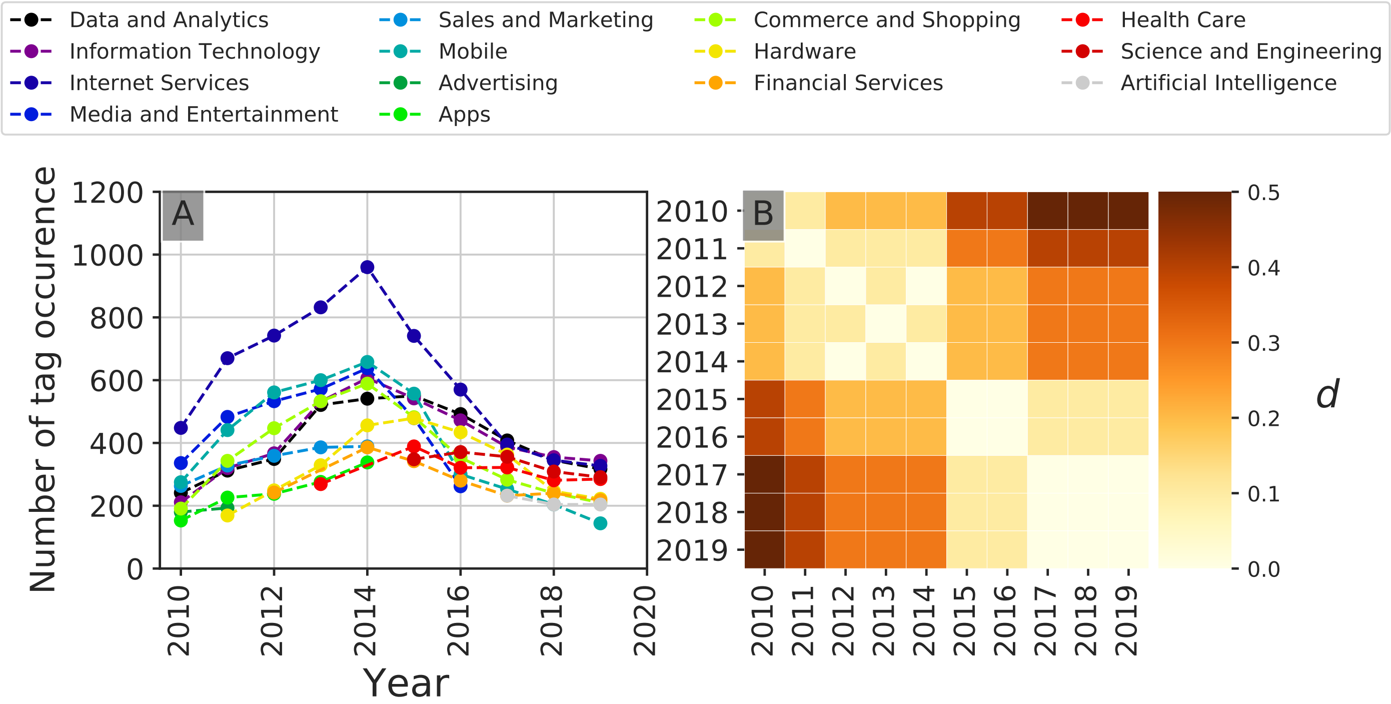

Fig S183. Temporal evolution of the investment patterns of community E1.

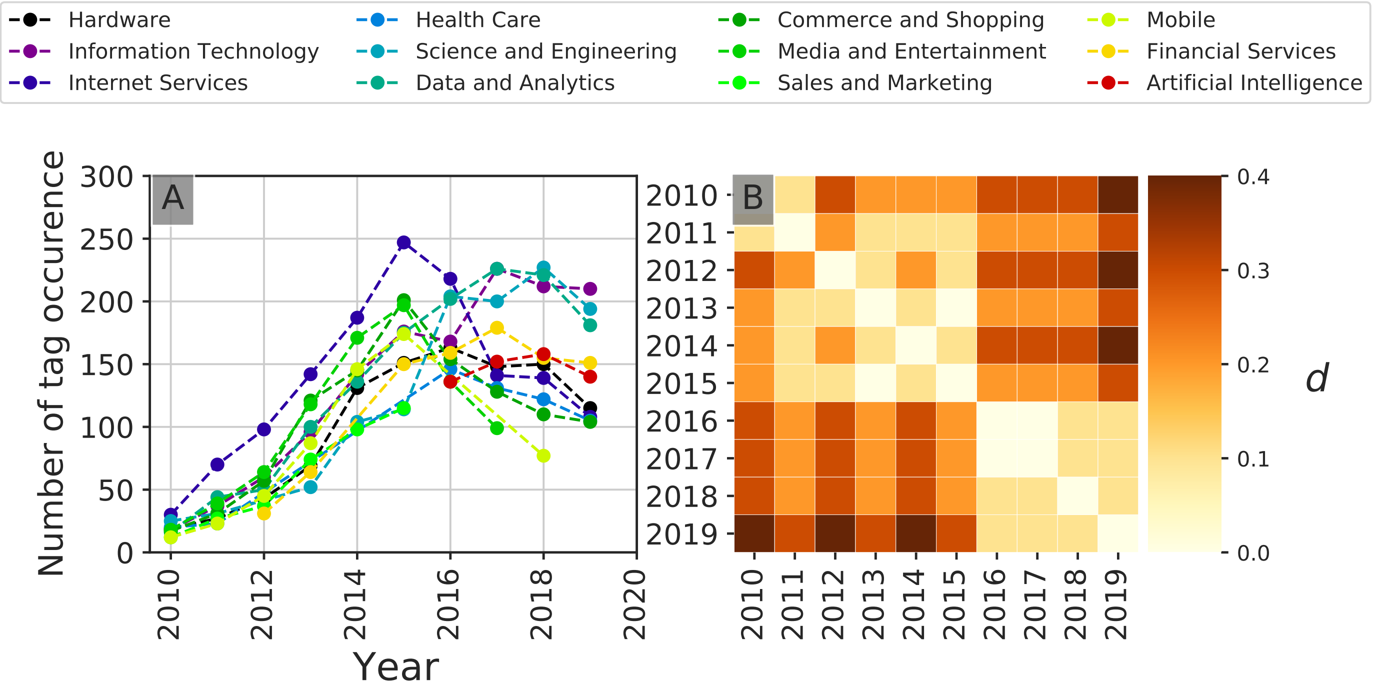

Fig S184. Temporal evolution of the investment patterns of community E2.

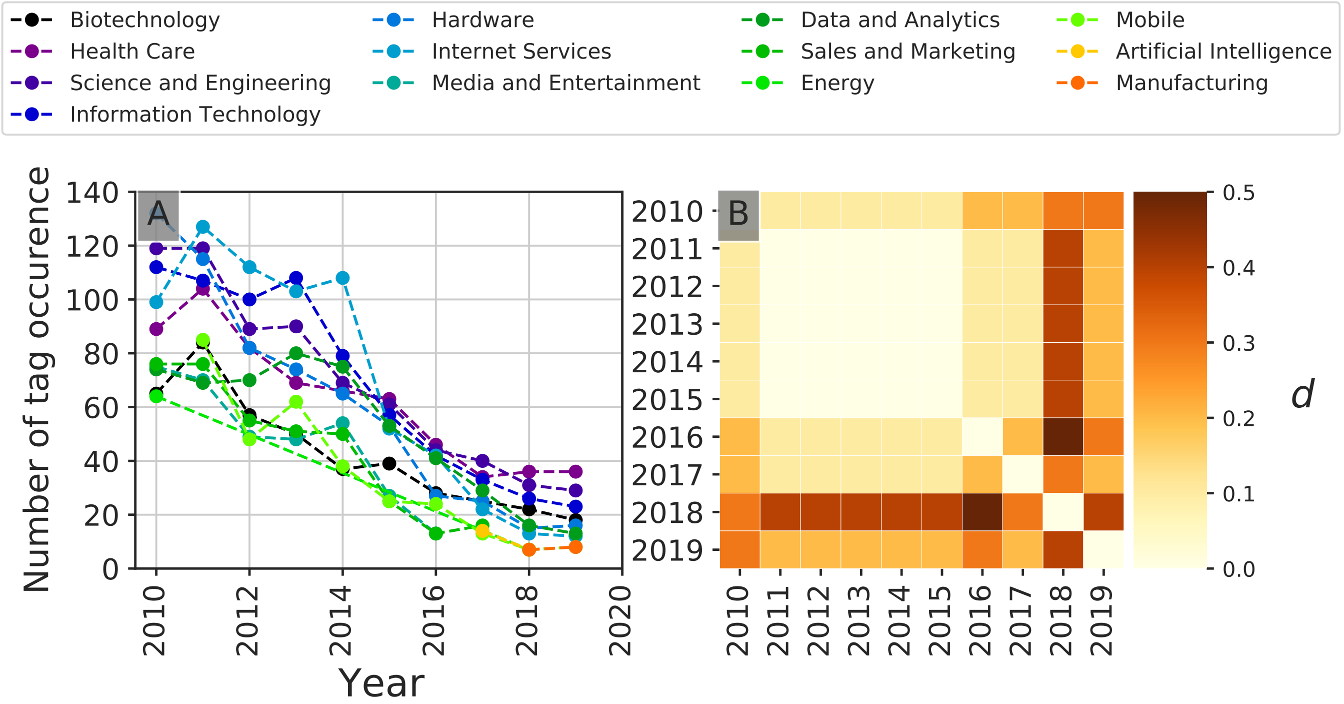

Fig S185. Temporal evolution of the investment patterns of community E3.

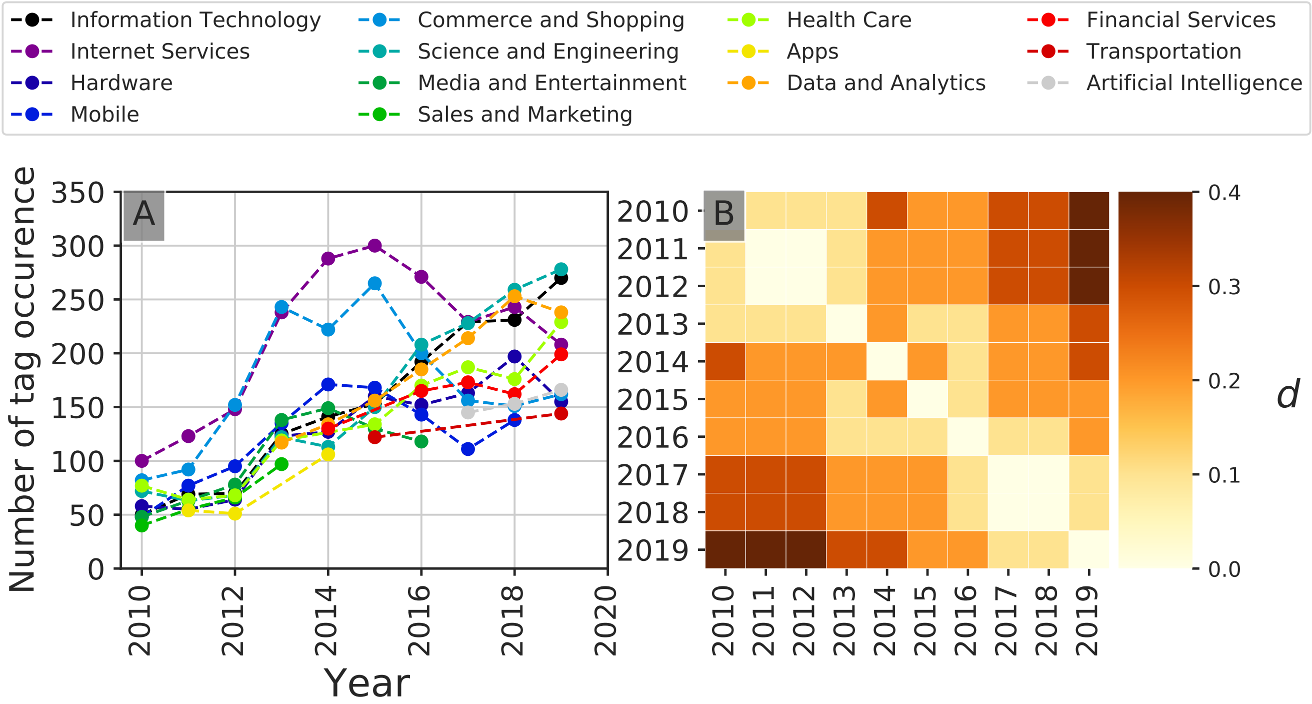

Fig S186. Temporal evolution of the investment patterns of community E4.

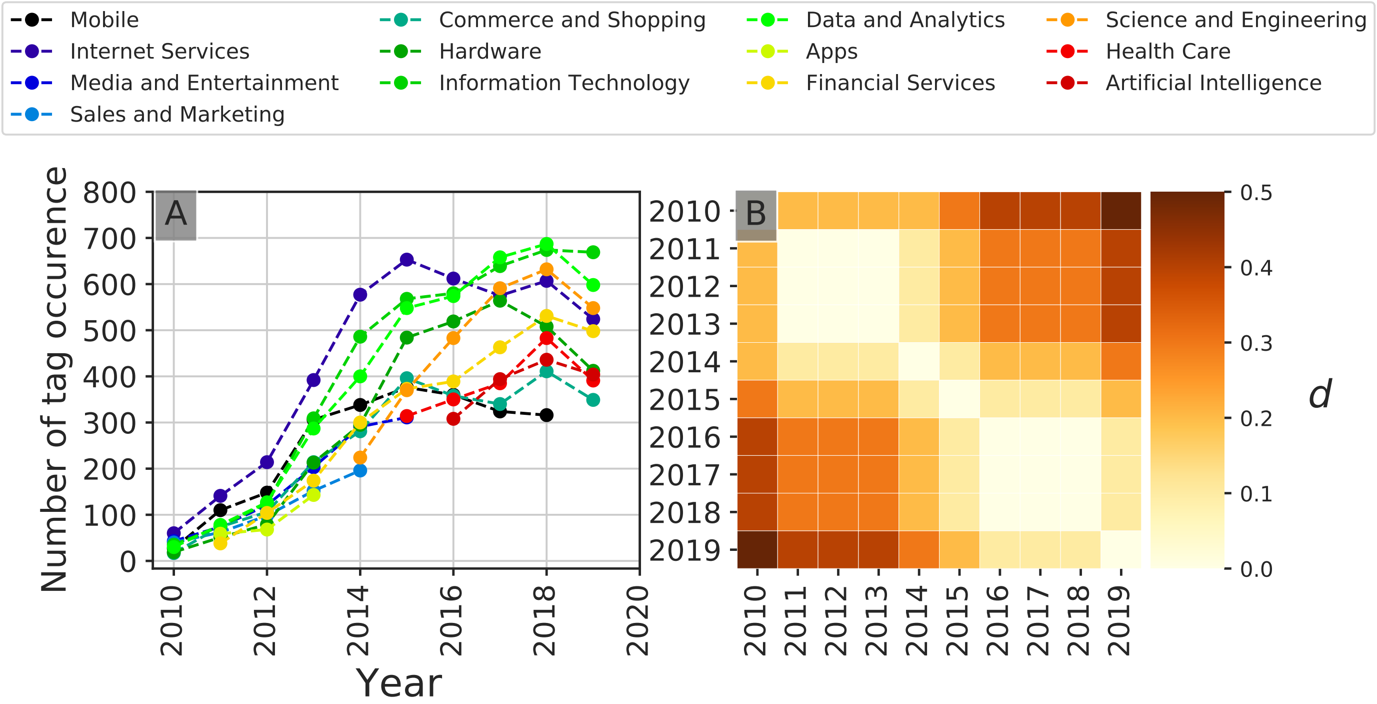

Fig S187. Temporal evolution of the investment patterns of community E5.

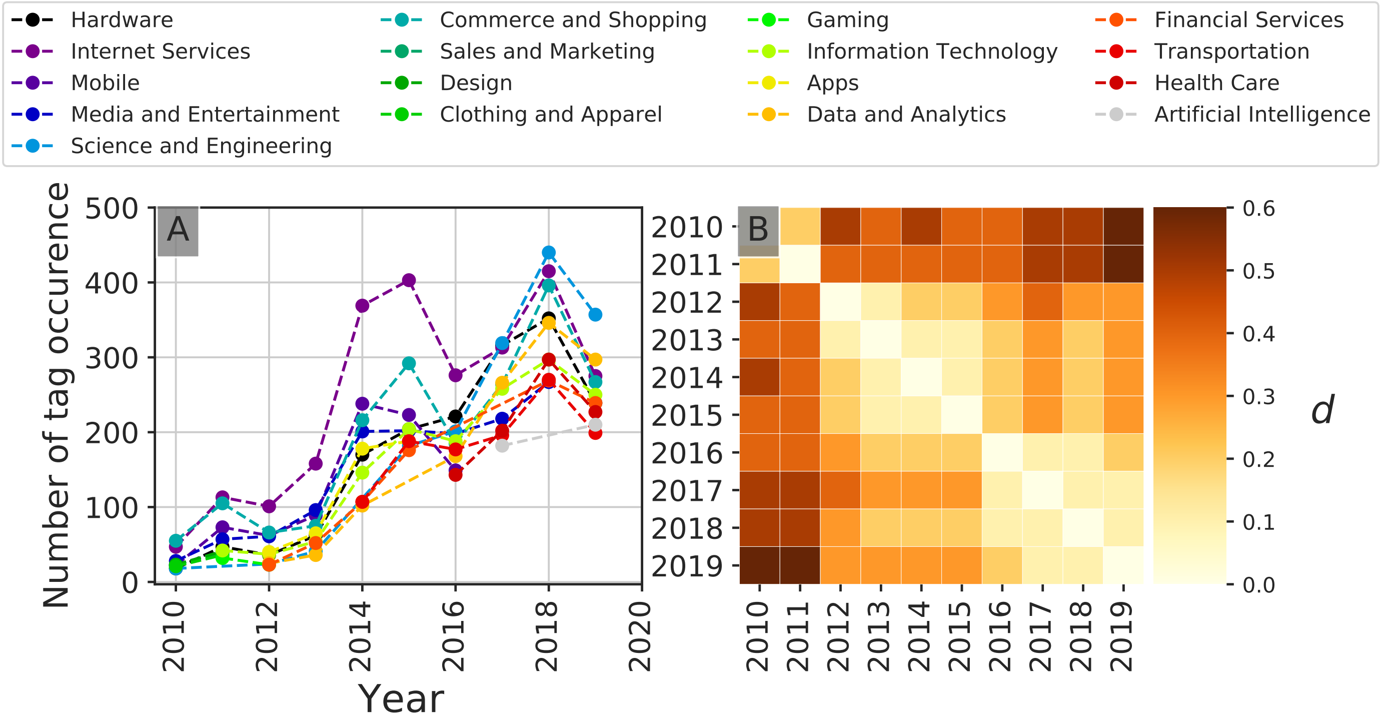

Fig S188. Temporal evolution of the investment patterns of community E6.

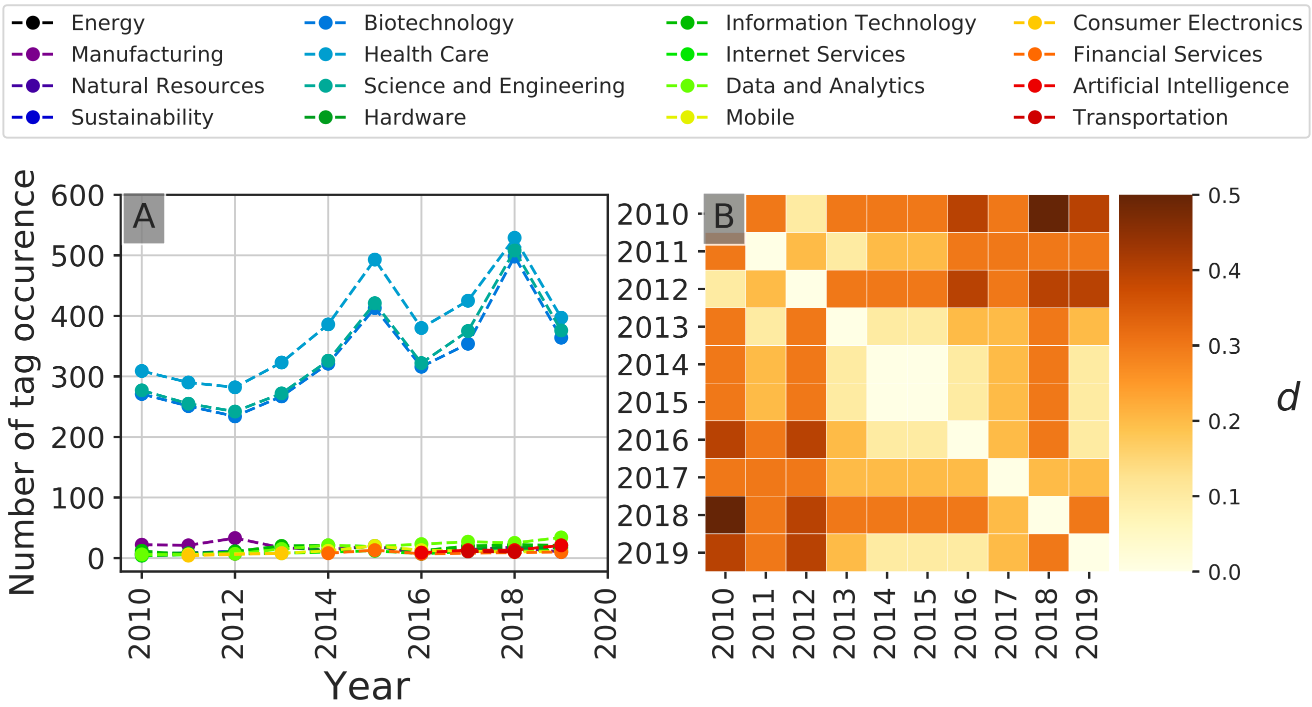

Fig S189. Temporal evolution of the investment patterns of community E7.

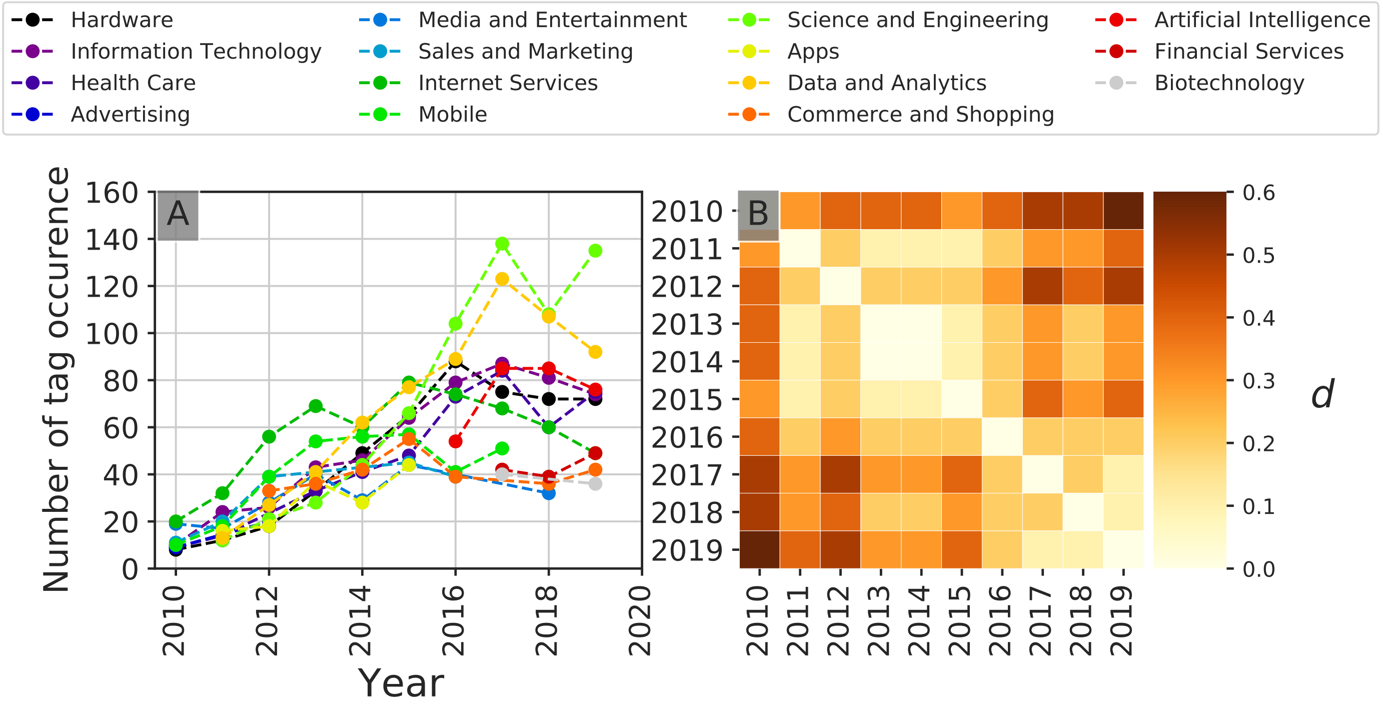

Fig S190. Temporal evolution of the investment patterns of community E8.

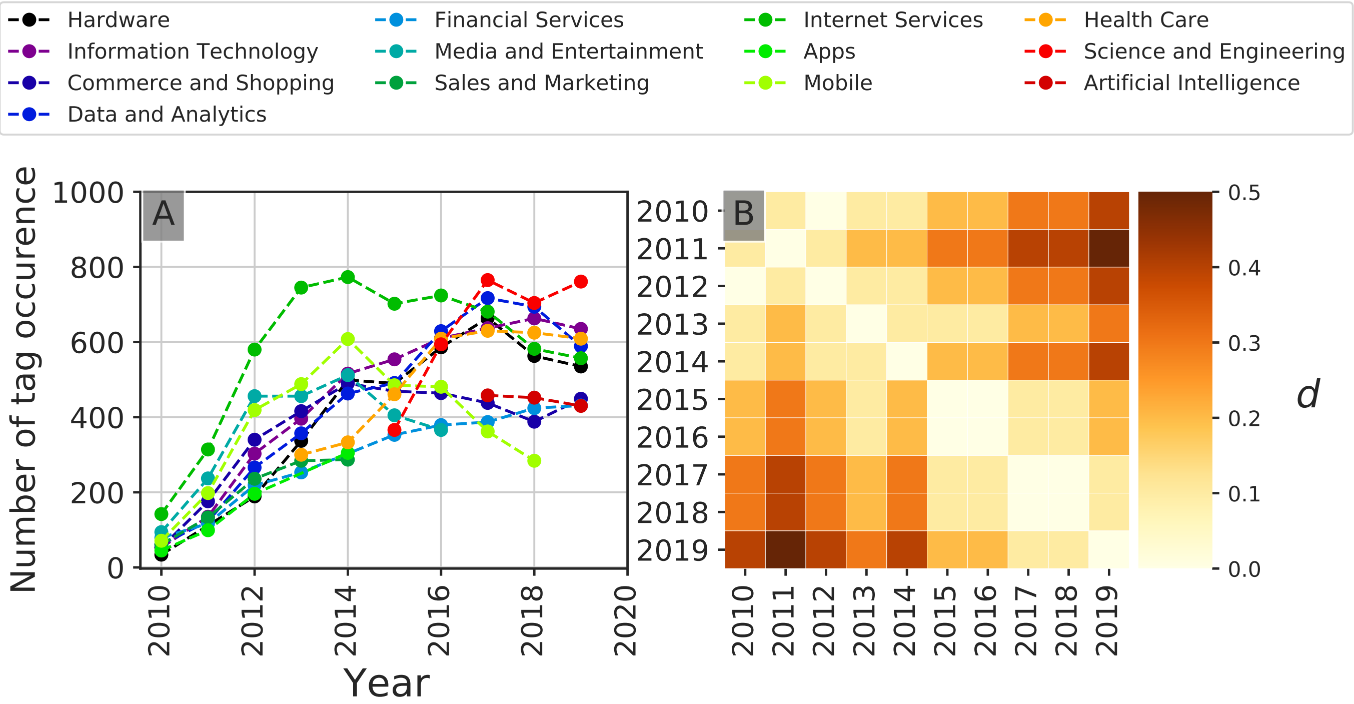

Fig S191. Temporal evolution of the investment patterns of community E9.

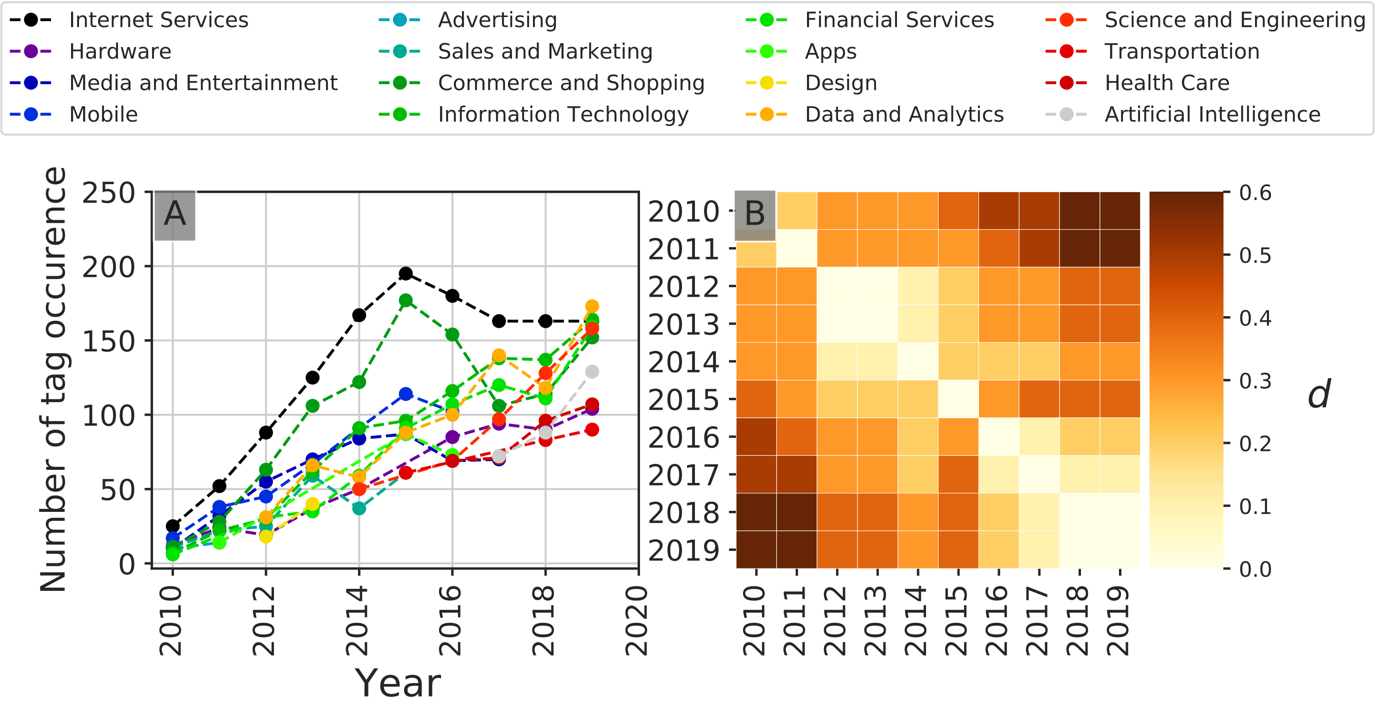

Fig S192. Temporal evolution of the investment patterns of community E10.

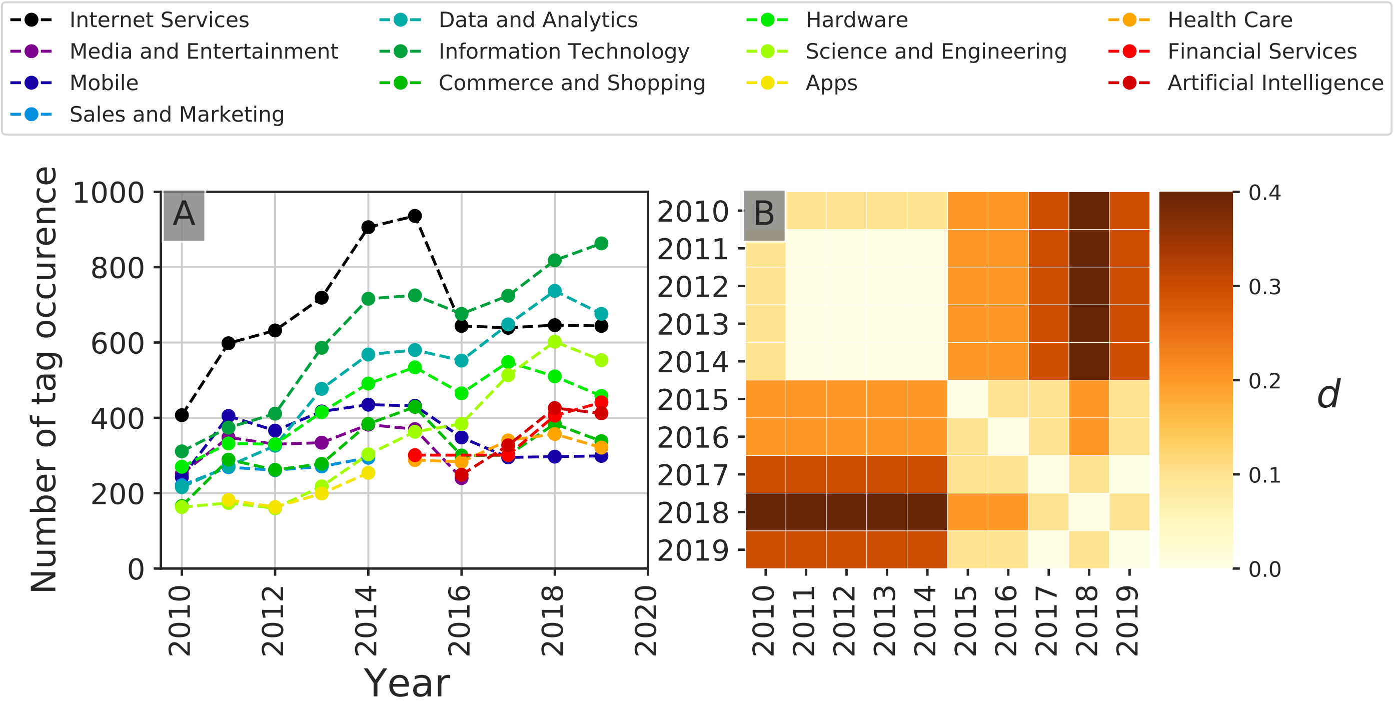

Fig S193. Temporal evolution of the investment patterns of community F0.

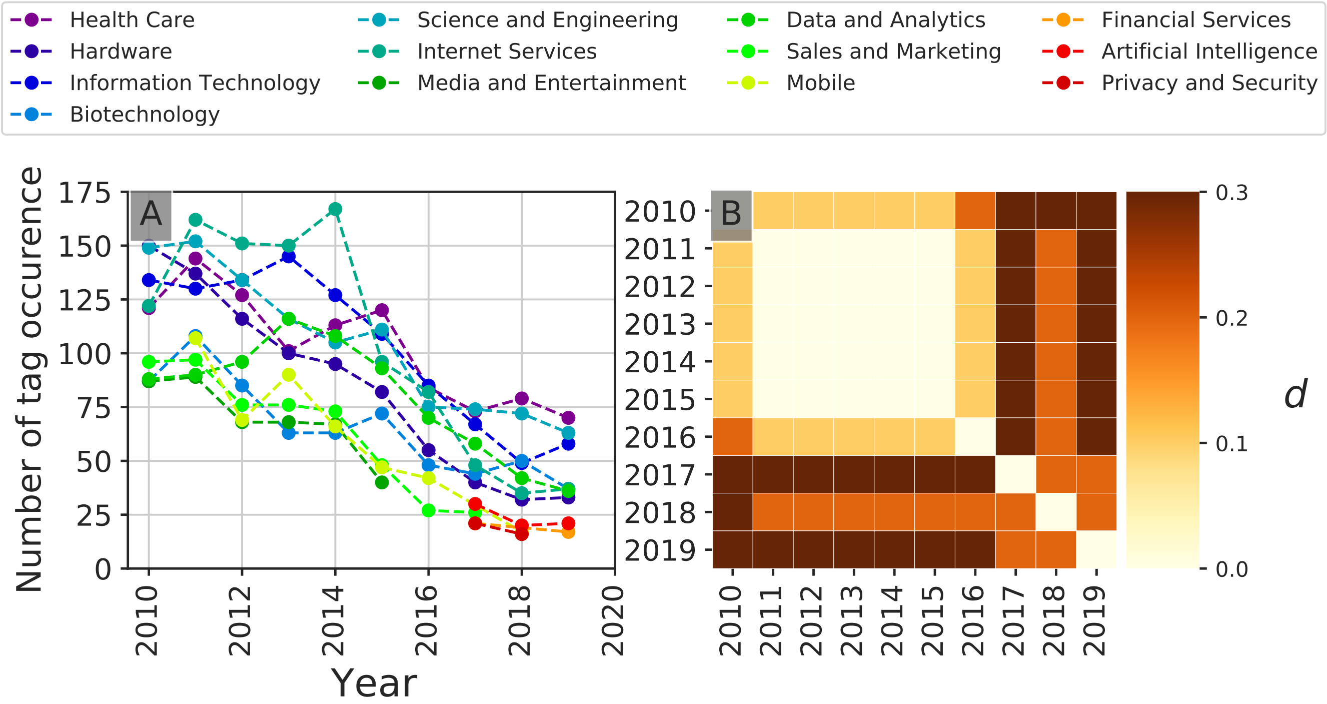

Fig S194. Temporal evolution of the investment patterns of community F1.

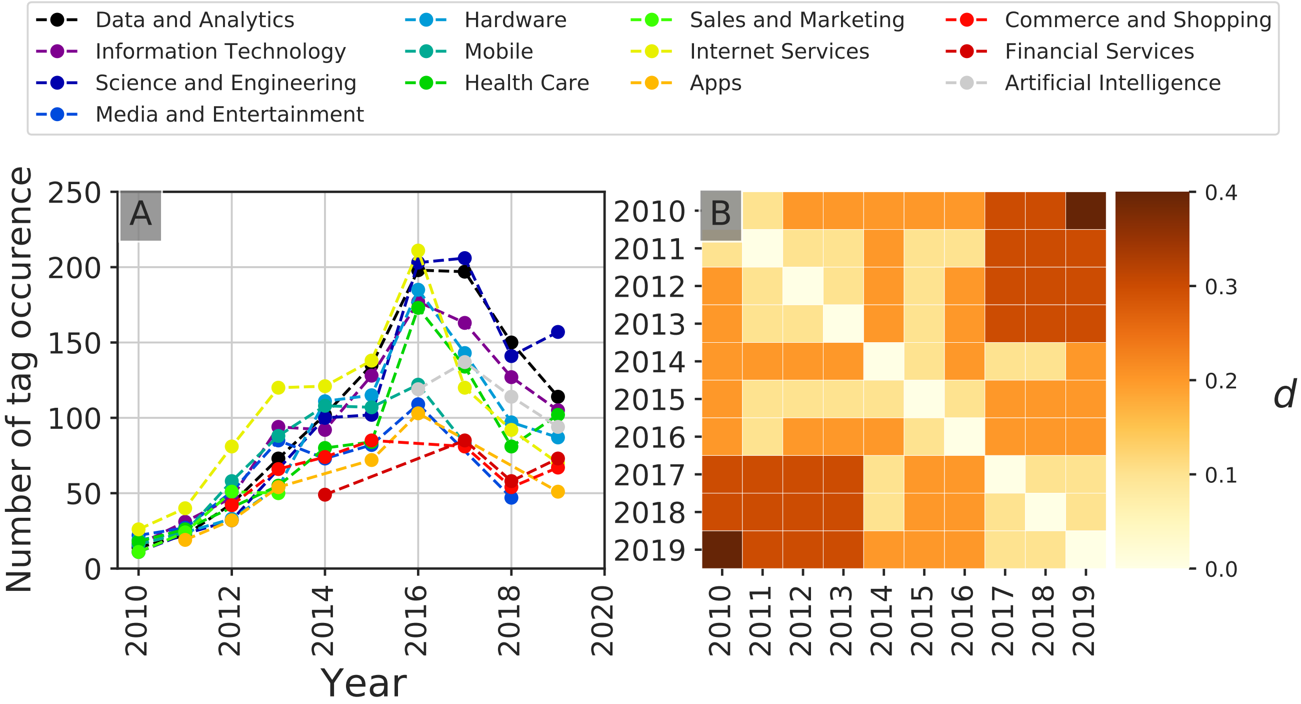

Fig S195. Temporal evolution of the investment patterns of community F2.

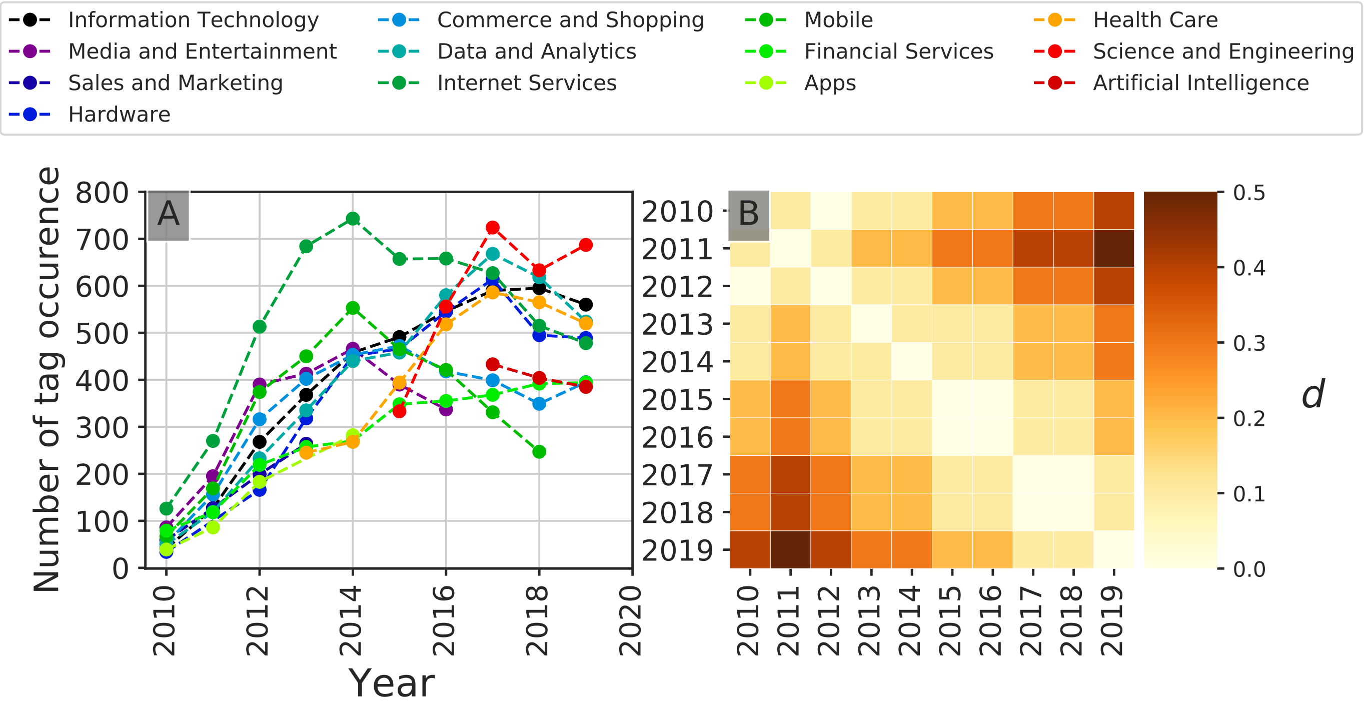

Fig S196. Temporal evolution of the investment patterns of community F3.

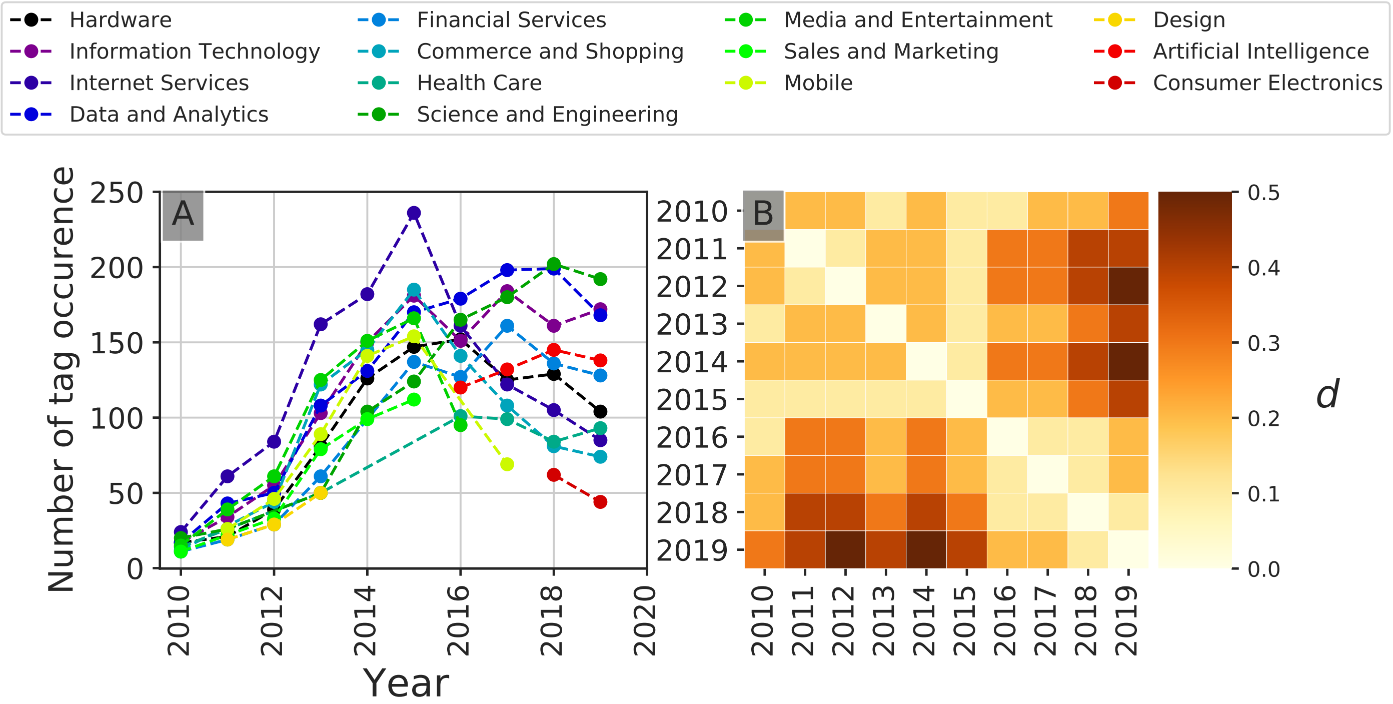

Fig S197. Temporal evolution of the investment patterns of community F4.

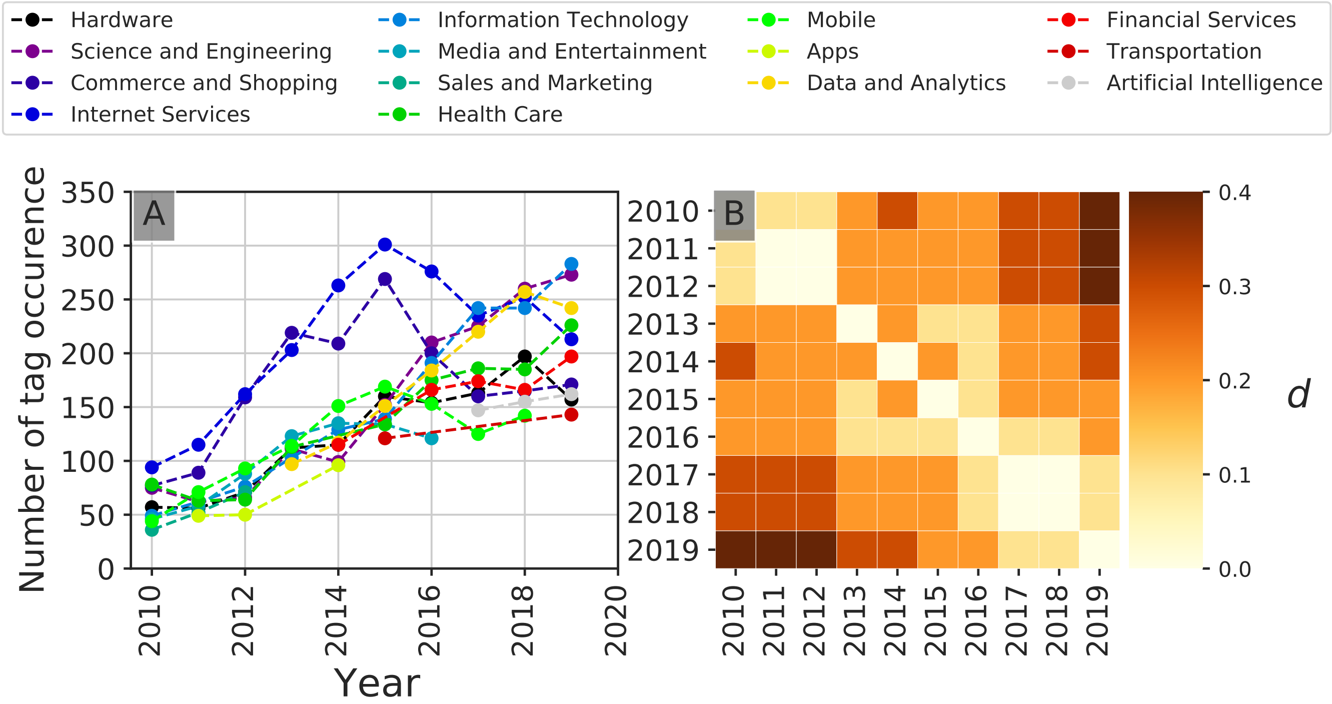

Fig S198. Temporal evolution of the investment patterns of community F5.

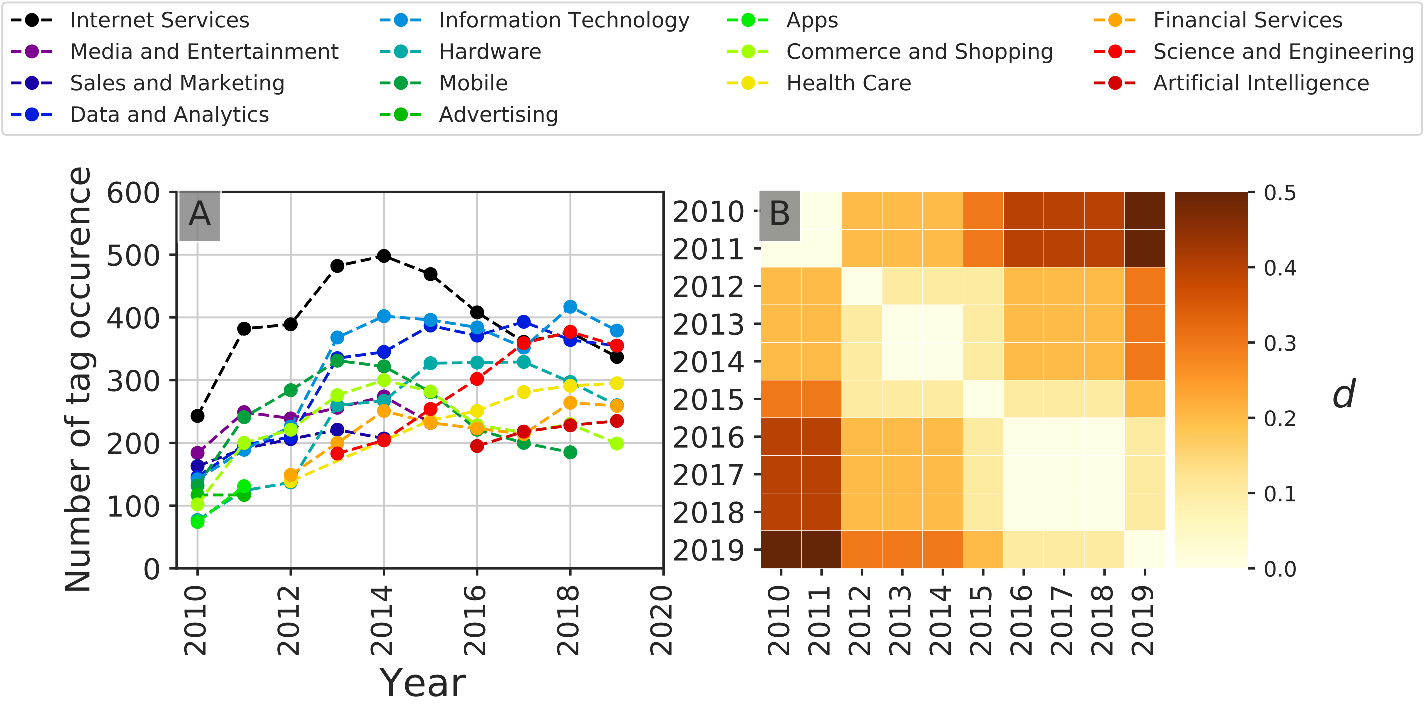

Fig S199. Temporal evolution of the investment patterns of community F6.

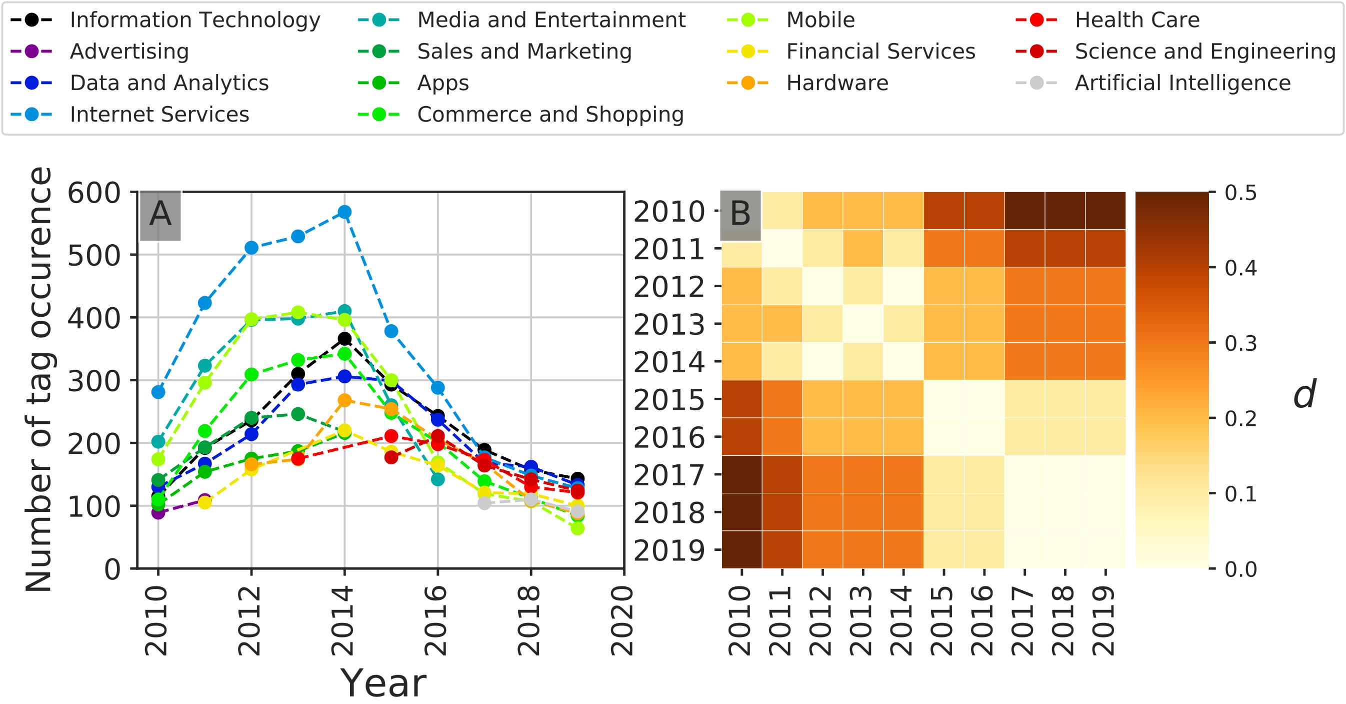

Fig S200. Temporal evolution of the investment patterns of community F7.

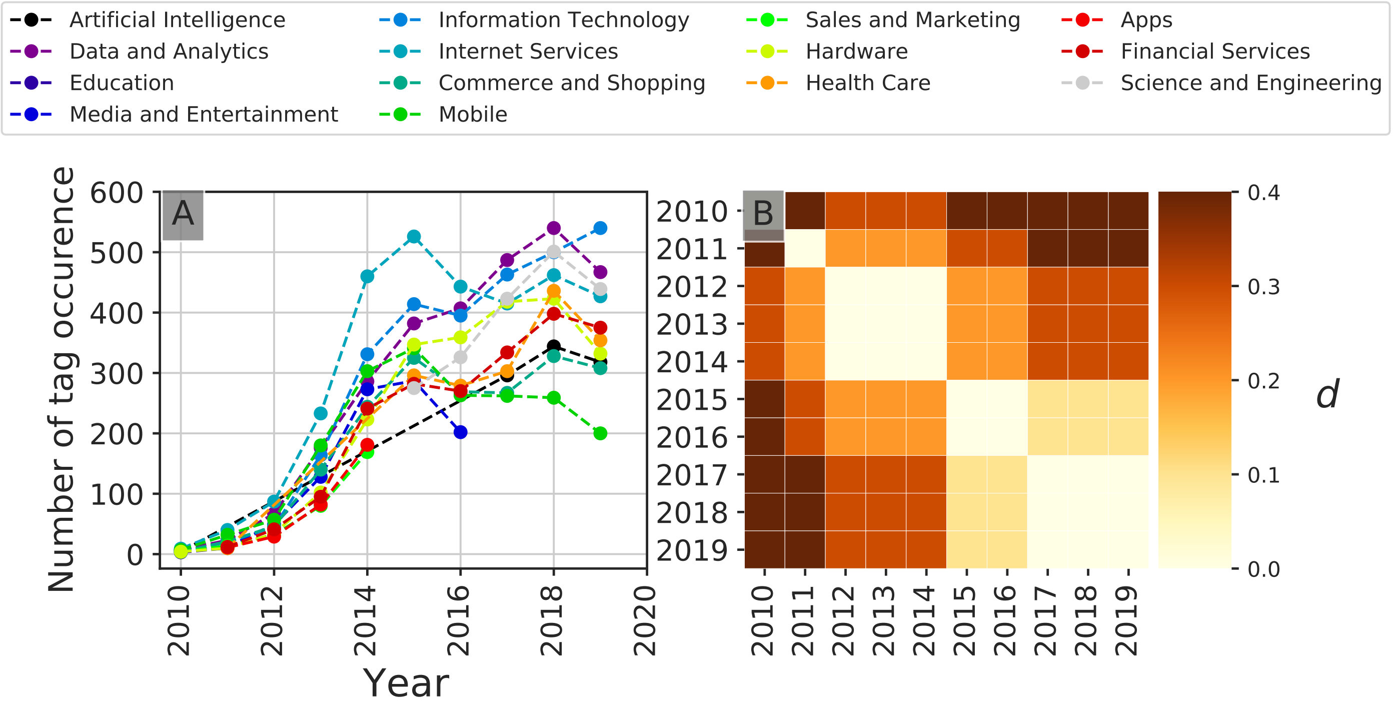

Fig S201. Temporal evolution of the investment patterns of community F8.

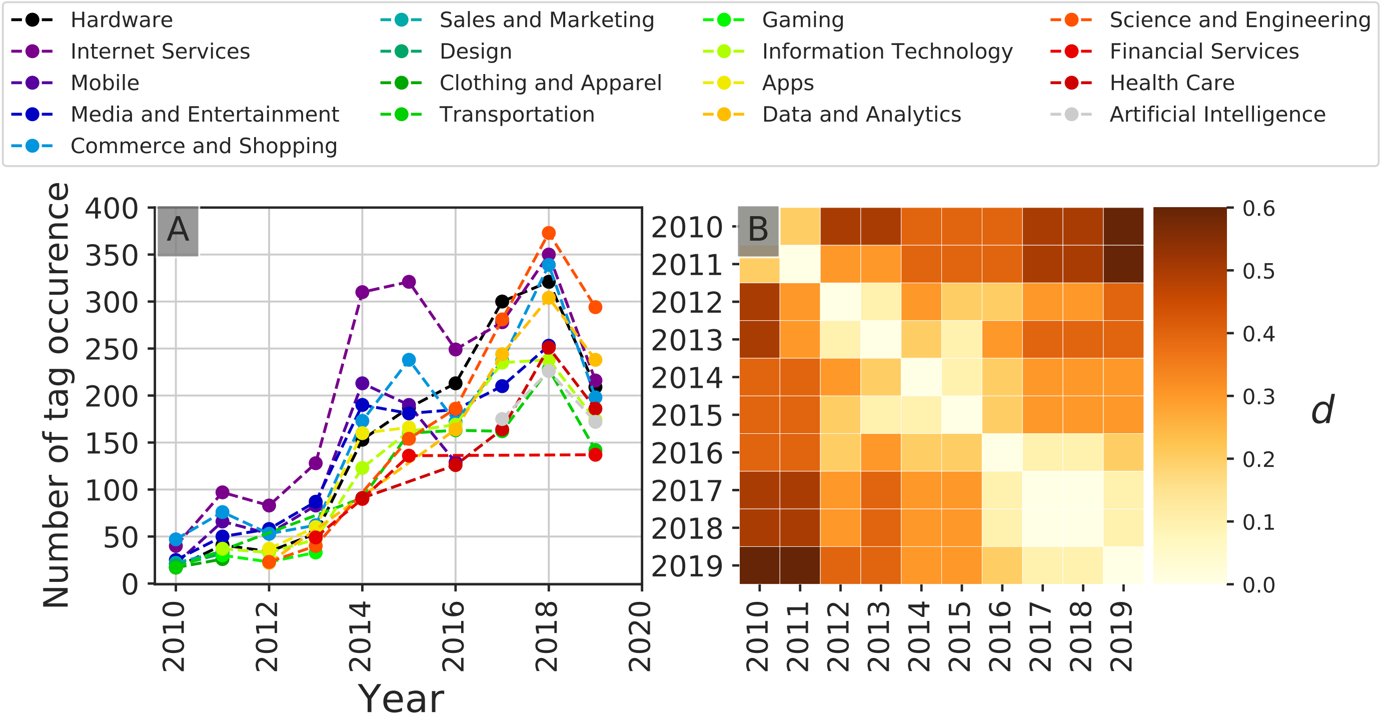

Fig S202. Temporal evolution of the investment patterns of community F9.

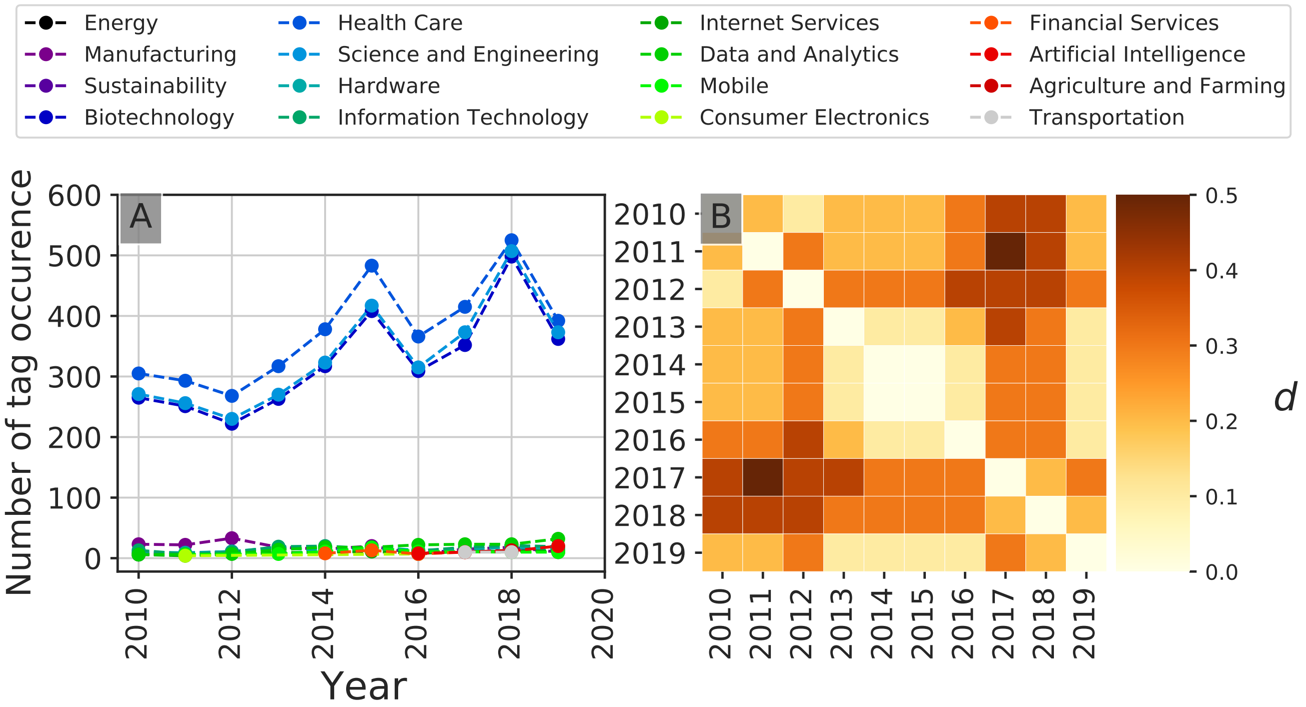

Fig S203. Temporal evolution of the investment patterns of community F10.

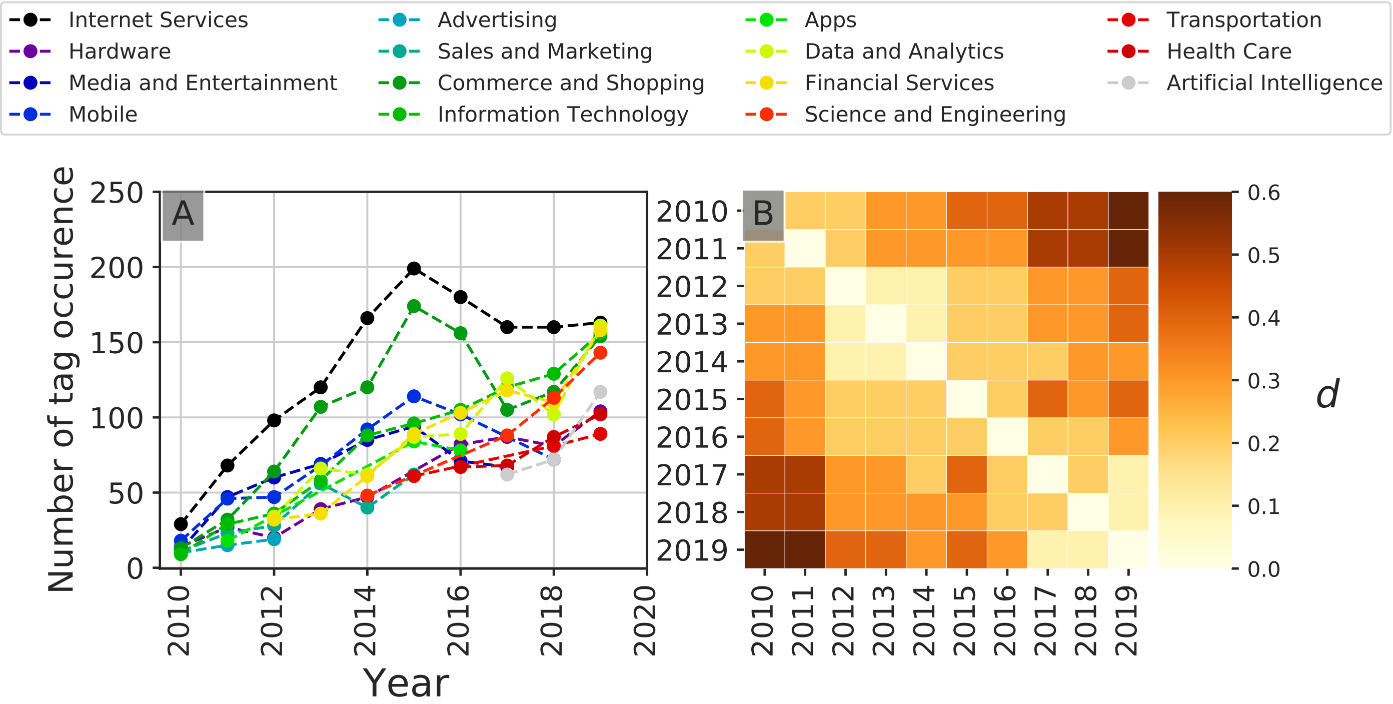

Fig S204. Temporal evolution of the investment patterns of community F11.
