# Supplementary material for: A novel clustering approach to bipartite investor-startup networks
Source: PLoS One. 2023 Jan 5;18(1):e0279780. doi: 10.1371/journal.pone.0279780 (PMC9815571; doi:10.1371/journal.pone.0279780)
Supplement: Attachment — Submitted filename: Response to Reviewers.pdf [file pone.0279780.s003.pdf]

We thank the referees for carefully reading our article and for their constructive and helpful comments. Here are the comments and changes in the article.

1. **The motivation for the utilization of the proposed community detection algorithm based on the Hellinger-based similarity measure is unclear. Please specify this more clearly.**

- As the investors in our network are highly heterogeneous, we believe that a suitable methodology to cluster similar investors is necessary in order to study investment dynamics. The investor-startup network, however, is a complex bipartite multilayer multigraph, meaning that investors are multi-dimensional items defined along the various axes of the graph. Efficient clustering algorithms that take into account all the information present in the graph (corresponding to edge and node attributes in the various layers), as far as we know, do not exist. We thus use domain knowledge from our team members in order to build characteristic investor dimensions in order to create meaningful investors clusters. A paragraph has been added in the *Introduction* to address this point.

2. **The Hellinger-based similarity measure plays a key role in the performance of community detection algorithm. There are many indices for the measures of distance and the similarity. such as the Manhattan distance measure and cosine similarity measure. Please explain the reasons for adopting the distance measure and similarity measure defined in Eq.(2) and Eq.(3), respectively.**

- Unlike for instance the Kullback-Leibler divergence, the Hellinger distance has all the properties of a distance measure. Here, we compute the distance between two low-dimensional probability distributions. Cosine distance (or similarity) is better suited for similarity measures in high-dimensional vector spaces. A number of other distance metrics exist, with the most frequently used being the Minkowski distance. The Minkowski distance, however, has been known to exhibit unintuitive behaviour in certain cases when comparing two probability distributions that the Hellinger distance does not show (see [1] for a more in-depth discussion). We thus decided to use the Hellinger distance for our study, as is often done when comparing probability vectors [2, 3]. We do note, however, that our results still qualitatively hold when using the Euclidean distance. A sentence has been added in the *Hellinger distance and investor similarity* subsection to justify this choice of distance.

3. **English writing and grammar of the manuscript needs improvement. For instance, on page 7, line 105-106, the sentence "The log-binning was chosen as startup funding round amounts have been shown to follow a power-law distribution" is grammatically incorrect.**

- This specific sentence has been reworded, and the manuscript has been reviewed.

4. A number of labels of the figures are missing, for example, on Page 4, line 65, "see Fig. ??". Please check this throughout the manuscript.

- This was a LaTeX compilation problem that has now been corrected, our thanks to the reviewer.

5. **On the third last line of Page 4, *interacton* $\longrightarrow$ *interaction***

- This has been corrected, our thanks to the reviewer.

1. There are many clustering algorithms. Is your research an application of existing clustering algorithms to empirical evidence? Please clearly state your innovation, especially in terms of algorithm improvement or the advantages of your chosen clustering algorithm for the bipartite graph of funding interactions between investors and startups.

- As the investors in our network are highly heterogeneous, we believe that a suitable methodology to cluster similar investors is necessary in order to study investment dynamics. The investor-startup network, however, is a complex bipartite multilayer multigraph, meaning that investors are multi-dimensional items defined along the various axes of the graph. Efficient clustering algorithms that take into account all the information present in the graph (corresponding to edge and node attributes in the various layers), as far as we know, do not exist. We thus use domain knowledge from our team members in order to select characteristic investor dimensions in order to create meaningful investors clusters. A paragraph has been added in the *Introduction* to address this point.

2. Please add the review of the literature about clustering algorithms of the bipartite graph and compare what advantages of the clustering method you choose in this paper.

- Techniques to perform clustering on multi-view data exist [4, 5, 6], but are not able to deal with our specific constraints : our data is fundamentally bipartite, with each of the views containing different types of data (numerical vs. categorical vs. logarithmic) that is either node-based or edge-based. A generic method to deal with complex data of this format does not exist. Furthermore, our approach also has the added benefit of requiring a single parameter, which is the pruning threshold for the graph, and does not constraint the number of classes outputted. A paragraph has been added in the *Introduction* to address this point, and modifications have been made to the *Objective* section.

3. When you built investor-startup network (Fig 1.), you said "As an investor can invest in a startup several times, multiple edges can connect two given nodes as represented by the arcs." Is there any difference between the arcs and edges? Do you treat them differently in the empirical? Why don't you use a weighted network to describe an investor invest in a startup several times?

- The terms "arcs" and "edges" in this context describe the same thing, and so this has been corrected to only use a single term. A weighted network would not be suitable here as each individual edge contains important information such as the date of the investment, the funding amount of the investment and

the stage of the investment; a multigraph is thus better-suited.

4. Why you built upon these 5 characteristic dimensions? What are your reasons for considering this, and please explain them clearly.

- These 5 characteristic dimensions were determined through the domain knowledge of some of our team members that have worked in entrepreneurial ecosystems for over 20 years. Each of them represent a different investor facet, and correspond to characteristics related to the commonly agreed types of investors in entrepreneurship practice and research. This is detailed in the introduction of the *Investor characterization* subsection.

5. In the conclusion you state only some of the phenomena you have observed through your study. You should state the practical implications of your study or how your conclusions will be applied in finance.

- We provided additional information on the impact of our work, both in terms of practical implications for financial actors and in terms of research implications for academics studying venture capital.

References

[1] Legendre P, Legendre L. Numerical ecology. Elsevier, 2012.

[2] Sohangir S, Wang D. Improved sqrt-cosine similarity measurement. Journal of Big Data, 4(1), 1-13.

[3] Zhu S, Lizhao L, Wang Y. Information retrieval using Hellinger distance and sqrt-cos similarity. 7th International Conference on Computer Science & Education (ICCSE) (pp. 925-929). IEEE 2012.

[4] Wang H, Yang Y, Li B, Fujita H. A study of graph-based system for multi-view clustering. Knowledge-Based Systems, 2019, vol. 163, p. 1009-1019.

[5] Li L, He H. Bipartite graph based multi-view clustering. IEEE Transactions on Knowledge and Data Engineering, 2020

[6] Yang, Y, Wang H. Multi-view clustering: A survey. Big Data Mining and Analytics 1.2 (2018): 83-107.
