# Supplementary material for: A novel clustering approach to bipartite investor-startup networks
Source: PLoS One. 2023 Jan 5;18(1):e0279780. doi: 10.1371/journal.pone.0279780 (PMC9815571; doi:10.1371/journal.pone.0279780)
Supplement: Attachment — Submitted filename: review for PONE-D-22-23540.docx [file pone.0279780.s002.docx]

Reports

This paper propose a similarity-based clustering approach to venture capital investors that takes as input the bipartite graph of funding interactions between investors and startups and returns clusterings of investors built upon 5 characteristic dimensions. Although this study is comprehensive and the results are interesting, I think that the paper needs a substantial revision before assessing whether it should be considered for publication. I give detailed comments below.

1. There are many clustering algorithms. Is your research an application of existing clustering algorithms to empirical evidence? Please clearly state your innovation, especially in terms of algorithm improvement or the advantages of your chosen clustering algorithm for the bipartite graph of funding interactions between investors and startups.

2. Please add the review of the literature about clustering algorithms of the bipartite graph and compare what advantages of the clustering method you choose in this paper.

3. When you built investor-startup network (**Fig 1.**), you said "As an investor can invest in a startup several times, multiple edges can connect two given nodes as represented by the arcs." Is there any difference between the arcs and edges？Do you treat them differently in the empirical？Why don't you use a weighted network to describe an investor invest in a startup several times？

4. Why you built upon these 5 characteristic dimensions? What are your reasons for considering this, and please explain them clearly.

5. In the conclusion you state only some of the phenomena you have observed through your study. You should state the practical implications of your study or how your conclusions will be applied in finance.
